# Estimates of the national burden of respiratory syncytial virus in Kenyan children aged under 5 years, 2010–2018

**DOI:** 10.1186/s12916-023-02787-w

**Published:** 2023-03-31

**Authors:** Bryan O. Nyawanda, Nickson Murunga, Nancy A. Otieno, Godfrey Bigogo, Joyce U. Nyiro, Elisabeth Vodicka, Marc Bulterys, D. James Nokes, Patrick K. Munywoki, Gideon O. Emukule

**Affiliations:** 1 Kenya Medical Research Institute, Centre for Global Health Research, Kisumu, Kenya; 2 Kenya Medical Research Institute–Wellcome Trust Research Programme, Kilifi, Kenya; 3 PATH, Seattle, USA; 4 US Centers for Disease Control and Prevention, Nairobi, Kenya

**Keywords:** Burden, Epidemiology, National estimates, Pneumonia, Respiratory syncytial virus

## Abstract

**Background:**

Respiratory syncytial virus (RSV) is among the leading childhood causes of viral pneumonia worldwide. Establishing RSV-associated morbidity and mortality is important in informing the development, delivery strategies, and evaluation of interventions.

**Methods:**

Using data collected during 2010–2018 from base regions (population-based surveillance studies in western Kenya and the Kilifi Health and Demographic Surveillance Study), we estimated age-specific rates of acute respiratory illness (ARI), severe acute respiratory illness (SARI—defined as hospitalization with cough or difficulty breathing with onset within the past 10 days), and SARI-associated deaths. We extrapolated the rates from the base regions to other regions of Kenya, while adjusting for risk factors of ARI and healthcare seeking behavior, and finally applied the proportions of RSV-positive cases identified from various sentinel and study facilities to the rates to obtain regional age-specific rates of RSV-associated outpatient and non-medically attended ARI and hospitalized SARI and severe ARI that was not hospitalized (non-hospitalized SARI). We applied age-specific RSV case fatality ratios to SARI to obtain estimates of RSV-associated in- and out-of-hospital deaths.

**Results:**

Among Kenyan children aged < 5 years, the estimated annual incidence of outpatient and non-medically attended RSV-associated ARI was 206 (95% credible interval, CI; 186–229) and 226 (95% CI; 204–252) per 1000 children, respectively. The estimated annual rates of hospitalized and non-hospitalized RSV-associated SARI were 349 (95% CI; 303–404) and 1077 (95% CI; 934–1247) per 100,000 children respectively. The estimated annual number of in- and out-of-hospital deaths associated with RSV infection in Kenya were 539 (95% CI; 420–779) and 1921 (95% CI; 1495–2774), respectively. Children aged < 6 months had the highest burden of RSV-associated severe disease: 2075 (95% CI; 1818–2394) and 44 (95% CI 25–71) cases per 100,000 children for hospitalized SARI and in-hospital deaths, respectively.

**Conclusions:**

Our findings suggest a substantial disease burden due to RSV infection, particularly among younger children. Prioritizing development and use of maternal vaccines and affordable long-lasting monoclonal antibodies could help reduce this burden.

**Supplementary Information:**

The online version contains supplementary material available at 10.1186/s12916-023-02787-w.

## Background

Respiratory syncytial virus (RSV) is among the leading causes of acute lower respiratory infections (ALRI) worldwide, especially among younger children. Data suggest that the highest burden of RSV disease is in low- and middle-income countries (LMIC) [1–5]. While RSV infection is associated with mild disease in adults, it can cause severe disease leading to hospitalization and even death in younger children [6, 7]. Recent estimates suggest that, RSV is associated with 33.0 (25.4–44.6) million ALRI episodes, 3.6 (2.9–4.6) million hospitalizations, 26,300 (15,100–49,100) in-hospital deaths, and 101,400 (84,500–125,200) overall deaths in children aged < 5 years worldwide and approximately half of the deaths occur in infants younger than 6 months [5]. More than 97% of these deaths occur in LMICs [5].

An effective maternal vaccine against RSV could have a major impact on respiratory outcomes due to RSV. While there are no licensed maternal vaccines yet, promising candidate vaccines and new monoclonal therapies that would potentially offer protection to infants who bear the highest burden of severe RSV disease are undergoing evaluation [8–10]. Although a recent multi-site evaluation of a nanoparticle maternal RSV vaccine missed its primary endpoints [11], clinical trials of one prefusion F protein candidate (NCT04605159, NCT04980391, NCT05229068) were discontinued in February 2022, while another prefusion F protein subunit candidate (NCT04032093) [12] may be close to licensure after the US Food and Drugs Administration (FDA) granted it a Breakthrough Therapy designation in March 2022. Currently, a monoclonal antibody (palivizumab) is available and its use reduces RSV related hospitalizations by 50%; however, its cost limits its usage to very high-risk newborns in well-resourced settings only [10, 13, 14]. More recently, in November 2022, nirsevimab (a long acting mAb, NCT03979313) [15] was approved for use in the UK and the European Union, while other monoclonal antibodies (mAbs) and maternal vaccines are still in development [12]. As these vaccines and preventive therapeutics become available, policy makers will benefit from age-specific disease burden data to help with local decision making and prioritization of their introduction.

In Kenya, several studies have been carried out, mainly in three regions (Nairobi, Coast, and Nyanza), to estimate RSV-associated disease incidence [9, 16–19]. While burden estimates from these regions were comparable, these results cannot be generalized to other regions because of differences in the prevalence of risk factors for acute respiratory illness (ARI) and severe acute respiratory illness (SARI), underlying population structures, health-seeking behavior, and access to healthcare. Risk factors for ARI in children aged < 5 years include malnutrition (weight for age *Z*-score ≤  − 2), household pollution (as indicated by use of solid fuels for cooking), mother’s education level, passive smoking, and HIV prevalence [20], whereas risk factors for SARI in this age group include malnutrition, low birthweight (< 2500 g), non-exclusive breastfeeding (during the first 4 months of life), household pollution, crowding (≥ 5 persons in a household), and HIV prevalence [21–23]. Further, these studies did not report incidence by fine age bands. As such, there is a need to estimate the national burden of RSV-associated disease in Kenya and delineate any variations by region and age. Here, we estimate the age-specific burden of medically (in-and outpatient) and non-medically attended RSV-associated acute respiratory illness, and in and out-of-hospital deaths associated with RSV infection among children aged < 5 years by regions and counties of Kenya.

## Methods

### Base-site description

To estimate the rates of outpatient burden and non-medically attended ARI for the base region (base rates, from where rates were extrapolated to the other regions), we used data previously collected by the Kenya Medical Research Institute – Center for Global Health Research (KEMRI–CGHR) with support from the US Centers of Disease Control and Prevention (CDC) in Siaya County, Nyanza region, western Kenya. Siaya County has high mortality among children aged < 5 years, as well as high malaria and HIV prevalence [24, 25]. Data collected from 2010 to 2018 among children aged < 5 years visiting St. Elizabeth Lwak Mission Hospital (LMH), which is the referral clinic for the Asembo Population-Based Infectious Disease Surveillance (PBIDS) platform, were used to estimate the Nyanza region ARI base rates [26]. Asembo PBIDS is located within the Siaya Health and Demographic Surveillance System (HDSS) [27], a comprehensive surveillance program for evaluating disease burden/indicators and impact of public health interventions. The PBIDS covers the population within a 5-km radius from LMH; here, the residents were asked about children’s history of ARI in the last 14 days from the date of interview and health-seeking patterns [26, 28]. ARI was defined as an acute respiratory infection (onset within 10 days from interview date) with cough, difficulty breathing, sore throat, or runny nose.

**Fig. 1:**
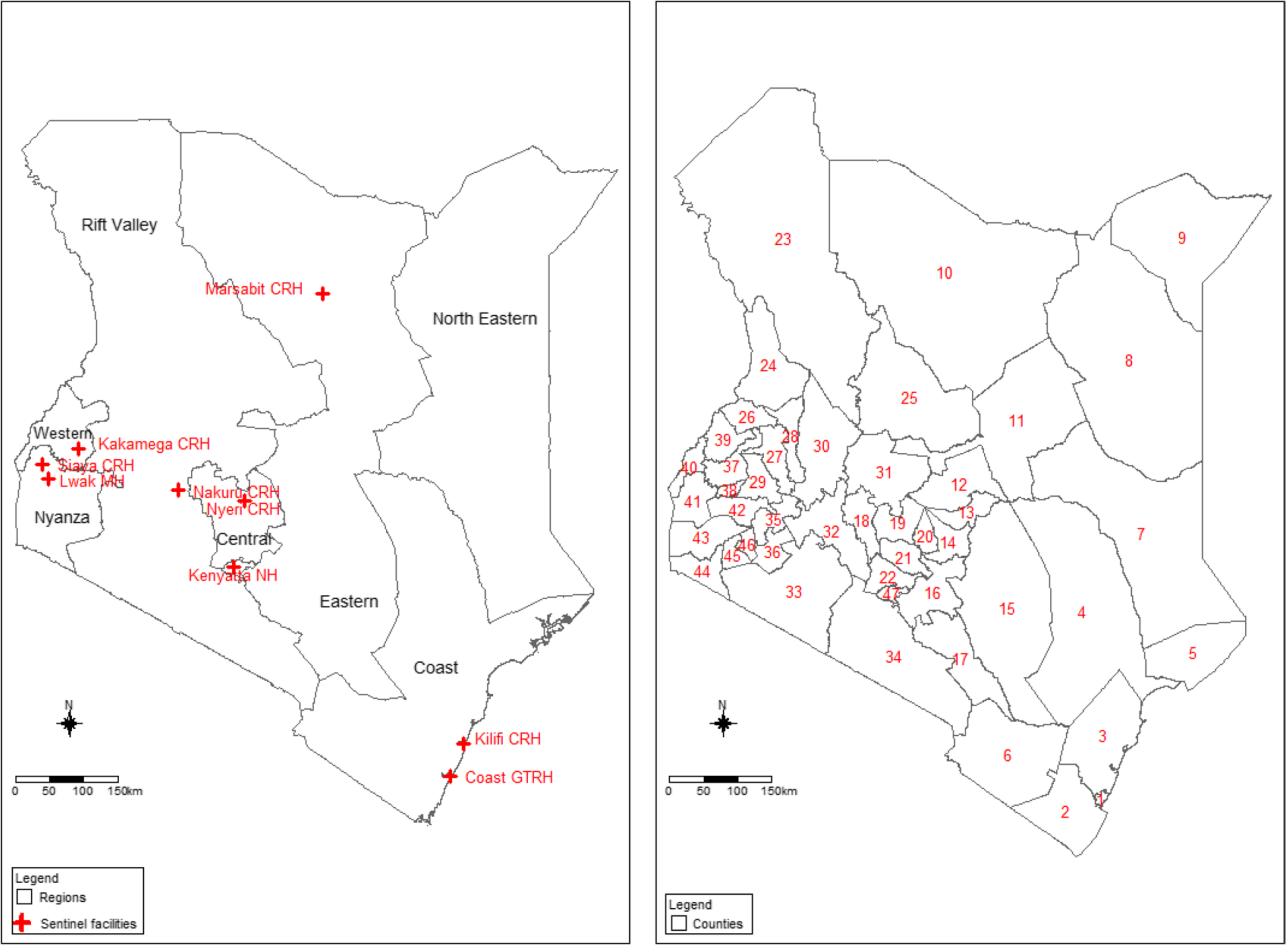
Map showing the regions and RSV surveillance sites (Left) and county outlines (right): 1—Mombasa, 2—Kwale, 3—Kilifi, 4—Tana River, 5—Lamu, 6—Taita Taveta, 7—Garissa, 8—Wajir, 9—Mandera, 10—Marsabit, 11—Isiolo, 12—Meru, 13—Tharaka Nithi, 14—Embu, 15—Kitui, 16—Machakos, 17—Makueni, 18—Nyandarua, 19—Nyeri, 20—Kirinyaga, 21—Murang’s, 22—Kiambu, 23—Turkana, 24—West Pokot, 25—Samburu, 26—Trans Nzoia, 27—Uasin Gishu, 28—Elgeyo Marakwet, 29—Nandi, 30—Baringo, 31—Laikipia, 32—Nakuru, 33—Narok, 34—Kajiado, 35—Kericho, 36—Bomet, 37—Kakamega,38—Vihiga,39—Bungoma, 40—Busia, 41—Siaya, 42—Kisumu, 43—Homabay, 44—Migori, 45—Kisii, 46—Nyamira, 47—Nairobi

For the estimation of the base rates of hospitalized SARI and severe ARI that was not hospitalized (non-hospitalized SARI), data collected through pediatric surveillance of RSV at the Kilifi County Referral Hospital (CRH) from 2010 to 2018 were used. This surveillance platform is a component of HDSS run by KEMRI–Center for Geographic Medical Research Coast (CGMR–C) funded by the Welcome Trust, UK. Kilifi County has low per capita income, malaria is endemic, and < 5 year and infant mortality ratios are high at 41 and 28.2 per 1000 live births, respectively [29, 30]. We defined SARI as hospitalization with an acute respiratory infection (i.e., cough or difficulty breathing with an onset within the past 10 days from interview date). The location of the base sites, sentinel facilities, regions, and counties are presented in Fig. 1.

### Estimation of RSV-associated outpatient ARI

**Fig. 2:**
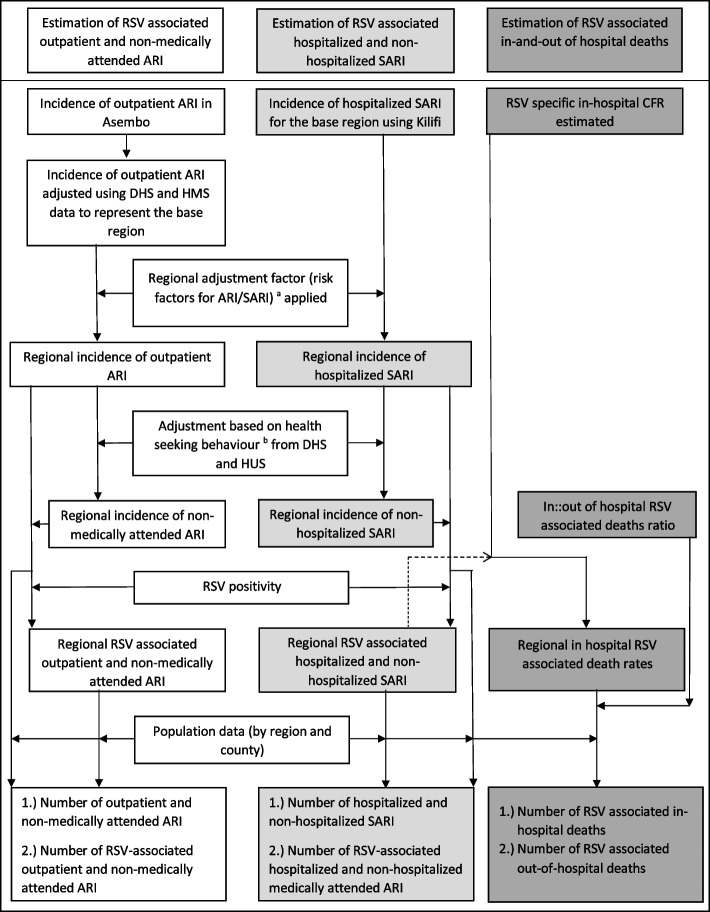
Flow chart outlining steps followed in estimating the burden of RSV-associated disease and deaths

The steps used to estimate RSV-associated outpatient ARI are summarized in Fig. 2. We estimated the age-specific annual rates of outpatient ARI for the Asembo PBIDS by dividing the age-specific number of ARI cases by the age-specific population of PBIDS participants. To account for potential variation in health-seeking behavior among patients attending LMH vs. other facilities in the study area, this rate was adjusted by applying the proportion of those with ARI seeking care at any facility to the LMH-specific rates (Additional file 1: Eq. 1). Given that the PBIDS participants are offered free treatment for ARI at LMH, the adjusted rates may not be generalizable to the entire Nyanza region (“base region”). To estimate the outpatient ARI in the base region, we therefore multiplied the rates by the Kenya Demographic and Health Survey (KDHS) health-seeking estimate for the entire base region divided by the health-seeking estimate from the PBIDS (Additional file 1: Eq. 2). Rates of outpatient ARI in the other 7 regions were then calculated by first estimating region-specific adjustment factors based on the relative risk of known risk factors for ARI (Additional file 1: Eq. 3) obtained from Tazinya et al. [20]. The adjustment factors also incorporated the region-specific prevalence of these risk factors, as reported from the demographic and health survey conducted in 2014, and the region-specific health-seeking behavior for ARI as summarized in Additional file 2: Table S1a [31, 32]. We then applied the region-specific adjustment factors to the ARI base rate to estimate the region-specific outpatient ARI rates (Additional file 1: Eq. 4). Similar approaches have previously been used to extrapolate disease burden rates from one base region to the national level [21, 22].

Data on RSV testing at LMH, from nasopharyngeal (NP) and/or oropharyngeal (OP) swabs collected from outpatients who presented with ARI, were available up to 2014. We aggregated this over the years stratified by age categories to estimate age-specific proportions of RSV positive ARI between 2015 and 2018. These proportions were applied to the ARI rates to estimate the age-specific RSV-associated outpatient ARI rate in the base region (Additional file 1: Eq. 6). Data on RSV testing were not available for any other outpatient facility; we therefore generated an ARI to SARI RSV positivity ratio from the base region (i.e., age-specific percent RSV-positive ARI from LMH divided by age-specific percent RSV-positive SARI from LMH and Siaya CRH), which was applied to the proportion of RSV-positive SARI estimated for the other regions to obtain the proportion of RSV-positive ARI for each region assuming that this ratio would be relatively similar across regions. We applied the regional-age-specific proportion RSV positive to the regional rates of outpatient ARI to obtain the regional age-specific rates of RSV-associated outpatient ARI (Additional file 1: Eq. 6).

### Estimation of RSV-associated non-medically attended ARI

We used 2018 data from a Health Utilization Survey (HUS) conducted in Siaya County—Nyanza region, Nakuru County—Rift Valley region, Kakamega County—Western region, and Marsabit County—Eastern region, to estimate the proportion of ARI cases seeking care https://bmcpublichealth.biomedcentral.com/articles/10.1186/s12889-023-15252-3. For regions where the HUS was not conducted, we extrapolated the proportion of ARI cases seeking care from Siaya County by multiplying the Siaya proportion of ARI cases seeking care by region-specific proportion of individuals seeking care for ARI (as defined in KDHS—cough and difficulty breathing > 2 days) divided by the proportion of individuals seeking care for ARI (as defined in KDHS) in Nyanza. To estimate the regional-age-specific rates of non-medically attended ARI, we divided the regional-age-specific rates of outpatient ARI by the region-specific proportion of ARI patients who sought care as estimated from the HUS and KDHS ARI [31, 32] then subtracted the rate of outpatient ARI (Fig. 2 and Additional file 1: Eq. 5). Assuming similar RSV positivity as outpatient RSV, we estimated the regional age-specific rates of non-medically attended ARI associated with RSV by applying the regional age-specific proportion positive for RSV to the regional rates of non-medically attended ARI (Additional file 1: Eq. 7).

### Estimation of RSV-associated hospitalized SARI

Age-specific annual base rates (Coastal region) of hospitalized SARI were estimated by dividing age-specific number of hospitalized SARI cases from Kilifi HDSS residents at the Kilifi CRH by the age-specific population of Kilifi HDSS participants (Fig. 2 and Additional file 3: Eq. 1). This rate was adjusted to include residents hospitalized elsewhere (~ 20%) using admissions data for inpatient hospitals within the Kilifi HDSS catchment area available from the District Health Information Software 2 (DHIS2) (https://dhis2.org/). Regional rates of hospitalized SARI in the other 7 regions relied on a region-specific adjustment factor (Additional file 3: Eq. 2) based on the relative risk and regional prevalence of risk factors for SARI. The region-specific adjustment factor was then applied to the SARI base rate to estimate the region-specific hospitalized SARI rates (Additional file 3: Eq. 3).

To estimate the proportion of SARI cases positive for RSV, we divided the number of RSV-positive cases by SARI cases. NP and/or OP samples obtained from patients hospitalized with SARI at Kilifi and Siaya CRHs were systematically tested for RSV and therefore represented the Coastal and Nyanza regions respectively. Samples were only intermittently tested for RSV in the other regions; we therefore used data from Siaya CRH together with Kakamega CRH for the Western region. We used data from Kenyatta National hospital (NH) and Nakuru and Nyeri CRHs to estimate the proportions for Rift Valley, Central, and Nairobi regions (Fig. 1). For Eastern and Northeastern regions, we used the national average percent RSV positive from the SARI cases in the absence of region-specific data. Regional age-specific rates of hospitalized SARI associated with RSV were then estimated by applying the age-specific proportion RSV-positive cases among the hospitalized SARI patients to the regional age-specific rates of hospitalized SARI, (Additional file 3: Eq. 6).

### Estimation of RSV-associated non-hospitalized SARI

Using the HUS data described above (unpublished data), we first estimated the proportion of reported pneumonia cases (SARI cases) that resulted in hospitalization—defined as hospitalized cases of a reported episode of respiratory illness with cough and difficulty breathing for > 2 days or a clinician’s diagnosis of pneumonia within the past 12 months [33]. For regions where the HUS was not conducted, we extrapolated the proportion of hospitalized SARI cases from Siaya County by multiplying the Siaya proportion of hospitalized SARI by region-specific proportion of individuals seeking care for ARI divided by the proportion of individuals seeking care for ARI in Nyanza (Fig. 2 and Additional file 3: Eq. 5). Non-hospitalized SARI cases were those who reported an episode of pneumonia as defined above with a clinician’s recommendation for hospitalization or presentation with any one of the danger signs (inability to breastfeed or drink, persistent vomiting, convulsions or seizures, loss of consciousness), yet they were not hospitalized. The rate of non-hospitalized SARI was estimated by dividing the regional-age-specific rates of hospitalized SARI by the region-specific proportion of hospitalized SARI then subtracting the rate of hospitalized SARI (Additional file 3: Eq. 4). Assuming similar proportion of RSV positivity for non-hospitalized SARI as for hospitalized SARI, regional-age-specific rates of non-hospitalized RSV-associated SARI were estimated by applying the age-specific proportion of RSV positivity among the hospitalized SARI cases to the regional age-specific rates of non-hospitalized SARI (Additional file 3: Eq. 7).

### Estimation of RSV-associated in- and out-of-hospital deaths

RSV-associated in-hospital deaths were estimated by first calculating the age-specific RSV-associated case fatality rate (CFR) using data from Kilifi and Siaya and then applying the age-specific CFRs to regional-age-specific hospitalized SARI rates associated with RSV infection (Fig. 2) to get the RSV-associated mortality rate. We then obtained the number of in hospital deaths by applying the age-specific RSV-associated mortality rate to the age-specific population. Given that data on out-of-hospital deaths are missing for Kenya, we first derived multipliers from in and out of hospital RSV-associated deaths by age groups (0–5, 6–11, 12–59, and 0–59 months) from LMIC estimates by Li et al. [5]. We then applied those age-specific multipliers to the age-specific in-hospital deaths to obtain the number of out-of-hospital deaths. The age-specific in-hospital CFRs and derived age-specific out- and in-hospital multipliers are summarized in Additional file 2: Table S1c.

### Number of RSV-associated ARI and SARI cases by counties

The county-specific annual numbers of outpatient and non-medically attended ARI and their respective RSV-associated cases (Additional file 1: Eq. 8–11) and the hospitalized and non-hospitalized SARI and RSV-associated SARI cases (Additional file 3: Eq. 8–11) were estimated by applying the region-specific rates to the population of each county in the region. The national, regional, and county population data were obtained from the 2019 national census data [34] and extrapolated for the other years using the World Bank annual percentage growth rate for Kenya [35]. Since census data were only available in broader age categories, we applied population proportions from Siaya and Kilifi HDSS to the census data to obtain fine age bands used in this study. County borders are displayed in Fig. 1.

### Statistical analysis

To estimate adjustment factors to apply to base rates, we estimated the prevalence of each risk factor by running 1000 iterations while allowing it to vary within a binomial distribution. The 50th, 2.5th, and 97.5th percentile values were reported as the estimate and 95% CIs, respectively. ARI rates are reported per 1000 population, whereas SARI and death rates are reported per 100,000 population. Age groups included monthly delineated categories up to 11 months, 12–14 months, 15–17 months, 18–20 months, 21–23 months, 24–35 months, 36–47 months, and 48–59 months. Analyses were performed using Stata version 16 (StataCorp, College Station, Texas) and R version 3.6.3 (Vienna, Austria). Maps of counties, regions, and facilities were assembled using QGIS version 3.10.13 (https://qgis.org).

## Results

### Rates of ARI and SARI

Using data collected from 2010 through 2018, we estimated the national annual rate of outpatient and non-medically attended ARI among Kenyan children aged < 5 years as 1725 (95% CI 1577–1899) and 1896 (95% CI 1733–2088) cases per 1000 children respectively (Table 1). The national rates of hospitalized and non-hospitalized SARI were estimated as 2501 (95% CI 2182–2869) and 7717 (95% CI 6734–8855) cases per 100,000 children respectively (Table 2). The distribution by year and regions are provided in Additional file 2: Tables S2, S4, S6, and S8.

**Table 1:** Annual rate (per 1000) of outpatient and non-medically attended ARI and RSV-associated ARI in children < 5 years

Age (months)	Outpatient		Non-medically attended	
	ARI	RSV-associated ARI	ARI	RSV-associated ARI
	Rate (95% CI)	Rate (95% CI)	Rate (95% CI)	Rate (95% CI)
Fine age bands				
< 1	391 (357, 430)	38 (27, 51)	430 (392, 473)	42 (29, 56)
1	702 (644, 770)	93 (74, 116)	772 (707, 847)	102 (81, 127)
2	1214 (1096, 1331)	160 (125, 199)	1334 (1205, 1463)	175 (137, 219)
3	1689 (1547, 1858)	350 (289, 415)	1857 (1700, 2042)	385 (317, 457)
4	1982 (1821, 2173)	410 (343, 489)	2179 (2002, 2389)	450 (377, 538)
5	2229 (2032, 2455)	390 (322, 463)	2451 (2234, 2699)	428 (354, 509)
6	2445 (2245, 2677)	290 (229, 360)	2688 (2468, 2943)	318 (252, 396)
7	2489 (2281, 2727)	217 (164, 278)	2737 (2507, 2997)	239 (180, 306)
8	2361 (2159, 2591)	243 (182, 315)	2596 (2373, 2848)	268 (200, 346)
9	1910 (1748, 2095)	329 (267, 403)	2099 (1922, 2303)	362 (293, 443)
10	2087 (1910, 2301)	326 (265, 405)	2294 (2099, 2529)	359 (291, 446)
11	1840 (1683, 2032)	286 (221, 355)	2023 (1850, 2233)	315 (243, 390)
12–14	1753 (1602, 1935)	219 (183, 262)	1927 (1761, 2127)	241 (201, 288)
15–17	1972 (1807, 2165)	185 (145, 228)	2167 (1986, 2380)	204 (159, 251)
18–20	1975 (1796, 2171)	162 (122, 205)	2171 (1974, 2387)	179 (134, 225)
21–23	1766 (1616, 1944)	168 (126, 215)	1941 (1777, 2137)	185 (139, 236)
24–35	1882 (1725, 2076)	141 (114, 168)	2069 (1897, 2282)	156 (125, 185)
36–47	1840 (1684, 2028)	132 (102, 165)	2023 (1851, 2229)	145 (112, 181)
48–59	1756 (1605, 1939)	60 (39, 86)	1930 (1764, 2132)	66 (42, 95)
Broader age categories				
0–5	1313 (1204, 1448)	208 (184, 235)	1444 (1323, 1592)	229 (202, 258)
6–11	2202 (2008, 2412)	291 (256, 331)	2420 (2207, 2652)	320 (281, 363)
12–23	1844 (1685, 2027)	183 (160, 210)	2027 (1852, 2228)	201 (176, 230)
24–59	1815 (1653, 2001)	120 (102, 140)	1995 (1817, 2200)	132 (112, 154)
< 60	1725 (1577, 1899)	206 (186, 229)	1896 (1733, 2088)	226 (204, 252)

**Table 2:** Annual rate (per 100,000) of hospitalized and non-hospitalized SARI and RSV-associated SARI in children < 5 years

Age (months)	Hospitalized		Non–hospitalized	
	SARI	RSV-associated SARI	SARI	RSV-associated SARI
	Rate (95% CI)	Rate (95% CI)	Rate (95% CI)	Rate (95% CI)
Fine age bands				
< 1	7110 (6202, 8160)	2366 (2042, 2751)	21,939 (19,138, 25,179)	7302 (6301, 8489)
1	10,221 (8966, 11,818)	3420 (2911, 4012)	31,540 (27,667, 36,469)	10,553 (8983, 12,382)
2	7468 (6557, 8576)	2493 (2142, 2912)	23,044 (20,233, 26,463)	7692 (6611, 8986)
3	7180 (6261, 8241)	1516 (1297, 1790)	22,155 (19,321, 25,429)	4679 (4004, 5524)
4	6451 (5646, 7344)	1372 (1169, 1597)	19,905 (17,422, 22,663)	4234 (3607, 4928)
5	7110 (6221, 8116)	1510 (1288, 1766)	21,941 (19,196, 25,045)	4659 (3975, 5450)
6	6978 (6047, 8060)	1052 (877, 1269)	21,533 (18,660, 24,871)	3246 (2706, 3917)
7	6024 (5223, 6967)	916 (764, 1086)	18,590 (16,116, 21,498)	2825 (2357, 3353)
8	5646 (4863, 6459)	853 (714, 1023)	17,423 (15,008, 19,930)	2632 (2203, 3156)
9	4469 (3894, 5133)	513 (417, 618)	13,790 (12,017, 15,841)	1583 (1287, 1907)
10	4958 (4341, 5676)	575 (475, 685)	15,300 (13,395, 17,515)	1773 (1465, 2115)
11	4060 (3560, 4643)	465 (389, 567)	12,528 (10,985, 14,328)	1434 (1202, 1749)
12–14	3608 (3142, 4158)	383 (310, 466)	11,133 (9696, 12,830)	1183 (958, 1439)
15–17	2984 (2616, 3460)	285 (223, 362)	9207 (8071, 10,676)	878 (689, 1118)
18–20	3030 (2594, 3498)	318 (249, 399)	9350 (8005, 10,795)	982 (769, 1233)
21–23	2128 (1856, 2465)	184 (133, 238)	6565 (5727, 7605)	567 (412, 736)
24–35	1505 (1320, 1724)	120 (99, 148)	4643 (4073, 5319)	372 (304, 456)
36–47	972 (845, 1120)	58 (43, 75)	2998 (2608, 3455)	179 (133, 231)
48–59	569 (493, 653)	13 (7, 21)	1755 (1521, 2015)	41 (23, 63)
Broader age categories				
0–5	7601 (6662, 8702)	2075 (1818, 2394)	23,455 (20,556, 26,852)	6404 (5610, 7386)
6–11	5256 (4589, 6067)	705 (599, 824)	16,221 (14,160, 18,722)	2174 (1848, 2543)
12–23	2945 (2565, 3393)	294 (248, 351)	9088 (7917, 10,469)	907 (766, 1083)
24–59	1018 (886, 1164)	63 (52, 77)	3141 (2736, 3593)	195 (161, 236)
< 60	2501 (2182, 2869)	349 (303, 404)	7717 (6734, 8855)	1077 (934, 1247)

### Rates of outpatient and non-medically attended ARI associated with RSV

**Fig. 3:**
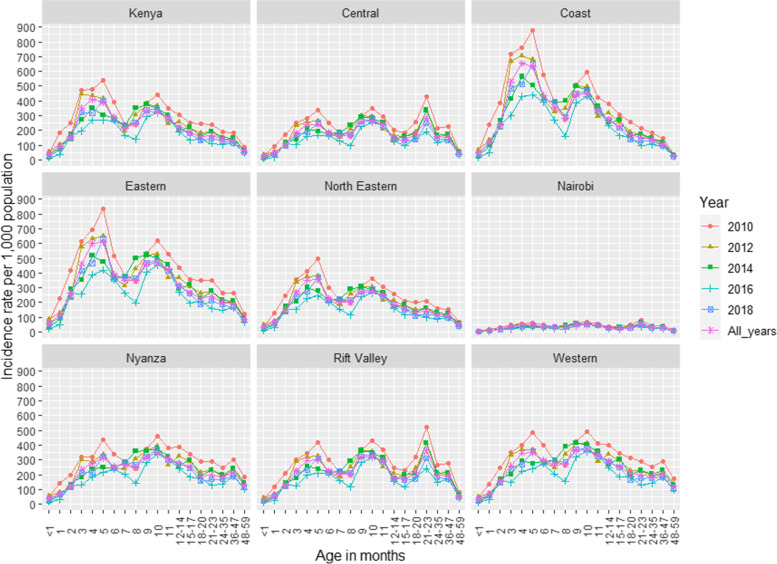
Annual rate (per 1000) of RSV-associated outpatient ARI by region, age and year

Among Kenyan children aged < 5 years, the average annual rate of RSV-associated outpatient ARI was 206 (95% CI 186–229) cases per 1000 children (Table 1). The highest rate of RSV-associated outpatient ARI was observed in children aged 4 months (410, 95% CI 343–489) followed by 5 months of age (390, 95% CI 322–463), whereas the lowest rate was observed among children aged 48–59 months (60, 95% CI 39–86). Coast (298, 95% CI 254–345) and Eastern regions (297, 95% CI 262–336) had the highest, whereas Nairobi had the lowest rates of outpatient RSV-associated ARI (29, 95% CI 16–45) cases per 1000 children (Fig. 3 and Additional file 2: Table S3). When considering the disease burden over time, the rate of RSV-associated outpatient ARI was lowest in 2015 – 137 (95% CI, 124–153) (Additional file 2: Table S3). The national annual rate of RSV-associated non-medically attended ARI was 226 (204–252) cases per 1000 children (Table 1).

### Rates of hospitalized and non-hospitalized SARI associated with RSV

**Fig. 4:**
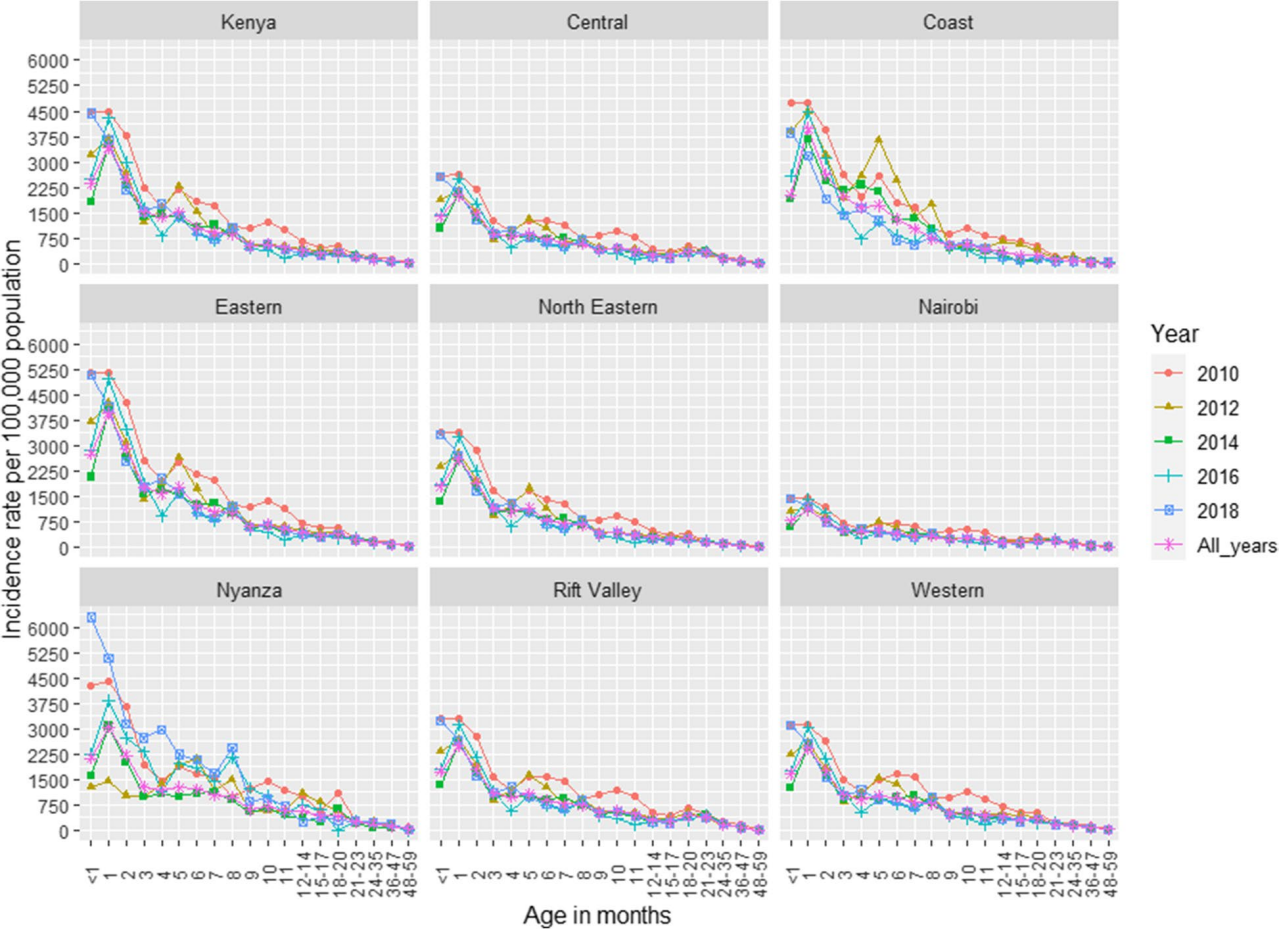
Annual rate (per 100,000) of RSV-associated hospitalized SARI by region, age, and year

The national annual rate of RSV-associated hospitalized SARI among children < 5 years of age was 349 (95% CI 303–404) per 100,000 children (Table 2). The highest rate was observed in 1-month-old infants at 3420 (95% CI 2911–4012) and the lowest in children aged 48–59 months at 13 (95% CI 7–21). The rate of RSV-associated hospitalized SARI steadily decreased with increase in age with the highest rates observed in younger age groups (< 6 months old and even higher among children aged < 3 months) and lowest in older children (Fig. 4). Eastern and Nairobi regions had the highest 400 (95% CI 338–474) and the lowest 138 (95% CI 84–214) annual rate of RSV-associated hospitalized SARI, respectively (Fig. 4 and Additional file 2: Table S7). The rates were highest in 2010—547 (95% CI 475–640)—and lowest in 2017—168 (95% CI 146–193) (Additional file 2: Table S7). The national annual rate of RSV-associated non-hospitalized SARI was estimated as 1077 (95% CI 934–1247) per 100,000 population (Table 2).

### Rates of RSV-associated deaths

The annual rate of RSV-associated in-hospital deaths per 100,000 children aged < 5 years was estimated at 9 (95% CI 7–13) in Kenya (Table 3). RSV-associated deaths declined with increasing age: the rate of RSV-associated in hospital deaths among infants aged < 6 months was 44 (95% CI 25–71) (Table 3).

**Table 3:** Annual average rate (per 100,000) and number of RSV-associated deaths among children < 5 years

Age in months	In-hospital deaths		Out-of-hospital deaths	Total deaths
	Rate (95% CI)	Number (95% CI)	Number (95% CI)	Number (95% CI)
0–59 months	9 (7, 13)	539 (420, 779)	1921 (1495, 2774)	2461 (1914, 3553)
0–5 months	44 (25, 71)	245 (139, 395)	372 (211, 600)	617 (350, 995)
6–11 months	29 (15, 46)	159 (82, 252)	723 (373, 1147)	882 (455, 1399)
12–23 months	6 (2, 12)	69 (23, 139)	174 (58, 346)	243 (81, 484)
24–59 months	2 (0, 3)	75 (0, 112)	187 (0, 280)	261 (0, 392)

### Number of RSV-associated ARI, SARI, and deaths by counties

Among Kenyan children aged < 5 years, the total annual number of RSV-associated outpatient ARI was estimated to be between 1,114,748 and 1,372,458, while the number of RSV-associated non-medically attended ARIs was estimated to be between 1,222,626 and 1,510,303 cases (Additional file 2: Table S10). The counties with most outpatient ARI cases were Kilifi, Meru, and Nakuru, while Samburu, Kirinyaga, and Lamu had the lowest, in that order. For non-medically attended ARI, Mandera, Wajir, and Kakamega were the leading counties, while Samburu, Kirinyaga, and Lamu had the least cases in that order.

The annual number of RSV-associated hospitalized SARI cases was estimated to be between 18,160 and 24,213, whereas the number of RSV-associated non-hospitalized SARI cases was 55,977 to 74,736 (Additional file 2: Table S10). Nakuru, Kilifi, and Nairobi had the highest number, while Lamu, Kirinyaga, and Taita–Taveta had the least number of RSV-associated hospitalized SARI in that order. We also estimated that the highest number of RSV-associated non-hospitalized SARI were highest in Nakuru, Mandera, and Kilifi and lowest in Lamu, Kirinyaga, and Vihiga counties.

Further, we estimate the annual number of RSV-associated deaths to be between 1914 and 3553 cases with in-hospital and out-of-hospital deaths ranging from 420 to 779 and from 1495 to 2774 cases, respectively (Additional file 2: Table S10). Nakuru County had the highest number of RSV-associated deaths, whereas Lamu County had the lowest number of RSV-associated deaths.

## Discussion

RSV has been shown to be the most common virus causing severe pneumonia among children in Africa and Asia [36, 37]. Our estimates suggest that during 2010–2018, the average annual rate of outpatient and non-medically attended ARI that was associated with RSV among children aged < 5 years ranged between 186 and 229 (outpatient) and between 204 and 252 (non-medically attended) cases per 1000 children, respectively. We also estimated that 303 to 404 per 100,000 children were hospitalized with RSV-associated SARI annually. Further, we estimated that RSV was associated with 1914 to 3553 deaths annually among children aged < 5 years in Kenya. Our results also show that the highest burden of RSV-associated morbidity and mortality was within the first 6 months of life, which suggests that RSV prevention measures targeting younger infants could have the greatest impact on reducing RSV-associated disease burden.

Our RSV-associated ARI estimates were higher than estimates reported in two studies conducted in Western Kenya and a multi-country study among patients with influenza like illness (ILI) [16, 18, 38]. These differences may be due to the difference in case definitions used. Unlike the current study, these studies included fever in the case definition. It has been shown that case definitions which include fever have low sensitivity of detecting RSV [39, 40]; therefore, the estimates from the current study may be more reflective of the RSV disease burden compared to the previous estimates. Our estimates of RSV-associated ARI were higher than earlier observed by Okiro et al. in Kilifi [41], and this difference may be due to our use of adjustment factors which account for health-seeking in facilities other than the study facility for medically attended ARI and implies that Okiro et al. may have underestimated the RSV-associated ARI burden. When comparing our results to other RSV burden studies in Africa, our estimates among infants were 44% lower than those observed among Malian infants with ILI/pneumonia [42]. This difference likely reflects a difference in study designs; whereas our study uses data from passive surveillance, in the Malian study, infants were actively followed every week to detect ILI and pneumonia. Importantly, maternal vaccination reduces the burden of RSV among infants through maternal antibody transfer to the newborns that confer protection during their first 6 months of life. Our findings suggest that this is a critical period for age-targeted prevention technologies.

We estimated that the mean annual rate of RSV-associated hospitalized SARI was 303–404 cases per 100,000 children in Kenya. A previous study conducted in Western Kenya and two studies in the Coastal region had comparable results of 400–680 cases per 100,000 children in the 2009–2012 period [18], 271–317 cases per 100,000 children annually [9] in the 2002–2007 period, and 535 cases per 100,000 children annually in 2007 [43]. Further, our SARI results were comparable to results from a study in Madagascar that reported 295–349 cases per 100,000 children (Rabarison et al., 2019) but lower when compared to findings from South Africa where they report 960–1180 cases per 100,000 children during 2010–2011 (Moyes et al., 2013). It is important to note that a substantial proportion of SARI cases do not get hospitalized in Sub-Saharan Africa [46], and data on unattended SARI is limited. Distance from home to health facilities is one of the leading barriers to healthcare seeking [28]. Extrapolating from the healthcare utilization surveys (unpublished data), we determined that the rates of RSV-associated non-hospitalized SARI were threefold higher than hospitalized rates. These findings suggest that advancing the development and delivery of effective prevention measures, such as maternal vaccination, is critical for reducing the burden of medically unattended RSV.

In this study, we found that RSV CFR among children aged < 5 years was 2.6% of hospitalized SARI which translated to 420–779 in-hospital deaths annually. Using in- and out-of-hospital RSV-associated deaths ratio derived from LMIC estimates from Li et al. [5], we estimate that 1495–2774 RSV-associated deaths occur out of hospital annually. Our RSV-associated mortality rate among children aged < 5 years was comparable to earlier studies in Western Kenya and South Africa [47–49] but higher compared to rates observed in America and England [50–52], probably due to the differences in the quality of care provided in these settings. Recent estimates suggest that most RSV-associated deaths occur out of hospital [5, 53]. Preventive measures such as maternal immunization (when available), cocooning strategies [54], or use of mAbs could help in reducing RSV-associated deaths.

We observed that the highest rates of RSV-associated ARI, SARI, and deaths were among children aged < 6 months. Similar findings have been reported in studies conducted in Africa and elsewhere [1, 9, 45, 55]. We also observed that RSV circulation has been relatively the same over the years. RSV epidemics (periods of significantly increased activity) have previously been shown to have consistent patterns occurring around the same months across different years; however, these patterns differ by regions in Kenya [3, 18, 56]. Nakuru, Kilifi, and Nairobi were among the leading counties with RSV-associated morbidity and mortality. Unlike Nakuru in the Rift Valley region and Kilifi in the Coastal region, Nairobi had the lowest rate of RSV-associated disease; similar findings have been reported for influenza in Kenya [21, 22]. If a national maternal vaccination program were to be implemented in Kenya, timing for vaccination campaigns could be considered 1–2 months before the onset of the RSV season in the different regions.

When interpreting our results, several limitations should be noted. First, data by the finer age categories as used in this study were not available from the published population census data; we therefore used proportions from the Siaya and Kilifi HDSS which may not be similar to the population distribution by age categories in other regions. Second, as much as we used known ARI and SARI risk factors and health-seeking behavior from the demographic and health survey to account for potential geographical differences, these might not necessarily capture all geographical variability. This may explain why we observed low rates of RSV-associated disease in Nairobi region (an urban area where the prevalence of the risk factors were low) compared to the other regions. However, a previous population-based study comparing RSV-associated SARI rates between Lwak (rural Nyanza region) and Kibera (urban informal settlement in Nairobi region) observed significantly higher rates of RSV-associated SARI among children aged < 5 years in the rural setting compared to the urban setting [16]. Third, RSV testing was not routinely done in our outpatient settings throughout the analysis period, and such data were missing entirely for most of the regions. Our use of similar RSV prevalence for estimation of year-to-year RSV-associated ARI may not be accurate since we expect annual variations in RSV prevalence. Lastly, assuming that the in- and out-of-hospital RSV-associated deaths ratio for LMICs applies to Kenya should be interpreted with caution as access to healthcare varies from one-country to another, the use of country-specific data will be more appropriate for similar analyses.

## Conclusions

Our estimates suggest a substantial burden of RSV-associated illness and deaths in Kenya. The evidence from this study is consistent with other studies which have reported the highest burden of RSV-associated illness during the first 6 months of life, with more severe outcomes (hospitalization and death) in the first 3 months of life. Prioritizing prevention measures that target early infancy such as maternal vaccination and use of mAbs could potentially help in reducing this burden, should the vaccines and affordable long-lasting mAbs become available. In the meantime, promotion of preventive measures such as hand washing and cocooning among other measures to prevent transmission of respiratory illnesses could be prioritized. Finally, estimates generated from this work could be useful for cost-effectiveness analyses of different immunization strategies.

## Supplementary Information

**Additional file 1. **Methods for estimating RSV associated acute respiratory illness (ARI).

**Additional file 2. Table S1a.** Regional prevalence of risk factors for acute respiratory illness (ARI) and healthcare seeking behaviour for ARI and relative risks for the risk factors, Kenya. **Table S1b.** Regional prevalence of risk factors for severe acute respiratory illness and healthcare seeking behaviour for acute respiratory illness and relative risks for the risk factors, Kenya.  **Table S1c.** Age-specific in-hospital RSV case fatality rate (CFR) and the out:in hospital RSV deaths multiplier. **Table S2.** Annual rates of outpatient acute respiratory illness per 1000 children by regions and age in months, 2010-2018. **Table S3.** Annual rates of outpatient RSV associated acute respiratory illness per 1000 children by regions and age in months, 2010-2018. **Table S4.** Annual rates of non-medically attended acute respiratory illness per 1000 children by regions and age in months, 2010-2018. **Table S5.** Annual rates of RSV associated non-medically attended acute respiratory illness per 1000 children by regions and age in months, 2010-2018. **Table S6.** Annual rates of hospitalized severe acute respiratory illness per 100,000 children by regions and age in months, 2010-2018. **Table S7.** Annual rates of RSV associated-hospitalized severe acute respiratory illness per 100,000 children by regions and age in months, 2010-2018. **Table S8.** Annual rates of non-hospitalized severe acute respiratory illness per 100,000 children by regions and age in months, 2010-2018. **Table S9.** Annual rates of RSV associated non-hospitalized severe acute respiratory illness per 100,000 children by regions and age in months, 2010-2018. **Table S10.** Annual average number of RSV associated acute respiratory illnesses (ARI), severe acute respiratory illness (SARI) and deaths among children aged <5 years in Kenya, by County.

**Additional file 3. **Methods for estimating RSV associated severe acute respiratory illness (SARI).

## Data Availability

The data that support the findings of this study are from various sources. Data from national reports are available from the relevant websites, whereas de-identified data/summaries may be availed through the corresponding author upon authorization by KEMRI’s Data Governance Committee.

## Abbreviations

ALRI: Acute lower respiratory infections
ARI: Acute respiratory illness
CDC: US. Centers for Disease Control and Prevention
CFR: Case fatality ratios
CGMR-C: Center for Geographic Medical Research—Coast
CGHR: Center for Global Health Research
CI: Credible intervals
CRH: County Referral Hospital
HDSS: Health and Demographic Surveillance System
HIV: Human immunodeficiency virus
HUS: Health utilization survey
ILI: Influenza like illness
IRB: Institutional review board
KDHS: Kenya Demographic and Health Survey
KEMRI: Kenya Medical Research Institute
LMH: St. Elizabeth Lwak Mission Hospital
LMIC: Low- and middle-income countries
mAbs: Monoclonal antibodies
NH: National hospital
NP: Nasopharyngeal
OP: Oropharyngeal
PBIDS: Population-Based Infectious Disease Surveillance
RSV: Respiratory syncytial virus
SARI: Severe acute respiratory illness
SERU: Scientific ethics review unit
