# Supplementary material for: Estimates of the national burden of respiratory syncytial virus in Kenyan children aged under 5 years, 2010–2018
Source: BMC Med. 2023 Mar 31;21:122. doi: 10.1186/s12916-023-02787-w (PMC10067313; doi:10.1186/s12916-023-02787-w)
Supplement: Supplementary file 1 — Additional file 1. Methods for estimating RSV associated acute respiratory illness (ARI). [file 12916_2023_2787_MOESM1_ESM.docx]

# **Additional file 1 - Methodology:** **The burden of outpatient and non-medically attended RSV-associated mild acute respiratory illness (ARI) among children aged <5 years**

Using data collected from St. Elizabeth Lwak Mission Hospital (LMH) and the Health and Demographic Surveillance System (HDSS) in Asembo- western Kenya, we used the methods and equations outlined in the steps below to estimate the burden of medically and non-medically attended RSV associated acute respiratory illness (ARI);

1. Annual rates of medically attended ARI were calculated for the Asembo residents by dividing the age specific number of ARI cases visiting LMH by the age-specific population of the HDSS residents. This was then adjusted by multiplying the rate by the reciprocal of proportion of those with ARI who reported visiting LMH among those who sought care, from the Household Morbidity Surveillance (HMS) to adjust for health utilization.

$$I_{A}=\frac{{ARI}_{A}}{{Pop}_{A}}*\frac{1}{W}$$

Equation 1

Where:

$I_{A}$ = Rate of medically attended ARI in Asembo

${ARI}_{A}$ = Total number of medically attended ARI cases in Asembo per year

${Pop}_{A}$ = Population of surveillance catchment area

*W* = Proportion of ARI cases visiting LMH among those who sought care (HMS)

1. Annual rate of medically attended ARI for the base region was estimated by multiply the age-specific rate of medically attended ARI in Asembo by the proportion of those seeking care for ARI in the base region divided by the proportion of those seeking care for ARI in the HMS for the region.

$$I_{B}=I_{A}\times\frac{{DHS}_{B}}{{HMS}_{A}}$$

Equation 2

Where:

$I_{B}$ = Rate of medically attended ARI in the base region

$I_{A}$ = Rate of medically attended ARI in Asembo

${DHS}_{B}$ = Proportion of ARI cases seeking care in base region (from DHS)

${HMS}_{A}$ = Proportion of ARI cases seeking care in Asembo (from HMS)- ARI defined as cough and difficulty breathing for >2 days similar to DHS definition

1. We estimated regional adjustment factor for medically attended ARI by summing the net prevalence (regional prevalence less base prevalence) of risk factors multiplied by the relative risk of the risk factor then multiplying by the health utilization in each region divided by the health utilization in the base region to account for health care seeking behavior in different regions.

$${Adj}_{Y}=\left( 1+\sum_{i} \left( P_{i,Y}-P_{i,B} \right)\times\left( {RR}_{i}-1 \right) \right)\times\frac{{DHS}_{Y}}{{DHS}_{B}}$$

Equation 3

Where:

${Adj}_{Y}$ = Adjustment factor for region Y

$P_{i,Y}$ = Prevalence of risk factor *i* in region Y

$P_{i,B}$ = Prevalence of risk factor *i* in base region

${RR}_{i}$ = Relative risk of ARI due to risk factor *i*

${DHS}_{Y}$ = Proportion of ARI cases seeking care in region Y (from DHS)

${DHS}_{B}$ = Proportion of ARI cases seeking care in base region (from DHS)

1. The incidence of medically attended ARI in each region was then estimated by multiplying the base rate by the regional adjustment factors.

$$I_{M,Y}=I_{B} \times{Adj}_{Y}$$

Equation 4

Where:

$I_{M,Y}$ = Incidence of medically attended ARI in region Y

1. The annual region and age specific rates of non-medically attended ARI were calculated by multiplying the region and age specific rates of medically attended ARI by the reciprocal of the proportion of individuals seeking care in each region then subtracting the region and age specific rate of medically attended ARI.

$$I_{NM,Y}=\left( I_{M,Y} \times\frac{1}{{DHS}_{Y}} \right)- I_{M,Y}$$

Equation 5

Where:

$I_{NM,Y}$ = Incidence of non-medically attended ARI in region Y

$I_{M,Y}$ = Incidence of medically attended ARI in region Y

${DHS}_{Y}$ = Proportion of all medically attended ARI in region Y- (from DHS)

1. The annual region and age specific rates of medically attended RSV associated ARI were obtained by multiplying the regional age specific medically attended ARI by the proportion of medically attended ARI positive for RSV. The proportion positive for RSV were only available for the base region, we therefore generated an ARI:SARI RSV positivity factor from the base region, which was applied to the proportion of RSV positive SARI estimated for the other regions to obtain the RSV positivity rate for each region. Due to the limited number of specimens tested by age groups, the percent of ARI positive for RSV was aggregated for the entire period for which results were available (2010-2014) and grouped by the following age categories; <3 months, 3-5 months, 6-8 months, 9-11 months, 12-14 months,15-17 months, 18-20 months,21-23 months, 24-35 months, 36-47 months and 48-59 months.

$${IF}_{M,Y}=I_{M,Y} \times F_{Y}$$

Equation 6

Where:

${IF}_{M,Y}$ = Incidence of medically attended RSV-associated ARI in region Y

$F_{Y}$ = Proportion of ARI due to RSV in region Y

Note:

Out of the 21,021 NPOP specimens collected from patients in Asembo between 2010--2014, 2,425 (12%) were tested for RSV and 257 (11%) were positive for RSV, these represented the base (Nyanza) region (see the distribution by age groups in the table below). The available test results from the 2425 specimens were from a mix of three studies; one had specimen systematically selected for testing while two had specimen randomly selected for testing. There were no other samples tested in the outpatient setting, we therefore applied the ARI:SARI RSV positivity factor from the base region to the severe acute respiratory illness (SARI) testing data from different regions. We used data from Siaya County Referral Hospital (CRH) together with Kakamega CRH for the Western region, data from Kenyatta National hospital (NH) and Nakuru and Nyeri CRHs to estimate the positivity rate for Rift valley, Central and Nairobi regions. For Eastern and North Eastern regions we used the national average percent positive from all the mentioned health facilities including Marsabit CRH and Coast General Teaching and Referral Hospital (GTRH).

| Age group | ARI cases | Tested | | RSV Positive | | |
| --- | --- | --- | --- | --- | --- | --- |
|  |  | n | % | n | % |  |
| <3 months | 548 | 20 | 3.65 | 2 | 10 |  |
| 3-5 months | 1262 | 96 | 7.61 | 13 | 13.54 |  |
| 6-8 months | 1480 | 157 | 10.61 | 16 | 10.19 |  |
| 9-11 months | 1424 | 156 | 10.96 | 25 | 16.03 |  |
| 12-14 months | 1346 | 138 | 10.25 | 21 | 15.22 |  |
| 15-17 months | 1303 | 155 | 11.9 | 19 | 12.26 |  |
| 18-20 months | 1204 | 158 | 13.12 | 15 | 9.49 |  |
| 21-23 months | 1194 | 138 | 11.56 | 15 | 10.87 |  |
| 24-35 months | 4224 | 575 | 13.61 | 54 | 9.39 |  |
| 36-47 months | 3726 | 451 | 12.1 | 50 | 11.09 |  |
| 48-59 months | 3301 | 381 | 11.54 | 27 | 7.09 |  |
| Total | 21012 | 2425 | 11.54 | 257 | 10.60 |  |

1. The region and age specific rates of non-medically attended RSV-associated ARI were estimated by multiplying the region and age specific incidence of non-medically attended ARI by the region and age specific proportion of RSV positive ARI.

$${IF}_{NH,Y}=I_{NH,Y} \times F_{Y}$$

Equation 7

Where:

${IF}_{NM,Y}$ = Incidence of non-medically attended RSV-associated ARI in region Y

$F_{Y}$ = Proportion of ARI due to RSV in region Y

1. We obtained the number of medically and non-medically attended cases of ARI and RSV-associated ARI by multiplying the rates in each region by the county population data obtained from the 2019 census. Since census data were only available in broader age categories, we combined data from Siaya and Kilifi HDSS to build an age structure for every year and applied population proportions to the Census data to obtain fine age bands used in this study.

$${NI}_{M,Y}=I_{M,Y} \times P{op}_{Y}$$

Equation 8

$${NI}_{NM,Y}=I_{NM,Y} \times P{op}_{Y}$$

Equation 9

$${NF}_{M,Y}={IF}_{M,Y} \times P{op}_{Y}$$

Equation 10

$${NF}_{NM,Y}={IF}_{NM,Y} \times P{op}_{Y}$$

Equation 11

Where:

${NI}_{H,Y}$ = Number of medically attended ARI cases in region Y

${NI}_{NH,Y}$ = Number of non-medically attended ARI cases in region Y

${NF}_{H,Y}$ = Number of medically attended RSV-associated ARI cases in region Y

${NF}_{NH,Y}$ = Number of non-medically attended RSV-associated ARI cases in region Y

$P{op}_{Y}$ = Population in region Y
