# Supplementary material for: Estimates of the national burden of respiratory syncytial virus in Kenyan children aged under 5 years, 2010–2018
Source: BMC Med. 2023 Mar 31;21:122. doi: 10.1186/s12916-023-02787-w (PMC10067313; doi:10.1186/s12916-023-02787-w)
Supplement: Supplementary file 2 — Additional file 2. Table S1a. Regional prevalence of risk factors for acute respiratory illness (ARI) and healthcare seeking behaviour for ARI and relative risks for the risk factors, Kenya. Table S1b. Regional prevalence of risk factors for severe acute respiratory illness and healthcare seeking behaviour for acute respiratory illness and relative risks for the risk factors, Kenya. Table S1c. Age-specific in-hospital RSV case fatality rate (CFR) and the out:in hospital RSV deaths multiplier. Table S2. Annual rates of outpatient acute respiratory illness per 1000 children by regions and age in months, 2010-2018. Table S3. Annual rates of outpatient RSV associated acute respiratory illness per 1000 children by regions and age in months, 2010-2018. Table S4. Annual rates of non-medically attended acute respiratory illness per 1000 children by regions and age in months, 2010-2018. Table S5. Annual rates of RSV associated non-medically attended acute respiratory illness per 1000 children by regions and age in months, 2010-2018. Table S6. Annual rates of hospitalized severe acute respiratory illness per 100,000 children by regions and age in months, 2010-2018. Table S7. Annual rates of RSV associated-hospitalized severe acute respiratory illness per 100,000 children by regions and age in months, 2010-2018. Table S8. Annual rates of non-hospitalized severe acute respiratory illness per 100,000 children by regions and age in months, 2010-2018. Table S9. Annual rates of RSV associated non-hospitalized severe acute respiratory illness per 100,000 children by regions and age in months, 2010-2018. Table S10. Annual average number of RSV associated acute respiratory illnesses (ARI), severe acute respiratory illness (SARI) and deaths among children aged <5 years in Kenya, by County. [file 12916_2023_2787_MOESM2_ESM.docx]

**Table S1a: Regional prevalence of risk factors for acute respiratory illness (ARI) and healthcare seeking behaviour for ARI and relative risks for the risk factors, Kenya**

|  | Malnutrition | Household air pollution | Mother's Education (Primary+ None) | Passive smoking | HIV prevalence (<15 yrs) | Prop of ARI seeking care |
| --- | --- | --- | --- | --- | --- | --- |
| Central | 0.054 (0.041,0.067) | 0.767 (0.762,0.773) | 0.453 (0.432,0.474) | 0.164 (0.146,0.185) | 0.007 (0.006,0.007) | 0.689 (0.595,0.792) |
| Coast | 0.137 (0.119,0.155) | 0.685 (0.679,0.691) | 0.685 (0.666,0.703) | 0.149 (0.130,0.170) | 0.009 (0.009,0.010) | 0.654 (0.572,0.740) |
| Eastern | 0.123 (0.108,0.137) | 0.833 (0.828,0.837) | 0.643 (0.627,0.660) | 0.203 (0.185,0.223) | 0.006 (0.006,0.006) | 0.674 (0.606,0.740) |
| Nairobi | 0.042 (0.023,0.062) | 0.456 (0.448,0.465) | 0.339 (0.309,0.371) | 0.077 (0.058,0.102) | 0.012 (0.012,0.012) | 0.622 (0.459,0.806) |
| North Eastern | 0.191 (0.166,0.216) | 0.857 (0.853,0.861) | 0.897 (0.880,0.912) | 0.041 (0.026,0.063) | 0.002 (0.002,0.002) | 0.377 (0.241,0.520) |
| Nyanza | 0.075 (0.065,0.086) | 0.824 (0.819,0.828) | 0.601 (0.585,0.618) | 0.066 (0.055,0.078) | 0.026 (0.026,0.026) | 0.705 (0.644,0.769) |
| Rift Valley | 0.153 (0.142,0.165) | 0.807 (0.802,0.812) | 0.597 (0.584,0.609) | 0.101 (0.091,0.112) | 0.008 (0.008,0.008) | 0.687 (0.642,0.733) |
| Western | 0.091 (0.076,0.107) | 0.864 (0.860,0.868) | 0.632 (0.611,0.653) | 0.102 (0.087,0.120) | 0.009 (0.009,0.009) | 0.565 (0.494,0.639) |
|  |  |  |  |  |  |  |
| Relative Risk | 1.91 | 1.85 | 2.8 | 3.58 | 2.76 | - |

**Table S1b: Regional prevalence of risk factors for severe acute respiratory illness and healthcare seeking behaviour for acute respiratory illness and relative risks for the risk factors, Kenya**

| Region | Malnutrition | Low birth weight | Non-exclusive breastfeeding | Household air pollution | Crowding | HIV prevalence (<15 years) | Health care seeking | Proportion hospitalized |
| --- | --- | --- | --- | --- | --- | --- | --- | --- |
| Central | 0.054 (0.041,0.067) | 0.096 (0.067,0.125) | 0.26 (0.098,0.417) | 0.767 (0.762,0.773) | 0.230 (0.214,0.246) | 0.007 (0.006,0.007) | 0.689 (0.595,0.792) | 0.257 |
| Coast | 0.137 (0.119,0.155) | 0.131 (0.099,0.162) | 0.277 (0.158,0.401) | 0.685 (0.679,0.691) | 0.355 (0.339,0.372) | 0.009 (0.009,0.01) | 0.654 (0.572,0.74) | 0.244 |
| Eastern | 0.123 (0.108,0.137) | 0.087 (0.063,0.112) | 0.36 (0.237,0.487) | 0.833 (0.828,0.837) | 0.379 (0.363,0.394) | 0.006 (0.006,0.006) | 0.674 (0.606,0.74) | 0.236 |
| Nairobi | 0.042 (0.023,0.062) | 0.096 (0.058,0.136) | 0.225 (0.031,0.41) | 0.456 (0.448,0.465) | 0.176 (0.155,0.198) | 0.012 (0.012,0.012) | 0.622 (0.459,0.806) | 0.232 |
| North Eastern | 0.191 (0.166,0.216) | 0.089 (0.046,0.133) | 0.424 (0.263,0.588) | 0.857 (0.853,0.861) | 0.665 (0.64,0.691) | 0.002 (0.002,0.002) | 0.377 (0.241,0.52) | 0.140 |
| Nyanza | 0.075 (0.065,0.086) | 0.037 (0.024,0.05) | 0.357 (0.214,0.494) | 0.824 (0.819,0.828) | 0.467 (0.452,0.483) | 0.026 (0.026,0.026) | 0.705 (0.644,0.769) | 0.231 |
| Rift Valley | 0.153 (0.142,0.165) | 0.067 (0.054,0.081) | 0.3 (0.205,0.395) | 0.807 (0.802,0.812) | 0.396 (0.385,0.407) | 0.008 (0.008,0.008) | 0.687 (0.642,0.733) | 0.228 |
| Western | 0.091 (0.076,0.107) | 0.052 (0.031,0.074) | 0.272 (0.131,0.411) | 0.864 (0.86,0.868) | 0.488 (0.468,0.508) | 0.009 (0.009,0.009) | 0.565 (0.494,0.639) | 0.305 |
|  |  |  |  |  |  |  |  |  |
| Relative Risk | 1.8 | 1.4 | 1.9 | 1.8 | 1.4 | 3.1 | - | - |

**Table S1c: Age specific in-hospital RSV case fatality rate (CFR) and the out:in hospital RSV deaths multiplier**

| Age Categories | In hospital RSV CFR | Multiplier (out:in^a^ of hospital ratio) |
| --- | --- | --- |
|  | % |  |
| 0-5 months | 2.17 | 1.52 |
| 6-11 months | 4.13 | 4.55 |
| 12-23 months | 2.13 | 2.50 |
| 24-59 months | 2.73 | 2.50 |
| 0-59 months | 2.64 | 3.56 |

^a^ This is the inverse of in:out of hospital ratio

**Table S2: Annual rates of outpatient acute respiratory illness per 1,000 children by regions and age in months, 2010-2018**

| Year and Age in months | Central Rate (95% CI) | Coast Rate (95% CI) | Eastern Rate (95% CI) | North Eastern Rate (95% CI) | Nairobi Rate (95% CI) | Nyanza Rate (95% CI) | Rift Valley Rate (95% CI) | Western Rate (95% CI) | Kenya Rate (95% CI) |
| --- | --- | --- | --- | --- | --- | --- | --- | --- | --- |
| 2010-2018 |  |  |  |  |  |  |  |  |  |
| <1 | 356 (304,419) | 485 (417,554) | 563 (499,631) | 331 (229,452) | 71 (42,109) | 409 (365,466) | 439 (399,491) | 437 (379,500) | 391 (357,430) |
| 1 | 638 (539,748) | 865 (758,991) | 1008 (885,1139) | 593 (411,815) | 124 (68,197) | 734 (650,827) | 786 (707,877) | 787 (670,902) | 702 (644,770) |
| 2 | 1108 (933,1277) | 1495 (1309,1710) | 1741 (1557,1938) | 1023 (679,1381) | 214 (121,326) | 1266 (1120,1423) | 1363 (1226,1518) | 1356 (1174,1565) | 1214 (1096,1331) |
| 3 | 1541 (1279,1806) | 2087 (1825,2373) | 2423 (2163,2722) | 1446 (990,1933) | 301 (169,454) | 1772 (1574,2001) | 1896 (1714,2104) | 1896 (1638,2177) | 1689 (1547,1858) |
| 4 | 1792 (1525,2077) | 2433 (2146,2745) | 2857 (2502,3220) | 1681 (1143,2229) | 356 (192,548) | 2069 (1839,2336) | 2210 (1999,2462) | 2221 (1930,2541) | 1982 (1821,2173) |
| 5 | 2024 (1699,2364) | 2757 (2418,3165) | 3199 (2856,3634) | 1873 (1288,2479) | 397 (221,622) | 2322 (2052,2616) | 2504 (2256,2791) | 2488 (2148,2875) | 2229 (2032,2455) |
| 6 | 2233 (1874,2593) | 3025 (2668,3455) | 3523 (3137,3950) | 2079 (1430,2787) | 444 (255,681) | 2544 (2281,2881) | 2734 (2486,3045) | 2717 (2364,3122) | 2445 (2245,2677) |
| 7 | 2259 (1908,2657) | 3077 (2659,3473) | 3583 (3183,4018) | 2078 (1431,2815) | 450 (251,675) | 2598 (2303,2953) | 2787 (2526,3116) | 2766 (2403,3204) | 2489 (2281,2727) |
| 8 | 2147 (1818,2514) | 2931 (2557,3325) | 3389 (3012,3804) | 2011 (1386,2711) | 417 (223,643) | 2469 (2179,2802) | 2655 (2401,2945) | 2641 (2289,3077) | 2361 (2159,2591) |
| 9 | 1748 (1466,2043) | 2353 (2075,2683) | 2747 (2440,3104) | 1622 (1126,2156) | 338 (192,530) | 1991 (1775,2263) | 2139 (1927,2402) | 2134 (1863,2454) | 1910 (1748,2095) |
| 10 | 1910 (1623,2199) | 2576 (2254,2949) | 2991 (2686,3380) | 1758 (1188,2367) | 372 (206,580) | 2174 (1932,2462) | 2342 (2123,2613) | 2338 (1997,2671) | 2087 (1910,2301) |
| 11 | 1683 (1414,1994) | 2278 (2002,2575) | 2657 (2357,2984) | 1558 (1051,2069) | 332 (186,513) | 1923 (1688,2146) | 2068 (1863,2296) | 2066 (1782,2376) | 1840 (1683,2032) |
| <12 | 1600 (1359,1902) | 2177 (1907,2488) | 2519 (2222,2842) | 1502 (1042,1983) | 316 (178,474) | 1829 (1622,2080) | 1976 (1769,2182) | 1971 (1697,2242) | 1758 (1610,1925) |
| 12-14 | 1598 (1351,1905) | 2172 (1893,2458) | 2507 (2250,2825) | 1479 (1028,2043) | 318 (170,493) | 1827 (1617,2055) | 1967 (1765,2203) | 1967 (1706,2253) | 1753 (1602,1935) |
| 15-17 | 1796 (1528,2123) | 2432 (2123,2757) | 2820 (2487,3170) | 1645 (1110,2264) | 346 (199,548) | 2063 (1840,2322) | 2211 (2002,2458) | 2199 (1926,2518) | 1972 (1807,2165) |
| 18-20 | 1805 (1526,2095) | 2434 (2104,2768) | 2824 (2529,3191) | 1662 (1143,2231) | 346 (191,533) | 2057 (1824,2330) | 2212 (2003,2439) | 2218 (1920,2540) | 1975 (1796,2171) |
| 21-23 | 1601 (1353,1887) | 2195 (1918,2510) | 2538 (2255,2853) | 1497 (1020,2077) | 316 (175,488) | 1847 (1643,2067) | 1980 (1789,2212) | 1976 (1703,2274) | 1766 (1616,1944) |
| 12-23 | 1689 (1417,1953) | 2280 (1987,2608) | 2655 (2382,2990) | 1555 (1052,2135) | 332 (174,512) | 1930 (1719,2178) | 2077 (1870,2313) | 2071 (1777,2381) | 1844 (1685,2027) |
| <24 | 1640 (1373,1895) | 2214 (1918,2527) | 2567 (2291,2912) | 1513 (1037,2046) | 319 (185,488) | 1869 (1654,2128) | 2016 (1812,2255) | 2011 (1735,2315) | 1797 (1643,1974) |
| 24-35 | 1724 (1460,2008) | 2333 (2035,2691) | 2718 (2419,3066) | 1591 (1117,2170) | 339 (190,516) | 1963 (1760,2218) | 2124 (1915,2357) | 2112 (1818,2448) | 1882 (1725,2076) |
| 36-47 | 1676 (1411,1972) | 2271 (1976,2586) | 2645 (2344,2947) | 1568 (1048,2094) | 333 (176,516) | 1927 (1717,2167) | 2065 (1866,2306) | 2054 (1810,2374) | 1840 (1684,2028) |
| 48-59 | 1593 (1349,1857) | 2167 (1903,2469) | 2523 (2234,2816) | 1465 (1005,2038) | 316 (183,469) | 1827 (1607,2069) | 1970 (1779,2188) | 1955 (1687,2247) | 1756 (1605,1939) |
| 24-59 | 1655 (1401,1954) | 2242 (1958,2511) | 2605 (2338,2923) | 1552 (1059,2050) | 326 (184,502) | 1900 (1681,2130) | 2043 (1846,2276) | 2018 (1734,2357) | 1815 (1653,2001) |
| <60 | 1568 (1326,1857) | 2144 (1849,2455) | 2483 (2226,2812) | 1455 (962,1982) | 311 (170,479) | 1794 (1585,2031) | 1935 (1745,2137) | 1929 (1677,2220) | 1725 (1577,1899) |
| 2010 |  |  |  |  |  |  |  |  |  |
| <1 | 414 (351,484) | 563 (489,641) | 656 (580,737) | 385 (262,511) | 80 (44,128) | 478 (416,539) | 513 (462,569) | 514 (441,585) | 458 (418,506) |
| 1 | 1266 (1077,1475) | 1729 (1511,1966) | 1997 (1764,2268) | 1165 (800,1567) | 248 (142,391) | 1449 (1293,1631) | 1559 (1402,1739) | 1560 (1341,1787) | 1395 (1275,1528) |
| 2 | 1746 (1457,2058) | 2368 (2058,2699) | 2733 (2404,3077) | 1612 (1108,2127) | 336 (185,515) | 1986 (1769,2229) | 2132 (1933,2388) | 2130 (1846,2429) | 1903 (1737,2088) |
| 3 | 2068 (1751,2431) | 2812 (2464,3193) | 3282 (2898,3715) | 1916 (1302,2627) | 406 (235,624) | 2383 (2104,2674) | 2567 (2310,2845) | 2540 (2198,2932) | 2278 (2079,2484) |
| 4 | 2092 (1760,2451) | 2841 (2488,3266) | 3308 (2936,3712) | 1953 (1259,2620) | 411 (232,634) | 2403 (2141,2694) | 2589 (2338,2875) | 2581 (2214,2941) | 2296 (2115,2525) |
| 5 | 2823 (2377,3277) | 3817 (3308,4389) | 4425 (3912,4986) | 2606 (1729,3519) | 546 (322,815) | 3232 (2871,3628) | 3452 (3098,3860) | 3475 (2976,3997) | 3094 (2835,3407) |
| 6 | 2994 (2515,3466) | 4051 (3535,4604) | 4703 (4187,5302) | 2771 (1845,3781) | 589 (322,919) | 3406 (3043,3849) | 3661 (3335,4106) | 3657 (3096,4218) | 3271 (3007,3624) |
| 7 | 2488 (2097,2928) | 3381 (2965,3848) | 3926 (3492,4465) | 2301 (1581,3115) | 487 (260,753) | 2857 (2528,3222) | 3057 (2777,3431) | 3052 (2660,3496) | 2737 (2490,2988) |
| 8 | 2123 (1773,2507) | 2888 (2554,3308) | 3353 (2985,3771) | 1960 (1318,2703) | 414 (233,660) | 2446 (2138,2735) | 2617 (2382,2906) | 2615 (2245,3000) | 2331 (2146,2567) |
| 9 | 2047 (1725,2420) | 2777 (2416,3194) | 3221 (2865,3619) | 1877 (1235,2574) | 402 (224,601) | 2347 (2085,2622) | 2524 (2281,2789) | 2511 (2175,2927) | 2248 (2060,2471) |
| 10 | 2555 (2153,3048) | 3445 (3012,3948) | 4007 (3575,4493) | 2359 (1614,3214) | 493 (283,771) | 2915 (2593,3312) | 3131 (2833,3486) | 3134 (2719,3568) | 2795 (2551,3062) |
| 11 | 2066 (1752,2444) | 2807 (2453,3193) | 3263 (2901,3638) | 1920 (1339,2609) | 401 (217,627) | 2375 (2100,2685) | 2551 (2301,2838) | 2548 (2221,2920) | 2269 (2068,2494) |
| <12 | 1993 (1707,2331) | 2728 (2409,3129) | 3178 (2811,3576) | 1869 (1282,2463) | 387 (222,591) | 2299 (2052,2605) | 2473 (2240,2781) | 2474 (2133,2827) | 2203 (2020,2433) |
| 12-14 | 2234 (1867,2588) | 3018 (2642,3426) | 3490 (3106,3980) | 2028 (1390,2726) | 434 (239,679) | 2544 (2205,2891) | 2736 (2477,3019) | 2741 (2367,3133) | 2437 (2245,2678) |
| 15-17 | 2435 (2072,2852) | 3284 (2866,3782) | 3822 (3399,4244) | 2257 (1496,3035) | 475 (264,729) | 2777 (2462,3141) | 2988 (2716,3321) | 2989 (2592,3430) | 2659 (2425,2922) |
| 18-20 | 2680 (2280,3148) | 3639 (3172,4156) | 4269 (3779,4799) | 2500 (1758,3350) | 520 (283,812) | 3069 (2704,3507) | 3306 (2973,3659) | 3322 (2880,3781) | 2954 (2684,3277) |
| 21-23 | 2343 (1933,2733) | 3175 (2779,3621) | 3687 (3244,4157) | 2195 (1481,2907) | 459 (246,724) | 2676 (2380,3035) | 2882 (2606,3238) | 2870 (2475,3339) | 2561 (2347,2811) |
| 12-23 | 2396 (1984,2820) | 3261 (2822,3700) | 3777 (3350,4249) | 2211 (1492,2951) | 461 (258,715) | 2744 (2422,3100) | 2944 (2654,3254) | 2955 (2551,3399) | 2625 (2397,2883) |
| <24 | 2190 (1849,2571) | 2973 (2611,3386) | 3467 (3075,3881) | 2032 (1372,2727) | 428 (247,652) | 2509 (2217,2831) | 2695 (2441,3024) | 2687 (2332,3094) | 2404 (2212,2629) |
| 24-35 | 2297 (1961,2714) | 3122 (2721,3561) | 3595 (3181,4036) | 2126 (1441,2820) | 447 (231,684) | 2636 (2297,2956) | 2835 (2537,3162) | 2824 (2439,3185) | 2519 (2308,2784) |
| 36-47 | 2380 (2008,2772) | 3223 (2831,3636) | 3752 (3311,4261) | 2191 (1518,2984) | 466 (249,708) | 2722 (2422,3045) | 2930 (2647,3291) | 2918 (2549,3365) | 2613 (2388,2867) |
| 48-59 | 2286 (1914,2713) | 3104 (2690,3567) | 3617 (3212,4055) | 2146 (1448,2805) | 446 (252,718) | 2616 (2320,2956) | 2820 (2528,3144) | 2801 (2436,3224) | 2512 (2293,2762) |
| 24-59 | 2315 (1955,2707) | 3136 (2737,3570) | 3638 (3222,4084) | 2152 (1497,2949) | 452 (250,702) | 2645 (2341,2996) | 2847 (2556,3158) | 2849 (2470,3261) | 2535 (2307,2776) |
| <60 | 2156 (1811,2519) | 2935 (2543,3336) | 3382 (3013,3801) | 2031 (1394,2776) | 424 (249,659) | 2481 (2199,2824) | 2645 (2400,2958) | 2641 (2307,3052) | 2365 (2165,2611) |
| 2011 |  |  |  |  |  |  |  |  |  |
| <1 | 694 (586,814) | 943 (824,1080) | 1099 (976,1230) | 649 (445,865) | 139 (81,216) | 797 (708,900) | 860 (778,955) | 858 (742,976) | 765 (700,832) |
| 1 | 1106 (934,1290) | 1491 (1306,1694) | 1734 (1538,1947) | 1025 (701,1397) | 217 (123,347) | 1257 (1111,1426) | 1353 (1225,1515) | 1343 (1163,1566) | 1209 (1103,1330) |
| 2 | 991 (845,1159) | 1345 (1173,1534) | 1565 (1382,1782) | 917 (637,1239) | 196 (106,297) | 1135 (1001,1289) | 1219 (1104,1351) | 1222 (1049,1397) | 1091 (999,1190) |
| 3 | 1907 (1612,2224) | 2577 (2255,2917) | 2997 (2672,3386) | 1753 (1233,2325) | 372 (208,564) | 2176 (1937,2459) | 2336 (2094,2616) | 2331 (2009,2689) | 2079 (1899,2295) |
| 4 | 2776 (2341,3274) | 3731 (3266,4252) | 4350 (3861,4867) | 2536 (1729,3451) | 534 (293,857) | 3156 (2807,3569) | 3393 (3062,3808) | 3385 (2935,3932) | 3026 (2774,3312) |
| 5 | 2928 (2457,3413) | 3965 (3479,4545) | 4608 (4063,5195) | 2726 (1841,3746) | 573 (317,850) | 3355 (2962,3784) | 3593 (3239,3962) | 3588 (3142,4181) | 3210 (2931,3511) |
| 6 | 2687 (2249,3137) | 3615 (3163,4119) | 4193 (3728,4734) | 2468 (1688,3292) | 528 (300,837) | 3061 (2700,3444) | 3274 (2976,3639) | 3284 (2835,3762) | 2935 (2696,3237) |
| 7 | 3342 (2806,3918) | 4543 (3965,5239) | 5275 (4673,5922) | 3129 (2129,4084) | 660 (356,1004) | 3844 (3416,4332) | 4128 (3707,4597) | 4126 (3548,4741) | 3673 (3361,4016) |
| 8 | 2487 (2113,2945) | 3381 (2953,3867) | 3908 (3481,4406) | 2302 (1603,3147) | 489 (271,759) | 2842 (2507,3210) | 3052 (2792,3368) | 3046 (2615,3488) | 2724 (2508,2984) |
| 9 | 1834 (1550,2134) | 2490 (2163,2842) | 2889 (2571,3253) | 1713 (1176,2253) | 362 (202,573) | 2095 (1858,2398) | 2257 (2041,2510) | 2250 (1938,2578) | 2017 (1837,2222) |
| 10 | 2262 (1926,2592) | 3046 (2627,3470) | 3537 (3139,3984) | 2074 (1418,2771) | 439 (242,656) | 2567 (2273,2887) | 2764 (2486,3080) | 2755 (2390,3180) | 2473 (2262,2697) |
| 11 | 1923 (1644,2302) | 2631 (2293,2979) | 3058 (2689,3430) | 1801 (1252,2415) | 379 (214,591) | 2212 (1958,2497) | 2390 (2165,2661) | 2378 (2054,2746) | 2128 (1942,2341) |
| <12 | 2003 (1703,2339) | 2728 (2393,3120) | 3173 (2804,3560) | 1888 (1275,2487) | 394 (220,598) | 2306 (2023,2611) | 2473 (2231,2770) | 2474 (2150,2853) | 2207 (2010,2427) |
| 12-14 | 1733 (1473,2040) | 2364 (2052,2701) | 2740 (2453,3076) | 1629 (1085,2145) | 337 (190,516) | 1985 (1774,2237) | 2140 (1938,2393) | 2135 (1848,2469) | 1910 (1747,2088) |
| 15-17 | 2038 (1716,2404) | 2776 (2424,3142) | 3225 (2849,3625) | 1886 (1314,2535) | 401 (209,619) | 2333 (2052,2639) | 2511 (2260,2824) | 2499 (2170,2866) | 2243 (2048,2450) |
| 18-20 | 2052 (1716,2375) | 2782 (2440,3163) | 3222 (2875,3598) | 1905 (1299,2578) | 402 (224,625) | 2343 (2082,2642) | 2521 (2293,2802) | 2507 (2171,2889) | 2243 (2065,2481) |
| 21-23 | 1846 (1544,2142) | 2495 (2200,2840) | 2874 (2544,3260) | 1711 (1168,2246) | 355 (197,562) | 2101 (1867,2376) | 2257 (2051,2504) | 2256 (1965,2589) | 2019 (1834,2224) |
| 12-23 | 1894 (1583,2235) | 2574 (2215,2937) | 2981 (2624,3403) | 1753 (1202,2387) | 373 (214,575) | 2169 (1913,2442) | 2330 (2110,2603) | 2324 (2020,2666) | 2074 (1898,2287) |
| <24 | 1950 (1649,2287) | 2644 (2315,3015) | 3072 (2707,3420) | 1812 (1254,2436) | 379 (219,582) | 2234 (1978,2529) | 2396 (2162,2652) | 2388 (2062,2772) | 2138 (1956,2342) |
| 24-35 | 2069 (1732,2431) | 2807 (2464,3190) | 3264 (2895,3678) | 1947 (1323,2648) | 407 (243,621) | 2368 (2116,2700) | 2562 (2316,2875) | 2549 (2228,2946) | 2280 (2078,2502) |
| 36-47 | 1847 (1579,2167) | 2492 (2161,2845) | 2898 (2585,3252) | 1709 (1142,2290) | 361 (211,564) | 2114 (1862,2388) | 2271 (2060,2536) | 2250 (1932,2584) | 2018 (1851,2225) |
| 48-59 | 1767 (1469,2084) | 2380 (2081,2721) | 2771 (2457,3140) | 1619 (1107,2180) | 339 (191,532) | 2014 (1775,2285) | 2163 (1945,2395) | 2164 (1870,2468) | 1932 (1765,2129) |
| 24-59 | 1884 (1613,2173) | 2553 (2242,2943) | 2972 (2638,3371) | 1755 (1186,2388) | 368 (201,579) | 2165 (1922,2444) | 2325 (2081,2594) | 2320 (2017,2676) | 2073 (1904,2284) |
| <60 | 1833 (1542,2145) | 2482 (2188,2855) | 2892 (2566,3210) | 1703 (1191,2324) | 362 (200,568) | 2094 (1861,2378) | 2256 (2047,2497) | 2248 (1932,2579) | 2011 (1831,2204) |
| 2012 |  |  |  |  |  |  |  |  |  |
| <1 | 520 (438,610) | 703 (618,811) | 815 (724,922) | 476 (325,648) | 100 (54,158) | 593 (521,674) | 636 (575,702) | 638 (550,733) | 568 (520,628) |
| 1 | 728 (618,855) | 988 (866,1129) | 1137 (1018,1277) | 673 (465,917) | 142 (81,219) | 833 (735,941) | 894 (804,994) | 895 (767,1023) | 794 (725,877) |
| 2 | 971 (830,1128) | 1311 (1139,1504) | 1523 (1346,1718) | 890 (621,1204) | 192 (105,293) | 1114 (983,1249) | 1188 (1072,1334) | 1186 (1027,1360) | 1061 (965,1170) |
| 3 | 1957 (1647,2273) | 2632 (2282,3018) | 3073 (2725,3449) | 1799 (1229,2440) | 382 (224,580) | 2240 (1975,2543) | 2399 (2162,2681) | 2391 (2076,2783) | 2140 (1959,2361) |
| 4 | 1908 (1600,2234) | 2622 (2291,2985) | 3023 (2699,3415) | 1784 (1164,2394) | 374 (200,591) | 2202 (1965,2507) | 2364 (2126,2634) | 2355 (2037,2705) | 2108 (1933,2318) |
| 5 | 2192 (1869,2556) | 2947 (2594,3371) | 3417 (3052,3858) | 2013 (1388,2730) | 419 (237,643) | 2489 (2188,2810) | 2687 (2433,2970) | 2659 (2289,3110) | 2385 (2180,2637) |
| 6 | 2120 (1784,2488) | 2882 (2533,3278) | 3349 (2963,3787) | 1957 (1364,2657) | 413 (234,635) | 2429 (2156,2768) | 2612 (2363,2881) | 2615 (2273,2990) | 2337 (2132,2579) |
| 7 | 2084 (1763,2421) | 2826 (2469,3221) | 3280 (2906,3696) | 1909 (1308,2625) | 408 (228,624) | 2386 (2111,2683) | 2554 (2318,2825) | 2548 (2187,2931) | 2281 (2097,2505) |
| 8 | 2676 (2260,3144) | 3624 (3152,4155) | 4225 (3751,4697) | 2530 (1750,3373) | 522 (299,821) | 3066 (2712,3445) | 3304 (2980,3655) | 3296 (2859,3790) | 2936 (2694,3251) |
| 9 | 2016 (1704,2340) | 2724 (2353,3071) | 3172 (2801,3567) | 1856 (1256,2490) | 387 (213,602) | 2299 (2039,2573) | 2479 (2243,2743) | 2471 (2115,2832) | 2201 (2009,2435) |
| 10 | 2144 (1788,2507) | 2893 (2502,3303) | 3372 (2976,3781) | 1962 (1381,2621) | 421 (232,648) | 2452 (2157,2761) | 2625 (2372,2928) | 2619 (2286,3010) | 2342 (2138,2583) |
| 11 | 1441 (1204,1686) | 1965 (1724,2226) | 2283 (2009,2564) | 1343 (919,1788) | 284 (162,440) | 1653 (1472,1862) | 1777 (1594,1965) | 1773 (1537,2042) | 1589 (1443,1756) |
| <12 | 1692 (1413,1971) | 2301 (2020,2644) | 2673 (2373,2981) | 1567 (1068,2084) | 330 (180,526) | 1944 (1730,2192) | 2097 (1889,2337) | 2079 (1816,2376) | 1863 (1705,2051) |
| 12-14 | 1863 (1553,2187) | 2539 (2215,2886) | 2937 (2609,3337) | 1714 (1163,2313) | 363 (212,557) | 2134 (1882,2402) | 2288 (2042,2520) | 2275 (1975,2600) | 2040 (1865,2238) |
| 15-17 | 2064 (1734,2430) | 2792 (2453,3172) | 3254 (2882,3687) | 1938 (1338,2622) | 406 (231,620) | 2356 (2097,2652) | 2542 (2288,2819) | 2532 (2227,2938) | 2259 (2080,2489) |
| 18-20 | 2012 (1680,2360) | 2731 (2411,3111) | 3163 (2813,3581) | 1870 (1272,2454) | 390 (219,610) | 2298 (2050,2610) | 2473 (2240,2764) | 2469 (2137,2820) | 2205 (2020,2430) |
| 21-23 | 1848 (1581,2159) | 2509 (2208,2847) | 2904 (2582,3257) | 1718 (1138,2300) | 360 (205,547) | 2106 (1873,2384) | 2267 (2042,2504) | 2269 (1955,2600) | 2024 (1848,2204) |
| 12-23 | 1935 (1627,2246) | 2604 (2263,2973) | 3028 (2675,3412) | 1788 (1253,2386) | 381 (214,581) | 2203 (1972,2467) | 2370 (2137,2649) | 2371 (2039,2728) | 2122 (1934,2323) |
| <24 | 1808 (1511,2108) | 2447 (2132,2797) | 2833 (2520,3164) | 1666 (1166,2202) | 353 (199,538) | 2060 (1831,2337) | 2213 (2007,2468) | 2210 (1899,2525) | 1983 (1801,2183) |
| 24-35 | 1731 (1437,2021) | 2342 (2043,2666) | 2724 (2410,3061) | 1593 (1119,2139) | 334 (194,524) | 1981 (1759,2251) | 2122 (1926,2357) | 2120 (1817,2447) | 1890 (1727,2080) |
| 36-47 | 1815 (1540,2111) | 2467 (2158,2802) | 2861 (2552,3189) | 1656 (1082,2289) | 357 (194,551) | 2083 (1844,2372) | 2234 (2023,2475) | 2222 (1927,2552) | 1983 (1817,2187) |
| 48-59 | 1592 (1339,1878) | 2153 (1879,2440) | 2497 (2221,2831) | 1451 (1013,1985) | 316 (172,483) | 1819 (1620,2060) | 1960 (1766,2191) | 1943 (1697,2245) | 1749 (1592,1915) |
| 24-59 | 1695 (1433,1982) | 2299 (2000,2637) | 2675 (2370,3006) | 1566 (1103,2110) | 337 (186,520) | 1948 (1714,2180) | 2092 (1886,2317) | 2081 (1813,2387) | 1864 (1703,2047) |
| <60 | 1679 (1416,1956) | 2259 (1972,2551) | 2638 (2321,2947) | 1537 (1021,2079) | 327 (190,517) | 1914 (1704,2166) | 2046 (1857,2279) | 2053 (1772,2335) | 1823 (1666,2011) |
| 2013 |  |  |  |  |  |  |  |  |  |
| <1 | 412 (348,484) | 556 (488,644) | 646 (576,727) | 381 (261,507) | 80 (45,126) | 471 (414,528) | 504 (457,560) | 504 (436,581) | 449 (410,498) |
| 1 | 466 (396,544) | 629 (552,715) | 729 (649,821) | 432 (289,578) | 90 (51,141) | 529 (468,599) | 569 (517,633) | 569 (491,655) | 508 (464,563) |
| 2 | 1199 (1017,1410) | 1628 (1413,1868) | 1889 (1688,2145) | 1114 (746,1476) | 238 (131,364) | 1378 (1211,1552) | 1480 (1339,1647) | 1478 (1266,1712) | 1318 (1204,1451) |
| 3 | 1808 (1521,2128) | 2434 (2108,2771) | 2830 (2508,3202) | 1667 (1132,2235) | 352 (192,544) | 2064 (1835,2324) | 2213 (1985,2478) | 2216 (1906,2561) | 1973 (1813,2167) |
| 4 | 2227 (1888,2648) | 3042 (2636,3471) | 3527 (3131,3979) | 2084 (1409,2828) | 431 (240,671) | 2559 (2265,2887) | 2745 (2490,3056) | 2737 (2373,3155) | 2452 (2244,2691) |
| 5 | 2366 (1973,2775) | 3206 (2809,3637) | 3720 (3306,4177) | 2187 (1531,2904) | 461 (263,721) | 2708 (2385,3048) | 2909 (2622,3211) | 2898 (2525,3342) | 2593 (2368,2872) |
| 6 | 2282 (1921,2637) | 3109 (2725,3539) | 3601 (3193,4043) | 2119 (1461,2861) | 452 (245,701) | 2608 (2317,2950) | 2802 (2546,3120) | 2804 (2427,3214) | 2510 (2295,2742) |
| 7 | 2360 (1993,2781) | 3228 (2792,3664) | 3743 (3313,4218) | 2197 (1416,3001) | 464 (259,707) | 2708 (2416,3069) | 2910 (2633,3222) | 2914 (2511,3316) | 2601 (2364,2857) |
| 8 | 2148 (1816,2509) | 2908 (2534,3319) | 3378 (2994,3806) | 1977 (1346,2706) | 421 (216,658) | 2461 (2172,2779) | 2643 (2374,2938) | 2625 (2281,3049) | 2345 (2149,2590) |
| 9 | 2200 (1873,2554) | 2980 (2597,3385) | 3469 (3088,3858) | 2056 (1408,2766) | 431 (238,649) | 2514 (2236,2852) | 2708 (2448,3036) | 2710 (2338,3126) | 2410 (2211,2659) |
| 10 | 1829 (1550,2141) | 2478 (2177,2852) | 2877 (2545,3239) | 1693 (1177,2321) | 361 (200,568) | 2093 (1846,2371) | 2247 (2033,2476) | 2236 (1937,2573) | 1997 (1832,2206) |
| 11 | 1620 (1379,1892) | 2207 (1926,2500) | 2564 (2293,2899) | 1511 (1038,2026) | 317 (182,488) | 1868 (1646,2100) | 2003 (1798,2216) | 1978 (1738,2292) | 1774 (1633,1957) |
| <12 | 1687 (1418,1996) | 2291 (1998,2622) | 2658 (2348,2968) | 1565 (1081,2118) | 335 (194,520) | 1942 (1723,2188) | 2086 (1893,2316) | 2073 (1802,2376) | 1858 (1700,2024) |
| 12-14 | 1603 (1346,1910) | 2181 (1908,2493) | 2537 (2260,2859) | 1493 (986,1998) | 314 (173,496) | 1841 (1635,2094) | 1983 (1787,2203) | 1980 (1721,2284) | 1759 (1617,1939) |
| 15-17 | 2192 (1846,2587) | 2954 (2594,3403) | 3431 (3056,3868) | 2026 (1429,2666) | 425 (229,660) | 2490 (2213,2817) | 2689 (2417,2976) | 2684 (2320,3076) | 2390 (2181,2626) |
| 18-20 | 1837 (1539,2173) | 2475 (2186,2836) | 2887 (2561,3263) | 1698 (1124,2293) | 352 (199,552) | 2097 (1853,2372) | 2259 (2047,2496) | 2241 (1935,2593) | 2008 (1846,2199) |
| 21-23 | 1763 (1498,2041) | 2389 (2066,2714) | 2765 (2454,3107) | 1639 (1122,2148) | 335 (196,547) | 2018 (1784,2281) | 2167 (1957,2399) | 2157 (1873,2474) | 1927 (1757,2126) |
| 12-23 | 1811 (1541,2101) | 2456 (2153,2822) | 2852 (2522,3219) | 1683 (1159,2216) | 350 (193,534) | 2072 (1842,2322) | 2236 (2009,2468) | 2221 (1932,2562) | 1984 (1823,2190) |
| <24 | 1740 (1472,2050) | 2359 (2072,2699) | 2742 (2424,3078) | 1614 (1099,2167) | 341 (189,548) | 2000 (1772,2236) | 2139 (1934,2393) | 2134 (1855,2457) | 1910 (1739,2108) |
| 24-35 | 1830 (1538,2146) | 2483 (2188,2829) | 2886 (2539,3226) | 1713 (1172,2245) | 359 (197,550) | 2087 (1839,2344) | 2246 (2035,2495) | 2236 (1953,2575) | 2002 (1840,2196) |
| 36-47 | 1655 (1408,1948) | 2253 (1965,2588) | 2606 (2307,2937) | 1525 (1063,2025) | 325 (184,491) | 1895 (1671,2143) | 2039 (1831,2263) | 2033 (1773,2327) | 1818 (1659,1991) |
| 48-59 | 1903 (1593,2216) | 2577 (2277,2914) | 3000 (2657,3392) | 1760 (1230,2389) | 365 (210,564) | 2179 (1915,2471) | 2330 (2111,2582) | 2338 (1997,2699) | 2081 (1889,2295) |
| 24-59 | 1782 (1490,2065) | 2403 (2105,2750) | 2782 (2477,3129) | 1630 (1141,2170) | 352 (194,534) | 2034 (1799,2294) | 2188 (1966,2422) | 2187 (1865,2487) | 1947 (1765,2144) |
| <60 | 1695 (1429,1972) | 2290 (2019,2591) | 2647 (2344,2988) | 1549 (1053,2089) | 328 (185,505) | 1927 (1707,2188) | 2073 (1857,2305) | 2066 (1785,2377) | 1843 (1683,2028) |
| 2014 |  |  |  |  |  |  |  |  |  |
| <1 | 165 (141,193) | 225 (198,256) | 262 (234,297) | 154 (103,207) | 32 (19,50) | 191 (170,217) | 205 (183,228) | 205 (176,236) | 182 (167,201) |
| 1 | 508 (422,596) | 687 (606,780) | 798 (708,899) | 464 (325,634) | 99 (55,152) | 579 (513,651) | 624 (564,693) | 618 (534,711) | 554 (507,610) |
| 2 | 1199 (1021,1417) | 1638 (1436,1868) | 1894 (1685,2144) | 1121 (785,1487) | 234 (134,365) | 1382 (1207,1550) | 1480 (1324,1659) | 1475 (1284,1696) | 1319 (1209,1441) |
| 3 | 1201 (1012,1409) | 1616 (1417,1845) | 1891 (1665,2120) | 1113 (782,1485) | 236 (130,364) | 1370 (1211,1544) | 1473 (1324,1625) | 1467 (1274,1673) | 1316 (1196,1444) |
| 4 | 1561 (1322,1816) | 2110 (1862,2408) | 2451 (2171,2758) | 1437 (956,1938) | 301 (171,468) | 1775 (1574,2005) | 1910 (1724,2131) | 1901 (1640,2167) | 1702 (1559,1863) |
| 5 | 1616 (1353,1857) | 2178 (1897,2478) | 2516 (2211,2835) | 1482 (1022,1987) | 312 (173,486) | 1835 (1645,2098) | 1970 (1787,2197) | 1972 (1703,2265) | 1757 (1614,1936) |
| 6 | 2158 (1821,2535) | 2920 (2548,3332) | 3403 (3024,3881) | 2000 (1322,2667) | 419 (227,674) | 2461 (2177,2780) | 2653 (2411,2949) | 2628 (2285,3050) | 2353 (2159,2590) |
| 7 | 2496 (2106,2899) | 3349 (2934,3790) | 3903 (3485,4448) | 2297 (1525,3113) | 491 (283,739) | 2832 (2495,3181) | 3063 (2761,3380) | 3043 (2659,3471) | 2712 (2481,2980) |
| 8 | 3098 (2637,3647) | 4205 (3664,4780) | 4880 (4318,5514) | 2852 (1910,3806) | 602 (340,940) | 3535 (3124,4005) | 3816 (3449,4260) | 3791 (3287,4377) | 3390 (3099,3734) |
| 9 | 1998 (1689,2320) | 2705 (2361,3065) | 3142 (2778,3530) | 1847 (1228,2492) | 396 (220,597) | 2282 (2018,2581) | 2472 (2226,2751) | 2462 (2117,2787) | 2194 (1992,2407) |
| 10 | 2041 (1720,2383) | 2770 (2415,3183) | 3222 (2864,3621) | 1884 (1228,2554) | 399 (211,614) | 2342 (2070,2649) | 2533 (2267,2823) | 2527 (2176,2911) | 2245 (2057,2489) |
| 11 | 1792 (1528,2072) | 2430 (2107,2759) | 2806 (2497,3175) | 1669 (1149,2271) | 342 (197,540) | 2042 (1807,2309) | 2199 (1985,2451) | 2195 (1871,2495) | 1957 (1799,2156) |
| <12 | 1630 (1382,1901) | 2193 (1928,2496) | 2548 (2270,2861) | 1509 (1021,2022) | 317 (182,498) | 1855 (1645,2105) | 1987 (1786,2220) | 1985 (1711,2293) | 1778 (1621,1953) |
| 12-14 | 1471 (1244,1727) | 2007 (1758,2273) | 2324 (2073,2598) | 1366 (920,1836) | 282 (154,446) | 1691 (1485,1914) | 1823 (1654,2025) | 1819 (1575,2073) | 1621 (1486,1786) |
| 15-17 | 2151 (1855,2521) | 2935 (2573,3329) | 3430 (3018,3838) | 2001 (1411,2703) | 424 (218,661) | 2479 (2201,2800) | 2666 (2387,2963) | 2649 (2294,3045) | 2375 (2159,2619) |
| 18-20 | 1755 (1480,2027) | 2378 (2073,2731) | 2768 (2458,3116) | 1617 (1126,2192) | 344 (194,526) | 2013 (1786,2271) | 2163 (1957,2413) | 2161 (1867,2477) | 1932 (1772,2127) |
| 21-23 | 1877 (1583,2187) | 2546 (2248,2907) | 2945 (2640,3354) | 1738 (1186,2286) | 365 (201,562) | 2138 (1895,2431) | 2305 (2072,2560) | 2306 (1975,2636) | 2054 (1878,2255) |
| 12-23 | 1785 (1502,2074) | 2414 (2117,2733) | 2808 (2484,3150) | 1662 (1132,2225) | 349 (191,548) | 2022 (1809,2291) | 2185 (1982,2455) | 2178 (1897,2523) | 1956 (1789,2148) |
| <24 | 1686 (1427,1971) | 2298 (2017,2590) | 2678 (2362,2998) | 1574 (1050,2114) | 325 (183,497) | 1934 (1707,2186) | 2078 (1883,2294) | 2075 (1777,2353) | 1852 (1701,2033) |
| 24-35 | 1859 (1550,2166) | 2496 (2193,2865) | 2908 (2576,3268) | 1706 (1173,2338) | 365 (207,577) | 2111 (1885,2405) | 2270 (2058,2531) | 2261 (1965,2625) | 2033 (1853,2238) |
| 36-47 | 1899 (1591,2194) | 2569 (2248,2920) | 3000 (2670,3356) | 1741 (1245,2391) | 365 (207,554) | 2173 (1939,2460) | 2329 (2099,2602) | 2323 (1998,2674) | 2081 (1903,2309) |
| 48-59 | 1798 (1507,2098) | 2449 (2135,2793) | 2823 (2512,3173) | 1650 (1128,2260) | 354 (187,536) | 2050 (1837,2312) | 2215 (1997,2462) | 2207 (1914,2506) | 1973 (1801,2182) |
| 24-59 | 1841 (1566,2172) | 2487 (2179,2840) | 2895 (2559,3250) | 1680 (1198,2281) | 357 (201,546) | 2110 (1852,2402) | 2257 (2031,2512) | 2259 (1947,2590) | 2013 (1842,2220) |
| <60 | 1692 (1447,1977) | 2286 (2012,2601) | 2659 (2366,3002) | 1576 (1035,2116) | 325 (180,523) | 1932 (1714,2188) | 2072 (1873,2298) | 2071 (1784,2379) | 1847 (1691,2029) |
| 2015 |  |  |  |  |  |  |  |  |  |
| <1 | 243 (207,285) | 330 (290,379) | 383 (340,433) | 227 (156,309) | 49 (27,73) | 279 (244,313) | 299 (269,332) | 299 (258,343) | 267 (245,295) |
| 1 | 381 (322,451) | 513 (449,582) | 600 (531,681) | 353 (237,472) | 75 (42,114) | 436 (385,490) | 469 (423,521) | 468 (409,535) | 418 (382,460) |
| 2 | 727 (617,858) | 994 (872,1137) | 1151 (1017,1300) | 674 (465,911) | 143 (81,220) | 833 (740,948) | 896 (811,990) | 895 (773,1028) | 801 (734,889) |
| 3 | 1172 (981,1374) | 1590 (1377,1810) | 1843 (1636,2066) | 1079 (735,1476) | 230 (130,355) | 1340 (1187,1515) | 1440 (1291,1594) | 1437 (1247,1656) | 1283 (1175,1414) |
| 4 | 1427 (1219,1683) | 1918 (1694,2197) | 2232 (1985,2543) | 1326 (948,1821) | 271 (154,429) | 1623 (1440,1817) | 1750 (1578,1959) | 1747 (1517,1979) | 1555 (1413,1719) |
| 5 | 982 (836,1150) | 1339 (1179,1532) | 1560 (1382,1754) | 915 (624,1216) | 195 (107,306) | 1127 (1000,1262) | 1215 (1099,1355) | 1208 (1049,1396) | 1079 (986,1202) |
| 6 | 1929 (1624,2272) | 2630 (2297,3000) | 3047 (2696,3445) | 1798 (1264,2440) | 376 (212,591) | 2204 (1972,2508) | 2378 (2163,2631) | 2380 (2064,2757) | 2115 (1929,2332) |
| 7 | 2098 (1783,2441) | 2849 (2482,3246) | 3297 (2918,3741) | 1946 (1307,2612) | 409 (222,633) | 2399 (2120,2699) | 2584 (2335,2849) | 2581 (2231,2966) | 2298 (2109,2534) |
| 8 | 1618 (1385,1909) | 2214 (1936,2538) | 2572 (2285,2872) | 1525 (990,2023) | 321 (184,494) | 1876 (1648,2115) | 2008 (1810,2226) | 1992 (1734,2304) | 1791 (1639,1976) |
| 9 | 843 (720,983) | 1143 (998,1301) | 1327 (1173,1482) | 785 (518,1055) | 166 (94,265) | 963 (848,1075) | 1032 (932,1147) | 1030 (895,1186) | 921 (844,1018) |
| 10 | 1212 (1031,1404) | 1652 (1443,1885) | 1920 (1701,2186) | 1118 (760,1505) | 239 (128,368) | 1389 (1227,1561) | 1494 (1343,1652) | 1492 (1295,1710) | 1336 (1220,1469) |
| 11 | 1103 (929,1287) | 1490 (1306,1692) | 1732 (1547,1954) | 1023 (719,1367) | 213 (125,325) | 1263 (1111,1420) | 1357 (1227,1509) | 1355 (1176,1546) | 1207 (1103,1331) |
| <12 | 1103 (926,1285) | 1502 (1310,1707) | 1734 (1548,1947) | 1024 (714,1381) | 215 (118,333) | 1264 (1122,1426) | 1357 (1227,1502) | 1360 (1161,1569) | 1210 (1109,1318) |
| 12-14 | 1115 (938,1304) | 1510 (1314,1702) | 1754 (1561,1976) | 1038 (704,1382) | 218 (128,333) | 1284 (1125,1442) | 1375 (1227,1534) | 1371 (1181,1563) | 1224 (1126,1345) |
| 15-17 | 924 (783,1079) | 1252 (1100,1422) | 1446 (1285,1640) | 853 (601,1138) | 184 (104,279) | 1056 (934,1193) | 1131 (1018,1254) | 1128 (976,1300) | 1007 (923,1119) |
| 18-20 | 1021 (868,1198) | 1393 (1202,1595) | 1611 (1430,1809) | 945 (640,1259) | 196 (118,304) | 1171 (1031,1308) | 1253 (1131,1382) | 1256 (1097,1443) | 1121 (1032,1232) |
| 21-23 | 1024 (846,1196) | 1381 (1204,1562) | 1609 (1424,1814) | 945 (648,1293) | 200 (116,315) | 1163 (1018,1319) | 1256 (1130,1401) | 1254 (1086,1446) | 1114 (1027,1232) |
| 12-23 | 1011 (859,1193) | 1366 (1186,1575) | 1586 (1416,1789) | 931 (664,1270) | 197 (113,302) | 1151 (1026,1310) | 1242 (1127,1375) | 1243 (1070,1419) | 1108 (1014,1221) |
| <24 | 1044 (886,1231) | 1429 (1242,1629) | 1662 (1473,1882) | 980 (651,1298) | 204 (114,318) | 1208 (1064,1362) | 1292 (1171,1443) | 1292 (1118,1478) | 1154 (1059,1280) |
| 24-35 | 1232 (1056,1451) | 1683 (1472,1913) | 1953 (1738,2226) | 1144 (801,1518) | 246 (130,377) | 1423 (1245,1610) | 1526 (1380,1702) | 1526 (1331,1741) | 1364 (1246,1499) |
| 36-47 | 1139 (967,1338) | 1550 (1369,1759) | 1799 (1606,2022) | 1064 (727,1401) | 223 (117,347) | 1308 (1158,1475) | 1403 (1267,1566) | 1403 (1212,1604) | 1250 (1148,1388) |
| 48-59 | 1071 (910,1266) | 1454 (1276,1663) | 1688 (1505,1909) | 995 (688,1335) | 209 (113,324) | 1234 (1088,1388) | 1322 (1188,1461) | 1321 (1140,1498) | 1180 (1083,1292) |
| 24-59 | 1149 (973,1356) | 1558 (1354,1783) | 1810 (1606,2037) | 1070 (706,1401) | 220 (118,349) | 1320 (1164,1492) | 1408 (1275,1565) | 1409 (1233,1616) | 1264 (1157,1381) |
| <60 | 1053 (877,1214) | 1423 (1248,1626) | 1658 (1473,1872) | 983 (679,1311) | 208 (113,318) | 1207 (1064,1358) | 1297 (1158,1437) | 1289 (1108,1489) | 1153 (1057,1266) |
| 2016 |  |  |  |  |  |  |  |  |  |
| <1 | 136 (114,159) | 185 (162,211) | 214 (189,241) | 126 (86,169) | 26 (15,43) | 156 (138,175) | 168 (151,185) | 168 (146,191) | 149 (136,164) |
| 1 | 269 (227,314) | 364 (323,411) | 421 (372,475) | 252 (171,334) | 52 (29,80) | 307 (269,344) | 330 (300,367) | 329 (284,377) | 293 (268,321) |
| 2 | 1100 (937,1297) | 1488 (1317,1709) | 1727 (1537,1944) | 1017 (699,1375) | 216 (126,333) | 1256 (1119,1402) | 1352 (1218,1509) | 1347 (1168,1550) | 1207 (1102,1334) |
| 3 | 863 (730,1001) | 1174 (1028,1342) | 1365 (1215,1539) | 809 (551,1102) | 168 (94,254) | 989 (879,1127) | 1063 (959,1180) | 1061 (918,1230) | 952 (871,1055) |
| 4 | 1171 (993,1394) | 1591 (1399,1825) | 1850 (1629,2075) | 1082 (751,1456) | 230 (129,354) | 1349 (1193,1515) | 1448 (1309,1610) | 1454 (1266,1673) | 1287 (1183,1425) |
| 5 | 1409 (1185,1655) | 1916 (1686,2182) | 2223 (1970,2496) | 1294 (894,1744) | 275 (154,438) | 1619 (1412,1816) | 1736 (1575,1929) | 1747 (1500,1993) | 1547 (1425,1708) |
| 6 | 2029 (1721,2354) | 2758 (2395,3135) | 3195 (2850,3595) | 1881 (1349,2506) | 392 (217,609) | 2326 (2064,2636) | 2505 (2257,2769) | 2497 (2154,2875) | 2223 (2033,2471) |
| 7 | 1751 (1479,2043) | 2359 (2054,2682) | 2730 (2422,3067) | 1621 (1110,2206) | 345 (193,528) | 1997 (1774,2246) | 2145 (1931,2370) | 2140 (1861,2446) | 1911 (1750,2114) |
| 8 | 1246 (1054,1458) | 1684 (1485,1908) | 1967 (1748,2205) | 1150 (791,1521) | 243 (139,380) | 1423 (1265,1605) | 1530 (1388,1688) | 1532 (1314,1771) | 1366 (1248,1509) |
| 9 | 1548 (1311,1799) | 2108 (1849,2424) | 2442 (2181,2765) | 1430 (982,1935) | 301 (170,461) | 1787 (1572,2008) | 1908 (1724,2131) | 1900 (1648,2190) | 1706 (1565,1889) |
| 10 | 1848 (1568,2160) | 2510 (2197,2854) | 2917 (2579,3252) | 1704 (1159,2324) | 359 (193,565) | 2114 (1876,2370) | 2273 (2050,2526) | 2279 (1933,2612) | 2030 (1853,2243) |
| 11 | 1677 (1421,1949) | 2287 (2025,2612) | 2646 (2341,2985) | 1555 (1034,2106) | 329 (175,517) | 1927 (1712,2179) | 2076 (1872,2318) | 2063 (1780,2355) | 1856 (1684,2047) |
| <12 | 1195 (995,1416) | 1621 (1418,1856) | 1894 (1675,2121) | 1112 (770,1474) | 237 (129,361) | 1375 (1208,1541) | 1475 (1329,1648) | 1473 (1251,1707) | 1314 (1201,1450) |
| 12-14 | 1381 (1172,1609) | 1864 (1632,2117) | 2158 (1908,2435) | 1276 (837,1709) | 269 (153,416) | 1571 (1410,1783) | 1690 (1524,1869) | 1678 (1470,1926) | 1508 (1371,1658) |
| 15-17 | 1319 (1122,1545) | 1784 (1557,2045) | 2072 (1836,2345) | 1217 (827,1644) | 256 (141,393) | 1509 (1332,1705) | 1621 (1469,1809) | 1617 (1394,1891) | 1441 (1322,1584) |
| 18-20 | 1594 (1339,1857) | 2147 (1890,2462) | 2498 (2224,2826) | 1465 (1003,1979) | 313 (163,467) | 1815 (1612,2048) | 1947 (1772,2172) | 1951 (1710,2252) | 1740 (1595,1926) |
| 21-23 | 1069 (903,1266) | 1458 (1273,1665) | 1691 (1489,1891) | 996 (674,1340) | 211 (122,335) | 1230 (1094,1394) | 1317 (1204,1480) | 1318 (1125,1509) | 1175 (1071,1289) |
| 12-23 | 1326 (1102,1530) | 1799 (1570,2043) | 2090 (1859,2336) | 1234 (832,1642) | 257 (142,401) | 1513 (1346,1720) | 1627 (1473,1813) | 1636 (1408,1867) | 1449 (1316,1579) |
| <24 | 1255 (1052,1477) | 1702 (1489,1955) | 1973 (1749,2211) | 1173 (765,1583) | 248 (137,377) | 1428 (1276,1610) | 1548 (1389,1730) | 1538 (1339,1780) | 1372 (1261,1516) |
| 24-35 | 1272 (1072,1483) | 1716 (1502,1957) | 1996 (1758,2252) | 1182 (783,1594) | 248 (141,386) | 1452 (1295,1640) | 1564 (1413,1765) | 1561 (1357,1796) | 1396 (1272,1530) |
| 36-47 | 1497 (1254,1732) | 2028 (1761,2307) | 2358 (2109,2642) | 1382 (930,1844) | 289 (168,460) | 1716 (1527,1922) | 1837 (1660,2039) | 1834 (1599,2108) | 1636 (1495,1799) |
| 48-59 | 1258 (1054,1478) | 1710 (1499,1933) | 1987 (1760,2237) | 1157 (807,1561) | 246 (137,373) | 1441 (1274,1616) | 1557 (1402,1717) | 1541 (1334,1765) | 1380 (1257,1520) |
| 24-59 | 1321 (1114,1545) | 1798 (1579,2068) | 2088 (1856,2342) | 1225 (822,1646) | 259 (143,401) | 1519 (1345,1691) | 1630 (1473,1809) | 1625 (1417,1861) | 1455 (1333,1595) |
| <60 | 1232 (1040,1458) | 1679 (1460,1902) | 1944 (1739,2182) | 1142 (790,1574) | 240 (128,363) | 1418 (1244,1593) | 1521 (1377,1695) | 1516 (1310,1750) | 1359 (1224,1477) |
| 2017 |  |  |  |  |  |  |  |  |  |
| <1 | 338 (282,401) | 458 (398,521) | 534 (477,602) | 313 (214,424) | 65 (38,105) | 385 (340,436) | 416 (374,462) | 415 (360,473) | 369 (338,405) |
| 1 | 476 (402,549) | 645 (563,738) | 751 (661,843) | 442 (298,589) | 92 (53,141) | 544 (484,615) | 586 (530,651) | 587 (506,672) | 520 (476,571) |
| 2 | 972 (815,1134) | 1318 (1141,1508) | 1529 (1351,1727) | 905 (610,1201) | 191 (106,295) | 1111 (995,1269) | 1196 (1068,1334) | 1189 (1022,1361) | 1066 (975,1177) |
| 3 | 1560 (1299,1825) | 2102 (1841,2392) | 2456 (2175,2751) | 1427 (983,1904) | 310 (177,468) | 1779 (1562,1998) | 1907 (1721,2120) | 1907 (1638,2198) | 1703 (1549,1885) |
| 4 | 1938 (1641,2279) | 2615 (2277,3019) | 3037 (2693,3442) | 1791 (1230,2410) | 379 (220,580) | 2214 (1950,2507) | 2385 (2145,2645) | 2367 (2054,2711) | 2118 (1939,2346) |
| 5 | 1919 (1620,2258) | 2610 (2267,2983) | 3032 (2698,3400) | 1775 (1193,2401) | 373 (212,578) | 2192 (1956,2477) | 2356 (2122,2630) | 2351 (2034,2731) | 2107 (1937,2325) |
| 6 | 1681 (1419,1968) | 2268 (1985,2584) | 2628 (2362,2965) | 1543 (1073,2073) | 326 (174,496) | 1916 (1703,2161) | 2056 (1865,2280) | 2051 (1760,2365) | 1832 (1670,2012) |
| 7 | 1649 (1394,1933) | 2235 (1935,2573) | 2578 (2295,2910) | 1523 (988,2069) | 316 (179,483) | 1874 (1669,2138) | 2028 (1812,2255) | 2017 (1739,2331) | 1797 (1641,1980) |
| 8 | 2167 (1837,2554) | 2926 (2566,3303) | 3402 (3037,3873) | 2004 (1355,2681) | 427 (230,661) | 2476 (2189,2811) | 2660 (2414,2955) | 2664 (2301,3086) | 2371 (2166,2612) |
| 9 | 1432 (1199,1671) | 1942 (1690,2226) | 2244 (1993,2513) | 1314 (898,1735) | 278 (164,436) | 1638 (1441,1858) | 1764 (1582,1958) | 1746 (1520,2028) | 1566 (1435,1733) |
| 10 | 1314 (1106,1520) | 1777 (1560,2009) | 2054 (1832,2322) | 1218 (842,1630) | 254 (144,393) | 1491 (1323,1684) | 1607 (1441,1789) | 1600 (1397,1829) | 1434 (1294,1566) |
| 11 | 1938 (1624,2226) | 2615 (2292,2973) | 3040 (2721,3411) | 1783 (1197,2367) | 378 (212,577) | 2215 (1961,2501) | 2385 (2138,2653) | 2380 (2072,2721) | 2123 (1947,2327) |
| <12 | 1427 (1197,1662) | 1931 (1680,2210) | 2248 (1980,2535) | 1318 (909,1748) | 275 (154,422) | 1623 (1439,1833) | 1756 (1581,1938) | 1756 (1514,2021) | 1560 (1433,1725) |
| 12-14 | 1357 (1136,1587) | 1842 (1590,2091) | 2135 (1915,2404) | 1251 (856,1659) | 269 (149,412) | 1559 (1380,1762) | 1673 (1519,1858) | 1658 (1448,1928) | 1489 (1356,1628) |
| 15-17 | 1372 (1172,1604) | 1861 (1634,2149) | 2174 (1917,2465) | 1279 (866,1721) | 264 (145,415) | 1576 (1391,1772) | 1691 (1542,1882) | 1692 (1471,1966) | 1519 (1384,1672) |
| 18-20 | 1656 (1388,1950) | 2267 (1968,2590) | 2626 (2327,2935) | 1547 (1021,2035) | 329 (182,502) | 1903 (1706,2138) | 2041 (1851,2277) | 2047 (1770,2355) | 1834 (1678,2027) |
| 21-23 | 1268 (1063,1477) | 1718 (1510,1956) | 1995 (1782,2236) | 1167 (783,1572) | 251 (136,381) | 1452 (1285,1662) | 1559 (1409,1737) | 1566 (1352,1806) | 1390 (1270,1537) |
| 12-23 | 1417 (1193,1663) | 1912 (1676,2161) | 2220 (1970,2497) | 1308 (876,1783) | 274 (148,438) | 1615 (1427,1827) | 1739 (1569,1930) | 1727 (1503,1991) | 1544 (1410,1711) |
| <24 | 1409 (1209,1676) | 1919 (1666,2192) | 2222 (1969,2510) | 1300 (881,1748) | 278 (152,428) | 1614 (1434,1826) | 1737 (1559,1924) | 1736 (1495,1995) | 1547 (1411,1707) |
| 24-35 | 1528 (1291,1793) | 2081 (1809,2386) | 2410 (2136,2711) | 1411 (969,1908) | 294 (167,460) | 1766 (1555,1992) | 1880 (1691,2098) | 1877 (1641,2137) | 1681 (1534,1836) |
| 36-47 | 1342 (1135,1563) | 1807 (1577,2069) | 2098 (1870,2359) | 1250 (841,1631) | 259 (142,398) | 1533 (1350,1712) | 1641 (1473,1816) | 1637 (1409,1875) | 1458 (1332,1607) |
| 48-59 | 1281 (1082,1504) | 1730 (1519,1985) | 2013 (1798,2272) | 1184 (785,1573) | 249 (134,374) | 1467 (1289,1645) | 1571 (1421,1758) | 1563 (1365,1809) | 1403 (1274,1548) |
| 24-59 | 1379 (1179,1602) | 1866 (1619,2108) | 2169 (1909,2456) | 1291 (873,1735) | 272 (148,412) | 1571 (1390,1805) | 1691 (1536,1870) | 1686 (1465,1936) | 1505 (1371,1662) |
| <60 | 1330 (1114,1571) | 1799 (1585,2039) | 2101 (1865,2363) | 1257 (848,1664) | 263 (147,407) | 1527 (1337,1736) | 1640 (1486,1821) | 1639 (1415,1888) | 1464 (1341,1607) |
| 2018 |  |  |  |  |  |  |  |  |  |
| <1 | 328 (278,380) | 449 (392,504) | 519 (462,580) | 305 (202,412) | 64 (37,100) | 378 (333,426) | 404 (363,454) | 404 (346,463) | 361 (330,398) |
| 1 | 560 (475,658) | 759 (662,871) | 882 (788,986) | 517 (344,696) | 111 (65,168) | 642 (564,719) | 691 (621,767) | 687 (598,787) | 615 (563,677) |
| 2 | 998 (841,1172) | 1360 (1183,1556) | 1577 (1397,1786) | 923 (610,1286) | 198 (106,303) | 1146 (1009,1297) | 1234 (1114,1365) | 1229 (1056,1412) | 1099 (1011,1206) |
| 3 | 1425 (1204,1670) | 1923 (1668,2193) | 2227 (1973,2503) | 1304 (914,1792) | 272 (147,428) | 1617 (1437,1827) | 1742 (1574,1933) | 1734 (1489,1995) | 1546 (1417,1702) |
| 4 | 1403 (1192,1630) | 1913 (1683,2169) | 2208 (1970,2488) | 1297 (901,1794) | 278 (158,428) | 1613 (1430,1809) | 1732 (1567,1924) | 1720 (1490,1986) | 1544 (1409,1695) |
| 5 | 2102 (1810,2450) | 2860 (2507,3228) | 3328 (2918,3704) | 1953 (1369,2610) | 411 (226,618) | 2416 (2140,2746) | 2594 (2323,2862) | 2591 (2246,2983) | 2308 (2110,2540) |
| 6 | 2121 (1773,2464) | 2856 (2498,3300) | 3307 (2930,3710) | 1945 (1331,2631) | 414 (230,650) | 2410 (2143,2727) | 2592 (2343,2887) | 2590 (2260,2962) | 2310 (2106,2551) |
| 7 | 2475 (2064,2888) | 3369 (2947,3875) | 3907 (3451,4416) | 2306 (1620,3157) | 484 (269,759) | 2831 (2522,3209) | 3044 (2749,3403) | 3044 (2637,3502) | 2720 (2474,3002) |
| 8 | 2269 (1910,2668) | 3082 (2704,3496) | 3586 (3179,4004) | 2128 (1486,2894) | 442 (255,697) | 2605 (2318,2940) | 2791 (2519,3141) | 2801 (2415,3192) | 2483 (2275,2741) |
| 9 | 1791 (1495,2104) | 2421 (2104,2771) | 2804 (2466,3169) | 1656 (1125,2213) | 346 (196,532) | 2042 (1804,2303) | 2191 (1995,2427) | 2183 (1880,2532) | 1960 (1782,2147) |
| 10 | 1994 (1675,2355) | 2726 (2381,3101) | 3156 (2809,3565) | 1865 (1247,2490) | 391 (223,609) | 2292 (2039,2580) | 2461 (2227,2727) | 2438 (2123,2801) | 2197 (2012,2419) |
| 11 | 1631 (1386,1901) | 2194 (1912,2501) | 2565 (2266,2904) | 1498 (996,1993) | 318 (183,509) | 1863 (1648,2089) | 1997 (1807,2235) | 1995 (1723,2285) | 1781 (1632,1956) |
| <12 | 1581 (1332,1843) | 2137 (1873,2441) | 2490 (2211,2802) | 1456 (1019,1951) | 307 (171,472) | 1812 (1598,2037) | 1950 (1757,2175) | 1939 (1686,2229) | 1735 (1584,1910) |
| 12-14 | 1587 (1320,1860) | 2142 (1857,2439) | 2496 (2202,2831) | 1471 (952,1974) | 304 (171,480) | 1812 (1614,2038) | 1944 (1764,2162) | 1946 (1687,2229) | 1734 (1585,1905) |
| 15-17 | 1758 (1493,2052) | 2373 (2090,2706) | 2755 (2453,3157) | 1632 (1100,2140) | 342 (192,538) | 2022 (1798,2281) | 2162 (1955,2406) | 2162 (1867,2475) | 1929 (1762,2113) |
| 18-20 | 1432 (1202,1688) | 1930 (1691,2202) | 2255 (2006,2547) | 1315 (929,1779) | 277 (161,424) | 1642 (1461,1849) | 1757 (1599,1959) | 1752 (1507,2022) | 1571 (1440,1720) |
| 21-23 | 1417 (1182,1659) | 1911 (1665,2191) | 2221 (1958,2491) | 1301 (877,1724) | 278 (152,437) | 1615 (1435,1793) | 1734 (1570,1920) | 1723 (1488,1997) | 1545 (1413,1702) |
| 12-23 | 1523 (1277,1793) | 2056 (1799,2346) | 2395 (2137,2704) | 1407 (978,1899) | 302 (176,455) | 1737 (1539,1972) | 1872 (1687,2076) | 1866 (1611,2151) | 1661 (1527,1828) |
| <24 | 1541 (1302,1808) | 2095 (1817,2428) | 2430 (2148,2734) | 1423 (975,1926) | 301 (162,458) | 1770 (1576,1987) | 1901 (1708,2125) | 1905 (1639,2199) | 1685 (1541,1862) |
| 24-35 | 1590 (1337,1854) | 2145 (1898,2447) | 2487 (2224,2801) | 1461 (997,1983) | 312 (172,476) | 1817 (1602,2022) | 1950 (1755,2156) | 1955 (1689,2233) | 1735 (1593,1904) |
| 36-47 | 1501 (1252,1754) | 2022 (1762,2309) | 2342 (2074,2676) | 1397 (965,1848) | 293 (159,454) | 1705 (1515,1908) | 1828 (1656,2045) | 1834 (1584,2115) | 1642 (1493,1799) |
| 48-59 | 1406 (1188,1624) | 1904 (1656,2166) | 2212 (1975,2507) | 1292 (902,1757) | 279 (156,417) | 1610 (1436,1809) | 1728 (1555,1914) | 1717 (1481,1977) | 1540 (1410,1690) |
| 24-59 | 1487 (1281,1741) | 2023 (1779,2311) | 2349 (2092,2666) | 1374 (935,1850) | 289 (168,451) | 1706 (1509,1913) | 1836 (1658,2025) | 1830 (1579,2112) | 1637 (1495,1804) |
| <60 | 1440 (1209,1683) | 1961 (1707,2239) | 2281 (2008,2569) | 1325 (901,1817) | 283 (165,429) | 1644 (1463,1867) | 1770 (1601,1978) | 1773 (1536,2026) | 1582 (1452,1741) |

**Table S3: Annual rates of outpatient RSV associated acute respiratory illness per 1,000 children by regions and age in months, 2010-2018**

| Year and Age in months | Central Rate (95% CI) | Coast Rate (95% CI) | Eastern Rate (95% CI) | North Eastern Rate (95% CI) | Nairobi Rate (95% CI) | Nyanza Rate (95% CI) | Rift Valley Rate (95% CI) | Western Rate (95% CI) | Kenya Rate (95% CI) |
| --- | --- | --- | --- | --- | --- | --- | --- | --- | --- |
| 2010-2018 |  |  |  |  |  |  |  |  |  |
| <1 | 24 (11,42) | 47 (31,63) | 58 (41,80) | 34 (20,51) | 5 (2,10) | 40 (0,102) | 29 (12,48) | 34 (19,52) | 38 (27,51) |
| 1 | 47 (19,80) | 120 (90,155) | 115 (87,147) | 67 (43,101) | 9 (4,18) | 71 (0,183) | 58 (29,100) | 69 (39,104) | 93 (74,116) |
| 2 | 108 (57,172) | 244 (181,318) | 265 (209,328) | 155 (98,229) | 21 (9,40) | 126 (0,324) | 135 (73,211) | 158 (100,233) | 160 (125,199) |
| 3 | 182 (131,248) | 528 (411,662) | 456 (373,546) | 268 (178,379) | 35 (19,58) | 235 (129,374) | 225 (168,295) | 259 (198,335) | 350 (289,415) |
| 4 | 242 (172,318) | 654 (526,812) | 601 (492,728) | 351 (234,488) | 48 (24,78) | 276 (150,443) | 297 (222,383) | 346 (266,440) | 410 (343,489) |
| 5 | 242 (176,328) | 634 (498,794) | 609 (500,740) | 356 (238,482) | 47 (25,78) | 314 (167,489) | 301 (221,398) | 348 (264,451) | 390 (322,463) |
| 6 | 184 (122,256) | 435 (307,565) | 390 (301,481) | 227 (146,333) | 36 (19,61) | 256 (134,389) | 226 (153,306) | 297 (216,395) | 290 (229,360) |
| 7 | 165 (107,234) | 357 (242,495) | 347 (261,439) | 203 (129,293) | 32 (16,55) | 262 (153,394) | 203 (139,282) | 267 (195,354) | 217 (164,278) |
| 8 | 165 (109,234) | 281 (181,394) | 347 (254,443) | 203 (125,291) | 32 (15,54) | 248 (133,380) | 201 (137,282) | 268 (195,355) | 243 (182,315) |
| 9 | 258 (181,346) | 439 (311,586) | 461 (362,571) | 270 (174,384) | 49 (26,85) | 316 (204,442) | 319 (230,414) | 361 (275,456) | 329 (267,403) |
| 10 | 261 (185,361) | 448 (322,582) | 466 (373,578) | 273 (179,397) | 50 (27,86) | 349 (233,489) | 319 (236,419) | 371 (281,473) | 326 (265,405) |
| 11 | 240 (167,327) | 339 (234,473) | 428 (329,538) | 250 (160,354) | 47 (25,77) | 307 (196,448) | 294 (210,388) | 338 (255,434) | 286 (221,355) |
| <12 | 176 (141,222) | 465 (401,539) | 431 (376,490) | 258 (179,340) | 35 (19,55) | 239 (174,309) | 217 (179,260) | 261 (216,316) | 302 (272,336) |
| 12-14 | 144 (93,210) | 273 (205,349) | 314 (256,377) | 185 (121,262) | 28 (13,49) | 276 (166,397) | 178 (116,253) | 291 (207,386) | 219 (183,262) |
| 15-17 | 136 (60,235) | 226 (159,305) | 265 (209,331) | 155 (99,224) | 26 (9,53) | 254 (151,382) | 169 (75,284) | 252 (170,349) | 185 (145,228) |
| 18-20 | 172 (91,284) | 170 (108,245) | 232 (174,296) | 136 (86,195) | 32 (14,63) | 192 (102,303) | 215 (101,345) | 209 (129,296) | 162 (122,205) |
| 21-23 | 285 (164,454) | 153 (87,223) | 241 (179,314) | 141 (91,213) | 56 (25,106) | 199 (108,310) | 363 (203,539) | 197 (119,291) | 168 (126,215) |
| 12-23 | 163 (120,209) | 208 (171,254) | 264 (227,307) | 155 (107,213) | 32 (17,54) | 228 (172,293) | 202 (151,250) | 237 (188,294) | 183 (160,210) |
| <24 | 171 (136,211) | 393 (337,460) | 379 (333,433) | 223 (155,305) | 33 (18,53) | 231 (186,283) | 212 (179,250) | 251 (209,295) | 265 (241,294) |
| 24-35 | 158 (101,229) | 140 (102,182) | 203 (162,248) | 118 (81,167) | 32 (16,54) | 183 (136,235) | 198 (131,280) | 191 (140,251) | 141 (114,168) |
| 36-47 | 155 (82,247) | 107 (67,155) | 188 (146,238) | 113 (70,162) | 30 (13,56) | 213 (152,280) | 193 (112,295) | 206 (140,279) | 132 (102,165) |
| 48-59 | 46 (11,100) | 28 (6,62) | 86 (55,124) | 50 (29,79) | 9 (2,22) | 128 (85,184) | 55 (13,116) | 121 (72,182) | 60 (39,86) |
| 24-59 | 132 (93,181) | 109 (84,137) | 172 (146,202) | 102 (69,138) | 26 (14,42) | 177 (142,215) | 161 (117,213) | 172 (135,218) | 120 (102,140) |
| <60 | 146 (117,178) | 298 (254,345) | 297 (262,336) | 174 (115,236) | 29 (16,45) | 190 (160,223) | 178 (150,211) | 199 (169,236) | 206 (186,229) |
| 2010 |  |  |  |  |  |  |  |  |  |
| <1 | 28 (12,47) | 54 (36,75) | 68 (46,91) | 40 (24,60) | 5 (2,11) | 47 (0,124) | 34 (15,58) | 41 (23,63) | 44 (31,60) |
| 1 | 92 (43,157) | 237 (185,307) | 227 (174,290) | 131 (87,193) | 18 (7,36) | 141 (0,374) | 115 (53,190) | 135 (77,204) | 184 (146,226) |
| 2 | 171 (88,278) | 385 (282,489) | 417 (326,518) | 244 (163,345) | 32 (15,62) | 196 (0,543) | 211 (109,329) | 249 (159,361) | 251 (193,310) |
| 3 | 249 (172,331) | 716 (568,875) | 614 (503,753) | 357 (236,511) | 48 (25,81) | 317 (168,500) | 303 (226,402) | 349 (264,448) | 473 (390,561) |
| 4 | 281 (201,377) | 761 (599,938) | 692 (574,846) | 410 (269,562) | 55 (28,91) | 321 (170,494) | 346 (249,448) | 400 (309,503) | 476 (399,567) |
| 5 | 338 (241,446) | 877 (685,1097) | 836 (677,1020) | 496 (327,704) | 64 (35,106) | 434 (221,677) | 418 (300,543) | 483 (360,620) | 539 (447,651) |
| 6 | 249 (163,348) | 578 (433,772) | 514 (404,648) | 302 (193,441) | 48 (25,85) | 337 (187,526) | 304 (205,418) | 399 (294,517) | 393 (309,483) |
| 7 | 178 (118,261) | 393 (268,551) | 378 (286,490) | 221 (145,317) | 35 (16,61) | 289 (162,444) | 221 (146,309) | 298 (206,394) | 238 (180,304) |
| 8 | 162 (102,232) | 279 (170,395) | 342 (254,448) | 196 (124,299) | 32 (16,54) | 243 (138,373) | 199 (131,271) | 266 (197,344) | 242 (178,312) |
| 9 | 301 (213,409) | 509 (361,675) | 535 (426,666) | 313 (201,452) | 59 (32,97) | 375 (239,520) | 371 (268,492) | 426 (325,542) | 389 (311,473) |
| 10 | 350 (242,492) | 592 (429,781) | 617 (480,780) | 365 (239,542) | 67 (35,115) | 461 (305,649) | 431 (302,569) | 491 (366,640) | 440 (349,550) |
| 11 | 295 (206,397) | 422 (289,584) | 525 (408,657) | 308 (208,446) | 57 (29,98) | 382 (240,541) | 366 (259,481) | 414 (316,517) | 352 (277,437) |
| <12 | 220 (175,277) | 581 (504,674) | 545 (472,620) | 322 (220,428) | 43 (24,68) | 299 (223,393) | 273 (224,329) | 332 (272,402) | 377 (343,424) |
| 12-14 | 203 (129,296) | 380 (290,484) | 436 (356,524) | 256 (172,353) | 39 (19,67) | 386 (234,560) | 247 (163,347) | 400 (291,525) | 308 (256,360) |
| 15-17 | 185 (77,320) | 304 (215,414) | 359 (283,448) | 210 (127,304) | 35 (14,71) | 337 (206,510) | 227 (98,384) | 343 (235,491) | 251 (196,315) |
| 18-20 | 258 (131,419) | 255 (163,357) | 349 (270,449) | 205 (136,299) | 50 (21,91) | 288 (161,446) | 318 (154,528) | 314 (205,447) | 244 (188,308) |
| 21-23 | 427 (230,665) | 217 (129,320) | 351 (265,459) | 208 (131,292) | 80 (36,159) | 288 (166,457) | 523 (283,793) | 286 (168,427) | 242 (180,311) |
| 12-23 | 231 (171,310) | 296 (238,360) | 374 (321,432) | 219 (145,302) | 45 (24,73) | 323 (244,413) | 287 (214,368) | 338 (266,425) | 260 (226,296) |
| <24 | 231 (185,284) | 526 (454,610) | 511 (448,581) | 300 (205,406) | 45 (25,70) | 309 (247,374) | 284 (241,335) | 334 (282,397) | 355 (323,391) |
| 24-35 | 215 (140,302) | 185 (131,247) | 267 (219,329) | 159 (105,229) | 41 (20,74) | 245 (179,322) | 266 (171,372) | 253 (189,335) | 188 (156,224) |
| 36-47 | 225 (121,361) | 150 (98,218) | 267 (203,337) | 156 (101,224) | 42 (20,78) | 300 (216,399) | 274 (156,417) | 291 (204,397) | 187 (144,235) |
| 48-59 | 64 (15,144) | 39 (9,87) | 123 (82,177) | 71 (43,115) | 12 (3,31) | 183 (121,261) | 79 (19,171) | 173 (103,262) | 86 (53,122) |
| 24-59 | 184 (127,249) | 153 (119,195) | 240 (201,279) | 141 (96,199) | 36 (18,60) | 246 (197,302) | 225 (162,299) | 240 (192,304) | 166 (142,194) |
| <60 | 198 (161,240) | 407 (350,470) | 402 (355,455) | 241 (166,328) | 39 (22,62) | 263 (224,311) | 245 (207,284) | 274 (230,325) | 282 (257,313) |
| 2011 |  |  |  |  |  |  |  |  |  |
| <1 | 47 (20,80) | 91 (60,127) | 114 (77,154) | 67 (41,101) | 9 (3,18) | 78 (0,206) | 56 (24,94) | 67 (37,105) | 73 (52,99) |
| 1 | 81 (35,135) | 205 (156,261) | 197 (149,251) | 116 (75,171) | 16 (6,30) | 125 (0,323) | 100 (46,167) | 117 (66,185) | 161 (127,199) |
| 2 | 98 (51,155) | 219 (166,285) | 237 (185,294) | 139 (91,196) | 19 (8,36) | 113 (0,275) | 121 (64,188) | 141 (87,200) | 144 (112,181) |
| 3 | 225 (159,309) | 656 (516,811) | 562 (456,682) | 327 (225,450) | 44 (22,71) | 293 (155,454) | 277 (201,359) | 319 (240,410) | 430 (361,511) |
| 4 | 365 (265,494) | 1007 (796,1246) | 918 (756,1123) | 528 (354,748) | 71 (38,120) | 419 (217,673) | 456 (334,583) | 525 (404,669) | 622 (525,748) |
| 5 | 356 (248,479) | 904 (713,1138) | 870 (713,1068) | 517 (350,741) | 68 (35,113) | 448 (237,738) | 431 (318,570) | 497 (380,643) | 558 (459,669) |
| 6 | 221 (147,306) | 509 (372,684) | 460 (356,591) | 272 (176,380) | 42 (22,73) | 305 (173,478) | 270 (180,376) | 359 (268,477) | 350 (276,431) |
| 7 | 245 (154,350) | 526 (343,736) | 510 (392,652) | 299 (193,435) | 48 (23,81) | 387 (205,602) | 303 (205,423) | 400 (289,527) | 324 (240,410) |
| 8 | 191 (124,272) | 322 (203,454) | 400 (290,515) | 233 (144,342) | 37 (19,66) | 289 (155,447) | 235 (157,327) | 310 (232,407) | 283 (212,364) |
| 9 | 271 (192,361) | 460 (332,615) | 481 (380,599) | 285 (188,397) | 53 (29,88) | 337 (217,473) | 328 (240,436) | 380 (287,495) | 346 (280,428) |
| 10 | 313 (216,417) | 522 (384,706) | 552 (429,686) | 318 (205,459) | 60 (32,96) | 412 (272,573) | 381 (268,507) | 435 (334,565) | 388 (307,480) |
| 11 | 274 (193,364) | 397 (270,552) | 496 (379,610) | 287 (189,412) | 54 (28,90) | 355 (215,502) | 341 (248,447) | 390 (292,502) | 326 (254,406) |
| <12 | 220 (172,274) | 581 (498,670) | 544 (476,622) | 323 (216,433) | 43 (23,69) | 301 (222,396) | 274 (224,329) | 332 (271,396) | 379 (342,425) |
| 12-14 | 156 (101,230) | 298 (230,380) | 343 (285,405) | 204 (135,277) | 30 (16,53) | 306 (179,431) | 195 (131,276) | 319 (231,415) | 240 (202,284) |
| 15-17 | 155 (64,272) | 260 (180,342) | 302 (233,384) | 177 (117,256) | 30 (10,58) | 287 (166,429) | 191 (82,326) | 287 (194,388) | 212 (168,269) |
| 18-20 | 194 (93,329) | 197 (131,276) | 269 (203,347) | 156 (99,227) | 38 (16,75) | 223 (127,342) | 244 (124,385) | 235 (152,346) | 186 (143,237) |
| 21-23 | 328 (182,504) | 168 (102,251) | 274 (204,353) | 161 (105,234) | 63 (29,121) | 228 (131,353) | 410 (228,614) | 226 (132,340) | 192 (142,247) |
| 12-23 | 183 (135,237) | 234 (191,283) | 295 (253,345) | 174 (116,236) | 36 (20,58) | 258 (199,326) | 227 (172,288) | 269 (216,329) | 207 (179,236) |
| <24 | 203 (164,251) | 470 (401,538) | 452 (401,511) | 268 (184,362) | 40 (22,62) | 275 (224,336) | 251 (213,296) | 296 (245,353) | 316 (285,351) |
| 24-35 | 192 (116,279) | 166 (122,217) | 246 (198,295) | 144 (94,204) | 38 (19,64) | 222 (165,289) | 240 (155,342) | 230 (168,308) | 171 (142,204) |
| 36-47 | 172 (86,266) | 116 (72,170) | 207 (161,262) | 122 (75,174) | 34 (14,63) | 234 (167,307) | 211 (120,331) | 222 (152,300) | 144 (113,179) |
| 48-59 | 50 (12,113) | 30 (7,68) | 94 (60,138) | 55 (32,89) | 10 (2,23) | 142 (91,200) | 60 (14,132) | 133 (77,203) | 67 (45,91) |
| 24-59 | 147 (104,201) | 124 (95,158) | 197 (164,237) | 116 (76,161) | 29 (15,50) | 201 (165,243) | 184 (134,240) | 198 (157,248) | 137 (118,160) |
| <60 | 169 (136,208) | 345 (298,403) | 345 (302,385) | 203 (142,272) | 34 (18,54) | 221 (185,264) | 210 (177,244) | 232 (193,276) | 239 (216,264) |
| 2012 |  |  |  |  |  |  |  |  |  |
| <1 | 35 (15,59) | 67 (44,96) | 85 (59,114) | 49 (30,75) | 7 (2,14) | 57 (0,149) | 42 (18,73) | 50 (26,79) | 54 (37,75) |
| 1 | 53 (24,90) | 137 (102,175) | 129 (99,165) | 76 (51,108) | 10 (4,19) | 81 (0,202) | 66 (30,109) | 77 (41,118) | 106 (82,129) |
| 2 | 95 (49,148) | 214 (156,278) | 231 (182,291) | 135 (90,196) | 18 (8,34) | 111 (0,285) | 117 (59,187) | 138 (86,198) | 141 (110,176) |
| 3 | 231 (164,308) | 668 (528,826) | 576 (463,696) | 336 (214,478) | 45 (24,74) | 300 (159,488) | 287 (206,378) | 330 (244,429) | 446 (369,531) |
| 4 | 257 (182,342) | 705 (551,870) | 634 (525,771) | 375 (245,532) | 50 (26,85) | 290 (155,462) | 317 (235,415) | 368 (285,460) | 436 (362,520) |
| 5 | 262 (187,357) | 680 (524,868) | 650 (532,781) | 379 (253,536) | 50 (27,82) | 335 (179,515) | 323 (232,415) | 371 (279,482) | 416 (340,503) |
| 6 | 175 (119,246) | 410 (295,550) | 367 (282,461) | 213 (139,312) | 34 (18,58) | 248 (142,380) | 215 (150,294) | 288 (210,374) | 277 (219,345) |
| 7 | 152 (94,213) | 328 (214,459) | 315 (240,399) | 184 (118,273) | 29 (14,52) | 239 (132,369) | 187 (118,261) | 244 (171,323) | 199 (151,260) |
| 8 | 206 (136,289) | 347 (227,499) | 429 (314,567) | 257 (162,371) | 39 (20,69) | 307 (174,476) | 253 (167,354) | 337 (239,437) | 306 (225,397) |
| 9 | 298 (215,400) | 504 (356,667) | 524 (416,663) | 306 (199,435) | 56 (29,94) | 363 (235,537) | 368 (268,487) | 413 (315,516) | 377 (303,465) |
| 10 | 293 (207,402) | 496 (367,673) | 526 (420,658) | 304 (201,431) | 58 (30,98) | 394 (255,554) | 359 (258,473) | 412 (315,526) | 369 (290,454) |
| 11 | 206 (145,274) | 294 (202,400) | 370 (286,465) | 216 (142,303) | 41 (21,66) | 265 (177,364) | 252 (181,334) | 289 (221,368) | 247 (191,302) |
| <12 | 186 (147,232) | 490 (421,569) | 459 (400,518) | 269 (184,357) | 37 (19,59) | 254 (192,331) | 230 (187,279) | 277 (230,333) | 320 (287,356) |
| 12-14 | 167 (109,241) | 321 (247,413) | 368 (300,447) | 215 (141,303) | 33 (16,56) | 323 (196,458) | 209 (134,297) | 338 (248,447) | 256 (212,304) |
| 15-17 | 158 (64,265) | 258 (185,354) | 309 (235,387) | 181 (122,263) | 29 (11,61) | 287 (172,425) | 190 (82,323) | 294 (206,404) | 216 (172,264) |
| 18-20 | 193 (91,310) | 191 (123,270) | 263 (202,336) | 153 (100,216) | 37 (15,71) | 215 (117,341) | 243 (118,393) | 228 (146,335) | 182 (140,229) |
| 21-23 | 331 (179,514) | 171 (101,260) | 276 (208,360) | 161 (97,240) | 64 (30,115) | 232 (124,352) | 409 (227,616) | 229 (140,333) | 191 (145,244) |
| 12-23 | 188 (135,245) | 238 (195,286) | 301 (256,349) | 176 (125,242) | 37 (20,60) | 262 (201,329) | 231 (172,293) | 271 (217,334) | 210 (184,239) |
| <24 | 190 (150,233) | 436 (372,502) | 418 (366,472) | 246 (172,328) | 37 (20,58) | 256 (206,306) | 234 (195,279) | 274 (227,324) | 292 (260,326) |
| 24-35 | 160 (105,233) | 139 (102,187) | 203 (164,248) | 118 (80,171) | 31 (15,55) | 186 (140,242) | 198 (133,279) | 191 (139,247) | 143 (117,171) |
| 36-47 | 168 (92,266) | 117 (74,164) | 202 (159,256) | 117 (73,173) | 33 (14,62) | 231 (165,303) | 206 (109,317) | 217 (154,300) | 141 (111,177) |
| 48-59 | 45 (10,98) | 27 (6,58) | 86 (56,121) | 50 (29,78) | 9 (1,22) | 129 (82,183) | 55 (13,120) | 120 (70,186) | 60 (39,86) |
| 24-59 | 134 (95,185) | 112 (88,143) | 177 (150,207) | 104 (70,143) | 26 (13,44) | 181 (148,219) | 166 (115,217) | 177 (141,221) | 124 (104,144) |
| <60 | 156 (124,190) | 314 (273,362) | 314 (274,353) | 182 (121,250) | 30 (17,48) | 201 (170,241) | 189 (160,221) | 212 (179,249) | 217 (197,244) |
| 2013 |  |  |  |  |  |  |  |  |  |
| <1 | 27 (11,47) | 53 (35,74) | 67 (46,90) | 39 (25,58) | 5 (2,11) | 47 (0,121) | 34 (14,58) | 39 (21,65) | 43 (30,59) |
| 1 | 34 (16,58) | 87 (65,113) | 83 (63,105) | 49 (31,71) | 6 (3,13) | 52 (0,133) | 42 (20,70) | 50 (29,75) | 68 (53,83) |
| 2 | 119 (66,190) | 262 (194,346) | 290 (222,362) | 170 (106,234) | 23 (11,43) | 135 (0,339) | 145 (78,223) | 172 (106,258) | 174 (136,215) |
| 3 | 214 (152,287) | 618 (491,767) | 528 (434,641) | 311 (200,443) | 41 (20,69) | 277 (146,440) | 267 (190,349) | 304 (233,388) | 410 (341,483) |
| 4 | 300 (215,404) | 815 (647,1009) | 747 (617,891) | 437 (288,609) | 58 (30,98) | 343 (178,549) | 370 (274,480) | 423 (331,541) | 507 (427,598) |
| 5 | 288 (198,389) | 741 (575,923) | 702 (574,859) | 414 (280,583) | 55 (31,94) | 366 (191,587) | 346 (254,459) | 401 (301,522) | 448 (369,541) |
| 6 | 188 (121,263) | 441 (316,590) | 394 (310,499) | 231 (155,334) | 37 (18,64) | 267 (144,411) | 230 (158,318) | 308 (230,405) | 301 (236,364) |
| 7 | 172 (112,244) | 370 (245,524) | 366 (271,465) | 212 (130,313) | 33 (17,57) | 272 (152,414) | 212 (144,295) | 284 (206,374) | 226 (171,292) |
| 8 | 168 (110,238) | 282 (181,392) | 346 (253,451) | 202 (128,294) | 32 (15,57) | 250 (143,384) | 203 (133,283) | 269 (194,353) | 245 (183,310) |
| 9 | 321 (233,431) | 554 (381,736) | 579 (459,711) | 339 (226,486) | 64 (34,108) | 402 (261,575) | 401 (296,530) | 452 (345,588) | 417 (341,514) |
| 10 | 251 (177,343) | 429 (304,571) | 448 (347,562) | 262 (170,379) | 50 (26,85) | 336 (215,473) | 308 (223,402) | 353 (268,453) | 314 (245,386) |
| 11 | 230 (160,308) | 334 (225,454) | 412 (319,518) | 240 (153,346) | 44 (24,74) | 298 (188,411) | 285 (208,375) | 325 (249,412) | 276 (212,349) |
| <12 | 188 (148,231) | 488 (419,565) | 457 (401,518) | 267 (181,364) | 37 (21,59) | 253 (186,326) | 230 (188,276) | 278 (229,331) | 319 (287,353) |
| 12-14 | 147 (95,213) | 278 (210,349) | 317 (263,384) | 185 (123,256) | 28 (14,50) | 278 (175,406) | 179 (113,256) | 295 (213,390) | 221 (184,260) |
| 15-17 | 162 (68,285) | 271 (191,375) | 325 (254,405) | 189 (126,265) | 32 (11,65) | 304 (179,455) | 202 (86,341) | 308 (208,430) | 225 (183,278) |
| 18-20 | 176 (84,283) | 173 (113,246) | 239 (185,310) | 139 (90,199) | 33 (14,67) | 201 (99,307) | 221 (110,361) | 210 (133,299) | 166 (126,208) |
| 21-23 | 314 (179,489) | 165 (97,238) | 262 (195,338) | 152 (98,229) | 59 (26,116) | 216 (121,341) | 391 (223,603) | 221 (132,320) | 185 (140,233) |
| 12-23 | 177 (131,227) | 224 (184,275) | 282 (241,330) | 167 (113,220) | 33 (17,56) | 247 (186,308) | 217 (165,275) | 256 (201,310) | 196 (172,226) |
| <24 | 184 (146,225) | 419 (361,485) | 406 (354,458) | 238 (162,319) | 36 (19,58) | 246 (201,298) | 225 (190,266) | 265 (225,314) | 282 (253,313) |
| 24-35 | 168 (108,255) | 149 (103,192) | 215 (176,263) | 126 (84,174) | 33 (16,58) | 194 (142,254) | 208 (136,298) | 204 (147,267) | 149 (124,177) |
| 36-47 | 154 (86,238) | 106 (67,155) | 186 (144,230) | 109 (70,158) | 29 (13,55) | 209 (153,278) | 188 (101,297) | 201 (139,270) | 130 (101,161) |
| 48-59 | 54 (12,116) | 32 (8,66) | 103 (67,146) | 59 (34,93) | 10 (2,25) | 153 (99,218) | 65 (15,139) | 142 (85,221) | 71 (46,100) |
| 24-59 | 140 (98,188) | 117 (90,148) | 184 (153,216) | 108 (74,148) | 27 (14,45) | 189 (154,230) | 174 (126,230) | 185 (145,235) | 129 (110,151) |
| <60 | 156 (127,190) | 319 (275,366) | 315 (278,361) | 185 (128,251) | 31 (17,48) | 203 (172,241) | 191 (161,225) | 213 (181,251) | 220 (199,245) |
| 2014 |  |  |  |  |  |  |  |  |  |
| <1 | 11 (4,19) | 22 (15,31) | 27 (19,37) | 16 (9,24) | 2 (1,4) | 19 (0,49) | 14 (6,24) | 16 (9,26) | 17 (12,23) |
| 1 | 37 (17,62) | 95 (71,119) | 91 (69,116) | 52 (33,77) | 7 (3,14) | 58 (0,144) | 45 (22,77) | 54 (31,82) | 74 (58,91) |
| 2 | 118 (65,192) | 266 (195,348) | 291 (229,363) | 172 (113,239) | 23 (10,43) | 135 (0,361) | 145 (77,229) | 171 (109,248) | 175 (136,216) |
| 3 | 141 (100,192) | 412 (324,512) | 354 (283,432) | 207 (143,291) | 28 (15,47) | 184 (96,288) | 174 (124,228) | 202 (151,263) | 272 (224,328) |
| 4 | 209 (155,272) | 564 (451,711) | 518 (420,619) | 303 (200,431) | 40 (22,67) | 237 (118,376) | 256 (186,337) | 294 (223,376) | 352 (297,411) |
| 5 | 195 (138,260) | 504 (393,615) | 476 (383,578) | 279 (189,398) | 38 (19,63) | 248 (128,388) | 238 (171,307) | 274 (206,358) | 306 (253,367) |
| 6 | 178 (124,250) | 414 (297,539) | 374 (285,466) | 217 (141,310) | 34 (18,60) | 249 (136,379) | 217 (145,301) | 288 (215,375) | 281 (222,348) |
| 7 | 184 (122,260) | 388 (262,543) | 373 (289,484) | 220 (136,323) | 35 (18,59) | 284 (156,429) | 224 (145,308) | 295 (215,394) | 236 (176,302) |
| 8 | 236 (155,328) | 401 (256,572) | 502 (368,648) | 291 (181,414) | 46 (23,82) | 359 (202,564) | 294 (194,413) | 389 (285,507) | 351 (258,451) |
| 9 | 292 (205,398) | 498 (354,664) | 522 (420,654) | 307 (197,435) | 59 (31,98) | 361 (235,507) | 362 (261,475) | 416 (323,537) | 375 (299,459) |
| 10 | 282 (199,377) | 477 (351,630) | 501 (397,621) | 291 (190,409) | 54 (27,91) | 375 (246,523) | 348 (249,458) | 400 (296,508) | 351 (272,432) |
| 11 | 252 (183,347) | 367 (248,519) | 456 (347,572) | 266 (175,393) | 48 (27,82) | 324 (219,461) | 313 (227,423) | 356 (276,458) | 303 (238,379) |
| <12 | 180 (141,225) | 467 (402,536) | 437 (384,493) | 259 (173,348) | 35 (19,58) | 242 (178,305) | 221 (180,264) | 265 (223,320) | 305 (276,342) |
| 12-14 | 135 (86,193) | 254 (199,322) | 292 (245,351) | 171 (113,240) | 25 (12,45) | 257 (153,373) | 166 (108,232) | 269 (199,354) | 202 (169,239) |
| 15-17 | 164 (72,277) | 271 (188,371) | 321 (243,402) | 187 (128,275) | 32 (11,63) | 299 (179,451) | 200 (89,340) | 306 (203,418) | 223 (174,280) |
| 18-20 | 169 (79,274) | 167 (110,237) | 230 (177,294) | 133 (87,193) | 33 (14,63) | 189 (104,297) | 208 (102,332) | 202 (131,297) | 161 (125,205) |
| 21-23 | 337 (192,520) | 175 (102,261) | 279 (203,366) | 165 (107,240) | 64 (29,121) | 231 (121,360) | 415 (231,616) | 228 (134,350) | 194 (148,249) |
| 12-23 | 172 (126,226) | 220 (181,271) | 278 (240,323) | 164 (113,227) | 33 (17,55) | 240 (184,306) | 213 (162,269) | 248 (200,314) | 194 (168,224) |
| <24 | 178 (146,216) | 408 (350,467) | 395 (345,446) | 232 (157,311) | 34 (19,53) | 238 (195,290) | 218 (183,260) | 259 (216,302) | 274 (246,302) |
| 24-35 | 168 (108,247) | 148 (108,199) | 218 (177,265) | 129 (87,182) | 34 (17,58) | 198 (146,256) | 211 (140,306) | 205 (152,270) | 152 (125,181) |
| 36-47 | 175 (92,275) | 120 (74,176) | 212 (165,270) | 124 (81,181) | 34 (15,62) | 240 (174,317) | 214 (114,338) | 229 (155,317) | 149 (118,185) |
| 48-59 | 50 (12,107) | 32 (7,66) | 97 (61,139) | 56 (33,89) | 9 (2,24) | 146 (93,207) | 63 (15,135) | 135 (78,208) | 67 (44,96) |
| 24-59 | 146 (105,195) | 122 (94,156) | 191 (164,226) | 110 (79,154) | 28 (15,46) | 196 (158,242) | 179 (131,232) | 192 (149,240) | 133 (114,155) |
| <60 | 156 (129,189) | 317 (276,369) | 317 (280,360) | 187 (124,253) | 30 (17,49) | 205 (172,242) | 191 (164,224) | 215 (182,251) | 220 (199,245) |
| 2015 |  |  |  |  |  |  |  |  |  |
| <1 | 16 (7,28) | 32 (21,45) | 39 (28,53) | 23 (14,36) | 3 (1,6) | 28 (0,72) | 20 (8,34) | 24 (13,37) | 26 (17,35) |
| 1 | 28 (12,45) | 70 (54,91) | 68 (52,87) | 40 (26,57) | 5 (2,10) | 43 (0,113) | 34 (16,56) | 40 (23,62) | 55 (44,68) |
| 2 | 71 (40,114) | 161 (119,204) | 176 (136,221) | 102 (66,148) | 14 (6,26) | 81 (0,205) | 89 (46,140) | 104 (66,153) | 106 (83,134) |
| 3 | 139 (98,186) | 401 (313,504) | 344 (278,419) | 200 (136,286) | 27 (14,45) | 178 (89,274) | 170 (125,223) | 198 (151,255) | 265 (219,315) |
| 4 | 191 (139,252) | 519 (413,641) | 475 (392,574) | 279 (192,393) | 36 (20,60) | 214 (106,345) | 234 (170,304) | 271 (209,344) | 322 (270,383) |
| 5 | 118 (85,160) | 309 (239,383) | 296 (243,356) | 173 (116,244) | 23 (12,39) | 149 (78,240) | 147 (107,192) | 169 (127,214) | 188 (154,228) |
| 6 | 156 (108,224) | 376 (274,495) | 334 (259,416) | 197 (132,283) | 30 (15,55) | 225 (124,343) | 194 (135,260) | 261 (193,340) | 251 (197,309) |
| 7 | 150 (96,216) | 333 (222,456) | 321 (242,415) | 190 (119,268) | 30 (14,52) | 243 (142,361) | 185 (122,261) | 252 (178,329) | 201 (152,259) |
| 8 | 124 (81,178) | 211 (142,305) | 261 (189,337) | 154 (94,225) | 24 (13,40) | 187 (106,285) | 155 (107,215) | 204 (148,276) | 185 (135,237) |
| 9 | 124 (86,165) | 210 (147,276) | 221 (171,276) | 131 (85,182) | 24 (12,41) | 154 (101,218) | 151 (109,201) | 173 (133,219) | 158 (128,193) |
| 10 | 167 (117,227) | 284 (203,383) | 301 (238,377) | 172 (113,248) | 32 (17,55) | 222 (143,320) | 205 (145,273) | 234 (179,299) | 209 (165,259) |
| 11 | 157 (112,213) | 225 (157,308) | 279 (215,350) | 165 (106,239) | 30 (17,49) | 202 (134,275) | 194 (141,252) | 220 (170,280) | 184 (148,231) |
| <12 | 122 (96,153) | 320 (275,368) | 298 (263,340) | 175 (122,237) | 24 (13,38) | 166 (121,211) | 150 (122,180) | 180 (148,218) | 207 (188,231) |
| 12-14 | 100 (64,144) | 191 (144,243) | 219 (181,266) | 129 (87,179) | 20 (10,34) | 193 (120,273) | 125 (78,178) | 203 (150,265) | 154 (130,183) |
| 15-17 | 70 (30,123) | 115 (81,156) | 136 (107,171) | 79 (52,116) | 14 (5,27) | 130 (76,186) | 86 (38,146) | 130 (89,179) | 95 (75,119) |
| 18-20 | 97 (53,160) | 99 (64,138) | 134 (103,170) | 77 (49,111) | 18 (8,35) | 111 (61,169) | 121 (63,193) | 118 (73,170) | 92 (72,117) |
| 21-23 | 181 (106,282) | 95 (58,138) | 152 (112,201) | 89 (57,131) | 36 (16,65) | 124 (69,194) | 227 (123,336) | 126 (69,188) | 106 (79,136) |
| 12-23 | 98 (71,130) | 124 (103,150) | 157 (135,184) | 92 (65,128) | 19 (10,30) | 136 (104,177) | 120 (91,154) | 142 (112,176) | 110 (95,126) |
| <24 | 109 (88,135) | 253 (218,291) | 245 (215,280) | 144 (96,192) | 21 (12,35) | 150 (121,181) | 136 (115,162) | 160 (133,190) | 170 (154,191) |
| 24-35 | 114 (76,165) | 100 (72,134) | 147 (121,179) | 86 (58,119) | 23 (11,40) | 134 (99,179) | 141 (90,206) | 140 (103,180) | 102 (84,122) |
| 36-47 | 105 (54,164) | 74 (48,104) | 128 (98,162) | 76 (50,108) | 20 (9,38) | 143 (107,190) | 129 (69,201) | 138 (96,189) | 90 (70,113) |
| 48-59 | 29 (7,65) | 19 (4,41) | 58 (37,83) | 33 (19,53) | 6 (1,15) | 88 (56,122) | 37 (9,77) | 81 (48,123) | 41 (26,57) |
| 24-59 | 91 (64,122) | 76 (58,97) | 120 (101,142) | 70 (46,95) | 17 (9,29) | 122 (100,147) | 112 (80,148) | 120 (94,149) | 83 (72,97) |
| <60 | 97 (77,118) | 198 (171,227) | 197 (174,223) | 117 (81,156) | 19 (10,29) | 128 (109,149) | 120 (102,140) | 134 (112,157) | 137 (124,153) |
| 2016 |  |  |  |  |  |  |  |  |  |
| <1 | 9 (4,15) | 18 (12,24) | 22 (15,30) | 13 (8,19) | 2 (1,4) | 15 (0,41) | 11 (5,19) | 13 (7,20) | 14 (10,19) |
| 1 | 19 (9,33) | 50 (38,64) | 48 (36,62) | 29 (19,40) | 4 (1,8) | 30 (0,75) | 24 (11,41) | 29 (17,44) | 39 (31,48) |
| 2 | 108 (59,173) | 241 (181,314) | 263 (205,328) | 154 (100,221) | 21 (9,39) | 122 (0,316) | 134 (71,205) | 158 (96,229) | 159 (124,200) |
| 3 | 103 (73,139) | 300 (232,373) | 256 (208,312) | 151 (97,214) | 20 (11,33) | 132 (73,209) | 126 (92,164) | 146 (111,187) | 197 (164,236) |
| 4 | 157 (112,211) | 429 (335,525) | 389 (312,474) | 228 (149,314) | 31 (17,52) | 184 (97,286) | 194 (141,250) | 224 (174,285) | 267 (224,318) |
| 5 | 168 (122,225) | 439 (346,553) | 419 (342,517) | 245 (163,344) | 33 (17,55) | 215 (122,341) | 208 (152,272) | 241 (184,305) | 270 (220,325) |
| 6 | 165 (112,233) | 392 (285,503) | 353 (268,439) | 203 (134,290) | 32 (16,57) | 235 (129,349) | 207 (136,282) | 272 (199,355) | 265 (213,329) |
| 7 | 127 (80,182) | 271 (185,384) | 262 (200,334) | 155 (98,229) | 24 (12,41) | 202 (112,305) | 158 (102,219) | 205 (151,274) | 168 (125,217) |
| 8 | 96 (62,138) | 161 (105,232) | 198 (148,259) | 118 (72,169) | 18 (10,33) | 144 (82,215) | 118 (76,163) | 157 (114,209) | 141 (104,185) |
| 9 | 226 (162,312) | 387 (271,523) | 407 (324,501) | 240 (161,341) | 44 (24,75) | 283 (186,392) | 280 (204,371) | 318 (248,409) | 292 (235,358) |
| 10 | 253 (179,345) | 432 (311,574) | 455 (355,571) | 262 (175,375) | 49 (25,83) | 337 (220,465) | 315 (223,411) | 356 (268,455) | 320 (255,391) |
| 11 | 239 (170,320) | 348 (233,469) | 426 (321,542) | 252 (163,364) | 46 (25,79) | 308 (202,433) | 295 (216,397) | 337 (259,426) | 286 (224,357) |
| <12 | 131 (102,167) | 344 (298,401) | 324 (283,367) | 191 (131,252) | 26 (14,41) | 179 (133,229) | 163 (133,199) | 197 (163,237) | 226 (204,254) |
| 12-14 | 126 (81,184) | 235 (180,301) | 270 (222,326) | 158 (103,222) | 24 (12,42) | 240 (148,345) | 155 (97,216) | 249 (184,325) | 189 (158,224) |
| 15-17 | 100 (42,176) | 164 (114,225) | 195 (150,247) | 115 (76,166) | 18 (7,40) | 185 (109,270) | 120 (51,207) | 187 (122,268) | 136 (107,166) |
| 18-20 | 150 (82,252) | 152 (100,209) | 207 (157,265) | 120 (75,179) | 29 (12,58) | 171 (92,258) | 186 (93,301) | 184 (117,263) | 144 (111,182) |
| 21-23 | 192 (110,305) | 100 (58,148) | 159 (121,211) | 96 (59,139) | 38 (17,71) | 130 (74,204) | 239 (136,365) | 131 (78,191) | 112 (83,145) |
| 12-23 | 128 (93,167) | 163 (135,201) | 207 (179,240) | 122 (82,166) | 25 (12,42) | 179 (138,229) | 159 (119,205) | 188 (149,231) | 143 (125,163) |
| <24 | 132 (107,163) | 301 (257,350) | 291 (255,330) | 173 (114,234) | 26 (14,40) | 178 (144,216) | 163 (135,194) | 192 (158,230) | 203 (184,227) |
| 24-35 | 119 (75,169) | 102 (74,137) | 149 (120,184) | 88 (57,123) | 23 (12,40) | 136 (101,178) | 146 (100,207) | 140 (103,187) | 105 (86,125) |
| 36-47 | 137 (74,223) | 95 (59,140) | 167 (129,212) | 98 (64,140) | 26 (13,49) | 192 (139,250) | 171 (96,268) | 183 (125,251) | 117 (93,146) |
| 48-59 | 34 (8,79) | 22 (5,46) | 69 (43,97) | 40 (23,64) | 7 (1,17) | 102 (65,145) | 43 (10,92) | 95 (54,144) | 47 (31,67) |
| 24-59 | 105 (74,142) | 88 (68,110) | 138 (117,164) | 81 (54,111) | 20 (10,34) | 141 (114,174) | 129 (92,173) | 137 (109,173) | 96 (82,112) |
| <60 | 114 (91,140) | 233 (201,267) | 231 (205,262) | 136 (93,190) | 22 (12,34) | 150 (126,176) | 141 (121,164) | 157 (133,184) | 162 (145,179) |
| 2017 |  |  |  |  |  |  |  |  |  |
| <1 | 22 (9,38) | 44 (30,62) | 55 (40,74) | 32 (19,50) | 4 (2,8) | 37 (0,95) | 27 (12,46) | 33 (17,52) | 36 (25,48) |
| 1 | 34 (16,58) | 89 (68,114) | 85 (63,109) | 50 (31,70) | 7 (3,13) | 53 (0,136) | 43 (19,72) | 50 (29,76) | 69 (54,85) |
| 2 | 95 (47,151) | 213 (159,275) | 234 (181,288) | 138 (90,192) | 18 (8,35) | 109 (0,277) | 118 (59,186) | 140 (90,199) | 143 (112,177) |
| 3 | 185 (133,251) | 533 (412,665) | 460 (368,561) | 264 (176,379) | 36 (19,60) | 240 (134,369) | 225 (162,298) | 261 (197,338) | 353 (294,422) |
| 4 | 259 (186,348) | 700 (564,868) | 648 (528,774) | 378 (250,520) | 50 (26,82) | 296 (153,479) | 322 (234,416) | 365 (274,462) | 439 (361,525) |
| 5 | 233 (167,314) | 601 (468,761) | 572 (466,700) | 335 (224,477) | 44 (25,74) | 297 (156,457) | 280 (207,370) | 327 (247,433) | 367 (303,446) |
| 6 | 136 (90,191) | 327 (240,434) | 290 (223,360) | 170 (115,244) | 26 (13,44) | 195 (108,298) | 168 (114,233) | 225 (168,302) | 219 (172,271) |
| 7 | 120 (80,173) | 260 (176,361) | 250 (194,318) | 146 (92,212) | 23 (11,39) | 191 (110,289) | 149 (93,209) | 197 (145,264) | 158 (121,197) |
| 8 | 163 (104,235) | 283 (180,410) | 347 (259,463) | 203 (124,299) | 32 (16,56) | 251 (142,369) | 204 (133,292) | 271 (200,359) | 246 (183,315) |
| 9 | 210 (145,286) | 360 (256,479) | 374 (298,465) | 220 (141,308) | 41 (22,72) | 264 (172,371) | 259 (188,341) | 296 (229,372) | 269 (212,328) |
| 10 | 180 (128,240) | 304 (221,407) | 321 (250,404) | 188 (126,270) | 34 (18,59) | 238 (153,340) | 225 (160,290) | 250 (192,326) | 225 (177,272) |
| 11 | 275 (196,364) | 393 (266,545) | 489 (377,612) | 285 (186,404) | 53 (26,86) | 346 (231,493) | 342 (248,444) | 390 (301,491) | 326 (257,412) |
| <12 | 157 (124,196) | 411 (352,480) | 384 (339,437) | 226 (154,302) | 30 (17,49) | 212 (158,276) | 193 (160,234) | 234 (190,281) | 268 (241,300) |
| 12-14 | 122 (79,175) | 232 (178,300) | 265 (222,321) | 156 (105,218) | 24 (12,43) | 237 (141,344) | 153 (98,215) | 244 (181,324) | 187 (156,223) |
| 15-17 | 102 (46,177) | 173 (121,232) | 205 (160,258) | 119 (77,171) | 20 (7,41) | 192 (113,289) | 128 (54,210) | 195 (134,275) | 143 (112,175) |
| 18-20 | 158 (78,261) | 160 (105,230) | 217 (165,274) | 126 (79,179) | 31 (13,61) | 178 (96,281) | 199 (89,319) | 191 (121,281) | 152 (117,193) |
| 21-23 | 226 (132,352) | 117 (68,174) | 190 (144,246) | 111 (69,167) | 45 (20,80) | 157 (92,237) | 279 (160,425) | 156 (92,233) | 132 (100,173) |
| 12-23 | 137 (99,181) | 175 (142,211) | 221 (187,257) | 129 (87,179) | 27 (14,44) | 192 (146,248) | 167 (127,216) | 199 (160,245) | 153 (134,176) |
| <24 | 147 (122,183) | 341 (293,393) | 328 (290,375) | 191 (131,259) | 29 (16,46) | 199 (162,246) | 183 (153,216) | 215 (179,257) | 228 (207,253) |
| 24-35 | 143 (90,205) | 124 (89,166) | 181 (148,222) | 106 (70,148) | 27 (13,49) | 165 (120,218) | 175 (119,245) | 169 (124,219) | 125 (104,152) |
| 36-47 | 125 (69,199) | 85 (55,123) | 150 (117,189) | 89 (56,124) | 23 (11,45) | 171 (124,220) | 151 (83,233) | 161 (112,224) | 104 (82,131) |
| 48-59 | 36 (9,77) | 22 (5,48) | 68 (43,98) | 41 (24,63) | 7 (1,18) | 103 (65,147) | 44 (10,100) | 96 (57,147) | 48 (32,68) |
| 24-59 | 109 (77,147) | 91 (70,115) | 143 (120,170) | 85 (57,117) | 21 (11,36) | 146 (117,179) | 134 (98,177) | 143 (116,180) | 99 (84,116) |
| <60 | 124 (98,150) | 250 (216,290) | 250 (220,283) | 149 (100,200) | 24 (13,38) | 161 (138,192) | 152 (129,179) | 170 (142,200) | 175 (158,193) |
| 2018 |  |  |  |  |  |  |  |  |  |
| <1 | 22 (11,37) | 42 (28,60) | 519 (462,580) | 32 (19,47) | 4 (2,8) | 37 (0,97) | 27 (12,46) | 32 (16,50) | 34 (24,47) |
| 1 | 42 (19,71) | 105 (79,136) | 882 (788,986) | 58 (37,86) | 8 (3,15) | 64 (0,170) | 51 (25,85) | 59 (35,90) | 82 (64,100) |
| 2 | 97 (55,157) | 222 (160,289) | 1577 (1397,1786) | 139 (89,205) | 19 (9,35) | 113 (0,289) | 121 (62,187) | 144 (92,205) | 145 (114,182) |
| 3 | 169 (118,227) | 485 (378,605) | 2227 (1973,2503) | 244 (165,345) | 32 (16,54) | 213 (114,341) | 206 (149,269) | 239 (181,304) | 321 (265,378) |
| 4 | 186 (133,249) | 512 (405,624) | 2208 (1970,2488) | 273 (187,386) | 37 (20,61) | 216 (111,339) | 232 (170,299) | 269 (206,343) | 319 (263,376) |
| 5 | 251 (180,342) | 652 (497,818) | 3328 (2918,3704) | 370 (254,511) | 48 (27,79) | 324 (179,495) | 312 (226,413) | 359 (274,463) | 401 (329,483) |
| 6 | 175 (113,249) | 410 (306,540) | 3307 (2930,3710) | 211 (136,302) | 34 (18,59) | 243 (133,364) | 215 (148,292) | 283 (208,363) | 272 (216,339) |
| 7 | 178 (116,258) | 395 (254,545) | 3907 (3451,4416) | 222 (149,325) | 35 (18,62) | 287 (160,438) | 221 (149,309) | 294 (214,389) | 236 (178,302) |
| 8 | 175 (115,246) | 293 (193,416) | 3586 (3179,4004) | 216 (138,316) | 34 (17,59) | 265 (146,381) | 214 (140,295) | 285 (210,375) | 257 (189,328) |
| 9 | 263 (189,357) | 448 (316,603) | 2804 (2466,3169) | 275 (182,390) | 51 (27,85) | 323 (211,461) | 323 (237,426) | 370 (289,469) | 337 (271,412) |
| 10 | 276 (194,374) | 468 (340,628) | 3156 (2809,3565) | 289 (185,411) | 53 (28,90) | 368 (233,528) | 336 (240,445) | 380 (295,500) | 347 (280,424) |
| 11 | 232 (163,317) | 330 (227,448) | 2565 (2266,2904) | 241 (153,344) | 46 (24,76) | 298 (191,418) | 285 (212,375) | 323 (248,420) | 276 (215,346) |
| <12 | 175 (139,214) | 456 (394,526) | 2490 (2211,2802) | 249 (171,335) | 34 (18,53) | 235 (174,309) | 215 (178,261) | 259 (215,313) | 297 (268,332) |
| 12-14 | 144 (93,205) | 272 (206,350) | 2496 (2202,2831) | 185 (118,256) | 27 (13,48) | 274 (167,400) | 176 (115,253) | 288 (204,377) | 216 (183,259) |
| 15-17 | 132 (61,222) | 220 (151,298) | 2755 (2453,3157) | 151 (96,218) | 26 (10,50) | 245 (151,368) | 164 (72,275) | 248 (169,344) | 180 (142,228) |
| 18-20 | 136 (65,222) | 137 (91,194) | 2255 (2006,2547) | 108 (72,160) | 26 (11,50) | 156 (85,239) | 169 (81,271) | 166 (107,241) | 131 (103,162) |
| 21-23 | 250 (143,407) | 131 (79,193) | 2221 (1958,2491) | 121 (77,180) | 49 (23,92) | 169 (95,265) | 312 (169,477) | 175 (107,255) | 146 (108,188) |
| 12-23 | 147 (108,192) | 188 (153,230) | 2395 (2137,2704) | 140 (95,192) | 29 (15,46) | 206 (156,266) | 182 (136,234) | 215 (170,263) | 165 (144,190) |
| <24 | 162 (129,198) | 371 (321,434) | 2430 (2148,2734) | 210 (143,285) | 32 (16,48) | 218 (173,267) | 199 (170,234) | 236 (199,279) | 248 (225,278) |
| 24-35 | 147 (95,209) | 127 (92,170) | 2487 (2224,2801) | 109 (73,152) | 29 (14,52) | 169 (124,222) | 181 (116,254) | 177 (128,229) | 129 (108,157) |
| 36-47 | 135 (76,214) | 96 (62,138) | 2342 (2074,2676) | 99 (64,138) | 27 (12,51) | 190 (140,249) | 171 (90,266) | 182 (123,249) | 117 (89,146) |
| 48-59 | 39 (9,81) | 24 (5,52) | 2212 (1975,2507) | 44 (26,70) | 8 (2,18) | 113 (74,161) | 48 (11,105) | 105 (65,161) | 54 (34,74) |
| 24-59 | 117 (86,160) | 99 (74,123) | 2349 (2092,2666) | 90 (60,126) | 23 (12,39) | 159 (129,192) | 145 (107,194) | 156 (123,195) | 108 (92,127) |
| <60 | 132 (106,161) | 272 (234,312) | 2281 (2008,2569) | 158 (107,216) | 26 (15,40) | 174 (149,205) | 164 (136,194) | 183 (154,216) | 188 (171,209) |

**Table S4: Annual rates of non-medically attended acute respiratory illness per 1,000 children by regions and age in months, 2010-2018**

| Year and Age in months | Central Rate (95% CI) | Coast Rate (95% CI) | Eastern Rate (95% CI) | North Eastern Rate (95% CI) | Nairobi Rate (95% CI) | Nyanza Rate (95% CI) | Rift Valley Rate (95% CI) | Western Rate (95% CI) | Kenya Rate (95% CI) |
| --- | --- | --- | --- | --- | --- | --- | --- | --- | --- |
| 2010-2018 |  |  |  |  |  |  |  |  |  |
| <1 | 368 (314,434) | 555 (477,634) | 412 (365,462) | 899 (623,1229) | 89 (53,137) | 405 (361,461) | 379 (345,424) | 785 (681,899) | 430 (392,473) |
| 1 | 661 (558,774) | 990 (868,1134) | 738 (648,833) | 1613 (1119,2216) | 156 (86,247) | 726 (643,818) | 678 (610,756) | 1416 (1205,1623) | 772 (707,847) |
| 2 | 1147 (966,1323) | 1712 (1498,1957) | 1274 (1139,1419) | 2783 (1847,3757) | 269 (151,409) | 1253 (1108,1408) | 1176 (1058,1310) | 2439 (2112,2816) | 1334 (1205,1463) |
| 3 | 1596 (1325,1870) | 2389 (2089,2717) | 1773 (1583,1992) | 3934 (2693,5258) | 378 (211,570) | 1753 (1557,1980) | 1636 (1479,1816) | 3410 (2946,3915) | 1857 (1700,2042) |
| 4 | 1856 (1580,2151) | 2785 (2456,3142) | 2091 (1831,2356) | 4572 (3111,6065) | 447 (241,688) | 2047 (1820,2312) | 1907 (1725,2124) | 3994 (3471,4570) | 2179 (2002,2389) |
| 5 | 2097 (1760,2448) | 3156 (2768,3623) | 2341 (2090,2659) | 5095 (3503,6744) | 498 (277,780) | 2298 (2031,2589) | 2161 (1947,2409) | 4474 (3863,5171) | 2451 (2234,2699) |
| 6 | 2312 (1941,2686) | 3463 (3054,3955) | 2578 (2296,2891) | 5657 (3892,7581) | 558 (319,854) | 2517 (2257,2851) | 2359 (2145,2627) | 4886 (4251,5615) | 2688 (2468,2943) |
| 7 | 2339 (1976,2752) | 3522 (3044,3976) | 2623 (2329,2940) | 5652 (3894,7658) | 564 (315,847) | 2571 (2279,2922) | 2405 (2180,2688) | 4975 (4322,5762) | 2737 (2507,2997) |
| 8 | 2224 (1883,2604) | 3355 (2927,3806) | 2480 (2204,2784) | 5471 (3770,7375) | 523 (279,807) | 2443 (2156,2773) | 2291 (2072,2541) | 4749 (4117,5534) | 2596 (2373,2848) |
| 9 | 1810 (1519,2116) | 2694 (2375,3071) | 2010 (1786,2272) | 4411 (3063,5866) | 424 (241,665) | 1970 (1756,2239) | 1846 (1663,2072) | 3838 (3351,4413) | 2099 (1922,2303) |
| 10 | 1979 (1681,2278) | 2949 (2580,3376) | 2189 (1966,2474) | 4781 (3232,6438) | 467 (259,727) | 2152 (1912,2437) | 2021 (1832,2254) | 4205 (3592,4803) | 2294 (2099,2529) |
| 11 | 1743 (1464,2065) | 2607 (2292,2947) | 1945 (1725,2184) | 4239 (2860,5630) | 417 (234,644) | 1903 (1670,2124) | 1784 (1608,1981) | 3716 (3205,4273) | 2023 (1850,2233) |
| <12 | 1657 (1407,1970) | 2492 (2183,2848) | 1844 (1626,2080) | 4086 (2835,5395) | 396 (223,595) | 1809 (1605,2058) | 1705 (1527,1883) | 3544 (3053,4033) | 1933 (1770,2116) |
| 12-14 | 1655 (1399,1973) | 2486 (2167,2814) | 1835 (1646,2068) | 4024 (2796,5558) | 399 (213,619) | 1808 (1600,2034) | 1698 (1523,1901) | 3537 (3068,4052) | 1927 (1761,2127) |
| 15-17 | 1860 (1583,2199) | 2783 (2430,3155) | 2064 (1820,2320) | 4474 (3020,6159) | 434 (250,687) | 2041 (1821,2298) | 1907 (1727,2121) | 3955 (3465,4528) | 2167 (1986,2380) |
| 18-20 | 1870 (1581,2170) | 2787 (2409,3168) | 2067 (1851,2335) | 4520 (3110,6069) | 434 (240,668) | 2036 (1805,2305) | 1909 (1728,2104) | 3989 (3452,4569) | 2171 (1974,2387) |
| 21-23 | 1658 (1402,1955) | 2513 (2196,2874) | 1857 (1650,2088) | 4074 (2776,5651) | 397 (220,612) | 1828 (1625,2045) | 1709 (1544,1909) | 3554 (3063,4090) | 1941 (1777,2137) |
| 12-23 | 1750 (1468,2023) | 2609 (2275,2985) | 1943 (1743,2188) | 4231 (2862,5808) | 416 (218,642) | 1909 (1701,2155) | 1793 (1614,1996) | 3725 (3196,4283) | 2027 (1852,2228) |
| <24 | 1698 (1422,1962) | 2534 (2196,2893) | 1878 (1677,2131) | 4117 (2820,5565) | 401 (232,612) | 1850 (1637,2106) | 1739 (1564,1946) | 3617 (3121,4164) | 1975 (1806,2170) |
| 24-35 | 1785 (1513,2079) | 2670 (2330,3080) | 1989 (1770,2244) | 4329 (3039,5903) | 426 (239,648) | 1943 (1741,2195) | 1833 (1653,2034) | 3799 (3271,4402) | 2069 (1897,2282) |
| 36-47 | 1736 (1461,2043) | 2599 (2262,2960) | 1936 (1716,2157) | 4265 (2852,5698) | 418 (221,647) | 1907 (1699,2144) | 1782 (1610,1990) | 3694 (3255,4269) | 2023 (1851,2229) |
| 48-59 | 1650 (1398,1924) | 2481 (2179,2826) | 1847 (1635,2061) | 3986 (2735,5544) | 397 (230,589) | 1808 (1590,2048) | 1700 (1535,1888) | 3517 (3033,4042) | 1930 (1764,2132) |
| 24-59 | 1715 (1452,2024) | 2566 (2242,2875) | 1907 (1711,2139) | 4223 (2881,5576) | 409 (231,631) | 1880 (1663,2107) | 1763 (1592,1964) | 3629 (3119,4240) | 1995 (1817,2200) |
| <60 | 1625 (1374,1923) | 2454 (2116,2811) | 1817 (1629,2058) | 3959 (2618,5392) | 391 (213,601) | 1775 (1568,2010) | 1670 (1506,1844) | 3469 (3015,3993) | 1896 (1733,2088) |
| 2010 |  |  |  |  |  |  |  |  |  |
| <1 | 429 (364,501) | 644 (560,734) | 480 (424,539) | 1048 (713,1390) | 100 (55,160) | 473 (412,534) | 442 (399,491) | 925 (793,1053) | 504 (459,556) |
| 1 | 1311 (1116,1528) | 1979 (1730,2251) | 1462 (1291,1660) | 3170 (2177,4262) | 311 (179,491) | 1434 (1280,1614) | 1346 (1210,1501) | 2805 (2411,3214) | 1533 (1402,1680) |
| 2 | 1809 (1509,2132) | 2711 (2356,3090) | 2000 (1760,2252) | 4387 (3013,5786) | 421 (233,647) | 1966 (1751,2205) | 1840 (1668,2061) | 3831 (3321,4368) | 2092 (1909,2295) |
| 3 | 2142 (1814,2518) | 3219 (2820,3655) | 2402 (2121,2719) | 5213 (3541,7147) | 509 (295,783) | 2358 (2082,2646) | 2215 (1993,2455) | 4569 (3952,5274) | 2504 (2285,2730) |
| 4 | 2167 (1823,2539) | 3253 (2848,3739) | 2421 (2149,2717) | 5314 (3425,7129) | 516 (291,796) | 2378 (2119,2666) | 2234 (2018,2481) | 4643 (3981,5290) | 2524 (2325,2775) |
| 5 | 2924 (2462,3394) | 4369 (3787,5024) | 3239 (2863,3649) | 7089 (4703,9574) | 686 (405,1023) | 3199 (2841,3590) | 2979 (2673,3331) | 6251 (5352,7190) | 3401 (3116,3746) |
| 6 | 3101 (2605,3590) | 4637 (4047,5270) | 3442 (3064,3880) | 7538 (5019,10287) | 739 (404,1153) | 3370 (3012,3809) | 3159 (2878,3543) | 6577 (5568,7586) | 3596 (3305,3983) |
| 7 | 2577 (2172,3033) | 3870 (3394,4405) | 2873 (2556,3267) | 6259 (4302,8475) | 611 (326,945) | 2827 (2501,3188) | 2638 (2396,2961) | 5489 (4784,6288) | 3009 (2738,3284) |
| 8 | 2199 (1836,2596) | 3306 (2923,3787) | 2454 (2184,2760) | 5333 (3587,7354) | 520 (293,828) | 2420 (2115,2706) | 2259 (2055,2508) | 4704 (4037,5395) | 2562 (2359,2822) |
| 9 | 2120 (1787,2506) | 3179 (2765,3656) | 2358 (2097,2648) | 5107 (3361,7003) | 505 (282,754) | 2323 (2064,2595) | 2178 (1968,2407) | 4516 (3912,5264) | 2472 (2264,2716) |
| 10 | 2647 (2230,3157) | 3944 (3448,4519) | 2932 (2617,3288) | 6416 (4390,8743) | 618 (355,968) | 2884 (2566,3277) | 2701 (2445,3008) | 5636 (4890,6418) | 3073 (2805,3366) |
| 11 | 2140 (1815,2531) | 3213 (2807,3655) | 2388 (2123,2663) | 5222 (3643,7097) | 504 (272,786) | 2351 (2078,2657) | 2201 (1986,2449) | 4583 (3995,5251) | 2494 (2273,2742) |
| <12 | 2065 (1768,2415) | 3123 (2758,3582) | 2326 (2057,2617) | 5085 (3488,6702) | 485 (279,742) | 2275 (2031,2578) | 2134 (1933,2400) | 4450 (3837,5085) | 2422 (2221,2674) |
| 12-14 | 2314 (1934,2681) | 3454 (3024,3921) | 2554 (2273,2913) | 5517 (3781,7417) | 545 (300,853) | 2518 (2182,2861) | 2361 (2137,2605) | 4929 (4257,5634) | 2679 (2468,2943) |
| 15-17 | 2522 (2146,2954) | 3760 (3281,4329) | 2797 (2487,3106) | 6140 (4070,8258) | 596 (332,915) | 2748 (2436,3108) | 2579 (2343,2866) | 5375 (4663,6168) | 2923 (2666,3213) |
| 18-20 | 2776 (2361,3261) | 4165 (3631,4757) | 3124 (2766,3512) | 6801 (4783,9113) | 653 (355,1019) | 3037 (2676,3471) | 2853 (2566,3157) | 5976 (5180,6800) | 3247 (2950,3602) |
| 21-23 | 2427 (2003,2831) | 3635 (3181,4145) | 2698 (2374,3042) | 5972 (4030,7910) | 576 (309,909) | 2648 (2355,3003) | 2487 (2249,2794) | 5162 (4451,6005) | 2815 (2580,3090) |
| 12-23 | 2482 (2055,2921) | 3733 (3231,4235) | 2764 (2452,3110) | 6015 (4058,8029) | 579 (324,898) | 2715 (2397,3068) | 2540 (2291,2808) | 5314 (4589,6113) | 2885 (2635,3169) |
| <24 | 2268 (1915,2663) | 3403 (2989,3876) | 2537 (2250,2840) | 5528 (3733,7419) | 538 (310,818) | 2483 (2194,2802) | 2326 (2106,2610) | 4834 (4194,5566) | 2642 (2432,2890) |
| 24-35 | 2379 (2032,2811) | 3574 (3115,4077) | 2631 (2328,2954) | 5785 (3921,7673) | 562 (290,858) | 2608 (2273,2925) | 2446 (2189,2729) | 5079 (4386,5729) | 2769 (2537,3060) |
| 36-47 | 2465 (2080,2872) | 3690 (3240,4162) | 2746 (2423,3118) | 5962 (4129,8117) | 585 (313,889) | 2694 (2396,3013) | 2528 (2284,2840) | 5249 (4585,6051) | 2873 (2625,3152) |
| 48-59 | 2368 (1982,2810) | 3553 (3080,4083) | 2647 (2350,2968) | 5839 (3940,7630) | 560 (316,901) | 2588 (2296,2926) | 2433 (2181,2713) | 5039 (4381,5799) | 2761 (2521,3036) |
| 24-59 | 2397 (2024,2804) | 3590 (3133,4087) | 2663 (2358,2989) | 5854 (4074,8022) | 567 (314,881) | 2618 (2316,2965) | 2457 (2206,2725) | 5124 (4443,5866) | 2787 (2536,3052) |
| <60 | 2233 (1876,2609) | 3359 (2911,3819) | 2475 (2205,2782) | 5524 (3791,7551) | 532 (312,827) | 2455 (2176,2794) | 2283 (2071,2552) | 4750 (4150,5489) | 2599 (2380,2870) |
| 2011 |  |  |  |  |  |  |  |  |  |
| <1 | 719 (607,843) | 1079 (943,1236) | 804 (714,900) | 1766 (1211,2354) | 174 (101,271) | 789 (701,891) | 742 (672,824) | 1542 (1334,1755) | 841 (770,914) |
| 1 | 1146 (967,1336) | 1707 (1495,1939) | 1269 (1126,1425) | 2790 (1908,3802) | 272 (154,436) | 1244 (1099,1411) | 1167 (1057,1307) | 2416 (2092,2816) | 1329 (1212,1462) |
| 2 | 1027 (876,1200) | 1539 (1343,1756) | 1145 (1011,1304) | 2496 (1734,3370) | 246 (133,373) | 1123 (991,1275) | 1052 (953,1166) | 2199 (1887,2512) | 1199 (1099,1308) |
| 3 | 1975 (1670,2304) | 2949 (2581,3339) | 2194 (1955,2478) | 4768 (3356,6324) | 467 (262,707) | 2153 (1916,2433) | 2016 (1807,2258) | 4192 (3613,4836) | 2285 (2087,2523) |
| 4 | 2875 (2425,3391) | 4270 (3738,4868) | 3184 (2825,3562) | 6900 (4703,9390) | 670 (368,1075) | 3123 (2777,3531) | 2928 (2642,3286) | 6089 (5278,7072) | 3326 (3049,3641) |
| 5 | 3033 (2545,3535) | 4539 (3982,5202) | 3373 (2974,3802) | 7417 (5010,10192) | 719 (398,1066) | 3320 (2932,3745) | 3100 (2795,3419) | 6452 (5652,7520) | 3529 (3222,3860) |
| 6 | 2783 (2330,3249) | 4139 (3621,4715) | 3069 (2728,3465) | 6713 (4593,8956) | 663 (376,1050) | 3029 (2672,3408) | 2825 (2568,3140) | 5906 (5099,6765) | 3227 (2964,3558) |
| 7 | 3462 (2906,4059) | 5201 (4539,5997) | 3860 (3420,4334) | 8513 (5791,11112) | 829 (446,1259) | 3804 (3380,4286) | 3562 (3199,3967) | 7421 (6382,8528) | 4038 (3695,4414) |
| 8 | 2576 (2189,3050) | 3870 (3380,4426) | 2860 (2548,3225) | 6264 (4361,8561) | 614 (340,952) | 2812 (2481,3177) | 2633 (2409,2907) | 5479 (4703,6273) | 2994 (2757,3280) |
| 9 | 1900 (1606,2211) | 2850 (2476,3253) | 2114 (1881,2381) | 4661 (3200,6130) | 455 (254,719) | 2073 (1839,2373) | 1948 (1761,2166) | 4047 (3485,4637) | 2218 (2019,2443) |
| 10 | 2343 (1994,2685) | 3487 (3007,3973) | 2589 (2297,2915) | 5642 (3858,7539) | 551 (304,823) | 2540 (2250,2857) | 2385 (2145,2657) | 4956 (4299,5720) | 2719 (2487,2965) |
| 11 | 1991 (1702,2385) | 3012 (2624,3410) | 2238 (1968,2511) | 4901 (3407,6570) | 476 (269,742) | 2189 (1938,2471) | 2062 (1868,2296) | 4277 (3694,4940) | 2339 (2135,2573) |
| <12 | 2075 (1764,2422) | 3123 (2739,3572) | 2322 (2052,2605) | 5135 (3469,6767) | 495 (276,751) | 2282 (2002,2583) | 2134 (1925,2390) | 4450 (3867,5131) | 2426 (2210,2668) |
| 12-14 | 1795 (1526,2113) | 2706 (2349,3092) | 2005 (1795,2251) | 4431 (2951,5836) | 423 (239,648) | 1965 (1756,2214) | 1846 (1672,2065) | 3840 (3324,4441) | 2100 (1921,2295) |
| 15-17 | 2111 (1778,2490) | 3177 (2775,3597) | 2360 (2085,2653) | 5130 (3575,6895) | 503 (262,777) | 2309 (2031,2611) | 2167 (1950,2437) | 4494 (3903,5155) | 2465 (2251,2693) |
| 18-20 | 2125 (1777,2460) | 3184 (2793,3621) | 2358 (2104,2633) | 5184 (3534,7014) | 504 (281,784) | 2318 (2060,2615) | 2175 (1978,2418) | 4509 (3904,5196) | 2466 (2270,2727) |
| 21-23 | 1912 (1599,2219) | 2856 (2518,3251) | 2103 (1862,2386) | 4654 (3178,6111) | 446 (248,706) | 2079 (1847,2351) | 1948 (1770,2161) | 4057 (3535,4657) | 2220 (2016,2445) |
| 12-23 | 1962 (1640,2315) | 2946 (2535,3362) | 2181 (1920,2490) | 4768 (3270,6493) | 469 (268,721) | 2147 (1893,2417) | 2011 (1821,2246) | 4180 (3633,4795) | 2279 (2086,2514) |
| <24 | 2020 (1708,2369) | 3027 (2650,3451) | 2248 (1981,2503) | 4930 (3412,6627) | 475 (275,731) | 2210 (1957,2502) | 2067 (1866,2288) | 4296 (3709,4986) | 2350 (2150,2575) |
| 24-35 | 2143 (1794,2518) | 3213 (2821,3652) | 2389 (2119,2692) | 5297 (3598,7204) | 511 (305,779) | 2344 (2094,2672) | 2210 (1998,2480) | 4584 (4006,5299) | 2506 (2284,2751) |
| 36-47 | 1913 (1635,2245) | 2853 (2473,3257) | 2121 (1892,2380) | 4649 (3107,6231) | 453 (265,708) | 2091 (1843,2363) | 1959 (1778,2188) | 4048 (3475,4647) | 2218 (2035,2445) |
| 48-59 | 1830 (1521,2158) | 2724 (2382,3115) | 2028 (1798,2298) | 4405 (3010,5929) | 426 (239,667) | 1993 (1756,2261) | 1867 (1678,2067) | 3892 (3364,4440) | 2124 (1941,2340) |
| 24-59 | 1951 (1671,2251) | 2923 (2566,3368) | 2175 (1930,2467) | 4775 (3227,6497) | 462 (252,727) | 2143 (1902,2419) | 2006 (1796,2238) | 4173 (3627,4813) | 2278 (2093,2510) |
| <60 | 1898 (1597,2221) | 2841 (2505,3268) | 2117 (1878,2349) | 4634 (3240,6323) | 454 (251,713) | 2072 (1842,2353) | 1947 (1766,2154) | 4043 (3475,4639) | 2211 (2013,2423) |
| 2012 |  |  |  |  |  |  |  |  |  |
| <1 | 539 (454,632) | 805 (707,928) | 597 (530,675) | 1294 (883,1763) | 125 (68,198) | 587 (515,666) | 549 (496,606) | 1148 (990,1318) | 624 (571,691) |
| 1 | 754 (640,885) | 1131 (991,1293) | 832 (745,935) | 1831 (1266,2495) | 178 (102,274) | 825 (728,931) | 771 (694,858) | 1609 (1379,1839) | 873 (797,964) |
| 2 | 1006 (860,1169) | 1501 (1304,1721) | 1114 (985,1257) | 2421 (1688,3276) | 240 (132,367) | 1102 (973,1236) | 1025 (925,1151) | 2133 (1848,2446) | 1166 (1060,1286) |
| 3 | 2027 (1706,2354) | 3013 (2612,3455) | 2249 (1994,2524) | 4895 (3344,6638) | 480 (281,728) | 2216 (1955,2516) | 2070 (1865,2313) | 4300 (3734,5006) | 2352 (2153,2595) |
| 4 | 1976 (1658,2314) | 3002 (2622,3418) | 2212 (1975,2499) | 4855 (3166,6512) | 469 (252,742) | 2179 (1945,2481) | 2040 (1835,2273) | 4236 (3663,4866) | 2317 (2125,2548) |
| 5 | 2270 (1936,2647) | 3373 (2969,3858) | 2501 (2234,2824) | 5476 (3776,7426) | 526 (297,807) | 2463 (2165,2780) | 2318 (2099,2562) | 4782 (4117,5593) | 2621 (2397,2899) |
| 6 | 2196 (1848,2577) | 3299 (2900,3752) | 2451 (2168,2772) | 5324 (3710,7229) | 518 (293,797) | 2404 (2134,2740) | 2254 (2039,2486) | 4703 (4089,5377) | 2569 (2344,2835) |
| 7 | 2159 (1826,2508) | 3234 (2827,3687) | 2400 (2127,2705) | 5192 (3559,7142) | 512 (287,784) | 2361 (2089,2655) | 2204 (2000,2437) | 4583 (3934,5272) | 2508 (2306,2754) |
| 8 | 2772 (2341,3256) | 4148 (3608,4756) | 3092 (2745,3438) | 6884 (4762,9177) | 655 (375,1031) | 3034 (2684,3409) | 2851 (2571,3154) | 5929 (5142,6816) | 3228 (2961,3573) |
| 9 | 2088 (1765,2424) | 3118 (2694,3515) | 2321 (2050,2610) | 5050 (3417,6774) | 486 (267,756) | 2275 (2018,2547) | 2139 (1935,2367) | 4444 (3805,5094) | 2419 (2208,2677) |
| 10 | 2221 (1852,2597) | 3312 (2864,3781) | 2468 (2178,2767) | 5338 (3757,7132) | 528 (291,813) | 2426 (2135,2733) | 2265 (2047,2527) | 4711 (4111,5414) | 2574 (2351,2839) |
| 11 | 1492 (1247,1746) | 2250 (1974,2548) | 1671 (1470,1876) | 3654 (2501,4864) | 357 (203,552) | 1636 (1456,1842) | 1534 (1375,1695) | 3189 (2764,3673) | 1746 (1586,1930) |
| <12 | 1752 (1464,2042) | 2634 (2312,3027) | 1957 (1737,2181) | 4263 (2907,5669) | 414 (226,660) | 1924 (1712,2169) | 1810 (1630,2017) | 3738 (3266,4273) | 2047 (1875,2255) |
| 12-14 | 1930 (1608,2265) | 2907 (2535,3304) | 2149 (1909,2442) | 4663 (3163,6291) | 456 (266,699) | 2112 (1862,2377) | 1974 (1762,2175) | 4092 (3552,4676) | 2243 (2051,2460) |
| 15-17 | 2137 (1796,2517) | 3196 (2808,3631) | 2381 (2109,2698) | 5272 (3640,7133) | 510 (290,778) | 2332 (2075,2625) | 2194 (1975,2432) | 4555 (4005,5285) | 2483 (2287,2737) |
| 18-20 | 2084 (1740,2444) | 3126 (2760,3561) | 2315 (2059,2620) | 5088 (3460,6675) | 489 (275,765) | 2274 (2029,2583) | 2134 (1933,2385) | 4441 (3844,5071) | 2424 (2221,2671) |
| 21-23 | 1915 (1638,2236) | 2872 (2527,3259) | 2125 (1889,2383) | 4673 (3097,6257) | 452 (258,686) | 2084 (1854,2359) | 1957 (1762,2161) | 4082 (3516,4677) | 2224 (2032,2423) |
| 12-23 | 2004 (1685,2327) | 2981 (2591,3403) | 2216 (1958,2497) | 4865 (3408,6491) | 479 (269,730) | 2180 (1952,2441) | 2045 (1844,2286) | 4264 (3667,4907) | 2333 (2126,2553) |
| <24 | 1873 (1565,2184) | 2801 (2440,3202) | 2074 (1844,2316) | 4531 (3171,5991) | 443 (250,676) | 2038 (1812,2312) | 1909 (1732,2130) | 3974 (3416,4541) | 2179 (1980,2400) |
| 24-35 | 1793 (1489,2093) | 2681 (2339,3052) | 1993 (1764,2240) | 4334 (3044,5820) | 420 (243,658) | 1961 (1740,2228) | 1831 (1662,2034) | 3813 (3268,4400) | 2078 (1898,2287) |
| 36-47 | 1880 (1595,2187) | 2824 (2471,3208) | 2094 (1867,2334) | 4505 (2943,6228) | 449 (244,692) | 2061 (1825,2347) | 1928 (1745,2136) | 3997 (3465,4589) | 2180 (1997,2404) |
| 48-59 | 1649 (1387,1945) | 2465 (2151,2793) | 1827 (1625,2072) | 3947 (2756,5400) | 397 (216,606) | 1800 (1603,2039) | 1691 (1524,1891) | 3495 (3051,4038) | 1923 (1750,2105) |
| 24-59 | 1755 (1484,2053) | 2631 (2289,3019) | 1958 (1734,2200) | 4261 (3001,5741) | 423 (233,653) | 1927 (1696,2157) | 1805 (1628,1999) | 3743 (3261,4294) | 2049 (1872,2250) |
| <60 | 1739 (1466,2026) | 2586 (2258,2920) | 1930 (1699,2156) | 4182 (2778,5656) | 410 (238,649) | 1894 (1686,2144) | 1766 (1603,1966) | 3692 (3186,4199) | 2004 (1832,2211) |
| 2013 |  |  |  |  |  |  |  |  |  |
| <1 | 427 (360,502) | 636 (558,737) | 473 (421,532) | 1036 (711,1379) | 100 (56,158) | 466 (410,523) | 435 (394,483) | 907 (784,1045) | 494 (450,547) |
| 1 | 483 (410,564) | 721 (632,819) | 533 (475,601) | 1175 (786,1573) | 112 (64,177) | 523 (463,593) | 491 (446,546) | 1024 (883,1179) | 559 (510,619) |
| 2 | 1242 (1053,1460) | 1863 (1617,2139) | 1383 (1235,1570) | 3032 (2030,4017) | 299 (164,456) | 1363 (1199,1536) | 1277 (1155,1421) | 2659 (2276,3080) | 1449 (1323,1596) |
| 3 | 1872 (1575,2204) | 2786 (2413,3172) | 2071 (1835,2343) | 4536 (3080,6081) | 441 (242,683) | 2042 (1816,2300) | 1910 (1713,2138) | 3986 (3428,4606) | 2169 (1993,2382) |
| 4 | 2306 (1956,2743) | 3482 (3018,3974) | 2581 (2291,2912) | 5668 (3835,7693) | 541 (301,842) | 2533 (2241,2857) | 2369 (2149,2637) | 4923 (4268,5675) | 2695 (2467,2958) |
| 5 | 2451 (2043,2875) | 3670 (3216,4163) | 2723 (2420,3057) | 5951 (4166,7902) | 579 (330,905) | 2680 (2360,3017) | 2510 (2263,2771) | 5212 (4541,6011) | 2851 (2603,3157) |
| 6 | 2363 (1990,2731) | 3559 (3119,4051) | 2635 (2337,2959) | 5765 (3975,7783) | 567 (307,880) | 2581 (2292,2919) | 2418 (2197,2693) | 5042 (4365,5780) | 2759 (2523,3014) |
| 7 | 2445 (2064,2880) | 3695 (3196,4194) | 2739 (2425,3087) | 5978 (3852,8164) | 582 (325,887) | 2679 (2391,3037) | 2511 (2272,2780) | 5241 (4516,5964) | 2859 (2598,3141) |
| 8 | 2224 (1881,2599) | 3329 (2901,3799) | 2472 (2191,2786) | 5380 (3662,7362) | 528 (271,826) | 2436 (2150,2750) | 2280 (2048,2536) | 4722 (4103,5483) | 2578 (2363,2847) |
| 9 | 2278 (1940,2645) | 3411 (2973,3875) | 2539 (2260,2823) | 5593 (3830,7526) | 541 (298,815) | 2488 (2213,2823) | 2337 (2112,2620) | 4875 (4204,5622) | 2650 (2431,2923) |
| 10 | 1895 (1605,2218) | 2837 (2492,3264) | 2105 (1862,2371) | 4607 (3202,6314) | 452 (251,712) | 2071 (1827,2346) | 1939 (1755,2137) | 4022 (3483,4627) | 2195 (2014,2425) |
| 11 | 1678 (1428,1960) | 2526 (2205,2862) | 1877 (1678,2122) | 4110 (2825,5511) | 398 (228,613) | 1848 (1629,2078) | 1729 (1551,1912) | 3557 (3127,4123) | 1950 (1795,2151) |
| <12 | 1748 (1468,2068) | 2623 (2287,3002) | 1945 (1719,2172) | 4258 (2940,5761) | 421 (243,653) | 1921 (1705,2165) | 1800 (1633,1999) | 3728 (3240,4273) | 2042 (1868,2225) |
| 12-14 | 1660 (1394,1979) | 2497 (2184,2853) | 1856 (1654,2092) | 4061 (2682,5436) | 394 (217,623) | 1822 (1618,2072) | 1711 (1542,1901) | 3561 (3095,4107) | 1934 (1778,2131) |
| 15-17 | 2271 (1913,2679) | 3381 (2970,3896) | 2511 (2236,2831) | 5512 (3887,7252) | 533 (288,829) | 2464 (2190,2788) | 2320 (2086,2568) | 4827 (4172,5532) | 2627 (2397,2886) |
| 18-20 | 1902 (1594,2251) | 2834 (2502,3246) | 2113 (1875,2388) | 4618 (3058,6238) | 442 (249,693) | 2076 (1834,2347) | 1950 (1767,2154) | 4031 (3480,4665) | 2208 (2029,2417) |
| 21-23 | 1826 (1551,2114) | 2734 (2365,3106) | 2024 (1796,2274) | 4460 (3052,5844) | 420 (246,687) | 1997 (1765,2257) | 1870 (1689,2070) | 3879 (3369,4449) | 2118 (1932,2337) |
| 12-23 | 1876 (1596,2176) | 2812 (2465,3230) | 2087 (1845,2356) | 4578 (3154,6028) | 439 (242,670) | 2050 (1823,2298) | 1929 (1733,2130) | 3995 (3475,4609) | 2181 (2004,2408) |
| <24 | 1803 (1525,2123) | 2700 (2372,3089) | 2007 (1774,2252) | 4390 (2989,5894) | 429 (237,687) | 1979 (1753,2212) | 1845 (1669,2065) | 3838 (3337,4420) | 2100 (1912,2317) |
| 24-35 | 1895 (1593,2222) | 2842 (2505,3238) | 2112 (1858,2361) | 4659 (3188,6106) | 450 (248,691) | 2065 (1820,2319) | 1938 (1756,2153) | 4021 (3513,4631) | 2200 (2022,2414) |
| 36-47 | 1714 (1458,2017) | 2579 (2249,2962) | 1907 (1689,2150) | 4149 (2891,5510) | 407 (231,616) | 1875 (1653,2120) | 1759 (1580,1953) | 3656 (3188,4185) | 1999 (1823,2188) |
| 48-59 | 1971 (1650,2296) | 2949 (2607,3336) | 2196 (1945,2482) | 4789 (3346,6501) | 459 (264,708) | 2157 (1895,2445) | 2011 (1821,2228) | 4205 (3592,4854) | 2288 (2077,2522) |
| 24-59 | 1846 (1543,2139) | 2751 (2409,3148) | 2036 (1812,2290) | 4435 (3104,5905) | 442 (243,670) | 2013 (1781,2270) | 1888 (1696,2090) | 3933 (3354,4472) | 2141 (1940,2357) |
| <60 | 1756 (1480,2043) | 2622 (2311,2966) | 1937 (1716,2186) | 4214 (2865,5684) | 412 (232,633) | 1907 (1689,2165) | 1789 (1602,1989) | 3715 (3210,4275) | 2026 (1850,2230) |
| 2014 |  |  |  |  |  |  |  |  |  |
| <1 | 171 (146,200) | 258 (227,293) | 192 (171,217) | 418 (280,562) | 41 (24,63) | 189 (169,214) | 177 (158,197) | 369 (317,425) | 201 (184,221) |
| 1 | 526 (437,617) | 787 (693,893) | 584 (518,658) | 1263 (883,1726) | 124 (69,191) | 573 (508,644) | 538 (486,598) | 1111 (960,1279) | 609 (557,671) |
| 2 | 1242 (1057,1468) | 1875 (1644,2138) | 1386 (1233,1569) | 3049 (2135,4046) | 294 (168,459) | 1367 (1195,1533) | 1277 (1143,1431) | 2653 (2309,3051) | 1450 (1328,1584) |
| 3 | 1244 (1048,1460) | 1850 (1623,2113) | 1384 (1219,1551) | 3029 (2128,4040) | 296 (163,457) | 1356 (1199,1528) | 1271 (1142,1402) | 2638 (2291,3009) | 1446 (1315,1587) |
| 4 | 1617 (1369,1881) | 2416 (2131,2756) | 1794 (1589,2018) | 3911 (2600,5274) | 378 (214,588) | 1756 (1558,1984) | 1648 (1488,1839) | 3419 (2950,3898) | 1871 (1713,2048) |
| 5 | 1674 (1402,1924) | 2493 (2172,2837) | 1841 (1618,2075) | 4033 (2781,5404) | 391 (217,610) | 1815 (1628,2076) | 1700 (1542,1896) | 3546 (3063,4073) | 1931 (1775,2128) |
| 6 | 2235 (1886,2626) | 3342 (2916,3814) | 2491 (2213,2840) | 5441 (3595,7256) | 525 (285,846) | 2435 (2154,2751) | 2289 (2080,2545) | 4726 (4110,5486) | 2587 (2373,2847) |
| 7 | 2586 (2181,3002) | 3833 (3358,4339) | 2857 (2550,3256) | 6250 (4149,8470) | 616 (355,928) | 2803 (2469,3148) | 2643 (2382,2916) | 5473 (4783,6243) | 2981 (2727,3276) |
| 8 | 3208 (2731,3778) | 4813 (4194,5472) | 3572 (3160,4035) | 7760 (5197,10355) | 756 (426,1179) | 3498 (3091,3964) | 3292 (2976,3676) | 6818 (5911,7873) | 3726 (3407,4105) |
| 9 | 2070 (1750,2403) | 3096 (2703,3509) | 2300 (2033,2583) | 5024 (3342,6781) | 497 (277,750) | 2258 (1997,2555) | 2133 (1921,2374) | 4429 (3807,5012) | 2411 (2190,2645) |
| 10 | 2114 (1782,2468) | 3171 (2764,3644) | 2358 (2096,2650) | 5126 (3340,6948) | 501 (265,771) | 2318 (2048,2622) | 2185 (1956,2436) | 4545 (3914,5235) | 2468 (2262,2736) |
| 11 | 1856 (1582,2147) | 2781 (2412,3159) | 2054 (1827,2323) | 4541 (3126,6180) | 429 (248,677) | 2020 (1788,2285) | 1897 (1713,2115) | 3947 (3366,4488) | 2152 (1978,2370) |
| <12 | 1688 (1432,1968) | 2510 (2207,2858) | 1865 (1662,2094) | 4105 (2779,5500) | 398 (228,625) | 1835 (1628,2083) | 1714 (1541,1916) | 3571 (3078,4124) | 1954 (1782,2147) |
| 12-14 | 1524 (1289,1788) | 2297 (2013,2602) | 1700 (1517,1902) | 3716 (2503,4996) | 354 (193,560) | 1673 (1469,1894) | 1573 (1427,1747) | 3272 (2832,3728) | 1782 (1634,1964) |
| 15-17 | 2228 (1921,2612) | 3360 (2946,3811) | 2510 (2209,2809) | 5444 (3840,7354) | 532 (273,830) | 2453 (2178,2770) | 2301 (2060,2557) | 4764 (4125,5476) | 2611 (2374,2879) |
| 18-20 | 1817 (1533,2100) | 2722 (2373,3126) | 2026 (1799,2280) | 4399 (3063,5962) | 432 (243,660) | 1992 (1768,2248) | 1866 (1688,2082) | 3886 (3358,4454) | 2124 (1948,2338) |
| 21-23 | 1944 (1639,2265) | 2914 (2573,3328) | 2155 (1932,2454) | 4727 (3228,6219) | 458 (252,705) | 2116 (1876,2406) | 1989 (1788,2209) | 4147 (3551,4742) | 2258 (2064,2479) |
| 12-23 | 1848 (1556,2148) | 2763 (2424,3128) | 2055 (1818,2306) | 4521 (3079,6053) | 437 (240,687) | 2001 (1790,2267) | 1885 (1711,2118) | 3917 (3412,4537) | 2150 (1967,2361) |
| <24 | 1746 (1478,2042) | 2631 (2309,2965) | 1960 (1728,2194) | 4283 (2855,5751) | 407 (229,624) | 1913 (1690,2163) | 1793 (1625,1980) | 3733 (3196,4233) | 2036 (1870,2234) |
| 24-35 | 1926 (1606,2243) | 2857 (2510,3279) | 2128 (1885,2392) | 4642 (3192,6361) | 458 (260,724) | 2089 (1865,2380) | 1958 (1776,2184) | 4067 (3535,4721) | 2235 (2037,2460) |
| 36-47 | 1967 (1648,2272) | 2941 (2574,3343) | 2195 (1954,2456) | 4736 (3388,6504) | 458 (260,695) | 2150 (1918,2434) | 2010 (1811,2246) | 4179 (3593,4809) | 2288 (2092,2538) |
| 48-59 | 1862 (1561,2173) | 2803 (2444,3197) | 2066 (1838,2322) | 4488 (3068,6148) | 445 (234,672) | 2029 (1818,2287) | 1912 (1724,2124) | 3969 (3443,4507) | 2168 (1980,2398) |
| 24-59 | 1907 (1622,2250) | 2847 (2494,3251) | 2119 (1873,2378) | 4571 (3260,6206) | 448 (252,686) | 2088 (1833,2377) | 1948 (1753,2167) | 4063 (3502,4657) | 2213 (2025,2440) |
| <60 | 1752 (1498,2048) | 2617 (2304,2978) | 1946 (1731,2197) | 4287 (2815,5756) | 407 (226,656) | 1912 (1696,2165) | 1788 (1616,1983) | 3726 (3209,4279) | 2031 (1859,2231) |
| 2015 |  |  |  |  |  |  |  |  |  |
| <1 | 251 (215,295) | 378 (332,434) | 280 (249,317) | 617 (423,842) | 61 (34,92) | 276 (242,310) | 258 (232,287) | 539 (464,617) | 293 (270,324) |
| 1 | 394 (334,467) | 588 (514,667) | 439 (389,498) | 960 (646,1283) | 94 (53,144) | 431 (381,485) | 405 (365,450) | 841 (735,963) | 459 (419,506) |
| 2 | 753 (639,888) | 1137 (998,1302) | 843 (744,951) | 1834 (1266,2478) | 180 (102,276) | 825 (732,938) | 773 (700,854) | 1610 (1391,1850) | 880 (807,977) |
| 3 | 1214 (1016,1423) | 1820 (1577,2072) | 1349 (1197,1512) | 2936 (2001,4016) | 288 (163,445) | 1326 (1175,1499) | 1243 (1114,1376) | 2585 (2242,2979) | 1410 (1291,1555) |
| 4 | 1478 (1263,1743) | 2196 (1940,2515) | 1633 (1452,1861) | 3608 (2579,4953) | 341 (193,538) | 1606 (1425,1798) | 1510 (1362,1691) | 3143 (2728,3560) | 1710 (1554,1890) |
| 5 | 1017 (866,1191) | 1532 (1350,1754) | 1142 (1012,1284) | 2490 (1698,3307) | 245 (134,385) | 1115 (990,1249) | 1048 (949,1169) | 2173 (1886,2510) | 1186 (1083,1321) |
| 6 | 1998 (1682,2353) | 3010 (2629,3434) | 2230 (1973,2521) | 4892 (3440,6637) | 472 (266,742) | 2181 (1951,2481) | 2052 (1867,2271) | 4281 (3712,4959) | 2325 (2121,2563) |
| 7 | 2173 (1847,2528) | 3261 (2841,3715) | 2413 (2136,2738) | 5294 (3555,7106) | 513 (278,794) | 2374 (2098,2670) | 2230 (2015,2458) | 4642 (4013,5335) | 2526 (2319,2785) |
| 8 | 1676 (1434,1977) | 2535 (2216,2905) | 1883 (1673,2102) | 4148 (2694,5504) | 402 (232,620) | 1857 (1631,2093) | 1733 (1562,1921) | 3583 (3119,4144) | 1968 (1802,2173) |
| 9 | 873 (746,1018) | 1308 (1143,1489) | 971 (858,1085) | 2137 (1410,2869) | 208 (118,332) | 953 (839,1064) | 890 (804,989) | 1852 (1609,2132) | 1013 (927,1119) |
| 10 | 1256 (1068,1454) | 1891 (1651,2158) | 1405 (1245,1600) | 3040 (2067,4093) | 300 (161,462) | 1375 (1214,1545) | 1289 (1159,1426) | 2683 (2329,3076) | 1469 (1341,1615) |
| 11 | 1142 (962,1333) | 1705 (1495,1937) | 1267 (1132,1430) | 2784 (1955,3719) | 268 (156,408) | 1249 (1100,1405) | 1171 (1059,1302) | 2437 (2116,2781) | 1326 (1212,1463) |
| <12 | 1142 (959,1331) | 1719 (1499,1955) | 1269 (1133,1425) | 2785 (1942,3756) | 270 (148,418) | 1251 (1110,1411) | 1171 (1059,1296) | 2446 (2088,2822) | 1330 (1219,1449) |
| 12-14 | 1155 (971,1351) | 1728 (1504,1948) | 1284 (1142,1446) | 2824 (1916,3759) | 274 (161,418) | 1270 (1113,1427) | 1186 (1059,1324) | 2467 (2124,2810) | 1345 (1238,1479) |
| 15-17 | 957 (811,1118) | 1433 (1259,1628) | 1058 (941,1200) | 2322 (1635,3097) | 231 (130,350) | 1045 (924,1180) | 976 (878,1082) | 2028 (1756,2338) | 1107 (1014,1230) |
| 18-20 | 1058 (899,1241) | 1594 (1376,1826) | 1179 (1046,1324) | 2571 (1742,3424) | 246 (149,382) | 1159 (1020,1295) | 1081 (976,1192) | 2259 (1974,2596) | 1232 (1135,1355) |
| 21-23 | 1061 (877,1239) | 1581 (1379,1788) | 1178 (1042,1327) | 2572 (1763,3517) | 252 (146,395) | 1151 (1008,1305) | 1084 (975,1209) | 2256 (1953,2600) | 1225 (1128,1354) |
| 12-23 | 1047 (890,1236) | 1564 (1357,1803) | 1161 (1037,1309) | 2534 (1807,3456) | 247 (142,378) | 1139 (1016,1297) | 1072 (972,1186) | 2235 (1925,2551) | 1218 (1115,1342) |
| <24 | 1081 (918,1275) | 1636 (1422,1864) | 1216 (1078,1377) | 2666 (1771,3532) | 256 (143,399) | 1195 (1053,1348) | 1115 (1010,1245) | 2323 (2010,2658) | 1268 (1165,1407) |
| 24-35 | 1276 (1094,1502) | 1926 (1685,2190) | 1430 (1272,1629) | 3113 (2180,4130) | 309 (163,473) | 1408 (1232,1593) | 1317 (1190,1469) | 2744 (2394,3131) | 1499 (1369,1647) |
| 36-47 | 1179 (1002,1386) | 1774 (1567,2014) | 1316 (1175,1480) | 2896 (1977,3813) | 280 (147,435) | 1294 (1145,1459) | 1210 (1093,1351) | 2524 (2180,2885) | 1375 (1262,1526) |
| 48-59 | 1110 (943,1311) | 1665 (1460,1904) | 1235 (1101,1397) | 2707 (1871,3631) | 263 (142,407) | 1221 (1077,1373) | 1141 (1025,1261) | 2376 (2050,2694) | 1297 (1191,1420) |
| 24-59 | 1191 (1008,1404) | 1784 (1550,2041) | 1325 (1175,1491) | 2911 (1921,3810) | 276 (149,438) | 1306 (1152,1477) | 1215 (1100,1350) | 2535 (2217,2907) | 1389 (1271,1518) |
| <60 | 1090 (909,1257) | 1629 (1429,1861) | 1214 (1078,1370) | 2676 (1846,3567) | 261 (141,399) | 1194 (1053,1344) | 1119 (1000,1240) | 2318 (1993,2678) | 1268 (1162,1391) |
| 2016 |  |  |  |  |  |  |  |  |  |
| <1 | 141 (118,165) | 211 (185,241) | 156 (139,176) | 342 (233,461) | 33 (19,54) | 155 (136,173) | 145 (130,160) | 301 (262,343) | 164 (150,180) |
| 1 | 279 (235,325) | 417 (370,471) | 308 (273,348) | 685 (464,908) | 66 (37,100) | 303 (267,340) | 285 (259,317) | 592 (512,678) | 322 (295,352) |
| 2 | 1139 (971,1343) | 1703 (1508,1957) | 1264 (1125,1423) | 2767 (1901,3742) | 271 (158,418) | 1243 (1108,1387) | 1167 (1051,1302) | 2423 (2101,2788) | 1326 (1211,1466) |
| 3 | 894 (756,1036) | 1343 (1177,1536) | 999 (889,1126) | 2200 (1498,2998) | 210 (117,319) | 978 (870,1115) | 917 (828,1018) | 1908 (1651,2211) | 1046 (957,1160) |
| 4 | 1213 (1029,1444) | 1822 (1602,2089) | 1354 (1192,1519) | 2944 (2042,3961) | 289 (162,444) | 1335 (1181,1499) | 1249 (1130,1390) | 2614 (2277,3009) | 1415 (1301,1566) |
| 5 | 1459 (1227,1715) | 2194 (1930,2497) | 1627 (1442,1827) | 3520 (2431,4745) | 345 (193,550) | 1602 (1397,1797) | 1498 (1359,1665) | 3142 (2698,3585) | 1700 (1567,1877) |
| 6 | 2102 (1783,2438) | 3157 (2742,3589) | 2338 (2086,2631) | 5117 (3670,6818) | 492 (273,764) | 2301 (2042,2609) | 2161 (1947,2390) | 4492 (3875,5171) | 2444 (2235,2717) |
| 7 | 1814 (1531,2116) | 2700 (2351,3070) | 1998 (1773,2244) | 4409 (3021,6003) | 433 (242,663) | 1976 (1755,2222) | 1851 (1666,2045) | 3849 (3348,4400) | 2101 (1923,2323) |
| 8 | 1291 (1092,1510) | 1927 (1700,2184) | 1440 (1279,1613) | 3129 (2153,4138) | 305 (174,476) | 1408 (1252,1589) | 1320 (1198,1457) | 2756 (2363,3185) | 1502 (1372,1659) |
| 9 | 1603 (1358,1863) | 2413 (2117,2774) | 1787 (1596,2024) | 3889 (2670,5263) | 378 (213,578) | 1769 (1555,1987) | 1646 (1487,1838) | 3418 (2964,3939) | 1875 (1721,2076) |
| 10 | 1914 (1624,2237) | 2873 (2515,3266) | 2135 (1887,2380) | 4634 (3153,6323) | 450 (242,710) | 2092 (1856,2345) | 1961 (1769,2179) | 4099 (3477,4698) | 2231 (2037,2465) |
| 11 | 1737 (1472,2018) | 2618 (2318,2990) | 1937 (1713,2185) | 4229 (2814,5730) | 413 (220,649) | 1907 (1694,2157) | 1791 (1615,2000) | 3711 (3201,4236) | 2040 (1851,2250) |
| <12 | 1238 (1031,1467) | 1856 (1624,2124) | 1386 (1226,1552) | 3026 (2094,4009) | 297 (162,454) | 1361 (1195,1524) | 1272 (1147,1422) | 2649 (2250,3070) | 1444 (1320,1594) |
| 12-14 | 1430 (1214,1667) | 2134 (1869,2423) | 1579 (1396,1782) | 3472 (2277,4650) | 337 (192,523) | 1555 (1395,1765) | 1458 (1315,1613) | 3018 (2644,3464) | 1658 (1507,1822) |
| 15-17 | 1366 (1162,1600) | 2043 (1783,2341) | 1517 (1344,1716) | 3310 (2251,4473) | 321 (177,493) | 1494 (1318,1687) | 1399 (1267,1561) | 2907 (2508,3402) | 1584 (1453,1742) |
| 18-20 | 1651 (1387,1923) | 2457 (2164,2818) | 1828 (1628,2068) | 3984 (2728,5385) | 393 (205,586) | 1796 (1595,2027) | 1680 (1529,1874) | 3509 (3076,4051) | 1913 (1753,2117) |
| 21-23 | 1108 (935,1311) | 1669 (1458,1905) | 1237 (1089,1384) | 2710 (1833,3647) | 265 (154,421) | 1217 (1083,1379) | 1136 (1039,1277) | 2370 (2023,2715) | 1291 (1177,1417) |
| 12-23 | 1373 (1141,1585) | 2060 (1797,2339) | 1530 (1360,1710) | 3358 (2262,4466) | 323 (178,503) | 1497 (1332,1702) | 1404 (1271,1564) | 2943 (2532,3359) | 1593 (1447,1736) |
| <24 | 1300 (1089,1529) | 1949 (1705,2238) | 1444 (1280,1618) | 3191 (2081,4307) | 311 (172,473) | 1413 (1263,1593) | 1336 (1198,1493) | 2766 (2408,3202) | 1509 (1386,1666) |
| 24-35 | 1318 (1110,1536) | 1964 (1720,2240) | 1460 (1287,1648) | 3217 (2130,4337) | 311 (177,484) | 1437 (1281,1623) | 1350 (1220,1523) | 2808 (2440,3230) | 1534 (1398,1682) |
| 36-47 | 1551 (1299,1794) | 2322 (2016,2641) | 1726 (1543,1933) | 3760 (2531,5018) | 362 (210,577) | 1698 (1511,1902) | 1585 (1433,1759) | 3299 (2877,3792) | 1799 (1644,1977) |
| 48-59 | 1303 (1092,1530) | 1957 (1716,2213) | 1454 (1288,1637) | 3147 (2195,4247) | 309 (173,468) | 1425 (1261,1599) | 1344 (1210,1482) | 2772 (2399,3175) | 1517 (1382,1671) |
| 24-59 | 1368 (1154,1600) | 2059 (1808,2368) | 1528 (1358,1714) | 3332 (2236,4478) | 325 (180,504) | 1503 (1331,1674) | 1406 (1271,1561) | 2923 (2549,3348) | 1599 (1465,1753) |
| <60 | 1276 (1077,1510) | 1923 (1671,2178) | 1423 (1273,1597) | 3107 (2150,4283) | 301 (160,456) | 1403 (1231,1577) | 1313 (1188,1463) | 2727 (2356,3148) | 1494 (1345,1624) |
| 2017 |  |  |  |  |  |  |  |  |  |
| <1 | 350 (292,415) | 525 (456,596) | 390 (349,440) | 851 (582,1153) | 82 (48,131) | 381 (336,431) | 359 (323,399) | 746 (647,851) | 406 (372,445) |
| 1 | 493 (416,569) | 739 (644,844) | 550 (483,617) | 1202 (812,1603) | 116 (66,177) | 539 (479,609) | 505 (457,562) | 1056 (911,1209) | 572 (523,627) |
| 2 | 1007 (845,1174) | 1509 (1306,1726) | 1119 (989,1264) | 2461 (1660,3267) | 240 (133,370) | 1099 (984,1256) | 1032 (922,1151) | 2138 (1838,2448) | 1171 (1072,1293) |
| 3 | 1615 (1345,1890) | 2406 (2107,2738) | 1798 (1591,2013) | 3882 (2674,5179) | 389 (223,588) | 1760 (1546,1977) | 1646 (1485,1829) | 3430 (2946,3952) | 1872 (1703,2072) |
| 4 | 2007 (1699,2360) | 2994 (2607,3456) | 2223 (1971,2519) | 4873 (3347,6555) | 475 (276,727) | 2191 (1930,2481) | 2058 (1851,2283) | 4257 (3695,4876) | 2328 (2131,2579) |
| 5 | 1988 (1678,2339) | 2988 (2595,3414) | 2219 (1975,2488) | 4828 (3245,6532) | 468 (266,726) | 2169 (1936,2451) | 2033 (1831,2269) | 4229 (3658,4911) | 2316 (2130,2555) |
| 6 | 1741 (1470,2038) | 2597 (2272,2958) | 1923 (1729,2170) | 4199 (2918,5639) | 409 (219,622) | 1896 (1685,2138) | 1774 (1609,1968) | 3690 (3165,4254) | 2013 (1836,2212) |
| 7 | 1708 (1444,2002) | 2558 (2215,2945) | 1886 (1679,2130) | 4142 (2687,5629) | 397 (224,606) | 1854 (1652,2116) | 1750 (1564,1946) | 3628 (3127,4192) | 1976 (1803,2177) |
| 8 | 2244 (1902,2646) | 3349 (2937,3781) | 2490 (2223,2834) | 5453 (3686,7294) | 535 (289,829) | 2450 (2166,2782) | 2295 (2083,2550) | 4790 (4139,5551) | 2606 (2381,2871) |
| 9 | 1483 (1241,1731) | 2223 (1935,2548) | 1642 (1458,1839) | 3575 (2443,4721) | 349 (206,547) | 1621 (1426,1838) | 1522 (1365,1689) | 3141 (2733,3648) | 1722 (1578,1905) |
| 10 | 1361 (1145,1574) | 2034 (1786,2300) | 1503 (1341,1699) | 3314 (2291,4435) | 319 (181,494) | 1476 (1309,1666) | 1387 (1243,1543) | 2877 (2513,3289) | 1577 (1423,1722) |
| 11 | 2007 (1682,2306) | 2994 (2623,3404) | 2225 (1991,2496) | 4850 (3257,6440) | 474 (266,724) | 2192 (1940,2475) | 2058 (1845,2289) | 4280 (3726,4894) | 2334 (2140,2558) |
| <12 | 1478 (1240,1721) | 2210 (1923,2530) | 1645 (1449,1855) | 3586 (2472,4756) | 345 (194,530) | 1606 (1424,1814) | 1515 (1364,1673) | 3158 (2722,3635) | 1715 (1575,1896) |
| 12-14 | 1405 (1177,1643) | 2109 (1820,2394) | 1562 (1401,1760) | 3404 (2329,4513) | 338 (187,518) | 1543 (1366,1744) | 1443 (1311,1603) | 2983 (2605,3467) | 1636 (1490,1790) |
| 15-17 | 1421 (1214,1662) | 2130 (1871,2460) | 1591 (1403,1804) | 3479 (2355,4681) | 331 (182,521) | 1560 (1377,1754) | 1459 (1331,1624) | 3043 (2646,3535) | 1669 (1521,1838) |
| 18-20 | 1715 (1438,2020) | 2595 (2253,2965) | 1922 (1703,2148) | 4207 (2779,5535) | 413 (228,630) | 1884 (1688,2116) | 1761 (1597,1965) | 3682 (3183,4235) | 2016 (1844,2228) |
| 21-23 | 1314 (1101,1530) | 1966 (1729,2239) | 1460 (1304,1637) | 3175 (2130,4276) | 315 (171,478) | 1437 (1272,1644) | 1345 (1216,1499) | 2817 (2431,3247) | 1528 (1396,1690) |
| 12-23 | 1467 (1235,1722) | 2189 (1919,2474) | 1625 (1442,1827) | 3559 (2383,4852) | 344 (186,550) | 1599 (1412,1808) | 1500 (1354,1665) | 3106 (2703,3580) | 1697 (1550,1881) |
| <24 | 1460 (1252,1736) | 2197 (1908,2509) | 1626 (1441,1837) | 3536 (2396,4756) | 349 (191,537) | 1597 (1419,1807) | 1499 (1345,1660) | 3123 (2690,3588) | 1701 (1551,1876) |
| 24-35 | 1583 (1338,1857) | 2382 (2071,2731) | 1764 (1563,1984) | 3840 (2636,5191) | 369 (210,577) | 1748 (1539,1971) | 1622 (1459,1810) | 3377 (2951,3843) | 1848 (1686,2019) |
| 36-47 | 1390 (1175,1619) | 2068 (1805,2368) | 1536 (1368,1726) | 3401 (2287,4438) | 326 (178,499) | 1517 (1336,1694) | 1416 (1271,1567) | 2944 (2535,3372) | 1602 (1464,1766) |
| 48-59 | 1327 (1120,1557) | 1980 (1739,2272) | 1473 (1316,1662) | 3221 (2135,4280) | 312 (168,469) | 1452 (1276,1628) | 1355 (1226,1517) | 2812 (2455,3253) | 1542 (1401,1702) |
| 24-59 | 1428 (1221,1659) | 2136 (1853,2413) | 1587 (1397,1797) | 3511 (2376,4721) | 341 (186,517) | 1554 (1375,1787) | 1459 (1325,1614) | 3032 (2635,3483) | 1654 (1507,1827) |
| <60 | 1378 (1154,1627) | 2060 (1814,2334) | 1538 (1365,1729) | 3419 (2307,4526) | 330 (184,511) | 1511 (1324,1718) | 1416 (1282,1571) | 2948 (2545,3395) | 1609 (1474,1767) |
| 2018 |  |  |  |  |  |  |  |  |  |
| <1 | 339 (288,394) | 514 (449,577) | 380 (338,424) | 829 (548,1120) | 81 (47,125) | 374 (329,422) | 349 (313,392) | 727 (623,832) | 397 (363,437) |
| 1 | 580 (492,681) | 869 (758,997) | 646 (577,721) | 1407 (935,1894) | 139 (81,211) | 635 (558,712) | 596 (536,662) | 1235 (1076,1415) | 676 (619,744) |
| 2 | 1034 (871,1214) | 1557 (1354,1781) | 1154 (1023,1307) | 2510 (1661,3498) | 248 (133,381) | 1134 (998,1283) | 1064 (961,1178) | 2211 (1900,2540) | 1208 (1112,1326) |
| 3 | 1476 (1247,1730) | 2202 (1910,2510) | 1630 (1444,1832) | 3546 (2486,4874) | 342 (185,538) | 1600 (1422,1808) | 1503 (1358,1668) | 3118 (2678,3588) | 1699 (1557,1871) |
| 4 | 1454 (1235,1688) | 2190 (1927,2483) | 1616 (1442,1821) | 3528 (2451,4879) | 348 (198,537) | 1596 (1415,1791) | 1494 (1352,1661) | 3094 (2679,3572) | 1697 (1549,1863) |
| 5 | 2177 (1875,2538) | 3274 (2870,3695) | 2435 (2136,2711) | 5314 (3725,7100) | 515 (284,775) | 2391 (2118,2717) | 2239 (2004,2470) | 4660 (4039,5365) | 2537 (2320,2792) |
| 6 | 2197 (1837,2552) | 3269 (2859,3777) | 2420 (2144,2715) | 5292 (3621,7157) | 519 (289,816) | 2385 (2120,2698) | 2236 (2022,2491) | 4659 (4066,5327) | 2539 (2315,2804) |
| 7 | 2563 (2137,2992) | 3857 (3374,4436) | 2859 (2525,3232) | 6273 (4406,8589) | 608 (338,952) | 2802 (2496,3176) | 2626 (2372,2936) | 5474 (4744,6299) | 2990 (2719,3300) |
| 8 | 2350 (1978,2764) | 3528 (3095,4002) | 2624 (2327,2931) | 5788 (4042,7874) | 555 (320,875) | 2578 (2294,2909) | 2408 (2174,2710) | 5038 (4344,5741) | 2729 (2501,3012) |
| 9 | 1855 (1549,2179) | 2772 (2409,3172) | 2052 (1805,2319) | 4504 (3062,6021) | 434 (246,667) | 2021 (1785,2279) | 1890 (1721,2094) | 3926 (3381,4554) | 2154 (1959,2360) |
| 10 | 2066 (1735,2439) | 3120 (2726,3550) | 2310 (2056,2609) | 5073 (3393,6775) | 491 (280,764) | 2268 (2017,2553) | 2123 (1921,2353) | 4384 (3818,5038) | 2415 (2212,2659) |
| 11 | 1689 (1436,1969) | 2512 (2188,2863) | 1877 (1658,2125) | 4076 (2710,5423) | 399 (229,639) | 1843 (1631,2067) | 1723 (1559,1928) | 3588 (3098,4110) | 1958 (1794,2151) |
| <12 | 1638 (1380,1909) | 2447 (2144,2795) | 1822 (1618,2051) | 3961 (2772,5309) | 385 (214,593) | 1793 (1581,2015) | 1682 (1516,1877) | 3487 (3033,4009) | 1907 (1742,2100) |
| 12-14 | 1644 (1367,1927) | 2452 (2126,2792) | 1827 (1611,2072) | 4001 (2590,5371) | 382 (215,603) | 1794 (1597,2017) | 1677 (1522,1866) | 3501 (3034,4009) | 1906 (1742,2094) |
| 15-17 | 1821 (1546,2125) | 2716 (2392,3098) | 2016 (1795,2311) | 4441 (2994,5823) | 430 (240,675) | 2001 (1779,2257) | 1866 (1687,2076) | 3889 (3358,4451) | 2120 (1937,2322) |
| 18-20 | 1483 (1245,1749) | 2210 (1936,2520) | 1650 (1468,1864) | 3578 (2527,4841) | 347 (202,532) | 1625 (1446,1829) | 1516 (1379,1690) | 3151 (2710,3636) | 1727 (1583,1890) |
| 21-23 | 1467 (1224,1719) | 2188 (1906,2508) | 1626 (1433,1823) | 3539 (2385,4690) | 349 (190,549) | 1598 (1420,1774) | 1496 (1355,1657) | 3099 (2676,3591) | 1698 (1553,1871) |
| 12-23 | 1577 (1323,1857) | 2354 (2059,2686) | 1753 (1564,1979) | 3827 (2660,5167) | 379 (221,571) | 1719 (1523,1951) | 1615 (1456,1791) | 3356 (2898,3868) | 1826 (1679,2010) |
| <24 | 1596 (1349,1872) | 2398 (2080,2779) | 1779 (1572,2001) | 3870 (2652,5241) | 378 (203,575) | 1752 (1560,1967) | 1640 (1474,1833) | 3425 (2948,3954) | 1853 (1694,2047) |
| 24-35 | 1647 (1385,1920) | 2456 (2173,2801) | 1820 (1628,2050) | 3974 (2712,5394) | 392 (215,598) | 1798 (1585,2001) | 1683 (1515,1861) | 3517 (3039,4017) | 1908 (1751,2093) |
| 36-47 | 1554 (1297,1817) | 2315 (2017,2643) | 1714 (1518,1959) | 3801 (2626,5027) | 368 (200,570) | 1687 (1499,1888) | 1578 (1429,1765) | 3298 (2848,3804) | 1804 (1641,1978) |
| 48-59 | 1457 (1230,1682) | 2180 (1896,2480) | 1619 (1446,1835) | 3514 (2453,4779) | 350 (196,523) | 1593 (1421,1790) | 1491 (1342,1652) | 3088 (2664,3556) | 1692 (1550,1858) |
| 24-59 | 1541 (1326,1803) | 2316 (2036,2646) | 1719 (1531,1951) | 3739 (2544,5033) | 363 (211,566) | 1689 (1493,1893) | 1584 (1431,1747) | 3291 (2840,3799) | 1799 (1643,1983) |
| <60 | 1491 (1252,1744) | 2245 (1954,2563) | 1670 (1469,1880) | 3603 (2451,4944) | 355 (207,539) | 1627 (1448,1847) | 1527 (1381,1706) | 3189 (2763,3644) | 1739 (1597,1914) |

**Table S5: Annual rates of RSV associated non-medically attended acute respiratory illness per 1,000 children by regions and age in months, 2010-2018**

| Year and Age in months | Central Rate (95% CI) | Coast Rate (95% CI) | Eastern Rate (95% CI) | North Eastern Rate (95% CI) | Nairobi Rate (95% CI) | Nyanza Rate (95% CI) | Rift Valley Rate (95% CI) | Western Rate (95% CI) | Kenya Rate (95% CI) |
| --- | --- | --- | --- | --- | --- | --- | --- | --- | --- |
| 2010-2018 |  |  |  |  |  |  |  |  |  |
| <1 | 25 (11,43) | 53 (35,73) | 42 (30,58) | 93 (55,138) | 6 (2,12) | 40 (0,101) | 25 (10,41) | 61 (33,94) | 42 (29,56) |
| 1 | 48 (20,83) | 137 (103,177) | 84 (63,108) | 181 (117,275) | 11 (4,23) | 71 (0,181) | 50 (25,86) | 124 (71,187) | 102 (81,127) |
| 2 | 112 (59,178) | 279 (207,364) | 194 (153,240) | 421 (268,624) | 27 (11,50) | 124 (0,320) | 116 (63,182) | 284 (179,419) | 175 (137,219) |
| 3 | 189 (135,256) | 604 (471,758) | 334 (273,399) | 729 (485,1031) | 44 (23,73) | 232 (128,371) | 194 (145,255) | 466 (357,603) | 385 (317,457) |
| 4 | 250 (178,329) | 749 (602,929) | 440 (360,533) | 954 (635,1328) | 60 (31,98) | 273 (148,438) | 257 (192,330) | 623 (478,792) | 450 (377,538) |
| 5 | 251 (183,339) | 726 (570,909) | 446 (366,541) | 969 (647,1311) | 58 (31,98) | 311 (165,484) | 260 (191,343) | 625 (474,811) | 428 (354,509) |
| 6 | 190 (126,265) | 499 (352,646) | 285 (220,352) | 618 (397,906) | 45 (24,76) | 253 (133,385) | 195 (132,264) | 535 (389,710) | 318 (252,396) |
| 7 | 171 (111,242) | 408 (277,567) | 254 (191,321) | 552 (351,798) | 40 (21,69) | 259 (151,390) | 175 (120,243) | 480 (350,637) | 239 (180,306) |
| 8 | 171 (113,242) | 322 (207,452) | 254 (186,325) | 551 (341,792) | 40 (19,68) | 246 (131,376) | 173 (118,243) | 482 (351,638) | 268 (200,346) |
| 9 | 267 (188,359) | 503 (356,670) | 338 (265,418) | 736 (474,1046) | 62 (33,106) | 313 (202,438) | 275 (199,358) | 650 (495,821) | 362 (293,443) |
| 10 | 270 (191,374) | 513 (369,666) | 341 (273,423) | 743 (486,1081) | 63 (34,108) | 345 (231,484) | 275 (203,362) | 668 (506,850) | 359 (291,446) |
| 11 | 248 (173,338) | 388 (268,542) | 313 (241,394) | 680 (434,962) | 59 (31,97) | 303 (194,443) | 253 (182,335) | 607 (458,781) | 315 (243,390) |
| <12 | 182 (146,230) | 532 (459,618) | 315 (275,359) | 702 (486,926) | 43 (24,69) | 236 (172,306) | 188 (155,224) | 470 (389,568) | 332 (299,369) |
| 12-14 | 149 (96,218) | 313 (235,400) | 230 (187,276) | 503 (330,713) | 35 (17,61) | 273 (164,393) | 153 (101,219) | 524 (372,695) | 241 (201,288) |
| 15-17 | 141 (62,244) | 259 (182,349) | 194 (153,242) | 421 (268,610) | 32 (11,67) | 252 (149,378) | 146 (65,245) | 453 (305,629) | 204 (159,251) |
| 18-20 | 178 (94,294) | 194 (123,281) | 170 (127,216) | 369 (235,530) | 41 (17,79) | 190 (101,300) | 185 (87,298) | 375 (232,533) | 179 (134,225) |
| 21-23 | 295 (169,470) | 175 (100,256) | 177 (131,230) | 383 (249,578) | 70 (32,133) | 197 (107,307) | 313 (176,465) | 354 (214,524) | 185 (139,236) |
| 12-23 | 169 (124,216) | 238 (195,290) | 193 (166,224) | 422 (290,579) | 40 (21,68) | 226 (170,290) | 174 (130,216) | 426 (337,529) | 201 (176,230) |
| <24 | 178 (141,219) | 450 (386,526) | 277 (244,317) | 607 (422,829) | 42 (23,66) | 229 (184,280) | 183 (155,216) | 452 (375,531) | 291 (264,323) |
| 24-35 | 164 (105,238) | 160 (117,209) | 148 (118,182) | 322 (219,456) | 40 (20,67) | 181 (134,232) | 170 (113,242) | 343 (252,452) | 156 (125,185) |
| 36-47 | 160 (85,256) | 123 (77,177) | 137 (107,175) | 306 (191,440) | 37 (17,70) | 210 (150,277) | 167 (97,254) | 370 (251,502) | 145 (112,181) |
| 48-59 | 47 (11,103) | 32 (7,71) | 63 (40,91) | 135 (79,214) | 11 (2,27) | 127 (85,182) | 48 (12,100) | 218 (129,327) | 66 (42,95) |
| 24-59 | 137 (97,188) | 124 (97,156) | 126 (107,148) | 278 (187,376) | 32 (17,53) | 175 (141,213) | 139 (101,184) | 309 (243,392) | 132 (112,154) |
| <60 | 151 (121,184) | 341 (291,395) | 217 (192,246) | 473 (312,643) | 36 (19,57) | 188 (158,221) | 154 (129,182) | 358 (303,424) | 226 (204,252) |
| 2010 |  |  |  |  |  |  |  |  |  |
| <1 | 29 (12,48) | 62 (42,86) | 50 (34,67) | 108 (66,163) | 6 (2,13) | 46 (0,123) | 29 (13,50) | 74 (41,114) | 48 (34,66) |
| 1 | 95 (44,162) | 271 (212,351) | 166 (127,212) | 358 (237,525) | 22 (9,46) | 139 (0,370) | 99 (46,164) | 242 (139,367) | 203 (161,249) |
| 2 | 177 (91,288) | 440 (323,560) | 305 (239,379) | 665 (445,938) | 40 (19,78) | 194 (0,537) | 182 (94,284) | 448 (287,650) | 276 (212,341) |
| 3 | 258 (178,343) | 820 (650,1002) | 449 (368,551) | 971 (642,1391) | 60 (32,101) | 314 (166,495) | 262 (195,347) | 627 (476,805) | 520 (429,617) |
| 4 | 291 (208,390) | 872 (685,1074) | 507 (420,619) | 1115 (732,1529) | 69 (36,114) | 318 (168,489) | 298 (215,387) | 720 (556,905) | 524 (439,623) |
| 5 | 350 (249,462) | 1004 (785,1256) | 612 (495,747) | 1349 (890,1916) | 81 (44,134) | 430 (219,670) | 361 (259,469) | 868 (648,1115) | 593 (491,715) |
| 6 | 258 (169,361) | 662 (496,883) | 376 (295,474) | 821 (525,1199) | 60 (31,106) | 334 (185,520) | 262 (177,361) | 717 (528,930) | 432 (339,531) |
| 7 | 185 (122,271) | 450 (307,630) | 276 (210,359) | 601 (395,862) | 43 (20,77) | 286 (161,439) | 191 (126,266) | 535 (371,708) | 262 (198,335) |
| 8 | 167 (106,240) | 319 (195,452) | 250 (186,328) | 533 (338,813) | 40 (20,68) | 241 (137,369) | 172 (113,234) | 478 (354,619) | 266 (196,343) |
| 9 | 312 (221,424) | 583 (413,772) | 391 (312,487) | 851 (546,1229) | 74 (40,122) | 371 (237,515) | 320 (232,424) | 766 (584,975) | 428 (342,520) |
| 10 | 363 (250,509) | 678 (491,894) | 452 (351,571) | 992 (650,1474) | 84 (44,145) | 456 (301,643) | 372 (261,491) | 883 (659,1150) | 483 (384,604) |
| 11 | 305 (214,411) | 484 (331,669) | 384 (298,481) | 838 (567,1213) | 72 (37,124) | 378 (238,535) | 316 (224,415) | 744 (568,930) | 387 (305,481) |
| <12 | 228 (181,287) | 665 (577,772) | 399 (345,454) | 876 (598,1164) | 54 (31,85) | 296 (220,389) | 236 (194,284) | 596 (489,724) | 415 (377,466) |
| 12-14 | 210 (134,306) | 434 (332,554) | 319 (260,384) | 696 (468,961) | 49 (23,85) | 382 (232,554) | 213 (141,300) | 720 (523,945) | 339 (281,395) |
| 15-17 | 192 (80,332) | 348 (246,474) | 263 (207,328) | 570 (346,827) | 44 (17,89) | 333 (204,505) | 196 (85,332) | 616 (422,882) | 276 (216,346) |
| 18-20 | 267 (135,434) | 292 (187,409) | 256 (197,328) | 557 (370,814) | 63 (26,114) | 285 (159,441) | 275 (133,456) | 565 (368,804) | 269 (207,338) |
| 21-23 | 442 (238,689) | 249 (148,367) | 257 (194,336) | 565 (356,793) | 101 (45,199) | 285 (164,452) | 451 (244,685) | 515 (303,767) | 266 (198,342) |
| 12-23 | 239 (177,321) | 339 (273,412) | 273 (235,316) | 596 (396,822) | 56 (30,91) | 320 (242,409) | 248 (185,317) | 609 (479,764) | 285 (249,325) |
| <24 | 240 (192,295) | 603 (520,698) | 374 (328,425) | 817 (557,1103) | 56 (32,88) | 306 (245,370) | 245 (208,289) | 601 (508,714) | 391 (356,430) |
| 24-35 | 223 (145,313) | 212 (150,283) | 196 (160,241) | 431 (285,622) | 51 (25,92) | 243 (177,318) | 230 (148,321) | 456 (340,602) | 207 (171,246) |
| 36-47 | 234 (126,373) | 171 (112,250) | 196 (148,247) | 424 (274,608) | 53 (25,98) | 297 (214,395) | 236 (134,360) | 522 (366,715) | 205 (159,258) |
| 48-59 | 66 (15,149) | 44 (11,100) | 90 (60,129) | 194 (117,313) | 16 (3,40) | 181 (120,258) | 68 (16,148) | 311 (185,472) | 95 (59,134) |
| 24-59 | 191 (131,258) | 176 (136,223) | 176 (147,204) | 384 (262,540) | 45 (23,75) | 243 (195,299) | 194 (139,258) | 432 (345,547) | 183 (156,213) |
| <60 | 205 (166,249) | 466 (401,538) | 294 (260,333) | 655 (452,893) | 49 (28,78) | 260 (222,308) | 211 (179,245) | 493 (413,585) | 310 (282,344) |
| 2011 |  |  |  |  |  |  |  |  |  |
| <1 | 48 (21,82) | 104 (69,146) | 83 (56,113) | 183 (112,276) | 11 (4,22) | 77 (0,204) | 49 (21,81) | 120 (67,189) | 80 (57,109) |
| 1 | 84 (36,140) | 235 (179,298) | 144 (109,184) | 316 (203,464) | 20 (7,38) | 123 (0,319) | 87 (40,144) | 210 (119,333) | 177 (139,218) |
| 2 | 101 (53,161) | 251 (190,326) | 174 (135,215) | 378 (247,532) | 24 (10,45) | 112 (0,273) | 105 (55,163) | 253 (157,359) | 159 (123,199) |
| 3 | 233 (164,320) | 750 (591,928) | 411 (334,499) | 889 (612,1225) | 55 (28,89) | 289 (153,449) | 239 (173,310) | 574 (432,737) | 473 (397,562) |
| 4 | 378 (274,511) | 1153 (911,1426) | 672 (553,822) | 1437 (963,2035) | 89 (48,151) | 415 (214,666) | 393 (288,503) | 944 (727,1203) | 684 (577,822) |
| 5 | 368 (257,496) | 1035 (817,1302) | 637 (522,782) | 1408 (952,2016) | 86 (44,141) | 444 (234,730) | 372 (274,492) | 894 (684,1157) | 613 (504,736) |
| 6 | 228 (152,317) | 583 (426,783) | 337 (260,433) | 739 (480,1035) | 53 (27,92) | 302 (171,473) | 233 (155,324) | 645 (482,858) | 385 (304,474) |
| 7 | 253 (159,363) | 602 (392,842) | 373 (287,477) | 814 (525,1182) | 60 (28,101) | 383 (203,595) | 262 (177,365) | 719 (520,948) | 356 (264,450) |
| 8 | 198 (129,281) | 369 (232,519) | 293 (212,377) | 634 (391,930) | 47 (23,83) | 286 (154,442) | 202 (135,282) | 558 (417,732) | 311 (233,400) |
| 9 | 281 (199,374) | 527 (380,704) | 352 (278,439) | 774 (511,1080) | 67 (36,111) | 333 (215,468) | 283 (207,376) | 684 (517,891) | 380 (308,470) |
| 10 | 324 (223,432) | 597 (440,808) | 404 (314,502) | 866 (558,1249) | 75 (40,121) | 408 (269,567) | 328 (231,437) | 783 (602,1016) | 427 (337,527) |
| 11 | 284 (200,377) | 454 (309,632) | 363 (278,446) | 782 (514,1120) | 68 (35,112) | 351 (213,497) | 294 (214,385) | 702 (525,902) | 359 (279,446) |
| <12 | 227 (178,284) | 665 (571,767) | 398 (348,455) | 879 (586,1179) | 54 (29,87) | 298 (220,392) | 237 (193,284) | 597 (487,712) | 417 (376,467) |
| 12-14 | 162 (105,238) | 341 (263,435) | 251 (208,296) | 554 (367,754) | 38 (20,67) | 303 (177,427) | 168 (113,238) | 574 (416,746) | 264 (222,313) |
| 15-17 | 161 (66,282) | 298 (206,392) | 221 (170,281) | 482 (319,696) | 37 (13,72) | 284 (165,425) | 165 (71,281) | 516 (349,697) | 233 (185,295) |
| 18-20 | 201 (96,341) | 225 (149,316) | 197 (149,254) | 425 (270,617) | 47 (20,94) | 220 (126,339) | 210 (107,332) | 423 (274,622) | 204 (157,260) |
| 21-23 | 340 (189,522) | 193 (117,288) | 200 (150,258) | 438 (285,635) | 79 (36,152) | 226 (130,349) | 353 (197,529) | 406 (238,611) | 211 (156,272) |
| 12-23 | 190 (140,246) | 268 (218,324) | 216 (185,253) | 472 (315,641) | 45 (25,73) | 256 (197,323) | 195 (148,248) | 484 (388,592) | 227 (197,259) |
| <24 | 211 (170,260) | 538 (460,616) | 331 (293,374) | 728 (501,984) | 50 (28,77) | 272 (222,332) | 217 (184,256) | 533 (441,634) | 347 (314,386) |
| 24-35 | 199 (120,289) | 190 (139,249) | 180 (145,216) | 392 (254,556) | 47 (24,80) | 220 (164,286) | 207 (134,295) | 414 (302,554) | 188 (156,224) |
| 36-47 | 178 (89,275) | 133 (82,195) | 152 (118,192) | 331 (204,473) | 42 (18,79) | 232 (166,304) | 182 (104,285) | 400 (274,540) | 159 (125,197) |
| 48-59 | 52 (12,117) | 34 (8,78) | 69 (44,101) | 149 (86,241) | 12 (2,29) | 140 (90,197) | 51 (12,114) | 240 (138,365) | 73 (49,100) |
| 24-59 | 153 (108,208) | 142 (109,181) | 144 (120,173) | 315 (206,438) | 36 (19,63) | 199 (163,241) | 159 (115,207) | 356 (283,446) | 151 (130,176) |
| <60 | 175 (140,216) | 395 (341,461) | 252 (221,282) | 552 (386,740) | 42 (23,67) | 219 (183,261) | 181 (153,210) | 418 (347,496) | 263 (238,291) |
| 2012 |  |  |  |  |  |  |  |  |  |
| <1 | 36 (16,62) | 77 (50,110) | 62 (43,84) | 133 (81,205) | 8 (3,17) | 56 (0,147) | 36 (16,63) | 89 (47,143) | 60 (41,83) |
| 1 | 55 (25,93) | 156 (116,200) | 94 (72,121) | 207 (140,293) | 13 (5,24) | 81 (0,200) | 57 (26,94) | 139 (75,212) | 116 (90,141) |
| 2 | 98 (51,153) | 245 (178,319) | 169 (133,213) | 368 (246,533) | 23 (10,43) | 110 (0,282) | 101 (51,161) | 249 (155,355) | 155 (120,194) |
| 3 | 240 (170,319) | 765 (604,946) | 421 (339,509) | 913 (583,1300) | 57 (30,93) | 296 (157,483) | 248 (177,326) | 593 (439,772) | 491 (406,584) |
| 4 | 266 (188,355) | 807 (631,996) | 464 (384,564) | 1020 (666,1447) | 63 (33,107) | 287 (153,457) | 274 (203,358) | 663 (513,827) | 479 (398,572) |
| 5 | 272 (194,370) | 778 (600,993) | 476 (389,572) | 1031 (688,1459) | 63 (34,103) | 332 (177,509) | 278 (200,358) | 668 (502,866) | 457 (374,553) |
| 6 | 182 (123,255) | 469 (338,629) | 268 (207,337) | 579 (377,848) | 42 (22,73) | 245 (140,376) | 186 (130,254) | 518 (377,673) | 305 (241,379) |
| 7 | 158 (97,221) | 375 (245,526) | 231 (176,292) | 500 (320,743) | 37 (18,66) | 236 (130,365) | 161 (102,225) | 439 (308,582) | 219 (166,285) |
| 8 | 214 (141,300) | 397 (259,572) | 314 (230,415) | 698 (439,1009) | 49 (25,87) | 303 (172,471) | 218 (144,306) | 605 (431,786) | 337 (248,437) |
| 9 | 309 (223,414) | 577 (407,763) | 383 (305,485) | 833 (542,1182) | 71 (37,118) | 359 (233,531) | 318 (231,421) | 742 (567,929) | 415 (333,511) |
| 10 | 304 (214,417) | 568 (420,770) | 385 (307,481) | 828 (547,1171) | 72 (37,123) | 390 (253,548) | 310 (222,408) | 741 (567,945) | 405 (319,500) |
| 11 | 213 (150,284) | 336 (231,458) | 271 (210,340) | 588 (387,824) | 51 (26,82) | 262 (175,360) | 218 (156,288) | 520 (397,661) | 271 (210,332) |
| <12 | 193 (153,240) | 561 (482,651) | 336 (293,379) | 731 (500,972) | 46 (24,74) | 251 (190,328) | 199 (162,241) | 498 (414,598) | 352 (315,391) |
| 12-14 | 173 (113,249) | 367 (282,473) | 269 (220,327) | 584 (385,825) | 41 (20,70) | 319 (194,453) | 180 (115,256) | 607 (445,805) | 281 (233,334) |
| 15-17 | 163 (67,274) | 295 (212,405) | 226 (172,283) | 492 (331,716) | 37 (14,76) | 284 (170,420) | 164 (70,279) | 529 (371,727) | 237 (189,290) |
| 18-20 | 200 (95,321) | 219 (140,310) | 192 (148,246) | 415 (273,588) | 47 (19,89) | 213 (116,337) | 209 (102,339) | 410 (263,603) | 200 (154,251) |
| 21-23 | 343 (186,533) | 196 (116,298) | 202 (152,263) | 437 (264,653) | 80 (38,144) | 230 (123,348) | 353 (196,531) | 411 (252,599) | 210 (159,268) |
| 12-23 | 195 (140,254) | 272 (223,327) | 220 (187,255) | 479 (341,659) | 46 (25,76) | 259 (199,325) | 200 (149,253) | 487 (391,600) | 231 (203,262) |
| <24 | 197 (155,242) | 499 (426,575) | 306 (268,345) | 668 (467,893) | 47 (25,73) | 253 (204,303) | 202 (168,241) | 493 (409,583) | 321 (286,358) |
| 24-35 | 166 (109,242) | 159 (116,214) | 149 (120,182) | 322 (219,465) | 39 (19,69) | 184 (139,239) | 171 (115,241) | 343 (250,445) | 157 (129,188) |
| 36-47 | 174 (95,275) | 133 (85,188) | 148 (116,187) | 320 (200,470) | 42 (18,78) | 228 (164,300) | 178 (94,274) | 391 (277,540) | 155 (122,194) |
| 48-59 | 46 (11,102) | 31 (7,67) | 63 (41,89) | 136 (78,212) | 11 (2,27) | 127 (81,181) | 47 (11,103) | 216 (127,334) | 66 (43,94) |
| 24-59 | 139 (98,191) | 128 (100,163) | 129 (109,151) | 282 (190,390) | 33 (16,56) | 179 (146,217) | 144 (99,187) | 318 (254,397) | 136 (115,158) |
| <60 | 161 (128,197) | 359 (312,415) | 230 (201,259) | 496 (330,679) | 38 (22,61) | 199 (168,238) | 163 (138,191) | 381 (323,448) | 239 (216,268) |
| 2013 |  |  |  |  |  |  |  |  |  |
| <1 | 28 (12,49) | 61 (41,85) | 49 (34,66) | 105 (67,157) | 7 (2,13) | 46 (0,120) | 29 (12,50) | 70 (38,117) | 48 (33,64) |
| 1 | 36 (16,60) | 99 (74,129) | 61 (46,77) | 132 (83,193) | 8 (3,16) | 52 (0,131) | 36 (17,60) | 89 (52,136) | 74 (59,91) |
| 2 | 123 (68,197) | 300 (223,396) | 212 (163,265) | 464 (288,637) | 29 (14,54) | 134 (0,335) | 125 (67,193) | 309 (190,463) | 192 (150,236) |
| 3 | 222 (158,297) | 707 (562,878) | 387 (317,469) | 847 (545,1206) | 51 (26,87) | 274 (145,435) | 230 (164,302) | 547 (420,699) | 451 (375,531) |
| 4 | 311 (223,418) | 933 (740,1156) | 546 (452,652) | 1189 (783,1656) | 72 (38,123) | 340 (176,543) | 319 (237,414) | 761 (595,973) | 558 (470,658) |
| 5 | 298 (205,403) | 848 (658,1057) | 514 (420,628) | 1126 (762,1586) | 69 (38,118) | 362 (189,581) | 298 (219,396) | 721 (542,940) | 493 (405,595) |
| 6 | 195 (125,272) | 505 (361,675) | 288 (227,365) | 627 (421,909) | 46 (23,81) | 264 (142,407) | 198 (136,275) | 554 (414,728) | 331 (260,400) |
| 7 | 178 (116,252) | 424 (280,600) | 268 (198,341) | 576 (352,852) | 42 (21,72) | 269 (150,410) | 183 (124,255) | 511 (371,673) | 248 (188,321) |
| 8 | 174 (113,246) | 323 (207,449) | 253 (185,330) | 551 (349,800) | 40 (18,71) | 248 (141,380) | 176 (115,244) | 485 (349,635) | 269 (201,341) |
| 9 | 332 (241,446) | 635 (436,843) | 424 (336,520) | 921 (615,1323) | 81 (43,136) | 398 (258,569) | 346 (256,457) | 812 (620,1057) | 458 (375,565) |
| 10 | 260 (183,355) | 491 (348,654) | 328 (254,411) | 712 (463,1032) | 62 (32,106) | 333 (212,468) | 265 (192,347) | 635 (483,814) | 345 (270,425) |
| 11 | 238 (166,319) | 382 (257,520) | 301 (233,379) | 653 (418,942) | 55 (30,93) | 295 (186,407) | 246 (180,324) | 584 (448,742) | 304 (234,384) |
| <12 | 194 (154,239) | 559 (480,647) | 335 (294,379) | 727 (493,991) | 46 (26,74) | 250 (184,323) | 198 (162,238) | 499 (412,595) | 350 (316,388) |
| 12-14 | 152 (98,221) | 318 (240,399) | 232 (192,281) | 504 (336,698) | 35 (18,63) | 275 (173,402) | 155 (98,221) | 531 (384,702) | 242 (203,285) |
| 15-17 | 168 (71,295) | 311 (218,429) | 238 (186,297) | 513 (342,721) | 40 (13,82) | 301 (177,451) | 174 (74,294) | 554 (374,774) | 247 (201,306) |
| 18-20 | 182 (87,293) | 199 (129,282) | 175 (136,227) | 377 (245,541) | 42 (17,84) | 199 (98,304) | 191 (95,311) | 377 (239,538) | 182 (139,229) |
| 21-23 | 325 (185,506) | 189 (111,272) | 192 (143,247) | 412 (265,622) | 74 (33,145) | 214 (120,338) | 338 (192,521) | 398 (238,575) | 203 (154,256) |
| 12-23 | 183 (135,235) | 256 (210,315) | 206 (177,241) | 455 (308,600) | 42 (22,70) | 245 (184,305) | 187 (142,238) | 460 (362,557) | 216 (189,249) |
| <24 | 190 (151,233) | 479 (414,555) | 297 (259,335) | 648 (441,868) | 45 (24,72) | 244 (199,295) | 194 (164,229) | 476 (405,565) | 310 (278,344) |
| 24-35 | 174 (112,264) | 170 (118,220) | 158 (129,193) | 343 (229,473) | 41 (21,72) | 192 (141,251) | 180 (117,258) | 366 (265,481) | 164 (136,195) |
| 36-47 | 160 (89,247) | 121 (77,178) | 136 (105,169) | 296 (190,430) | 36 (17,70) | 206 (151,275) | 162 (87,256) | 362 (249,485) | 142 (111,177) |
| 48-59 | 56 (12,120) | 37 (9,76) | 75 (49,107) | 161 (92,253) | 13 (2,32) | 152 (98,215) | 56 (13,120) | 256 (152,398) | 78 (51,110) |
| 24-59 | 145 (102,195) | 134 (104,170) | 134 (112,158) | 295 (201,402) | 34 (17,57) | 187 (153,228) | 150 (108,199) | 332 (261,423) | 142 (121,166) |
| <60 | 162 (131,197) | 365 (315,419) | 231 (204,264) | 502 (347,682) | 38 (21,60) | 201 (170,239) | 165 (139,194) | 384 (326,451) | 242 (219,269) |
| 2014 |  |  |  |  |  |  |  |  |  |
| <1 | 12 (5,20) | 25 (17,35) | 20 (14,27) | 44 (25,66) | 3 (1,5) | 19 (0,49) | 12 (5,20) | 29 (16,47) | 19 (13,26) |
| 1 | 39 (18,65) | 109 (82,136) | 67 (51,85) | 142 (90,209) | 9 (4,18) | 57 (0,142) | 39 (19,66) | 97 (56,147) | 81 (64,100) |
| 2 | 123 (68,198) | 304 (223,398) | 213 (168,266) | 467 (306,651) | 29 (12,53) | 133 (0,358) | 125 (66,198) | 307 (197,446) | 193 (149,237) |
| 3 | 146 (103,199) | 472 (371,586) | 259 (207,316) | 562 (389,791) | 35 (18,58) | 182 (95,285) | 150 (107,196) | 363 (272,473) | 299 (246,361) |
| 4 | 217 (160,281) | 646 (517,814) | 379 (308,453) | 825 (545,1172) | 51 (27,84) | 235 (117,372) | 221 (161,291) | 529 (400,676) | 387 (327,452) |
| 5 | 202 (143,270) | 577 (450,703) | 349 (280,423) | 760 (515,1084) | 47 (24,79) | 246 (126,384) | 205 (148,265) | 493 (371,644) | 336 (278,403) |
| 6 | 184 (128,259) | 474 (340,617) | 273 (208,341) | 590 (384,845) | 43 (23,75) | 247 (134,375) | 188 (125,260) | 518 (387,675) | 309 (245,383) |
| 7 | 191 (127,269) | 444 (300,621) | 273 (211,354) | 597 (370,880) | 44 (23,75) | 282 (155,424) | 193 (125,265) | 531 (387,709) | 259 (194,332) |
| 8 | 244 (161,339) | 459 (293,655) | 367 (269,474) | 791 (492,1126) | 58 (29,103) | 355 (199,558) | 254 (168,357) | 699 (513,912) | 386 (284,496) |
| 9 | 303 (213,413) | 570 (406,760) | 382 (308,478) | 836 (537,1184) | 74 (38,123) | 357 (233,501) | 312 (225,410) | 749 (581,965) | 412 (328,504) |
| 10 | 292 (206,390) | 546 (402,722) | 366 (291,455) | 792 (518,1113) | 68 (34,115) | 371 (244,517) | 300 (215,395) | 719 (532,913) | 386 (299,474) |
| 11 | 261 (189,359) | 420 (284,594) | 334 (254,418) | 725 (477,1070) | 61 (33,103) | 320 (217,456) | 270 (196,365) | 641 (497,824) | 334 (261,417) |
| <12 | 187 (146,233) | 535 (460,613) | 320 (281,361) | 704 (470,948) | 43 (24,72) | 240 (176,302) | 191 (155,228) | 477 (400,575) | 336 (303,375) |
| 12-14 | 140 (89,200) | 291 (228,369) | 214 (179,257) | 465 (308,654) | 32 (16,57) | 254 (152,369) | 143 (93,200) | 485 (359,636) | 222 (185,262) |
| 15-17 | 169 (74,287) | 311 (215,425) | 235 (178,294) | 509 (348,748) | 40 (14,79) | 296 (177,447) | 173 (76,293) | 551 (365,752) | 245 (191,308) |
| 18-20 | 175 (82,284) | 191 (126,272) | 168 (130,215) | 362 (237,524) | 41 (18,79) | 187 (103,294) | 179 (88,287) | 363 (235,534) | 176 (138,226) |
| 21-23 | 349 (199,538) | 200 (117,299) | 204 (148,268) | 448 (292,654) | 80 (36,151) | 229 (120,356) | 358 (200,531) | 410 (240,630) | 214 (163,273) |
| 12-23 | 178 (130,234) | 252 (207,310) | 204 (176,237) | 447 (307,619) | 42 (22,69) | 238 (182,302) | 184 (140,232) | 447 (360,566) | 213 (185,246) |
| <24 | 184 (151,223) | 467 (401,535) | 289 (252,326) | 631 (427,846) | 43 (24,67) | 236 (192,287) | 188 (158,224) | 466 (389,544) | 302 (271,332) |
| 24-35 | 174 (112,256) | 169 (124,227) | 159 (129,194) | 350 (236,494) | 43 (22,72) | 196 (145,253) | 182 (121,264) | 368 (273,485) | 167 (137,199) |
| 36-47 | 181 (96,285) | 138 (85,202) | 155 (121,197) | 338 (220,494) | 42 (19,78) | 237 (172,314) | 185 (98,291) | 412 (279,570) | 163 (130,204) |
| 48-59 | 52 (12,111) | 36 (8,75) | 71 (45,102) | 151 (90,243) | 12 (2,30) | 144 (93,205) | 54 (13,116) | 243 (141,375) | 74 (48,105) |
| 24-59 | 151 (108,202) | 140 (108,178) | 140 (120,165) | 300 (214,420) | 35 (19,58) | 194 (157,239) | 155 (113,201) | 345 (269,431) | 146 (125,170) |
| <60 | 162 (133,195) | 363 (316,422) | 232 (205,263) | 510 (337,689) | 38 (21,62) | 202 (170,239) | 164 (142,194) | 386 (327,452) | 242 (219,270) |
| 2015 |  |  |  |  |  |  |  |  |  |
| <1 | 17 (7,29) | 37 (24,51) | 29 (20,39) | 63 (38,99) | 4 (1,8) | 27 (0,72) | 17 (7,29) | 43 (23,67) | 28 (19,38) |
| 1 | 29 (13,47) | 81 (62,104) | 50 (38,64) | 109 (70,154) | 7 (2,13) | 42 (0,112) | 29 (13,49) | 73 (41,112) | 61 (48,75) |
| 2 | 74 (41,118) | 185 (137,234) | 129 (99,162) | 277 (180,402) | 17 (8,32) | 81 (0,203) | 77 (40,121) | 187 (119,276) | 117 (92,147) |
| 3 | 144 (102,193) | 459 (358,577) | 252 (204,306) | 545 (370,778) | 34 (18,57) | 177 (89,271) | 147 (108,193) | 356 (272,459) | 292 (241,346) |
| 4 | 198 (144,261) | 594 (473,734) | 348 (287,420) | 758 (522,1068) | 45 (25,75) | 212 (105,341) | 202 (147,262) | 487 (376,620) | 354 (297,421) |
| 5 | 122 (88,166) | 354 (273,438) | 217 (178,261) | 470 (314,664) | 29 (15,49) | 148 (77,237) | 127 (92,165) | 304 (229,385) | 207 (169,251) |
| 6 | 161 (112,232) | 430 (314,567) | 244 (190,304) | 535 (360,771) | 38 (19,69) | 222 (123,339) | 168 (116,224) | 470 (347,612) | 276 (217,339) |
| 7 | 156 (100,224) | 382 (254,522) | 235 (177,303) | 517 (323,729) | 38 (18,65) | 240 (141,357) | 160 (105,225) | 453 (321,592) | 221 (167,284) |
| 8 | 128 (83,184) | 241 (162,349) | 191 (139,247) | 418 (256,612) | 31 (16,51) | 185 (105,282) | 134 (92,185) | 366 (266,497) | 203 (149,261) |
| 9 | 129 (89,171) | 240 (169,316) | 162 (125,202) | 356 (231,496) | 31 (15,52) | 153 (100,216) | 131 (94,173) | 311 (239,394) | 173 (141,212) |
| 10 | 173 (121,235) | 325 (233,439) | 220 (174,276) | 467 (308,675) | 40 (21,69) | 220 (142,317) | 177 (125,236) | 421 (321,538) | 229 (182,284) |
| 11 | 163 (116,221) | 258 (180,352) | 204 (157,256) | 450 (288,650) | 38 (21,62) | 200 (132,272) | 168 (122,217) | 396 (306,504) | 202 (162,254) |
| <12 | 126 (99,159) | 366 (315,421) | 218 (193,249) | 476 (331,644) | 30 (16,47) | 164 (120,209) | 129 (105,155) | 324 (267,393) | 228 (206,254) |
| 12-14 | 104 (66,149) | 218 (165,278) | 161 (132,195) | 350 (236,486) | 25 (12,43) | 191 (118,270) | 108 (67,154) | 364 (271,477) | 170 (143,202) |
| 15-17 | 72 (31,127) | 132 (93,178) | 100 (78,125) | 216 (140,317) | 17 (6,34) | 128 (76,184) | 74 (33,126) | 233 (160,322) | 104 (83,131) |
| 18-20 | 101 (55,165) | 113 (74,158) | 98 (76,124) | 210 (133,303) | 23 (10,44) | 110 (60,167) | 104 (55,166) | 213 (132,305) | 101 (79,128) |
| 21-23 | 188 (110,292) | 108 (66,158) | 112 (82,147) | 242 (156,355) | 45 (20,82) | 123 (69,192) | 196 (106,290) | 227 (125,338) | 117 (87,149) |
| 12-23 | 101 (73,135) | 142 (118,172) | 115 (99,135) | 251 (178,347) | 24 (13,38) | 134 (103,175) | 104 (79,133) | 255 (202,317) | 121 (105,138) |
| <24 | 113 (91,140) | 290 (249,334) | 179 (157,205) | 393 (260,523) | 27 (15,44) | 148 (120,179) | 117 (99,140) | 288 (240,342) | 187 (170,210) |
| 24-35 | 118 (79,171) | 114 (82,154) | 107 (88,131) | 233 (157,323) | 28 (14,51) | 132 (98,177) | 122 (78,178) | 252 (186,324) | 112 (92,134) |
| 36-47 | 109 (56,170) | 85 (55,119) | 94 (72,119) | 207 (136,294) | 25 (11,48) | 142 (105,188) | 111 (59,174) | 249 (173,339) | 99 (77,124) |
| 48-59 | 31 (7,67) | 21 (5,47) | 43 (27,61) | 91 (53,145) | 7 (1,19) | 87 (56,120) | 32 (7,67) | 146 (86,221) | 45 (28,62) |
| 24-59 | 94 (66,127) | 87 (66,111) | 88 (74,104) | 191 (126,259) | 22 (11,36) | 121 (99,145) | 96 (69,128) | 216 (170,268) | 92 (79,107) |
| <60 | 101 (80,122) | 227 (196,260) | 144 (127,163) | 319 (221,423) | 24 (13,37) | 126 (107,147) | 103 (88,121) | 240 (201,282) | 151 (136,168) |
| 2016 |  |  |  |  |  |  |  |  |  |
| <1 | 9 (4,16) | 20 (14,28) | 16 (11,22) | 35 (21,53) | 2 (1,4) | 15 (0,41) | 10 (4,17) | 24 (13,36) | 16 (11,21) |
| 1 | 20 (9,34) | 57 (43,73) | 35 (27,45) | 78 (50,109) | 5 (2,10) | 30 (0,75) | 21 (10,36) | 52 (30,79) | 43 (34,53) |
| 2 | 112 (61,179) | 276 (208,359) | 193 (150,240) | 419 (272,602) | 26 (12,49) | 121 (0,312) | 115 (62,177) | 284 (172,411) | 175 (137,220) |
| 3 | 107 (76,144) | 343 (266,427) | 188 (152,228) | 410 (264,582) | 25 (13,41) | 131 (72,207) | 109 (79,142) | 262 (200,336) | 217 (180,259) |
| 4 | 162 (116,218) | 491 (384,601) | 285 (229,347) | 620 (406,854) | 38 (21,65) | 182 (96,283) | 168 (121,216) | 403 (313,512) | 294 (246,350) |
| 5 | 175 (126,233) | 503 (396,634) | 307 (250,378) | 668 (443,937) | 42 (21,70) | 213 (121,338) | 180 (131,234) | 433 (331,549) | 297 (242,357) |
| 6 | 171 (116,241) | 449 (326,576) | 258 (196,321) | 553 (365,788) | 40 (20,71) | 233 (128,345) | 178 (118,244) | 489 (358,638) | 291 (234,361) |
| 7 | 132 (83,189) | 310 (211,440) | 192 (146,245) | 421 (268,622) | 31 (15,51) | 200 (111,302) | 136 (88,189) | 368 (272,492) | 185 (137,238) |
| 8 | 99 (64,143) | 184 (120,266) | 145 (108,189) | 321 (197,461) | 23 (12,41) | 142 (81,213) | 102 (65,141) | 283 (205,375) | 155 (114,203) |
| 9 | 234 (168,324) | 443 (310,598) | 298 (237,367) | 653 (437,927) | 55 (30,94) | 280 (185,388) | 242 (176,320) | 572 (445,735) | 321 (258,393) |
| 10 | 262 (185,357) | 495 (355,657) | 333 (260,418) | 713 (476,1020) | 61 (31,105) | 334 (218,460) | 272 (192,354) | 641 (483,819) | 352 (280,430) |
| 11 | 248 (176,331) | 398 (267,537) | 311 (235,396) | 686 (443,991) | 58 (31,99) | 305 (200,428) | 255 (186,343) | 606 (466,767) | 314 (247,393) |
| <12 | 136 (106,173) | 394 (341,459) | 237 (207,268) | 520 (358,687) | 33 (17,51) | 177 (132,227) | 141 (115,171) | 354 (293,427) | 248 (225,279) |
| 12-14 | 131 (84,191) | 269 (207,345) | 197 (163,239) | 430 (281,603) | 30 (15,52) | 237 (146,341) | 134 (84,186) | 447 (331,585) | 208 (174,246) |
| 15-17 | 104 (43,182) | 188 (130,257) | 143 (110,181) | 312 (206,451) | 22 (9,50) | 183 (108,267) | 104 (44,179) | 337 (219,483) | 149 (118,183) |
| 18-20 | 155 (85,261) | 174 (114,239) | 152 (115,194) | 328 (205,487) | 37 (14,72) | 169 (91,255) | 160 (80,260) | 330 (211,473) | 158 (122,200) |
| 21-23 | 199 (114,316) | 114 (67,170) | 117 (89,155) | 260 (160,378) | 47 (21,89) | 129 (74,202) | 206 (117,315) | 236 (139,343) | 124 (92,159) |
| 12-23 | 133 (97,173) | 187 (154,230) | 151 (131,176) | 332 (223,450) | 31 (15,53) | 177 (136,227) | 137 (103,177) | 338 (268,416) | 158 (137,179) |
| <24 | 137 (111,169) | 345 (294,401) | 213 (187,241) | 469 (310,636) | 32 (18,50) | 176 (143,213) | 141 (117,167) | 346 (285,414) | 223 (202,249) |
| 24-35 | 123 (77,175) | 117 (85,157) | 109 (88,134) | 241 (155,336) | 29 (14,51) | 135 (100,177) | 126 (86,179) | 253 (185,336) | 115 (94,137) |
| 36-47 | 141 (76,231) | 109 (68,160) | 122 (94,155) | 267 (174,382) | 33 (16,61) | 190 (138,247) | 148 (83,231) | 328 (226,452) | 129 (102,161) |
| 48-59 | 36 (8,82) | 25 (6,52) | 50 (32,71) | 108 (64,175) | 8 (2,22) | 101 (64,144) | 37 (9,79) | 171 (96,259) | 52 (34,74) |
| 24-59 | 108 (77,147) | 101 (77,126) | 101 (86,120) | 220 (146,302) | 25 (13,43) | 140 (113,172) | 111 (80,149) | 247 (196,311) | 105 (91,123) |
| <60 | 118 (94,145) | 267 (231,306) | 169 (150,192) | 370 (253,518) | 28 (15,43) | 149 (125,174) | 121 (104,142) | 282 (240,331) | 178 (159,196) |
| 2017 |  |  |  |  |  |  |  |  |  |
| <1 | 23 (10,39) | 51 (34,71) | 40 (29,54) | 87 (51,136) | 5 (2,11) | 37 (0,94) | 24 (10,40) | 59 (31,93) | 39 (28,53) |
| 1 | 36 (17,60) | 102 (77,130) | 62 (46,80) | 136 (86,191) | 8 (3,16) | 53 (0,135) | 37 (17,62) | 91 (53,137) | 76 (59,94) |
| 2 | 98 (48,156) | 244 (182,315) | 171 (132,210) | 377 (246,522) | 23 (10,44) | 108 (0,274) | 102 (51,161) | 251 (162,359) | 157 (123,194) |
| 3 | 192 (138,260) | 610 (472,762) | 337 (269,411) | 718 (479,1031) | 45 (24,75) | 237 (133,365) | 194 (139,257) | 470 (354,607) | 389 (323,464) |
| 4 | 269 (192,360) | 802 (646,993) | 474 (386,566) | 1027 (680,1414) | 63 (33,103) | 293 (152,474) | 278 (202,359) | 656 (493,830) | 483 (397,577) |
| 5 | 241 (173,326) | 688 (536,871) | 419 (341,512) | 911 (610,1298) | 56 (31,93) | 294 (154,452) | 242 (179,319) | 589 (443,779) | 404 (333,491) |
| 6 | 141 (93,198) | 374 (274,497) | 212 (163,264) | 464 (312,665) | 33 (16,56) | 193 (107,295) | 145 (98,201) | 405 (303,543) | 241 (189,298) |
| 7 | 125 (82,179) | 298 (202,413) | 183 (142,233) | 396 (249,578) | 28 (14,49) | 189 (109,286) | 128 (80,181) | 354 (261,474) | 173 (133,217) |
| 8 | 168 (108,243) | 324 (207,469) | 254 (189,339) | 553 (339,812) | 40 (20,70) | 249 (141,365) | 176 (115,252) | 488 (361,646) | 270 (201,347) |
| 9 | 218 (150,297) | 412 (294,548) | 274 (218,340) | 598 (385,838) | 52 (28,91) | 262 (171,367) | 224 (162,294) | 533 (411,669) | 295 (233,360) |
| 10 | 187 (132,248) | 348 (253,466) | 235 (183,296) | 512 (343,735) | 43 (22,75) | 236 (152,337) | 194 (138,250) | 450 (346,587) | 248 (194,299) |
| 11 | 285 (203,377) | 450 (304,624) | 358 (276,448) | 776 (507,1099) | 66 (33,108) | 342 (229,487) | 295 (214,383) | 702 (542,883) | 359 (283,453) |
| <12 | 163 (128,203) | 470 (403,549) | 281 (248,320) | 616 (420,822) | 38 (21,61) | 210 (157,273) | 166 (138,202) | 421 (342,505) | 294 (265,330) |
| 12-14 | 127 (82,181) | 265 (203,344) | 194 (163,235) | 425 (287,593) | 31 (15,54) | 234 (139,341) | 132 (84,186) | 439 (325,582) | 205 (171,245) |
| 15-17 | 105 (48,184) | 198 (138,266) | 150 (117,189) | 325 (209,465) | 25 (9,52) | 190 (112,286) | 110 (46,182) | 350 (241,494) | 157 (123,193) |
| 18-20 | 164 (80,270) | 183 (120,263) | 159 (121,200) | 344 (214,487) | 38 (16,76) | 176 (95,279) | 171 (77,275) | 343 (217,506) | 167 (128,212) |
| 21-23 | 234 (136,364) | 134 (78,200) | 139 (105,180) | 301 (189,453) | 56 (25,101) | 156 (91,234) | 240 (138,367) | 281 (166,419) | 146 (110,190) |
| 12-23 | 142 (102,188) | 201 (163,242) | 162 (137,188) | 352 (238,486) | 33 (17,56) | 190 (145,245) | 144 (110,187) | 357 (287,441) | 168 (147,193) |
| <24 | 152 (126,190) | 390 (335,450) | 240 (212,274) | 521 (357,704) | 37 (20,57) | 197 (161,244) | 158 (132,186) | 387 (322,462) | 251 (227,278) |
| 24-35 | 149 (94,213) | 142 (101,190) | 133 (108,163) | 287 (190,403) | 34 (17,61) | 163 (119,216) | 151 (102,211) | 303 (223,394) | 138 (114,167) |
| 36-47 | 130 (71,207) | 97 (63,141) | 110 (85,138) | 241 (153,337) | 29 (13,56) | 169 (122,217) | 130 (72,201) | 290 (201,402) | 115 (90,144) |
| 48-59 | 37 (9,79) | 25 (6,55) | 50 (31,72) | 111 (65,171) | 9 (2,22) | 102 (64,146) | 38 (9,87) | 173 (102,264) | 52 (35,75) |
| 24-59 | 113 (80,152) | 104 (80,132) | 105 (88,124) | 231 (155,319) | 27 (14,45) | 144 (116,177) | 115 (84,153) | 257 (209,324) | 109 (93,128) |
| <60 | 128 (102,155) | 287 (248,332) | 183 (161,207) | 406 (272,545) | 30 (17,48) | 160 (136,190) | 131 (112,155) | 305 (255,359) | 192 (173,212) |
| 2018 |  |  |  |  |  |  |  |  |  |
| <1 | 22 (11,39) | 49 (32,68) | 39 (28,53) | 86 (51,127) | 5 (2,11) | 37 (0,96) | 23 (10,40) | 57 (29,90) | 38 (27,52) |
| 1 | 43 (19,73) | 120 (90,156) | 73 (56,92) | 159 (100,234) | 10 (4,19) | 63 (0,169) | 44 (22,74) | 106 (63,162) | 90 (71,110) |
| 2 | 101 (57,162) | 254 (183,331) | 176 (137,219) | 379 (242,557) | 24 (11,44) | 111 (0,286) | 104 (54,161) | 258 (165,369) | 159 (125,201) |
| 3 | 175 (123,235) | 555 (432,693) | 303 (246,367) | 665 (450,938) | 40 (20,67) | 210 (113,338) | 178 (129,232) | 430 (325,547) | 353 (291,415) |
| 4 | 193 (138,258) | 587 (464,714) | 341 (278,411) | 743 (508,1050) | 47 (26,77) | 214 (110,335) | 200 (147,258) | 484 (370,617) | 351 (289,413) |
| 5 | 260 (186,355) | 746 (569,936) | 462 (375,559) | 1007 (692,1391) | 61 (34,99) | 321 (177,490) | 270 (195,356) | 646 (492,832) | 440 (362,530) |
| 6 | 181 (117,258) | 469 (351,619) | 263 (208,332) | 575 (371,822) | 42 (22,74) | 240 (132,360) | 186 (128,252) | 510 (374,653) | 299 (237,373) |
| 7 | 184 (120,267) | 453 (291,624) | 277 (208,349) | 603 (406,885) | 44 (22,78) | 284 (158,433) | 191 (129,266) | 529 (385,700) | 260 (196,332) |
| 8 | 182 (119,255) | 335 (221,476) | 267 (192,344) | 589 (374,859) | 42 (21,74) | 262 (144,377) | 185 (121,255) | 512 (379,675) | 283 (208,361) |
| 9 | 273 (195,370) | 513 (362,690) | 341 (269,421) | 749 (495,1062) | 64 (34,107) | 320 (209,456) | 279 (204,368) | 666 (520,844) | 370 (298,453) |
| 10 | 286 (201,387) | 536 (389,718) | 356 (286,451) | 787 (503,1118) | 67 (35,113) | 364 (230,522) | 290 (207,384) | 683 (531,899) | 381 (307,466) |
| 11 | 240 (169,328) | 377 (260,513) | 301 (235,382) | 655 (418,936) | 57 (30,96) | 295 (189,413) | 246 (183,323) | 581 (446,755) | 304 (236,380) |
| <12 | 181 (144,221) | 522 (451,602) | 312 (273,355) | 678 (465,912) | 42 (23,67) | 232 (172,306) | 185 (154,225) | 467 (387,564) | 327 (295,365) |
| 12-14 | 149 (97,213) | 311 (236,401) | 228 (187,275) | 502 (322,697) | 34 (17,60) | 272 (165,395) | 152 (100,219) | 518 (367,678) | 238 (201,285) |
| 15-17 | 136 (63,230) | 252 (173,341) | 189 (144,240) | 412 (262,592) | 32 (13,63) | 242 (149,364) | 142 (62,237) | 445 (305,619) | 198 (156,250) |
| 18-20 | 141 (68,230) | 157 (105,223) | 137 (104,173) | 293 (195,435) | 33 (14,62) | 154 (84,237) | 146 (70,234) | 299 (192,433) | 144 (113,178) |
| 21-23 | 259 (148,422) | 150 (91,221) | 154 (115,201) | 330 (210,489) | 62 (29,115) | 167 (94,262) | 269 (146,411) | 314 (192,458) | 161 (118,207) |
| 12-23 | 152 (112,199) | 215 (175,264) | 174 (149,205) | 380 (259,522) | 37 (19,58) | 204 (154,263) | 157 (118,202) | 386 (305,473) | 182 (158,209) |
| <24 | 167 (134,205) | 425 (367,497) | 263 (229,299) | 571 (390,775) | 40 (20,61) | 216 (171,264) | 172 (147,202) | 424 (357,502) | 273 (247,305) |
| 24-35 | 152 (98,216) | 145 (106,195) | 137 (113,163) | 296 (198,413) | 36 (18,65) | 167 (123,220) | 156 (100,219) | 318 (230,412) | 142 (118,173) |
| 36-47 | 140 (79,221) | 110 (72,158) | 123 (94,153) | 268 (173,376) | 34 (15,64) | 188 (138,246) | 148 (78,230) | 327 (221,447) | 128 (98,160) |
| 48-59 | 41 (9,84) | 28 (6,59) | 56 (36,81) | 120 (70,191) | 10 (2,23) | 112 (73,159) | 42 (10,90) | 188 (116,289) | 59 (37,81) |
| 24-59 | 121 (89,166) | 113 (85,141) | 113 (96,134) | 245 (164,343) | 28 (15,48) | 157 (128,190) | 125 (92,167) | 281 (221,350) | 118 (101,139) |
| <60 | 137 (110,167) | 311 (268,357) | 199 (175,225) | 429 (292,588) | 33 (19,51) | 172 (147,203) | 141 (118,168) | 329 (277,388) | 207 (188,230) |

**Table S6: Annual rates of hospitalized severe acute respiratory illness per 100,000 children by regions and age in months, 2010-2018**

| Year and Age in months | Central Rate (95% CI) | Coast Rate (95% CI) | Eastern Rate (95% CI) | North Eastern Rate (95% CI) | Nairobi Rate (95% CI) | Nyanza Rate (95% CI) | Rift Valley Rate (95% CI) | Western Rate (95% CI) | Kenya Rate (95% CI) |
| --- | --- | --- | --- | --- | --- | --- | --- | --- | --- |
| 2010-2018 |  |  |  |  |  |  |  |  |  |
| <1 | 6442 (4957,8027) | 6790 (5626,8102) | 8188 (6849,9643) | 5360 (3493,7558) | 3574 (2284,5457) | 8606 (7296,10129) | 7966 (6903,9264) | 6503 (5300,7887) | 7110 (6202,8160) |
| 1 | 9290 (7203,11737) | 9851 (8132,11879) | 11817 (9818,13863) | 7762 (5014,10713) | 5099 (3061,8060) | 12434 (10450,14724) | 11531 (10018,13421) | 9389 (7753,11384) | 10221 (8966,11818) |
| 2 | 6810 (5281,8491) | 7165 (5892,8641) | 8693 (7300,10313) | 5691 (3759,7921) | 3715 (2338,5798) | 9016 (7567,10728) | 8368 (7233,9773) | 6854 (5608,8391) | 7468 (6557,8576) |
| 3 | 6471 (5070,8099) | 6849 (5664,8246) | 8205 (6950,9739) | 5353 (3601,7387) | 3569 (2299,5779) | 8683 (7339,10216) | 8111 (6908,9492) | 6567 (5398,8068) | 7180 (6261,8241) |
| 4 | 5877 (4584,7305) | 6170 (5089,7398) | 7442 (6349,8894) | 4904 (3280,6636) | 3254 (2000,5059) | 7873 (6578,9319) | 7305 (6268,8529) | 5912 (4880,7078) | 6451 (5646,7344) |
| 5 | 6396 (5008,8326) | 6805 (5620,8186) | 8239 (6883,9700) | 5306 (3625,7450) | 3567 (2184,5711) | 8603 (7260,10139) | 7989 (6856,9277) | 6473 (5238,7961) | 7110 (6221,8116) |
| 6 | 6295 (4974,7839) | 6707 (5539,8106) | 8098 (6858,9517) | 5313 (3486,7452) | 3444 (2156,5322) | 8422 (7143,9824) | 7847 (6741,9072) | 6334 (5270,7699) | 6978 (6047,8060) |
| 7 | 5412 (4236,6927) | 5739 (4754,6964) | 6901 (5861,8245) | 4509 (3077,6200) | 3035 (1864,4525) | 7246 (6049,8565) | 6749 (5738,7817) | 5461 (4566,6602) | 6024 (5223,6967) |
| 8 | 5049 (4002,6261) | 5362 (4354,6488) | 6463 (5431,7694) | 4262 (2972,5855) | 2814 (1672,4390) | 6824 (5690,8072) | 6321 (5428,7353) | 5131 (4235,6294) | 5646 (4863,6459) |
| 9 | 4003 (3155,5045) | 4227 (3468,5092) | 5114 (4303,6082) | 3341 (2286,4591) | 2233 (1342,3511) | 5390 (4579,6422) | 5053 (4330,5825) | 4079 (3318,4969) | 4469 (3894,5133) |
| 10 | 4452 (3532,5620) | 4726 (3848,5616) | 5679 (4809,6731) | 3728 (2458,5119) | 2483 (1549,3880) | 5942 (5035,6954) | 5586 (4792,6476) | 4497 (3739,5466) | 4958 (4341,5676) |
| 11 | 3654 (2841,4613) | 3867 (3191,4694) | 4643 (3864,5509) | 3072 (2053,4201) | 2026 (1253,3162) | 4905 (4193,5827) | 4564 (3920,5290) | 3729 (3015,4430) | 4060 (3560,4643) |
| <12 | 5847 (4611,7272) | 6229 (5140,7403) | 7486 (6262,8870) | 4886 (3318,6877) | 3266 (2079,5068) | 7907 (6722,9443) | 7344 (6342,8391) | 5906 (4925,7256) | 6493 (5607,7458) |
| 12-14 | 3257 (2546,4130) | 3465 (2882,4151) | 4118 (3470,4943) | 2702 (1848,3764) | 1775 (1102,2800) | 4316 (3656,5137) | 4072 (3475,4740) | 3303 (2708,4016) | 3608 (3142,4158) |
| 15-17 | 2697 (2078,3406) | 2863 (2374,3434) | 3431 (2895,4108) | 2269 (1543,3086) | 1497 (895,2223) | 3649 (3045,4341) | 3364 (2915,3895) | 2745 (2255,3341) | 2984 (2616,3460) |
| 18-20 | 2731 (2139,3459) | 2899 (2376,3420) | 3489 (2920,4081) | 2276 (1528,3138) | 1483 (941,2317) | 3653 (3091,4311) | 3393 (2923,3927) | 2766 (2286,3365) | 3030 (2594,3498) |
| 21-23 | 1936 (1510,2451) | 2043 (1666,2470) | 2449 (2057,2905) | 1623 (1073,2215) | 1075 (655,1664) | 2585 (2182,3074) | 2412 (2096,2800) | 1955 (1622,2377) | 2128 (1856,2465) |
| 12-23 | 2653 (2098,3361) | 2821 (2338,3389) | 3386 (2798,3990) | 2228 (1488,3078) | 1492 (889,2301) | 3573 (2994,4265) | 3312 (2841,3840) | 2689 (2217,3283) | 2945 (2565,3393) |
| <24 | 4207 (3319,5341) | 4494 (3719,5475) | 5445 (4628,6386) | 3582 (2397,4908) | 2371 (1481,3626) | 5686 (4829,6678) | 5352 (4587,6188) | 4326 (3558,5292) | 4737 (4126,5427) |
| 24-35 | 1349 (1072,1695) | 1439 (1182,1730) | 1734 (1456,2059) | 1132 (770,1534) | 759 (467,1148) | 1821 (1566,2151) | 1691 (1448,1953) | 1364 (1122,1676) | 1505 (1320,1724) |
| 36-47 | 879 (686,1097) | 925 (757,1104) | 1121 (947,1322) | 725 (504,996) | 488 (290,764) | 1170 (984,1385) | 1090 (953,1281) | 888 (725,1072) | 972 (845,1120) |
| 48-59 | 515 (403,644) | 542 (448,646) | 649 (540,769) | 432 (282,587) | 285 (175,431) | 691 (587,822) | 641 (551,750) | 518 (422,630) | 569 (493,653) |
| 24-59 | 920 (718,1152) | 968 (799,1165) | 1168 (974,1354) | 757 (513,1039) | 506 (324,804) | 1224 (1037,1449) | 1143 (991,1318) | 934 (750,1141) | 1018 (886,1164) |
| <60 | 2257 (1794,2862) | 2383 (1975,2877) | 2866 (2442,3401) | 1896 (1268,2597) | 1277 (789,1995) | 3020 (2540,3616) | 2807 (2415,3247) | 2265 (1861,2792) | 2501 (2182,2869) |
| 2010 |  |  |  |  |  |  |  |  |  |
| <1 | 12160 (9500,15650) | 12884 (10579,15959) | 15435 (13066,18374) | 10112 (6763,14087) | 6753 (4161,10615) | 16285 (13762,19431) | 15108 (13038,17733) | 12328 (10061,14953) | 13410 (11770,15475) |
| 1 | 12066 (9548,15196) | 12890 (10558,15414) | 15421 (13040,18029) | 10117 (6626,14227) | 6646 (3923,10440) | 16216 (13684,19493) | 15121 (12920,17637) | 12215 (10057,15026) | 13433 (11592,15346) |
| 2 | 10113 (8089,13023) | 10761 (8944,12760) | 12770 (10885,15334) | 8555 (5458,11789) | 5531 (3370,8582) | 13474 (11408,16048) | 12656 (10978,14747) | 10210 (8399,12488) | 11252 (9781,12904) |
| 3 | 9400 (7305,11901) | 9997 (8243,12192) | 12041 (10056,14288) | 7906 (5426,10736) | 5265 (3306,8195) | 12674 (10625,14936) | 11796 (10016,13691) | 9541 (7727,11576) | 10475 (9095,11927) |
| 4 | 7104 (5523,8949) | 7543 (6212,9115) | 9001 (7575,10670) | 5953 (4032,8195) | 3944 (2467,6086) | 9511 (7985,11120) | 8809 (7587,10341) | 7241 (5841,8753) | 7852 (6846,9072) |
| 5 | 9237 (7299,11547) | 9887 (8174,11970) | 11840 (10025,14089) | 7786 (5280,10961) | 5209 (3212,8211) | 12469 (10591,14662) | 11602 (9955,13344) | 9492 (7762,11602) | 10291 (9012,11739) |
| 6 | 11122 (8572,13840) | 11675 (9709,13940) | 14131 (11756,16664) | 9138 (5897,12686) | 6140 (3843,9642) | 14790 (12509,17369) | 13799 (11962,16162) | 11226 (9296,13583) | 12210 (10812,14216) |
| 7 | 10229 (7953,12670) | 10670 (8896,12866) | 13023 (11147,15287) | 8510 (5751,11593) | 5599 (3451,9023) | 13720 (11459,16129) | 12734 (11059,14730) | 10278 (8359,12464) | 11231 (9848,12861) |
| 8 | 6358 (4985,7986) | 6713 (5702,8117) | 8083 (6854,9493) | 5334 (3493,7178) | 3540 (2184,5313) | 8520 (7166,9994) | 7950 (6801,9208) | 6415 (5279,7766) | 7051 (6050,8154) |
| 9 | 8229 (6436,10436) | 8569 (7136,10362) | 10365 (8767,12206) | 6831 (4663,9399) | 4552 (2804,7111) | 10898 (9195,12879) | 10162 (8831,11691) | 8161 (6737,10010) | 8969 (7741,10275) |
| 10 | 9466 (7500,11852) | 9932 (8313,11924) | 11913 (9984,14219) | 7782 (5300,10727) | 5123 (3332,8229) | 12650 (10629,14997) | 11728 (10039,13787) | 9510 (7851,11520) | 10358 (9030,12012) |
| 11 | 7766 (6093,9882) | 8224 (6832,9733) | 9895 (8389,11707) | 6471 (4459,8790) | 4322 (2681,6601) | 10512 (8846,12435) | 9726 (8376,11207) | 7949 (6459,9551) | 8629 (7529,9880) |
| <12 | 9500 (7398,11979) | 10018 (8083,12058) | 11985 (10161,14207) | 7857 (5285,10738) | 5250 (3242,8141) | 12634 (10585,14936) | 11787 (10145,13722) | 9520 (7764,11532) | 10420 (9037,11904) |
| 12-14 | 5398 (4183,6647) | 5719 (4722,6851) | 6788 (5780,8096) | 4422 (3106,6220) | 2974 (1822,4596) | 7206 (6038,8534) | 6659 (5709,7805) | 5432 (4531,6529) | 5932 (5171,6806) |
| 15-17 | 4643 (3613,5835) | 4842 (4013,5801) | 5831 (4875,6866) | 3806 (2562,5160) | 2506 (1556,3847) | 6157 (5259,7269) | 5721 (4970,6613) | 4636 (3784,5664) | 5077 (4451,5882) |
| 18-20 | 4309 (3370,5407) | 4548 (3718,5489) | 5475 (4556,6492) | 3596 (2385,4968) | 2401 (1546,3765) | 5778 (4812,6834) | 5337 (4621,6242) | 4340 (3579,5281) | 4754 (4143,5454) |
| 21-23 | 2483 (1921,3090) | 2611 (2177,3173) | 3127 (2637,3722) | 2046 (1402,2774) | 1350 (866,2107) | 3319 (2786,3973) | 3073 (2620,3578) | 2502 (2016,3059) | 2727 (2379,3107) |
| 12-23 | 4137 (3315,5210) | 4399 (3630,5297) | 5290 (4518,6339) | 3539 (2363,4789) | 2276 (1426,3727) | 5580 (4725,6590) | 5178 (4473,5998) | 4202 (3451,5165) | 4610 (4039,5266) |
| <24 | 6620 (5187,8285) | 7062 (5919,8501) | 8495 (7068,9995) | 5516 (3570,7636) | 3658 (2323,5710) | 8878 (7552,10487) | 8311 (7134,9534) | 6689 (5546,8177) | 7336 (6404,8386) |
| 24-35 | 2115 (1635,2661) | 2231 (1822,2708) | 2681 (2277,3200) | 1764 (1149,2392) | 1165 (723,1772) | 2815 (2369,3343) | 2624 (2272,3037) | 2139 (1746,2626) | 2338 (2028,2662) |
| 36-47 | 1563 (1258,1975) | 1671 (1378,2007) | 1989 (1661,2378) | 1304 (878,1806) | 860 (537,1313) | 2114 (1765,2498) | 1958 (1675,2273) | 1575 (1299,1911) | 1743 (1517,2000) |
| 48-59 | 874 (698,1083) | 921 (773,1113) | 1099 (926,1314) | 719 (482,1000) | 475 (294,732) | 1157 (975,1363) | 1084 (940,1250) | 876 (718,1063) | 965 (838,1111) |
| 24-59 | 1531 (1195,1912) | 1624 (1322,1947) | 1930 (1655,2294) | 1281 (857,1727) | 846 (521,1300) | 2039 (1722,2404) | 1901 (1649,2189) | 1539 (1267,1846) | 1684 (1479,1943) |
| <60 | 3564 (2731,4527) | 3760 (3124,4474) | 4517 (3762,5326) | 2960 (1979,4148) | 1997 (1233,3091) | 4768 (4024,5672) | 4421 (3837,5118) | 3607 (2919,4351) | 3918 (3428,4560) |
| 2011 |  |  |  |  |  |  |  |  |  |
| <1 | 8636 (6748,10879) | 9168 (7605,11005) | 11033 (9280,13092) | 7201 (4556,9780) | 4861 (3071,7479) | 11622 (9894,13800) | 10840 (9217,12470) | 8775 (7206,10602) | 9574 (8390,10955) |
| 1 | 9710 (7424,12102) | 10221 (8490,12227) | 12281 (10516,14650) | 8038 (5283,10973) | 5399 (3344,8262) | 13003 (10919,15196) | 12090 (10386,13946) | 9802 (8065,11837) | 10683 (9399,12349) |
| 2 | 7193 (5660,9018) | 7590 (6314,9059) | 9185 (7693,10974) | 6030 (4107,8348) | 3977 (2473,6244) | 9654 (8109,11593) | 9075 (7794,10555) | 7291 (5883,8813) | 7972 (6964,9138) |
| 3 | 9850 (7746,12264) | 10354 (8618,12604) | 12475 (10591,14898) | 8146 (5499,11259) | 5463 (3346,8480) | 13142 (11144,15575) | 12237 (10678,14195) | 9880 (8104,12044) | 10847 (9393,12507) |
| 4 | 7752 (6020,9862) | 8211 (6837,9885) | 9825 (8253,11522) | 6463 (4263,8908) | 4271 (2594,6576) | 10346 (8756,12268) | 9672 (8281,11190) | 7813 (6350,9610) | 8515 (7474,9858) |
| 5 | 7423 (5792,9300) | 7892 (6500,9443) | 9431 (7998,11254) | 6160 (4141,8592) | 4024 (2497,6439) | 9968 (8398,11603) | 9249 (7911,10738) | 7494 (6016,9094) | 8181 (7146,9461) |
| 6 | 8652 (6838,10981) | 9175 (7434,11156) | 10941 (9345,13059) | 7232 (4988,9841) | 4774 (2976,7569) | 11581 (9671,13521) | 10735 (9360,12434) | 8767 (7159,10559) | 9596 (8420,11011) |
| 7 | 9543 (7505,11833) | 10070 (8322,11984) | 12086 (10083,14478) | 7912 (5216,11014) | 5267 (3277,8471) | 12682 (10666,14985) | 11788 (10112,13531) | 9491 (7830,11633) | 10508 (9164,12059) |
| 8 | 6044 (4732,7626) | 6379 (5315,7627) | 7745 (6458,9021) | 5008 (3248,6960) | 3360 (2069,5128) | 8096 (6775,9608) | 7526 (6523,8727) | 6109 (5025,7495) | 6669 (5860,7655) |
| 9 | 4141 (3237,5225) | 4398 (3625,5250) | 5267 (4489,6290) | 3488 (2339,4780) | 2298 (1417,3569) | 5591 (4782,6539) | 5217 (4459,5983) | 4222 (3516,5067) | 4598 (4004,5359) |
| 10 | 4777 (3860,6025) | 5104 (4180,6057) | 6078 (5081,7224) | 4018 (2713,5439) | 2651 (1575,4156) | 6425 (5399,7589) | 5984 (5156,6918) | 4815 (3908,5930) | 5323 (4595,6117) |
| 11 | 5303 (4115,6715) | 5608 (4625,6657) | 6761 (5752,7943) | 4426 (2907,6088) | 2908 (1820,4602) | 7083 (6044,8442) | 6629 (5744,7743) | 5385 (4363,6522) | 5882 (5201,6832) |
| <12 | 7371 (5708,9283) | 7856 (6482,9467) | 9445 (7935,11051) | 6087 (4058,8417) | 4044 (2534,6270) | 9851 (8365,11677) | 9166 (7904,10649) | 7455 (6111,9019) | 8157 (7148,9321) |
| 12-14 | 3569 (2787,4494) | 3729 (3120,4550) | 4514 (3852,5294) | 2946 (2011,4098) | 1980 (1247,3072) | 4751 (4039,5599) | 4427 (3778,5125) | 3599 (2955,4340) | 3926 (3388,4507) |
| 15-17 | 2945 (2349,3749) | 3159 (2596,3761) | 3762 (3176,4449) | 2493 (1654,3338) | 1661 (1046,2490) | 3968 (3317,4685) | 3700 (3186,4302) | 3001 (2477,3686) | 3276 (2874,3748) |
| 18-20 | 4353 (3470,5514) | 4637 (3880,5569) | 5571 (4670,6550) | 3610 (2461,5038) | 2423 (1513,3814) | 5849 (4860,6884) | 5455 (4667,6352) | 4436 (3591,5367) | 4875 (4211,5549) |
| 21-23 | 2713 (2105,3438) | 2905 (2416,3467) | 3491 (2967,4080) | 2300 (1498,3101) | 1539 (923,2373) | 3645 (3079,4305) | 3416 (2907,3912) | 2765 (2316,3343) | 3032 (2648,3474) |
| 12-23 | 3352 (2668,4253) | 3574 (2955,4245) | 4274 (3629,5024) | 2818 (1949,3834) | 1872 (1157,2799) | 4489 (3789,5323) | 4208 (3590,4897) | 3429 (2768,4129) | 3732 (3224,4340) |
| <24 | 5479 (4299,6929) | 5814 (4800,7012) | 6987 (5853,8329) | 4570 (3109,6190) | 2969 (1866,4639) | 7376 (6259,8633) | 6829 (5897,7899) | 5556 (4563,6798) | 6078 (5261,6967) |
| 24-35 | 1612 (1251,2031) | 1711 (1436,2049) | 2044 (1725,2422) | 1337 (914,1850) | 888 (554,1380) | 2142 (1802,2526) | 2008 (1715,2341) | 1630 (1315,1968) | 1783 (1556,2047) |
| 36-47 | 1069 (816,1358) | 1127 (925,1335) | 1363 (1140,1602) | 882 (584,1247) | 590 (358,906) | 1425 (1208,1684) | 1327 (1141,1552) | 1079 (890,1299) | 1184 (1030,1350) |
| 48-59 | 670 (527,861) | 709 (579,837) | 847 (716,1006) | 551 (378,762) | 373 (232,577) | 891 (754,1055) | 832 (724,965) | 669 (547,825) | 738 (648,850) |
| 24-59 | 1132 (876,1431) | 1200 (981,1457) | 1433 (1193,1692) | 949 (648,1308) | 621 (392,961) | 1511 (1274,1778) | 1407 (1233,1626) | 1133 (917,1385) | 1246 (1085,1424) |
| <60 | 2861 (2223,3620) | 3025 (2539,3626) | 3608 (3061,4272) | 2387 (1593,3251) | 1579 (958,2484) | 3808 (3201,4527) | 3550 (3080,4129) | 2882 (2350,3485) | 3164 (2737,3609) |
| 2012 |  |  |  |  |  |  |  |  |  |
| <1 | 8686 (6663,11090) | 9211 (7618,11120) | 11066 (9276,13024) | 7156 (4956,10049) | 4873 (2889,7537) | 11784 (9851,13879) | 10861 (9325,12637) | 8787 (7295,10709) | 9643 (8362,10975) |
| 1 | 10029 (7784,12501) | 10538 (8782,12765) | 12738 (10715,14957) | 8243 (5434,11435) | 5523 (3362,8612) | 13387 (11307,15679) | 12449 (10839,14428) | 10123 (8253,12384) | 11061 (9615,12655) |
| 2 | 7122 (5619,9049) | 7565 (6361,9155) | 9127 (7680,10731) | 5889 (3814,8345) | 3963 (2435,6228) | 9605 (8074,11276) | 8935 (7653,10341) | 7197 (5885,8885) | 7939 (6882,9139) |
| 3 | 5189 (4052,6500) | 5462 (4497,6507) | 6560 (5510,7820) | 4277 (2878,5894) | 2901 (1785,4464) | 6880 (5768,8157) | 6423 (5510,7433) | 5223 (4202,6382) | 5712 (4987,6508) |
| 4 | 6926 (5371,8742) | 7320 (5973,8778) | 8737 (7434,10436) | 5770 (3870,7865) | 3918 (2413,5883) | 9235 (7696,11051) | 8608 (7379,10027) | 7001 (5718,8551) | 7605 (6680,8747) |
| 5 | 9715 (7634,12232) | 10291 (8530,12451) | 12359 (10376,14563) | 8164 (5494,11349) | 5472 (3319,8166) | 13080 (11070,15637) | 12190 (10476,14102) | 9799 (8057,11969) | 10751 (9337,12369) |
| 6 | 8958 (7084,11363) | 9538 (7876,11446) | 11407 (9745,13460) | 7500 (4973,10187) | 4929 (3191,7763) | 12029 (10263,14158) | 11248 (9656,12997) | 9066 (7369,10932) | 10009 (8704,11531) |
| 7 | 5074 (3981,6392) | 5424 (4448,6503) | 6465 (5417,7592) | 4199 (2829,5774) | 2874 (1774,4327) | 6849 (5713,8052) | 6378 (5440,7318) | 5151 (4208,6274) | 5624 (4929,6515) |
| 8 | 6357 (5015,8222) | 6796 (5616,8240) | 8100 (6884,9552) | 5273 (3705,7184) | 3490 (2172,5355) | 8555 (7115,10143) | 7986 (6832,9221) | 6447 (5297,7752) | 7021 (6157,8159) |
| 9 | 4468 (3451,5607) | 4746 (3940,5657) | 5684 (4882,6655) | 3745 (2460,5067) | 2490 (1541,3881) | 5972 (5013,7076) | 5596 (4762,6504) | 4491 (3670,5463) | 4939 (4330,5687) |
| 10 | 4397 (3418,5533) | 4657 (3881,5564) | 5578 (4698,6525) | 3655 (2520,5119) | 2395 (1459,3807) | 5881 (4959,6956) | 5460 (4719,6348) | 4437 (3591,5423) | 4834 (4245,5528) |
| 11 | 4165 (3246,5244) | 4395 (3584,5296) | 5296 (4444,6259) | 3515 (2236,4744) | 2276 (1410,3542) | 5565 (4699,6583) | 5159 (4464,5974) | 4197 (3458,5089) | 4601 (4006,5335) |
| <12 | 6736 (5261,8355) | 7144 (5924,8605) | 8560 (7247,10241) | 5593 (3754,7858) | 3763 (2366,5876) | 9028 (7645,10612) | 8357 (7218,9722) | 6780 (5549,8315) | 7411 (6475,8510) |
| 12-14 | 3628 (2847,4620) | 3860 (3180,4631) | 4605 (3906,5496) | 3050 (2060,4114) | 2007 (1224,3156) | 4873 (4037,5716) | 4522 (3837,5283) | 3681 (3034,4456) | 4008 (3482,4620) |
| 15-17 | 3607 (2747,4556) | 3792 (3115,4522) | 4541 (3811,5409) | 2962 (1952,4090) | 1988 (1223,3131) | 4790 (4073,5666) | 4452 (3810,5199) | 3604 (2974,4392) | 3954 (3455,4531) |
| 18-20 | 3316 (2593,4162) | 3533 (2904,4250) | 4239 (3593,5009) | 2779 (1894,3868) | 1831 (1090,2882) | 4438 (3724,5264) | 4140 (3566,4823) | 3346 (2772,4055) | 3681 (3207,4228) |
| 21-23 | 1667 (1328,2086) | 1771 (1440,2099) | 2141 (1789,2504) | 1391 (963,1892) | 933 (569,1412) | 2235 (1883,2647) | 2081 (1803,2422) | 1691 (1378,2071) | 1852 (1617,2115) |
| 12-23 | 3087 (2436,3825) | 3264 (2702,3930) | 3888 (3282,4582) | 2541 (1706,3443) | 1692 (1087,2611) | 4089 (3452,4909) | 3795 (3288,4387) | 3106 (2555,3739) | 3381 (2907,3889) |
| <24 | 4817 (3749,5934) | 5123 (4224,6154) | 6132 (5143,7204) | 4004 (2686,5551) | 2672 (1546,4216) | 6472 (5491,7677) | 6015 (5156,6936) | 4870 (3988,5933) | 5356 (4656,6175) |
| 24-35 | 1454 (1134,1816) | 1537 (1264,1891) | 1835 (1545,2197) | 1213 (788,1677) | 792 (508,1223) | 1940 (1635,2288) | 1805 (1556,2123) | 1462 (1208,1799) | 1607 (1404,1853) |
| 36-47 | 828 (656,1044) | 874 (718,1050) | 1059 (879,1257) | 700 (467,955) | 465 (274,715) | 1105 (937,1305) | 1032 (893,1201) | 838 (679,1019) | 920 (808,1051) |
| 48-59 | 490 (382,612) | 519 (427,624) | 624 (527,729) | 410 (276,575) | 272 (157,424) | 657 (548,779) | 613 (527,715) | 495 (407,598) | 544 (474,625) |
| 24-59 | 908 (703,1131) | 957 (790,1147) | 1153 (965,1361) | 766 (520,1043) | 505 (322,798) | 1221 (1032,1443) | 1138 (973,1313) | 924 (762,1150) | 1005 (882,1158) |
| <60 | 2477 (1992,3139) | 2636 (2203,3160) | 3168 (2699,3796) | 2092 (1417,2865) | 1394 (846,2062) | 3362 (2847,3959) | 3101 (2667,3617) | 2534 (2077,3083) | 2765 (2408,3185) |
| 2013 |  |  |  |  |  |  |  |  |  |
| <1 | 3835 (2978,4779) | 4086 (3390,4834) | 4906 (4153,5798) | 3223 (2112,4383) | 2151 (1299,3450) | 5103 (4286,6059) | 4827 (4089,5622) | 3892 (3206,4796) | 4262 (3695,4854) |
| 1 | 5852 (4539,7367) | 6178 (5177,7481) | 7444 (6182,8736) | 4851 (3207,6645) | 3227 (2037,4975) | 7770 (6534,9188) | 7288 (6251,8416) | 5894 (4856,7127) | 6440 (5613,7465) |
| 2 | 4882 (3872,6216) | 5233 (4351,6360) | 6298 (5283,7530) | 4173 (2893,5735) | 2735 (1742,4300) | 6650 (5548,7872) | 6112 (5302,7176) | 4985 (4149,6079) | 5459 (4738,6265) |
| 3 | 3941 (3104,4987) | 4140 (3458,4950) | 4971 (4176,5902) | 3279 (2243,4509) | 2209 (1410,3269) | 5267 (4435,6260) | 4886 (4252,5704) | 3958 (3335,4867) | 4364 (3780,5027) |
| 4 | 4592 (3578,5800) | 4859 (4061,5877) | 5815 (4871,6917) | 3814 (2643,5313) | 2534 (1577,3878) | 6184 (5132,7186) | 5720 (4905,6583) | 4641 (3724,5596) | 5094 (4444,5853) |
| 5 | 5450 (4262,6913) | 5763 (4757,6903) | 6938 (5847,8212) | 4495 (3043,6237) | 3014 (1920,4629) | 7241 (6150,8650) | 6791 (5856,7818) | 5501 (4496,6681) | 6013 (5222,6911) |
| 6 | 4625 (3643,5780) | 4903 (4019,5887) | 5944 (4988,6882) | 3889 (2590,5327) | 2571 (1585,3896) | 6186 (5148,7362) | 5757 (4985,6733) | 4728 (3842,5627) | 5123 (4432,5869) |
| 7 | 3507 (2739,4399) | 3717 (3043,4450) | 4458 (3763,5260) | 2906 (1931,4039) | 1910 (1217,3102) | 4695 (3953,5500) | 4350 (3782,5046) | 3561 (2929,4316) | 3872 (3368,4437) |
| 8 | 3028 (2379,3769) | 3224 (2665,3872) | 3876 (3216,4546) | 2536 (1662,3394) | 1687 (1058,2590) | 4080 (3435,4813) | 3806 (3255,4421) | 3076 (2513,3731) | 3359 (2926,3875) |
| 9 | 1381 (1094,1761) | 1463 (1211,1772) | 1769 (1509,2103) | 1168 (794,1624) | 771 (478,1228) | 1861 (1577,2228) | 1740 (1497,1998) | 1407 (1153,1688) | 1547 (1341,1779) |
| 10 | 3001 (2330,3777) | 3165 (2607,3800) | 3789 (3227,4458) | 2481 (1651,3421) | 1656 (1047,2605) | 4013 (3375,4715) | 3730 (3229,4346) | 3036 (2484,3655) | 3308 (2879,3789) |
| 11 | 2212 (1735,2795) | 2358 (1931,2801) | 2815 (2383,3374) | 1839 (1245,2571) | 1229 (767,1856) | 2962 (2500,3527) | 2761 (2371,3219) | 2229 (1817,2723) | 2452 (2152,2779) |
| <12 | 3877 (3071,4867) | 4138 (3434,4996) | 5002 (4176,5895) | 3250 (2137,4545) | 2163 (1343,3307) | 5239 (4400,6229) | 4869 (4202,5629) | 3925 (3216,4794) | 4338 (3763,4961) |
| 12-14 | 3118 (2455,3919) | 3334 (2752,3997) | 3975 (3343,4708) | 2611 (1763,3670) | 1737 (1052,2571) | 4193 (3536,4917) | 3883 (3331,4523) | 3182 (2596,3857) | 3472 (3068,4008) |
| 15-17 | 1526 (1199,1914) | 1601 (1328,1910) | 1937 (1625,2300) | 1268 (862,1704) | 857 (519,1309) | 2037 (1722,2448) | 1897 (1619,2208) | 1531 (1255,1889) | 1685 (1453,1928) |
| 18-20 | 1658 (1313,2128) | 1772 (1460,2129) | 2120 (1792,2508) | 1386 (920,1949) | 921 (587,1470) | 2246 (1907,2657) | 2085 (1784,2403) | 1689 (1383,2036) | 1853 (1611,2112) |
| 21-23 | 1676 (1284,2088) | 1760 (1441,2113) | 2119 (1781,2516) | 1396 (960,1933) | 923 (537,1432) | 2221 (1876,2609) | 2068 (1789,2407) | 1683 (1367,2016) | 1840 (1603,2105) |
| 12-23 | 2016 (1583,2547) | 2133 (1772,2573) | 2562 (2149,3028) | 1683 (1130,2352) | 1109 (651,1739) | 2674 (2253,3174) | 2521 (2170,2929) | 2042 (1679,2458) | 2214 (1943,2557) |
| <24 | 2976 (2339,3760) | 3133 (2563,3776) | 3796 (3193,4518) | 2484 (1714,3394) | 1642 (1029,2568) | 3964 (3371,4727) | 3690 (3217,4285) | 3017 (2459,3675) | 3302 (2871,3809) |
| 24-35 | 876 (687,1097) | 919 (764,1108) | 1114 (925,1327) | 726 (491,1001) | 492 (292,744) | 1169 (987,1381) | 1094 (949,1255) | 889 (727,1080) | 968 (850,1109) |
| 36-47 | 515 (398,659) | 546 (456,657) | 656 (556,789) | 427 (281,589) | 289 (181,444) | 693 (583,820) | 645 (558,753) | 524 (426,643) | 573 (496,653) |
| 48-59 | 288 (226,368) | 308 (255,366) | 366 (310,438) | 239 (165,320) | 160 (98,249) | 387 (325,452) | 359 (310,417) | 294 (239,356) | 320 (277,368) |
| 24-59 | 562 (443,713) | 596 (491,719) | 715 (598,836) | 474 (314,655) | 312 (189,489) | 753 (626,884) | 697 (603,811) | 569 (469,681) | 622 (541,719) |
| <60 | 1528 (1214,1932) | 1620 (1326,1932) | 1930 (1614,2263) | 1254 (839,1722) | 842 (512,1301) | 2026 (1699,2408) | 1885 (1609,2198) | 1533 (1262,1877) | 1677 (1460,1936) |
| 2014 |  |  |  |  |  |  |  |  |  |
| <1 | 4891 (3791,6192) | 5181 (4288,6280) | 6194 (5252,7361) | 4055 (2706,5455) | 2753 (1681,4187) | 6581 (5487,7772) | 6105 (5230,7081) | 4959 (4079,6048) | 5402 (4686,6164) |
| 1 | 9480 (7408,11871) | 9970 (8163,11929) | 12005 (10092,14354) | 7821 (5175,10736) | 5177 (3269,7953) | 12629 (10713,14963) | 11797 (10056,13655) | 9506 (7797,11588) | 10460 (9134,11929) |
| 2 | 6241 (4973,8127) | 6572 (5430,8090) | 7942 (6650,9346) | 5249 (3557,7310) | 3450 (2096,5336) | 8370 (7001,9879) | 7837 (6801,9037) | 6300 (5163,7584) | 6927 (6031,7894) |
| 3 | 5885 (4450,7460) | 6129 (5106,7439) | 7435 (6284,8804) | 4836 (3209,6615) | 3215 (1929,5058) | 7796 (6571,9201) | 7267 (6284,8535) | 5930 (4911,7100) | 6464 (5611,7465) |
| 4 | 6183 (4906,7760) | 6615 (5467,7973) | 7968 (6726,9422) | 5122 (3528,7202) | 3518 (2046,5251) | 8312 (6962,9872) | 7732 (6680,9017) | 6244 (5160,7636) | 6894 (6031,7850) |
| 5 | 5750 (4503,7163) | 6073 (4922,7341) | 7291 (6212,8502) | 4777 (3296,6582) | 3184 (1950,4904) | 7666 (6440,9059) | 7122 (6144,8219) | 5786 (4627,7080) | 6334 (5518,7274) |
| 6 | 6349 (4978,8070) | 6745 (5575,8090) | 8105 (6839,9575) | 5314 (3684,7262) | 3542 (2222,5598) | 8559 (7094,10154) | 7918 (6863,9234) | 6495 (5378,7837) | 7073 (6151,8075) |
| 7 | 6781 (5278,8322) | 7160 (5928,8538) | 8571 (7125,10152) | 5620 (3801,7863) | 3742 (2299,5782) | 9017 (7605,10759) | 8377 (7274,9728) | 6783 (5546,8292) | 7484 (6496,8569) |
| 8 | 5194 (4103,6621) | 5497 (4525,6583) | 6611 (5639,7818) | 4349 (2986,6075) | 2877 (1774,4564) | 6977 (5799,8215) | 6453 (5627,7549) | 5234 (4297,6472) | 5776 (5046,6595) |
| 9 | 3812 (2958,4856) | 4056 (3321,4880) | 4871 (4054,5786) | 3216 (2121,4442) | 2107 (1276,3290) | 5126 (4360,6008) | 4776 (4130,5552) | 3866 (3167,4684) | 4236 (3669,4907) |
| 10 | 4259 (3349,5339) | 4504 (3674,5422) | 5440 (4592,6512) | 3546 (2369,4934) | 2347 (1464,3577) | 5698 (4812,6825) | 5335 (4569,6154) | 4316 (3500,5330) | 4747 (4144,5464) |
| 11 | 3051 (2360,3795) | 3199 (2671,3840) | 3843 (3267,4491) | 2517 (1667,3433) | 1663 (1018,2638) | 4058 (3471,4811) | 3752 (3241,4336) | 3048 (2522,3749) | 3362 (2912,3866) |
| <12 | 5639 (4463,7141) | 5993 (4943,7124) | 7177 (5998,8544) | 4715 (3224,6543) | 3168 (1912,4837) | 7538 (6339,8992) | 7051 (6076,8156) | 5726 (4701,6980) | 6270 (5470,7186) |
| 12-14 | 2893 (2236,3643) | 3070 (2562,3683) | 3703 (3094,4387) | 2393 (1612,3366) | 1608 (1014,2499) | 3885 (3247,4584) | 3606 (3132,4188) | 2950 (2402,3553) | 3202 (2779,3676) |
| 15-17 | 2409 (1901,3047) | 2552 (2111,3070) | 3075 (2560,3614) | 2014 (1375,2730) | 1336 (834,2007) | 3226 (2726,3822) | 2990 (2581,3504) | 2425 (1990,3006) | 2673 (2343,3049) |
| 18-20 | 2642 (2029,3322) | 2795 (2283,3340) | 3363 (2830,3964) | 2200 (1476,3071) | 1449 (866,2281) | 3532 (3006,4144) | 3292 (2849,3798) | 2673 (2170,3207) | 2921 (2538,3357) |
| 21-23 | 2210 (1764,2746) | 2369 (1955,2841) | 2860 (2410,3400) | 1852 (1221,2524) | 1236 (745,1899) | 2979 (2560,3526) | 2778 (2422,3210) | 2252 (1836,2712) | 2472 (2143,2811) |
| 12-23 | 2536 (2015,3213) | 2693 (2249,3285) | 3240 (2711,3832) | 2160 (1441,2930) | 1436 (884,2269) | 3415 (2889,4130) | 3190 (2732,3708) | 2572 (2078,3150) | 2831 (2449,3269) |
| <24 | 4161 (3247,5275) | 4431 (3687,5234) | 5310 (4455,6225) | 3517 (2453,4775) | 2318 (1362,3676) | 5591 (4756,6674) | 5203 (4522,6022) | 4242 (3468,5158) | 4625 (4013,5274) |
| 24-35 | 1531 (1206,1918) | 1628 (1342,1935) | 1944 (1652,2317) | 1285 (869,1791) | 849 (530,1324) | 2052 (1725,2406) | 1906 (1628,2245) | 1534 (1253,1859) | 1704 (1458,1954) |
| 36-47 | 836 (659,1047) | 881 (731,1062) | 1056 (894,1242) | 694 (463,954) | 462 (287,737) | 1108 (938,1303) | 1039 (898,1200) | 839 (688,1029) | 917 (797,1057) |
| 48-59 | 467 (356,581) | 494 (410,589) | 592 (501,702) | 387 (260,529) | 264 (163,417) | 624 (522,731) | 579 (503,676) | 469 (381,561) | 515 (454,585) |
| 24-59 | 939 (741,1199) | 1003 (824,1219) | 1202 (1013,1430) | 777 (518,1090) | 522 (348,810) | 1262 (1078,1485) | 1175 (1002,1370) | 949 (788,1175) | 1040 (911,1194) |
| <60 | 2270 (1788,2876) | 2390 (1990,2861) | 2880 (2444,3394) | 1879 (1246,2625) | 1273 (778,1954) | 3021 (2564,3559) | 2796 (2410,3271) | 2281 (1870,2759) | 2500 (2181,2873) |
| 2015 |  |  |  |  |  |  |  |  |  |
| <1 | 3431 (2690,4347) | 3635 (2973,4324) | 4362 (3715,5174) | 2810 (1880,3855) | 1875 (1157,2851) | 4577 (3883,5417) | 4264 (3706,4991) | 3474 (2840,4207) | 3780 (3316,4336) |
| 1 | 9328 (7234,11661) | 9857 (8104,11971) | 11945 (10100,14066) | 7755 (5405,10667) | 5143 (3203,7973) | 12482 (10460,14767) | 11693 (10051,13517) | 9420 (7739,11510) | 10361 (9041,11928) |
| 2 | 7496 (5864,9340) | 7888 (6603,9435) | 9463 (7926,11294) | 6215 (4089,8463) | 4108 (2594,6387) | 9942 (8356,11807) | 9266 (8077,10721) | 7497 (6032,9208) | 8208 (7085,9450) |
| 3 | 6214 (4810,7745) | 6591 (5485,7847) | 7918 (6646,9382) | 5175 (3463,7095) | 3507 (2053,5383) | 8341 (6994,9972) | 7775 (6741,9140) | 6287 (5054,7632) | 6894 (5978,7910) |
| 4 | 5740 (4547,7223) | 6129 (5023,7327) | 7324 (6138,8686) | 4777 (3259,6594) | 3206 (2025,4940) | 7717 (6484,9037) | 7227 (6192,8411) | 5823 (4810,7028) | 6369 (5565,7322) |
| 5 | 6507 (4960,8098) | 6907 (5739,8413) | 8273 (6976,9942) | 5474 (3728,7558) | 3676 (2211,5579) | 8704 (7337,10246) | 8102 (7061,9359) | 6563 (5396,8125) | 7222 (6218,8295) |
| 6 | 5689 (4418,7251) | 6044 (4966,7276) | 7266 (6097,8557) | 4663 (3087,6416) | 3157 (1929,4827) | 7636 (6424,8986) | 7061 (6102,8221) | 5747 (4683,7020) | 6287 (5461,7308) |
| 7 | 4360 (3406,5452) | 4644 (3814,5646) | 5556 (4649,6576) | 3619 (2502,5007) | 2393 (1520,3754) | 5859 (4901,6879) | 5433 (4653,6233) | 4389 (3593,5325) | 4843 (4205,5592) |
| 8 | 3936 (3050,5017) | 4195 (3442,5033) | 5016 (4252,6002) | 3270 (2205,4460) | 2175 (1323,3404) | 5315 (4477,6205) | 4967 (4255,5746) | 3995 (3292,4861) | 4366 (3853,5074) |
| 9 | 4495 (3544,5594) | 4769 (4036,5757) | 5685 (4833,6779) | 3770 (2544,5085) | 2498 (1519,3835) | 6043 (5119,7048) | 5585 (4767,6547) | 4546 (3703,5502) | 4996 (4315,5699) |
| 10 | 4790 (3726,6074) | 5058 (4206,6174) | 6101 (5159,7179) | 3961 (2785,5409) | 2660 (1588,4077) | 6422 (5415,7592) | 5984 (5195,6969) | 4856 (3994,6006) | 5294 (4642,6108) |
| 11 | 3431 (2701,4359) | 3625 (3017,4364) | 4339 (3703,5191) | 2860 (1943,3932) | 1910 (1183,3001) | 4634 (3896,5456) | 4291 (3698,5022) | 3474 (2887,4189) | 3786 (3330,4324) |
| <12 | 5282 (4176,6591) | 5624 (4627,6817) | 6760 (5773,7841) | 4454 (2863,6099) | 2917 (1826,4499) | 7098 (5943,8318) | 6636 (5655,7680) | 5359 (4374,6577) | 5868 (5083,6742) |
| 12-14 | 3654 (2865,4634) | 3896 (3190,4702) | 4664 (3923,5555) | 3047 (2076,4168) | 2054 (1290,3138) | 4907 (4153,5744) | 4562 (3910,5308) | 3696 (3011,4480) | 4054 (3554,4678) |
| 15-17 | 2722 (2093,3503) | 2866 (2392,3485) | 3460 (2906,4134) | 2276 (1487,3101) | 1484 (928,2345) | 3657 (3082,4292) | 3385 (2914,3904) | 2740 (2264,3300) | 3021 (2641,3467) |
| 18-20 | 2995 (2347,3768) | 3172 (2609,3798) | 3821 (3227,4500) | 2505 (1703,3451) | 1663 (1027,2630) | 4009 (3370,4739) | 3706 (3186,4315) | 3013 (2475,3644) | 3301 (2918,3810) |
| 21-23 | 1625 (1257,2024) | 1714 (1389,2079) | 2052 (1730,2435) | 1345 (904,1854) | 903 (552,1379) | 2170 (1797,2567) | 2018 (1706,2318) | 1632 (1354,1993) | 1786 (1563,2054) |
| 12-23 | 2800 (2189,3582) | 2936 (2421,3510) | 3537 (2977,4138) | 2290 (1507,3150) | 1550 (943,2391) | 3697 (3137,4357) | 3453 (2995,4023) | 2793 (2296,3419) | 3058 (2662,3487) |
| <24 | 4046 (3144,5166) | 4268 (3558,5086) | 5138 (4356,6097) | 3310 (2295,4645) | 2240 (1371,3354) | 5400 (4542,6389) | 5014 (4308,5837) | 4105 (3322,4918) | 4469 (3906,5120) |
| 24-35 | 1264 (971,1587) | 1325 (1103,1584) | 1602 (1341,1884) | 1038 (708,1445) | 692 (431,1101) | 1686 (1428,2004) | 1566 (1350,1824) | 1265 (1041,1544) | 1389 (1209,1607) |
| 36-47 | 1040 (802,1302) | 1088 (886,1309) | 1308 (1108,1537) | 863 (572,1193) | 583 (359,888) | 1384 (1153,1648) | 1283 (1099,1486) | 1050 (851,1255) | 1145 (996,1311) |
| 48-59 | 518 (403,655) | 551 (453,654) | 658 (558,772) | 433 (287,593) | 289 (181,455) | 696 (587,815) | 645 (557,744) | 523 (432,639) | 574 (503,664) |
| 24-59 | 923 (729,1176) | 988 (807,1202) | 1179 (986,1383) | 771 (503,1051) | 511 (307,787) | 1240 (1060,1468) | 1152 (993,1335) | 934 (766,1147) | 1027 (900,1183) |
| <60 | 2194 (1730,2771) | 2325 (1898,2820) | 2784 (2367,3317) | 1815 (1225,2497) | 1228 (795,1898) | 2955 (2464,3470) | 2731 (2360,3171) | 2208 (1836,2681) | 2436 (2109,2799) |
| 2016 |  |  |  |  |  |  |  |  |  |
| <1 | 6751 (5302,8517) | 7156 (5912,8628) | 8563 (7188,10166) | 5529 (3706,7798) | 3654 (2235,5664) | 9017 (7601,10664) | 8390 (7198,9746) | 6811 (5534,8306) | 7441 (6473,8463) |
| 1 | 11698 (9095,14589) | 12412 (10094,14955) | 14881 (12547,17711) | 9671 (6366,13392) | 6514 (4082,10095) | 15640 (13126,18462) | 14497 (12454,16799) | 11829 (9666,14396) | 12860 (11179,14747) |
| 2 | 8099 (6291,10322) | 8603 (7109,10386) | 10321 (8772,12308) | 6751 (4437,9257) | 4522 (2835,6988) | 10893 (9192,12889) | 10086 (8657,11617) | 8213 (6695,9899) | 8958 (7812,10305) |
| 3 | 6913 (5477,8617) | 7341 (6183,8764) | 8788 (7471,10466) | 5788 (4016,7808) | 3850 (2407,5991) | 9311 (7806,11030) | 8636 (7477,10074) | 6961 (5695,8528) | 7672 (6732,8755) |
| 4 | 3402 (2626,4278) | 3622 (2991,4401) | 4337 (3684,5162) | 2843 (1867,3846) | 1893 (1192,2912) | 4597 (3883,5427) | 4268 (3633,4972) | 3467 (2846,4208) | 3778 (3282,4314) |
| 5 | 5907 (4545,7492) | 6279 (5175,7528) | 7512 (6246,8892) | 4916 (3348,6795) | 3336 (1954,5187) | 7931 (6600,9334) | 7368 (6320,8542) | 6010 (4883,7190) | 6556 (5741,7509) |
| 6 | 5096 (4005,6449) | 5429 (4541,6539) | 6533 (5533,7742) | 4311 (2855,5866) | 2856 (1772,4388) | 6895 (5871,8186) | 6442 (5517,7477) | 5198 (4250,6336) | 5705 (5015,6551) |
| 7 | 4032 (3156,5071) | 4282 (3611,5191) | 5159 (4369,6076) | 3409 (2257,4590) | 2281 (1372,3515) | 5449 (4548,6457) | 5062 (4347,5902) | 4106 (3417,5047) | 4498 (3929,5137) |
| 8 | 5944 (4709,7558) | 6351 (5252,7753) | 7626 (6367,9138) | 4977 (3316,6999) | 3273 (2010,5122) | 8008 (6705,9460) | 7431 (6453,8674) | 6019 (4913,7381) | 6625 (5772,7613) |
| 9 | 3519 (2792,4432) | 3712 (3044,4482) | 4469 (3725,5302) | 2932 (1926,4021) | 1967 (1194,3031) | 4681 (3947,5523) | 4365 (3796,5065) | 3541 (2918,4293) | 3886 (3388,4499) |
| 10 | 2870 (2245,3550) | 3016 (2502,3623) | 3659 (3092,4306) | 2377 (1601,3294) | 1608 (974,2515) | 3849 (3248,4559) | 3591 (3094,4116) | 2904 (2338,3537) | 3165 (2770,3632) |
| 11 | 1400 (1087,1747) | 1477 (1193,1743) | 1770 (1489,2100) | 1159 (792,1584) | 767 (489,1188) | 1856 (1549,2203) | 1723 (1485,2025) | 1401 (1144,1689) | 1541 (1331,1784) |
| <12 | 5494 (4336,6896) | 5824 (4838,6997) | 6980 (5833,8226) | 4646 (3034,6347) | 3065 (1864,4673) | 7376 (6212,8726) | 6846 (5938,7939) | 5525 (4519,6712) | 6084 (5294,7006) |
| 12-14 | 2547 (2003,3206) | 2716 (2223,3280) | 3277 (2764,3837) | 2114 (1444,2857) | 1466 (923,2246) | 3482 (2920,4075) | 3218 (2777,3732) | 2604 (2171,3130) | 2826 (2484,3246) |
| 15-17 | 3078 (2409,3897) | 3261 (2719,3916) | 3919 (3301,4660) | 2571 (1774,3632) | 1692 (1041,2624) | 4139 (3511,4883) | 3851 (3311,4418) | 3107 (2545,3750) | 3408 (3004,3927) |
| 18-20 | 1810 (1394,2304) | 1897 (1570,2275) | 2279 (1936,2672) | 1495 (1007,2044) | 1010 (609,1577) | 2407 (2026,2870) | 2246 (1949,2573) | 1824 (1498,2219) | 1996 (1728,2263) |
| 21-23 | 2306 (1830,2934) | 2449 (2026,2960) | 2940 (2476,3459) | 1928 (1280,2682) | 1306 (808,1992) | 3093 (2637,3636) | 2866 (2462,3332) | 2342 (1891,2859) | 2558 (2225,2937) |
| 12-23 | 2436 (1912,3054) | 2582 (2152,3053) | 3095 (2606,3653) | 2027 (1388,2840) | 1353 (851,2095) | 3254 (2728,3882) | 3033 (2622,3532) | 2468 (2009,2978) | 2697 (2347,3092) |
| <24 | 3941 (3140,4907) | 4142 (3425,4963) | 5025 (4222,5895) | 3297 (2155,4653) | 2151 (1348,3413) | 5251 (4432,6185) | 4899 (4243,5646) | 3953 (3268,4836) | 4347 (3802,5012) |
| 24-35 | 1199 (928,1512) | 1260 (1045,1518) | 1525 (1286,1809) | 998 (646,1339) | 665 (406,1018) | 1610 (1381,1910) | 1489 (1272,1741) | 1196 (989,1463) | 1322 (1163,1507) |
| 36-47 | 766 (606,970) | 818 (667,995) | 982 (832,1159) | 639 (431,888) | 425 (263,689) | 1036 (870,1230) | 962 (836,1116) | 784 (636,952) | 854 (749,980) |
| 48-59 | 506 (397,634) | 537 (445,646) | 648 (546,769) | 423 (281,586) | 282 (169,440) | 680 (570,812) | 629 (548,731) | 513 (424,632) | 562 (494,651) |
| 24-59 | 832 (648,1032) | 876 (733,1069) | 1055 (899,1262) | 694 (461,975) | 458 (286,712) | 1114 (928,1331) | 1028 (886,1202) | 839 (692,1031) | 919 (805,1050) |
| <60 | 2053 (1606,2590) | 2185 (1795,2611) | 2614 (2206,3115) | 1690 (1155,2355) | 1149 (706,1781) | 2742 (2291,3213) | 2556 (2195,2957) | 2083 (1710,2507) | 2269 (1988,2620) |
| 2017 |  |  |  |  |  |  |  |  |  |
| <1 | 4715 (3719,5949) | 4967 (4125,5919) | 5998 (5075,7085) | 3912 (2619,5336) | 2619 (1604,4168) | 6352 (5281,7529) | 5884 (5074,6816) | 4787 (3904,5847) | 5232 (4603,6039) |
| 1 | 5880 (4552,7502) | 6309 (5222,7545) | 7566 (6325,8954) | 4922 (3320,6796) | 3334 (1991,5140) | 7936 (6612,9466) | 7361 (6409,8481) | 5966 (4951,7209) | 6571 (5691,7462) |
| 2 | 3405 (2629,4296) | 3574 (2985,4229) | 4304 (3638,5102) | 2823 (1945,3912) | 1895 (1143,2944) | 4536 (3827,5363) | 4211 (3637,4902) | 3409 (2793,4114) | 3756 (3281,4296) |
| 3 | 4113 (3237,5185) | 4345 (3641,5203) | 5221 (4462,6126) | 3466 (2319,4738) | 2293 (1400,3613) | 5543 (4685,6519) | 5110 (4339,5953) | 4194 (3414,5060) | 4559 (3963,5270) |
| 4 | 3156 (2474,3959) | 3351 (2747,4051) | 4027 (3423,4772) | 2644 (1741,3672) | 1764 (1124,2691) | 4265 (3550,5021) | 3961 (3438,4586) | 3193 (2618,3886) | 3505 (3072,4028) |
| 5 | 2290 (1746,2857) | 2428 (2026,2884) | 2925 (2444,3459) | 1900 (1312,2633) | 1271 (782,1983) | 3062 (2584,3583) | 2865 (2487,3317) | 2327 (1883,2812) | 2533 (2221,2910) |
| 6 | 2037 (1568,2570) | 2159 (1802,2596) | 2593 (2176,3058) | 1707 (1162,2322) | 1113 (696,1790) | 2740 (2295,3253) | 2551 (2175,2913) | 2041 (1680,2487) | 2254 (1984,2605) |
| 7 | 2355 (1858,2960) | 2493 (2042,2991) | 2991 (2511,3518) | 1936 (1296,2688) | 1295 (786,1989) | 3145 (2641,3711) | 2916 (2500,3418) | 2375 (1936,2907) | 2596 (2275,3013) |
| 8 | 2065 (1619,2623) | 2188 (1788,2660) | 2638 (2223,3105) | 1708 (1153,2346) | 1151 (741,1745) | 2791 (2295,3309) | 2585 (2220,3015) | 2098 (1730,2537) | 2292 (1997,2639) |
| 9 | 2279 (1794,2868) | 2420 (2005,2909) | 2882 (2434,3441) | 1906 (1308,2657) | 1249 (787,1926) | 3052 (2583,3569) | 2844 (2453,3335) | 2277 (1876,2778) | 2516 (2223,2879) |
| 10 | 2010 (1571,2489) | 2146 (1774,2581) | 2580 (2151,3035) | 1689 (1120,2302) | 1112 (675,1754) | 2708 (2274,3160) | 2523 (2154,2909) | 2039 (1670,2497) | 2249 (1955,2570) |
| 11 | 2001 (1548,2514) | 2103 (1745,2554) | 2539 (2131,3018) | 1660 (1113,2280) | 1092 (671,1715) | 2694 (2241,3159) | 2499 (2144,2906) | 2018 (1665,2487) | 2204 (1929,2527) |
| <12 | 3115 (2432,4000) | 3285 (2730,3936) | 3969 (3308,4734) | 2565 (1720,3597) | 1721 (1087,2704) | 4166 (3499,4924) | 3867 (3358,4531) | 3129 (2558,3717) | 3454 (2974,3936) |
| 12-14 | 1589 (1222,2014) | 1661 (1378,1982) | 2017 (1690,2379) | 1311 (910,1828) | 885 (551,1333) | 2119 (1806,2490) | 1974 (1707,2280) | 1607 (1311,1909) | 1748 (1533,2003) |
| 15-17 | 1177 (928,1486) | 1245 (1026,1487) | 1498 (1273,1771) | 979 (664,1336) | 650 (405,1012) | 1575 (1317,1866) | 1476 (1262,1709) | 1181 (976,1451) | 1309 (1134,1495) |
| 18-20 | 946 (731,1166) | 998 (820,1199) | 1194 (1014,1401) | 787 (521,1076) | 522 (313,805) | 1268 (1069,1504) | 1173 (1015,1365) | 953 (787,1145) | 1044 (904,1204) |
| 21-23 | 775 (608,986) | 822 (692,998) | 992 (824,1168) | 644 (439,877) | 427 (266,664) | 1047 (887,1230) | 973 (841,1137) | 790 (635,961) | 863 (753,985) |
| 12-23 | 1115 (873,1399) | 1196 (1002,1424) | 1425 (1196,1679) | 924 (637,1302) | 618 (367,959) | 1495 (1278,1782) | 1388 (1211,1615) | 1141 (924,1371) | 1237 (1079,1420) |
| <24 | 2099 (1667,2641) | 2250 (1865,2701) | 2681 (2271,3195) | 1762 (1172,2466) | 1182 (724,1779) | 2846 (2397,3351) | 2641 (2290,3048) | 2141 (1745,2596) | 2333 (2054,2703) |
| 24-35 | 640 (506,801) | 676 (564,802) | 814 (681,956) | 536 (363,741) | 356 (225,563) | 853 (723,1009) | 795 (688,935) | 650 (539,783) | 706 (616,812) |
| 36-47 | 402 (316,498) | 422 (354,504) | 507 (423,596) | 333 (221,471) | 221 (138,341) | 535 (452,633) | 501 (430,573) | 405 (331,491) | 443 (381,509) |
| 48-59 | 237 (184,309) | 253 (207,303) | 302 (256,359) | 196 (133,272) | 131 (80,208) | 319 (268,377) | 295 (255,346) | 240 (195,288) | 262 (227,297) |
| 24-59 | 424 (335,539) | 454 (370,538) | 546 (462,644) | 354 (246,479) | 234 (145,377) | 571 (486,684) | 532 (454,621) | 434 (352,521) | 473 (415,541) |
| <60 | 1106 (847,1395) | 1166 (964,1406) | 1398 (1175,1629) | 913 (604,1290) | 608 (387,980) | 1464 (1250,1727) | 1368 (1184,1575) | 1112 (914,1345) | 1208 (1063,1379) |
| 2018 |  |  |  |  |  |  |  |  |  |
| <1 | 11966 (9458,14739) | 12709 (10540,15371) | 15306 (12836,17981) | 10064 (6538,13886) | 6672 (4099,10220) | 16017 (13386,19090) | 14897 (12804,17198) | 12078 (9893,14647) | 13340 (11632,15279) |
| 1 | 9756 (7798,12387) | 10376 (8628,12413) | 12425 (10551,14858) | 8132 (5487,11049) | 5441 (3305,8524) | 13002 (11055,15722) | 12248 (10508,14185) | 9963 (8138,12067) | 10882 (9474,12489) |
| 2 | 5916 (4696,7453) | 6292 (5228,7588) | 7572 (6373,8959) | 4926 (3233,6739) | 3283 (1975,5234) | 7983 (6857,9385) | 7332 (6296,8599) | 5984 (4911,7330) | 6577 (5742,7507) |
| 3 | 6595 (5208,8229) | 6956 (5788,8340) | 8378 (6967,9865) | 5397 (3638,7400) | 3664 (2243,5549) | 8779 (7432,10443) | 8176 (7116,9515) | 6585 (5356,7930) | 7221 (6276,8289) |
| 4 | 7474 (5901,9410) | 7889 (6521,9431) | 9475 (7909,11170) | 6155 (4124,8414) | 4098 (2491,6308) | 9948 (8322,11972) | 9295 (7933,10723) | 7521 (6058,9211) | 8218 (7208,9498) |
| 5 | 5798 (4540,7302) | 6124 (5066,7378) | 7439 (6246,8688) | 4820 (3329,6645) | 3226 (1854,4963) | 7812 (6601,9236) | 7269 (6241,8533) | 5832 (4775,7127) | 6449 (5633,7378) |
| 6 | 5441 (4350,6778) | 5750 (4792,6851) | 6966 (5854,8223) | 4580 (3125,6211) | 3007 (1881,4677) | 7297 (6133,8680) | 6791 (5801,7831) | 5498 (4491,6706) | 6048 (5252,6992) |
| 7 | 4408 (3461,5505) | 4626 (3850,5602) | 5602 (4637,6644) | 3693 (2534,5052) | 2460 (1491,3709) | 5887 (4951,6978) | 5497 (4675,6423) | 4463 (3618,5392) | 4870 (4247,5596) |
| 8 | 6254 (4911,7779) | 6660 (5507,7921) | 8006 (6699,9509) | 5238 (3629,7166) | 3483 (2094,5287) | 8393 (7117,9873) | 7810 (6707,9034) | 6343 (5142,7702) | 6951 (6086,7995) |
| 9 | 3953 (3135,5044) | 4187 (3478,5057) | 5065 (4207,6009) | 3318 (2214,4533) | 2197 (1397,3501) | 5352 (4428,6273) | 4971 (4250,5731) | 4014 (3275,4832) | 4372 (3817,5038) |
| 10 | 4372 (3353,5424) | 4617 (3828,5596) | 5598 (4692,6506) | 3668 (2450,4980) | 2406 (1508,3743) | 5840 (4915,6927) | 5451 (4750,6329) | 4448 (3632,5343) | 4835 (4207,5544) |
| 11 | 3282 (2539,4116) | 3455 (2841,4148) | 4129 (3461,4925) | 2722 (1802,3774) | 1810 (1106,2863) | 4386 (3727,5180) | 4060 (3470,4775) | 3267 (2697,4035) | 3623 (3173,4134) |
| <12 | 6101 (4751,7706) | 6494 (5299,7844) | 7771 (6565,9244) | 5103 (3482,6896) | 3349 (2011,5206) | 8189 (6947,9568) | 7632 (6497,8922) | 6148 (5091,7565) | 6799 (5900,7772) |
| 12-14 | 2585 (2011,3277) | 2744 (2233,3294) | 3268 (2778,3912) | 2158 (1428,2940) | 1449 (879,2243) | 3489 (2930,4135) | 3235 (2773,3740) | 2616 (2152,3161) | 2872 (2510,3267) |
| 15-17 | 2158 (1678,2722) | 2299 (1887,2751) | 2765 (2352,3288) | 1832 (1248,2491) | 1209 (730,1801) | 2915 (2417,3476) | 2702 (2350,3133) | 2200 (1807,2698) | 2408 (2102,2776) |
| 18-20 | 2698 (2085,3417) | 2867 (2353,3443) | 3453 (2920,4078) | 2228 (1519,3155) | 1486 (930,2314) | 3628 (3010,4267) | 3366 (2918,3881) | 2739 (2255,3277) | 2992 (2618,3407) |
| 21-23 | 1868 (1466,2320) | 1966 (1610,2384) | 2390 (2014,2820) | 1562 (1024,2129) | 1057 (628,1603) | 2499 (2112,2941) | 2335 (2021,2707) | 1884 (1548,2286) | 2071 (1797,2361) |
| 12-23 | 2339 (1843,2934) | 2489 (2065,2972) | 2976 (2516,3522) | 1957 (1344,2676) | 1304 (796,2005) | 3147 (2651,3746) | 2920 (2501,3406) | 2364 (1945,2899) | 2603 (2229,3014) |
| <24 | 4174 (3331,5275) | 4389 (3615,5242) | 5332 (4502,6289) | 3459 (2352,4706) | 2329 (1444,3482) | 5585 (4760,6502) | 5217 (4452,6074) | 4199 (3457,5059) | 4616 (4023,5385) |
| 24-35 | 1534 (1211,1947) | 1627 (1344,1968) | 1965 (1687,2328) | 1305 (886,1785) | 861 (553,1386) | 2080 (1736,2462) | 1944 (1675,2260) | 1555 (1265,1879) | 1711 (1478,1964) |
| 36-47 | 868 (682,1101) | 914 (756,1104) | 1099 (920,1292) | 718 (477,989) | 478 (306,729) | 1163 (984,1376) | 1079 (932,1252) | 876 (704,1072) | 956 (834,1090) |
| 48-59 | 572 (463,722) | 604 (507,733) | 729 (611,865) | 482 (315,650) | 320 (203,512) | 769 (645,903) | 714 (618,826) | 581 (470,713) | 636 (555,728) |
| 24-59 | 993 (776,1238) | 1053 (864,1263) | 1270 (1070,1511) | 828 (548,1144) | 550 (322,874) | 1332 (1123,1570) | 1239 (1070,1443) | 1007 (821,1227) | 1100 (964,1267) |
| <60 | 2238 (1762,2849) | 2361 (1941,2859) | 2835 (2394,3354) | 1862 (1233,2542) | 1227 (789,1924) | 2984 (2536,3509) | 2762 (2398,3274) | 2269 (1841,2757) | 2468 (2157,2807) |

**Table S7: Annual rates of RSV associated-hospitalized severe acute respiratory illness per 100,000 children by regions and age in months, 2010-2018**

| Year and Age in months | Central Rate (95% CI) | Coast Rate (95% CI) | Eastern Rate (95% CI) | North Eastern Rate (95% CI) | Nairobi Rate (95% CI) | Nyanza Rate (95% CI) | Rift Valley Rate (95% CI) | Western Rate (95% CI) | Kenya Rate (95% CI) |
| --- | --- | --- | --- | --- | --- | --- | --- | --- | --- |
| 2010-2018 |  |  |  |  |  |  |  |  |  |
| <1 | 1377 (906,2007) | 2033 (1602,2587) | 2732 (2235,3252) | 1779 (1186,2518) | 769 (439,1337) | 2116 (1510,2803) | 1724 (1192,2422) | 1668 (1226,2252) | 2366 (2042,2751) |
| 1 | 2008 (1311,2968) | 3989 (3171,4928) | 3936 (3253,4694) | 2592 (1678,3651) | 1099 (613,1927) | 3046 (2174,4136) | 2505 (1724,3456) | 2421 (1797,3177) | 3420 (2911,4012) |
| 2 | 1463 (950,2087) | 2623 (2128,3281) | 2895 (2395,3465) | 1904 (1256,2645) | 785 (444,1372) | 2213 (1572,2913) | 1801 (1249,2503) | 1754 (1242,2383) | 2493 (2142,2912) |
| 3 | 867 (599,1209) | 1984 (1557,2524) | 1738 (1432,2113) | 1133 (759,1591) | 485 (284,800) | 1264 (937,1656) | 1084 (811,1433) | 1028 (755,1328) | 1516 (1297,1790) |
| 4 | 786 (541,1099) | 1660 (1272,2137) | 1579 (1309,1908) | 1049 (689,1432) | 436 (251,742) | 1160 (870,1500) | 980 (692,1313) | 913 (673,1241) | 1372 (1169,1597) |
| 5 | 861 (577,1222) | 1713 (1290,2244) | 1747 (1441,2088) | 1132 (751,1600) | 474 (277,798) | 1263 (948,1656) | 1077 (786,1432) | 1022 (742,1367) | 1510 (1288,1766) |
| 6 | 715 (488,990) | 1304 (979,1726) | 1215 (999,1482) | 804 (519,1129) | 395 (225,667) | 1213 (926,1597) | 892 (644,1188) | 973 (717,1256) | 1052 (877,1269) |
| 7 | 616 (421,862) | 1027 (749,1410) | 1039 (858,1275) | 677 (459,965) | 346 (192,571) | 1037 (784,1376) | 769 (541,1028) | 826 (625,1095) | 916 (764,1086) |
| 8 | 577 (382,785) | 727 (516,1037) | 981 (801,1207) | 643 (439,908) | 315 (180,532) | 979 (728,1285) | 719 (518,952) | 780 (581,1019) | 853 (714,1023) |
| 9 | 404 (272,579) | 530 (355,763) | 592 (467,729) | 382 (256,543) | 223 (121,405) | 611 (436,841) | 522 (350,719) | 474 (332,649) | 513 (417,618) |
| 10 | 456 (294,688) | 562 (373,803) | 652 (529,819) | 429 (282,611) | 250 (134,437) | 690 (500,919) | 567 (370,796) | 524 (363,709) | 575 (475,685) |
| 11 | 371 (229,540) | 411 (262,613) | 539 (426,669) | 352 (234,501) | 203 (113,342) | 566 (394,758) | 470 (323,654) | 436 (306,589) | 465 (389,567) |
| <12 | 752 (576,992) | 1585 (1292,1907) | 1504 (1248,1797) | 982 (656,1375) | 423 (262,671) | 1185 (955,1464) | 953 (764,1158) | 928 (733,1171) | 1303 (1122,1507) |
| 12-14 | 249 (142,380) | 354 (262,469) | 438 (346,556) | 285 (190,421) | 136 (72,249) | 573 (393,775) | 310 (192,453) | 412 (276,583) | 383 (310,466) |
| 15-17 | 204 (96,359) | 234 (165,330) | 329 (251,425) | 216 (142,319) | 113 (48,229) | 420 (281,604) | 255 (111,450) | 319 (215,462) | 285 (223,362) |
| 18-20 | 334 (191,541) | 241 (166,332) | 366 (281,473) | 241 (151,355) | 183 (96,342) | 411 (263,593) | 423 (241,646) | 332 (217,480) | 318 (249,399) |
| 21-23 | 318 (162,519) | 111 (65,172) | 210 (152,285) | 139 (81,206) | 172 (78,330) | 248 (140,377) | 394 (211,609) | 178 (95,274) | 184 (133,238) |
| 12-23 | 261 (186,363) | 237 (190,294) | 339 (275,409) | 223 (147,312) | 146 (84,237) | 415 (323,527) | 322 (237,424) | 311 (244,402) | 294 (248,351) |
| <24 | 505 (384,652) | 907 (747,1121) | 906 (760,1081) | 595 (399,818) | 284 (176,437) | 777 (637,945) | 638 (517,772) | 608 (489,758) | 790 (686,909) |
| 24-35 | 135 (84,198) | 87 (63,118) | 139 (110,174) | 92 (60,130) | 76 (40,132) | 187 (138,250) | 170 (110,243) | 132 (96,182) | 120 (99,148) |
| 36-47 | 68 (33,118) | 33 (20,51) | 66 (49,89) | 43 (28,63) | 37 (15,73) | 111 (73,162) | 85 (43,140) | 73 (48,105) | 58 (43,75) |
| 48-59 | 9 (0,25) | 4 (0,10) | 15 (8,23) | 10 (5,16) | 5 (0,15) | 30 (13,51) | 13 (0,31) | 21 (11,35) | 13 (7,21) |
| 24-59 | 69 (46,97) | 42 (31,56) | 73 (58,89) | 47 (32,67) | 37 (22,65) | 108 (84,138) | 86 (59,116) | 75 (57,98) | 63 (52,77) |
| <60 | 246 (188,324) | 389 (318,473) | 400 (338,474) | 264 (175,364) | 138 (84,214) | 371 (304,446) | 304 (249,365) | 275 (224,345) | 349 (303,404) |
| 2010 |  |  |  |  |  |  |  |  |  |
| <1 | 2588 (1749,3790) | 4740 (3589,6175) | 5140 (4323,6188) | 3377 (2241,4713) | 1464 (806,2547) | 4255 (2764,6285) | 3279 (2206,4523) | 3125 (2251,4286) | 4465 (3888,5214) |
| 1 | 2620 (1762,3778) | 4735 (3666,6008) | 5152 (4301,6088) | 3391 (2195,4746) | 1434 (769,2375) | 4382 (2662,6333) | 3296 (2271,4540) | 3133 (2223,4287) | 4465 (3823,5230) |
| 2 | 2193 (1470,3184) | 3967 (3096,5052) | 4271 (3555,5182) | 2859 (1819,3999) | 1195 (646,2083) | 3633 (2368,5142) | 2776 (1890,3837) | 2628 (1890,3592) | 3760 (3200,4390) |
| 3 | 1262 (864,1762) | 2621 (1853,3641) | 2564 (2107,3089) | 1675 (1109,2322) | 707 (416,1142) | 1923 (1280,2753) | 1576 (1158,2107) | 1481 (1118,1961) | 2217 (1869,2607) |
| 4 | 951 (661,1322) | 1964 (1394,2679) | 1909 (1588,2306) | 1267 (844,1736) | 531 (305,856) | 1450 (933,2064) | 1193 (868,1605) | 1125 (827,1501) | 1665 (1408,1979) |
| 5 | 1261 (862,1728) | 2578 (1819,3480) | 2516 (2082,3032) | 1653 (1136,2337) | 694 (412,1134) | 1890 (1177,2707) | 1568 (1159,2026) | 1486 (1079,2012) | 2191 (1865,2576) |
| 6 | 1278 (861,1765) | 1802 (1134,2648) | 2139 (1733,2581) | 1380 (881,1948) | 691 (392,1185) | 1685 (979,2579) | 1577 (1120,2199) | 1685 (1260,2213) | 1852 (1556,2222) |
| 7 | 1154 (786,1611) | 1647 (1042,2476) | 1968 (1605,2357) | 1287 (861,1790) | 628 (351,1053) | 1593 (918,2466) | 1442 (1064,1965) | 1560 (1164,2090) | 1692 (1436,2021) |
| 8 | 722 (499,1039) | 1045 (647,1514) | 1220 (1008,1465) | 799 (527,1099) | 397 (234,654) | 979 (560,1450) | 902 (643,1213) | 964 (722,1286) | 1067 (881,1291) |
| 9 | 831 (531,1239) | 886 (528,1347) | 1194 (954,1491) | 782 (524,1121) | 457 (250,786) | 1246 (636,2050) | 1036 (708,1495) | 949 (677,1322) | 1030 (841,1248) |
| 10 | 962 (617,1385) | 1024 (580,1635) | 1374 (1101,1737) | 908 (606,1284) | 514 (300,937) | 1466 (727,2335) | 1191 (785,1708) | 1116 (799,1513) | 1199 (992,1467) |
| 11 | 788 (503,1174) | 843 (469,1270) | 1149 (924,1419) | 750 (501,1046) | 433 (239,716) | 1203 (595,1913) | 986 (646,1380) | 928 (646,1249) | 995 (808,1201) |
| <12 | 1227 (902,1606) | 2406 (1919,3027) | 2407 (2026,2896) | 1577 (1068,2181) | 678 (400,1095) | 1912 (1454,2467) | 1531 (1238,1843) | 1481 (1180,1856) | 2082 (1793,2428) |
| 12-14 | 419 (250,625) | 727 (382,1166) | 716 (577,897) | 469 (310,679) | 226 (120,409) | 991 (478,1640) | 516 (313,758) | 692 (488,950) | 628 (503,772) |
| 15-17 | 360 (140,629) | 668 (342,1018) | 556 (414,716) | 362 (230,533) | 190 (69,375) | 559 (191,1107) | 435 (196,741) | 541 (356,799) | 485 (373,624) |
| 18-20 | 532 (290,853) | 500 (197,854) | 575 (437,741) | 376 (242,544) | 288 (135,546) | 1091 (507,1811) | 658 (366,1016) | 521 (342,753) | 502 (383,629) |
| 21-23 | 404 (210,672) | 194 (45,412) | 269 (196,359) | 177 (109,265) | 219 (106,403) | 275 (67,544) | 494 (269,798) | 229 (130,342) | 235 (172,310) |
| 12-23 | 407 (285,556) | 521 (374,712) | 530 (444,640) | 353 (235,482) | 223 (131,378) | 720 (485,993) | 513 (384,656) | 490 (367,629) | 462 (388,546) |
| <24 | 782 (594,1012) | 1403 (1124,1759) | 1415 (1168,1685) | 919 (588,1284) | 435 (272,688) | 1279 (1004,1601) | 981 (802,1177) | 940 (760,1183) | 1221 (1066,1408) |
| 24-35 | 210 (138,319) | 126 (53,219) | 216 (173,267) | 141 (91,196) | 118 (63,205) | 238 (103,407) | 265 (173,378) | 207 (152,283) | 188 (153,231) |
| 36-47 | 121 (58,212) | 40 (0,97) | 119 (86,158) | 77 (48,113) | 66 (28,127) | 121 (29,258) | 150 (71,246) | 130 (84,193) | 104 (78,134) |
| 48-59 | 16 (0,39) | 13 (0,44) | 26 (14,40) | 17 (9,29) | 9 (0,24) | 83 (0,195) | 22 (0,50) | 37 (17,62) | 22 (13,34) |
| 24-59 | 114 (77,162) | 60 (32,99) | 120 (98,151) | 80 (52,111) | 63 (35,106) | 150 (86,243) | 142 (102,192) | 125 (95,159) | 105 (86,125) |
| <60 | 384 (286,502) | 590 (473,733) | 629 (523,746) | 412 (277,572) | 216 (131,336) | 616 (481,786) | 477 (399,579) | 437 (349,535) | 547 (475,640) |
| 2011 |  |  |  |  |  |  |  |  |  |
| <1 | 1860 (1218,2643) | 4150 (3184,5289) | 3674 (3043,4461) | 2399 (1503,3301) | 1046 (592,1795) | 1856 (657,3580) | 2354 (1599,3245) | 2245 (1595,2994) | 3193 (2740,3717) |
| 1 | 2103 (1387,3064) | 4618 (3617,5836) | 4104 (3431,4935) | 2688 (1772,3704) | 1176 (663,1960) | 2097 (690,4059) | 2612 (1782,3673) | 2505 (1813,3353) | 3565 (3071,4218) |
| 2 | 1563 (1002,2243) | 3434 (2652,4332) | 3065 (2562,3704) | 2013 (1365,2789) | 842 (470,1451) | 1523 (523,3015) | 1962 (1362,2721) | 1886 (1317,2542) | 2654 (2269,3108) |
| 3 | 1320 (901,1845) | 3258 (2490,4285) | 2641 (2192,3223) | 1720 (1155,2457) | 730 (433,1237) | 1601 (685,2727) | 1662 (1196,2167) | 1543 (1142,2003) | 2301 (1955,2747) |
| 4 | 1042 (715,1455) | 2610 (1849,3386) | 2096 (1711,2529) | 1366 (899,1889) | 567 (325,925) | 1246 (521,2148) | 1298 (968,1714) | 1217 (902,1603) | 1802 (1538,2135) |
| 5 | 1006 (693,1380) | 2471 (1861,3271) | 2007 (1637,2415) | 1304 (880,1824) | 546 (319,912) | 1188 (524,2013) | 1253 (904,1693) | 1175 (849,1533) | 1738 (1487,2051) |
| 6 | 984 (680,1395) | 1875 (1252,2686) | 1659 (1357,2019) | 1090 (735,1499) | 535 (317,914) | 1412 (667,2375) | 1216 (871,1683) | 1314 (994,1749) | 1460 (1220,1728) |
| 7 | 1076 (736,1488) | 2048 (1338,2890) | 1835 (1484,2250) | 1195 (773,1693) | 598 (328,1008) | 1588 (725,2554) | 1353 (966,1775) | 1438 (1084,1884) | 1588 (1321,1882) |
| 8 | 692 (457,958) | 1319 (856,1839) | 1168 (946,1415) | 753 (494,1098) | 379 (211,633) | 1008 (457,1657) | 855 (622,1153) | 926 (691,1230) | 1006 (857,1198) |
| 9 | 419 (273,611) | 727 (429,1119) | 606 (493,754) | 398 (264,569) | 233 (131,409) | 552 (252,922) | 526 (353,746) | 496 (344,665) | 532 (435,635) |
| 10 | 487 (317,729) | 832 (488,1238) | 703 (552,868) | 466 (303,643) | 266 (143,464) | 636 (289,1087) | 610 (414,840) | 561 (392,759) | 616 (504,747) |
| 11 | 549 (347,816) | 924 (531,1410) | 779 (620,978) | 511 (325,728) | 291 (159,533) | 713 (298,1231) | 678 (458,960) | 629 (437,853) | 684 (564,831) |
| <12 | 955 (710,1253) | 2410 (1925,3030) | 1895 (1577,2257) | 1222 (812,1686) | 524 (320,848) | 1186 (824,1664) | 1198 (964,1461) | 1170 (928,1460) | 1635 (1425,1882) |
| 12-14 | 275 (164,421) | 474 (219,830) | 480 (374,596) | 311 (204,447) | 148 (81,262) | 459 (163,847) | 339 (218,491) | 458 (328,637) | 418 (336,513) |
| 15-17 | 226 (95,400) | 339 (114,637) | 356 (273,454) | 234 (143,336) | 127 (52,240) | 462 (177,834) | 283 (134,483) | 353 (235,501) | 313 (240,392) |
| 18-20 | 531 (289,874) | 389 (122,750) | 589 (451,766) | 382 (238,572) | 290 (139,542) | 593 (128,1217) | 659 (378,1056) | 527 (350,752) | 508 (396,645) |
| 21-23 | 443 (223,745) | 212 (51,476) | 300 (216,400) | 195 (117,293) | 238 (109,457) | 621 (214,1210) | 558 (287,879) | 250 (141,392) | 260 (190,349) |
| 12-23 | 326 (230,454) | 366 (235,538) | 432 (353,515) | 281 (191,394) | 182 (105,296) | 524 (333,770) | 410 (307,531) | 395 (306,507) | 375 (314,444) |
| <24 | 652 (489,846) | 1434 (1151,1785) | 1160 (970,1392) | 757 (518,1036) | 354 (221,571) | 877 (654,1172) | 814 (669,980) | 785 (623,974) | 1010 (869,1163) |
| 24-35 | 160 (97,238) | 164 (82,267) | 165 (129,205) | 108 (72,156) | 88 (47,157) | 209 (88,355) | 201 (133,288) | 157 (115,212) | 144 (117,176) |
| 36-47 | 82 (41,136) | 50 (12,108) | 80 (59,106) | 53 (34,79) | 45 (21,88) | 203 (90,350) | 103 (50,167) | 90 (58,130) | 71 (53,91) |
| 48-59 | 12 (0,31) | 12 (0,43) | 20 (11,30) | 13 (7,22) | 7 (0,20) | 62 (0,166) | 16 (0,41) | 28 (14,47) | 17 (10,27) |
| 24-59 | 85 (55,122) | 73 (40,115) | 89 (72,111) | 59 (38,85) | 46 (27,76) | 153 (83,232) | 105 (73,144) | 92 (69,119) | 78 (65,93) |
| <60 | 309 (234,399) | 614 (495,756) | 503 (423,595) | 332 (218,458) | 171 (102,271) | 431 (326,563) | 384 (320,467) | 348 (280,432) | 441 (379,503) |
| 2012 |  |  |  |  |  |  |  |  |  |
| <1 | 1883 (1201,2707) | 3907 (3033,4979) | 3688 (3059,4424) | 2387 (1644,3355) | 1049 (554,1775) | 1278 (0,3437) | 2322 (1574,3240) | 2245 (1638,3044) | 3210 (2757,3754) |
| 1 | 2127 (1404,3162) | 4459 (3420,5809) | 4237 (3526,5048) | 2753 (1828,3849) | 1195 (644,2012) | 1435 (0,3549) | 2689 (1824,3753) | 2590 (1928,3495) | 3679 (3157,4325) |
| 2 | 1542 (1024,2181) | 3198 (2505,4266) | 3054 (2537,3634) | 1963 (1281,2774) | 850 (477,1470) | 1025 (0,2741) | 1927 (1297,2684) | 1855 (1332,2493) | 2659 (2269,3105) |
| 3 | 694 (477,951) | 1919 (1416,2537) | 1386 (1126,1684) | 912 (604,1271) | 390 (225,649) | 996 (417,1714) | 862 (618,1145) | 814 (606,1079) | 1208 (1031,1421) |
| 4 | 925 (656,1289) | 2590 (1899,3393) | 1863 (1544,2274) | 1226 (809,1684) | 521 (308,839) | 1349 (583,2290) | 1163 (837,1550) | 1093 (802,1422) | 1619 (1370,1919) |
| 5 | 1298 (904,1834) | 3622 (2621,4748) | 2615 (2157,3143) | 1728 (1171,2488) | 738 (412,1190) | 1935 (826,3417) | 1631 (1196,2153) | 1524 (1170,2021) | 2284 (1952,2691) |
| 6 | 1026 (698,1447) | 2445 (1677,3418) | 1727 (1399,2102) | 1133 (735,1572) | 558 (330,955) | 2105 (1047,3380) | 1279 (920,1697) | 1369 (1021,1787) | 1514 (1276,1784) |
| 7 | 583 (382,823) | 1385 (942,1957) | 973 (795,1186) | 639 (430,885) | 320 (191,521) | 1176 (629,1909) | 722 (514,960) | 783 (575,1037) | 849 (717,1026) |
| 8 | 727 (494,1025) | 1742 (1174,2475) | 1226 (992,1485) | 797 (551,1128) | 394 (229,655) | 1477 (774,2345) | 906 (629,1198) | 979 (719,1296) | 1059 (892,1263) |
| 9 | 456 (282,666) | 521 (231,898) | 652 (534,806) | 427 (288,601) | 250 (139,434) | 572 (188,1084) | 570 (389,799) | 529 (364,724) | 567 (466,693) |
| 10 | 448 (289,653) | 513 (235,865) | 645 (511,786) | 422 (286,600) | 242 (133,412) | 562 (175,1118) | 553 (365,796) | 512 (365,704) | 561 (457,680) |
| 11 | 419 (276,657) | 477 (207,810) | 612 (485,756) | 405 (251,559) | 232 (128,403) | 546 (181,1019) | 530 (348,742) | 493 (353,669) | 532 (435,646) |
| <12 | 873 (653,1131) | 2253 (1773,2829) | 1722 (1448,2061) | 1128 (750,1586) | 486 (299,798) | 1248 (834,1767) | 1080 (879,1324) | 1058 (839,1349) | 1490 (1297,1708) |
| 12-14 | 278 (170,421) | 638 (349,979) | 492 (389,622) | 325 (213,456) | 157 (81,275) | 1102 (530,1800) | 352 (212,504) | 462 (328,650) | 426 (349,518) |
| 15-17 | 276 (114,493) | 573 (288,956) | 430 (331,561) | 281 (183,421) | 150 (61,321) | 842 (330,1517) | 342 (142,590) | 420 (278,601) | 376 (290,484) |
| 18-20 | 411 (220,669) | 404 (167,710) | 447 (347,574) | 293 (188,428) | 226 (108,416) | 581 (184,1152) | 517 (290,810) | 402 (267,563) | 391 (292,491) |
| 21-23 | 273 (151,439) | 150 (0,336) | 181 (131,247) | 118 (71,178) | 147 (71,291) | 148 (0,394) | 341 (190,547) | 152 (94,238) | 159 (114,211) |
| 12-23 | 304 (209,422) | 454 (315,626) | 390 (321,473) | 254 (164,358) | 166 (97,272) | 667 (422,925) | 369 (276,480) | 359 (275,459) | 337 (285,403) |
| <24 | 574 (433,735) | 1312 (1051,1617) | 1019 (849,1213) | 669 (451,926) | 316 (182,520) | 964 (709,1282) | 714 (594,862) | 687 (549,845) | 891 (773,1040) |
| 24-35 | 147 (89,224) | 193 (104,314) | 148 (117,186) | 97 (62,141) | 80 (44,139) | 264 (148,430) | 183 (121,268) | 142 (107,192) | 130 (105,160) |
| 36-47 | 64 (32,108) | 60 (13,121) | 63 (46,84) | 41 (26,63) | 36 (16,70) | 157 (64,289) | 79 (40,130) | 70 (44,101) | 55 (40,71) |
| 48-59 | 9 (0,22) | 13 (0,44) | 14 (8,22) | 10 (5,16) | 5 (0,14) | 31 (0,112) | 12 (0,28) | 21 (10,35) | 13 (8,19) |
| 24-59 | 67 (45,95) | 87 (52,134) | 72 (58,89) | 48 (32,67) | 37 (21,63) | 158 (92,233) | 85 (60,114) | 74 (57,97) | 63 (53,74) |
| <60 | 269 (208,350) | 584 (471,724) | 443 (371,531) | 291 (197,401) | 150 (91,230) | 481 (363,624) | 337 (276,404) | 307 (245,378) | 386 (335,449) |
| 2013 |  |  |  |  |  |  |  |  |  |
| <1 | 825 (535,1174) | 1221 (889,1673) | 1640 (1365,1979) | 1075 (712,1501) | 459 (257,822) | 1709 (751,2844) | 1045 (703,1437) | 1000 (734,1363) | 1417 (1206,1655) |
| 1 | 1279 (823,1879) | 1873 (1292,2474) | 2482 (2062,2974) | 1613 (1068,2259) | 694 (371,1183) | 2543 (1122,4282) | 1563 (1050,2179) | 1497 (1124,2013) | 2150 (1853,2548) |
| 2 | 1051 (673,1506) | 1560 (1113,2130) | 2098 (1728,2530) | 1388 (947,1937) | 596 (323,981) | 2176 (932,3497) | 1328 (899,1837) | 1290 (929,1716) | 1826 (1572,2121) |
| 3 | 529 (359,729) | 684 (373,1101) | 1056 (860,1277) | 694 (471,963) | 293 (177,476) | 579 (186,1112) | 654 (470,868) | 622 (464,829) | 921 (787,1091) |
| 4 | 614 (429,862) | 822 (447,1287) | 1235 (1010,1493) | 808 (552,1144) | 342 (196,549) | 671 (230,1274) | 767 (563,1017) | 724 (550,932) | 1080 (910,1265) |
| 5 | 726 (508,1023) | 953 (520,1491) | 1480 (1219,1776) | 959 (646,1353) | 402 (239,641) | 804 (271,1656) | 910 (660,1202) | 858 (640,1143) | 1275 (1075,1506) |
| 6 | 526 (358,728) | 379 (80,734) | 893 (735,1081) | 587 (387,829) | 291 (167,463) | 908 (372,1584) | 650 (472,873) | 708 (529,923) | 779 (648,914) |
| 7 | 397 (272,573) | 274 (58,578) | 675 (545,833) | 438 (286,621) | 216 (130,363) | 707 (259,1248) | 497 (357,654) | 539 (397,702) | 586 (496,697) |
| 8 | 341 (238,490) | 243 (58,463) | 586 (469,704) | 383 (248,527) | 191 (112,317) | 614 (252,1070) | 432 (312,581) | 466 (346,600) | 506 (425,610) |
| 9 | 140 (91,207) | 66 (0,179) | 204 (164,253) | 134 (90,190) | 77 (41,135) | 145 (36,327) | 176 (115,249) | 164 (114,225) | 178 (149,216) |
| 10 | 309 (189,430) | 148 (0,417) | 438 (349,545) | 286 (189,412) | 168 (93,286) | 322 (76,709) | 380 (247,524) | 352 (252,485) | 381 (316,469) |
| 11 | 224 (144,334) | 105 (0,284) | 327 (265,405) | 213 (142,303) | 122 (66,208) | 236 (58,497) | 282 (187,399) | 261 (187,355) | 282 (231,342) |
| <12 | 502 (382,662) | 760 (562,996) | 1002 (830,1195) | 650 (428,924) | 281 (172,435) | 746 (480,1091) | 629 (516,767) | 617 (493,758) | 869 (753,1001) |
| 12-14 | 240 (143,372) | 252 (50,507) | 422 (331,528) | 276 (179,405) | 131 (68,233) | 648 (143,1250) | 304 (185,429) | 399 (279,560) | 368 (299,456) |
| 15-17 | 115 (49,211) | 103 (0,292) | 185 (141,239) | 120 (75,174) | 64 (23,128) | 196 (0,458) | 146 (64,242) | 181 (118,251) | 160 (126,204) |
| 18-20 | 208 (109,333) | 226 (51,464) | 224 (170,287) | 146 (94,222) | 112 (54,210) | 63 (0,215) | 257 (129,393) | 204 (136,288) | 194 (151,250) |
| 21-23 | 270 (143,433) | 51 (0,169) | 182 (131,244) | 119 (76,184) | 149 (71,292) | 220 (0,499) | 336 (181,552) | 152 (91,237) | 158 (111,211) |
| 12-23 | 198 (138,271) | 159 (77,264) | 257 (212,309) | 169 (111,240) | 108 (60,178) | 244 (121,404) | 249 (184,320) | 237 (182,308) | 222 (188,263) |
| <24 | 356 (267,462) | 464 (347,624) | 631 (524,751) | 414 (286,570) | 195 (120,307) | 492 (331,697) | 440 (362,534) | 425 (343,525) | 549 (474,640) |
| 24-35 | 88 (56,131) | 24 (0,66) | 90 (71,115) | 58 (38,84) | 49 (26,87) | 231 (122,342) | 111 (72,156) | 86 (62,116) | 78 (64,96) |
| 36-47 | 39 (19,67) | . (.,.) | 39 (28,52) | 25 (16,38) | 22 (10,45) | 51 (0,121) | 51 (26,81) | 44 (28,65) | 34 (26,44) |
| 48-59 | 5 (0,14) | 0 (0,30) | 9 (5,14) | 6 (3,10) | 3 (0,8) | 19 (0,51) | 7 (0,17) | 12 (6,20) | 7 (4,11) |
| 24-59 | 42 (29,61) | 8 (0,23) | 45 (36,55) | 29 (19,42) | 23 (13,39) | 99 (62,150) | 52 (37,70) | 46 (35,60) | 39 (32,47) |
| <60 | 166 (126,212) | 189 (143,246) | 269 (225,318) | 175 (117,240) | 92 (55,144) | 257 (186,341) | 204 (168,246) | 186 (150,233) | 234 (201,272) |
| 2014 |  |  |  |  |  |  |  |  |  |
| <1 | 1048 (674,1518) | 1898 (1438,2463) | 2062 (1729,2515) | 1347 (913,1845) | 591 (325,975) | 1591 (0,3882) | 1328 (886,1818) | 1261 (926,1710) | 1801 (1536,2092) |
| 1 | 2044 (1325,2890) | 3651 (2719,4790) | 4021 (3326,4789) | 2608 (1731,3636) | 1114 (637,1915) | 3078 (0,7453) | 2572 (1741,3578) | 2458 (1793,3295) | 3490 (2967,4031) |
| 2 | 1356 (873,1952) | 2423 (1780,3191) | 2652 (2203,3177) | 1757 (1164,2454) | 744 (407,1283) | 2005 (0,4726) | 1688 (1145,2329) | 1623 (1153,2190) | 2309 (1997,2690) |
| 3 | 789 (558,1113) | 2158 (1543,2928) | 1575 (1295,1917) | 1023 (678,1429) | 427 (244,711) | 1000 (325,1827) | 969 (702,1297) | 921 (695,1204) | 1374 (1163,1625) |
| 4 | 827 (573,1189) | 2321 (1659,3074) | 1692 (1396,2042) | 1094 (745,1535) | 466 (258,803) | 1057 (429,2004) | 1038 (768,1393) | 973 (725,1283) | 1461 (1247,1699) |
| 5 | 762 (540,1075) | 2110 (1552,2831) | 1545 (1283,1855) | 1012 (698,1419) | 423 (250,698) | 985 (289,1769) | 960 (710,1259) | 896 (661,1190) | 1344 (1147,1564) |
| 6 | 727 (491,1028) | 1275 (771,1860) | 1222 (1003,1476) | 798 (553,1160) | 399 (233,687) | 1072 (354,2114) | 896 (642,1216) | 976 (735,1288) | 1063 (902,1265) |
| 7 | 772 (522,1063) | 1332 (839,1977) | 1300 (1053,1561) | 848 (567,1201) | 415 (248,681) | 1129 (376,2126) | 953 (684,1269) | 1021 (751,1376) | 1129 (945,1351) |
| 8 | 593 (400,840) | 1013 (592,1511) | 1002 (831,1207) | 652 (450,924) | 323 (184,558) | 889 (300,1689) | 737 (517,990) | 790 (570,1059) | 876 (723,1031) |
| 9 | 383 (248,584) | 433 (156,771) | 560 (449,698) | 371 (241,524) | 218 (119,370) | 533 (0,1342) | 488 (321,680) | 446 (317,603) | 489 (404,597) |
| 10 | 440 (278,647) | 494 (179,883) | 627 (502,777) | 409 (269,591) | 242 (136,416) | 633 (0,1473) | 542 (356,766) | 502 (358,681) | 548 (454,657) |
| 11 | 304 (198,464) | 347 (144,616) | 442 (352,552) | 289 (190,407) | 166 (91,295) | 442 (0,999) | 382 (262,535) | 353 (255,485) | 387 (318,468) |
| <12 | 731 (544,962) | 1673 (1317,2091) | 1440 (1210,1729) | 951 (645,1306) | 406 (237,643) | 995 (587,1523) | 916 (743,1115) | 894 (715,1127) | 1259 (1089,1441) |
| 12-14 | 223 (132,337) | 284 (92,559) | 393 (305,490) | 253 (163,376) | 122 (66,221) | 355 (0,797) | 277 (168,410) | 369 (260,505) | 341 (278,420) |
| 15-17 | 179 (76,323) | 52 (0,183) | 292 (221,379) | 190 (123,273) | 100 (37,207) | 249 (0,632) | 229 (97,384) | 285 (187,405) | 255 (195,322) |
| 18-20 | 321 (175,526) | 116 (0,299) | 355 (265,461) | 230 (151,347) | 174 (85,322) | 614 (131,1177) | 413 (226,630) | 319 (212,458) | 307 (237,385) |
| 21-23 | 362 (191,573) | 106 (0,289) | 243 (171,331) | 158 (95,241) | 201 (92,376) | 216 (0,823) | 458 (221,710) | 206 (115,315) | 212 (157,280) |
| 12-23 | 249 (176,341) | 145 (66,256) | 324 (267,399) | 217 (143,296) | 140 (81,230) | 333 (150,545) | 315 (233,412) | 299 (227,379) | 285 (238,335) |
| <24 | 493 (382,649) | 950 (747,1175) | 884 (744,1046) | 585 (408,805) | 277 (163,447) | 647 (421,934) | 622 (505,746) | 595 (481,744) | 769 (666,884) |
| 24-35 | 153 (93,232) | 91 (27,173) | 157 (123,197) | 103 (69,149) | 84 (45,150) | 79 (0,182) | 194 (128,275) | 149 (108,201) | 137 (109,168) |
| 36-47 | 65 (30,112) | 37 (0,85) | 63 (47,82) | 41 (26,61) | 35 (16,68) | 82 (0,195) | 83 (40,134) | 69 (43,102) | 54 (41,71) |
| 48-59 | 9 (0,22) | 14 (0,46) | 14 (8,21) | 9 (5,15) | 5 (0,13) | 31 (0,77) | 11 (0,28) | 20 (9,33) | 12 (7,19) |
| 24-59 | 70 (49,102) | 44 (19,77) | 75 (60,93) | 49 (33,69) | 39 (24,67) | 30 (8,71) | 88 (62,122) | 77 (59,100) | 65 (54,78) |
| <60 | 245 (188,325) | 409 (324,531) | 402 (338,478) | 263 (174,364) | 137 (83,216) | 239 (157,337) | 302 (251,363) | 277 (221,340) | 347 (301,403) |
| 2015 |  |  |  |  |  |  |  |  |  |
| <1 | 749 (480,1088) | 1274 (941,1657) | 1454 (1203,1749) | 945 (622,1304) | 402 (234,698) | 506 (0,1315) | 923 (630,1286) | 886 (645,1188) | 1261 (1089,1474) |
| 1 | 2013 (1299,2966) | 3428 (2517,4476) | 3999 (3304,4779) | 2614 (1751,3620) | 1102 (631,1903) | 1410 (0,3885) | 2518 (1728,3531) | 2426 (1745,3262) | 3455 (2958,4054) |
| 2 | 1626 (1072,2360) | 2742 (2085,3569) | 3174 (2639,3796) | 2068 (1336,2868) | 892 (500,1527) | 1079 (0,2835) | 2026 (1368,2703) | 1920 (1385,2629) | 2741 (2327,3185) |
| 3 | 831 (576,1190) | 1831 (1229,2498) | 1675 (1374,2004) | 1096 (721,1508) | 467 (264,773) | 949 (0,2248) | 1038 (779,1381) | 982 (716,1260) | 1458 (1229,1720) |
| 4 | 772 (530,1079) | 1692 (1157,2357) | 1556 (1264,1857) | 1018 (699,1421) | 425 (241,698) | 868 (0,2249) | 963 (706,1311) | 898 (679,1203) | 1354 (1164,1607) |
| 5 | 879 (605,1218) | 1894 (1313,2653) | 1756 (1456,2182) | 1154 (786,1617) | 490 (278,809) | 969 (0,2385) | 1092 (801,1404) | 1023 (752,1339) | 1530 (1300,1800) |
| 6 | 646 (465,921) | 972 (532,1557) | 1094 (895,1327) | 708 (457,987) | 355 (201,600) | 1210 (401,2372) | 798 (563,1071) | 863 (640,1146) | 950 (797,1146) |
| 7 | 491 (339,685) | 755 (408,1194) | 837 (691,1014) | 548 (369,760) | 271 (156,456) | 934 (293,1771) | 610 (450,828) | 660 (497,878) | 732 (615,884) |
| 8 | 444 (309,639) | 671 (359,1063) | 760 (622,925) | 493 (334,685) | 250 (136,412) | 831 (292,1545) | 563 (401,744) | 606 (441,794) | 664 (557,792) |
| 9 | 457 (296,667) | 527 (243,895) | 660 (528,831) | 436 (283,610) | 251 (134,431) | 969 (318,1720) | 569 (382,797) | 525 (381,736) | 573 (466,693) |
| 10 | 487 (300,731) | 564 (251,969) | 707 (564,868) | 458 (312,645) | 266 (147,462) | 1015 (336,1948) | 605 (401,858) | 570 (406,773) | 611 (507,738) |
| 11 | 351 (223,515) | 402 (171,691) | 503 (407,625) | 333 (220,467) | 193 (106,332) | 727 (237,1381) | 436 (290,609) | 403 (292,548) | 439 (357,528) |
| <12 | 687 (527,897) | 1370 (1063,1771) | 1351 (1148,1574) | 889 (574,1221) | 377 (230,610) | 1029 (414,1848) | 859 (709,1053) | 835 (660,1052) | 1180 (1010,1362) |
| 12-14 | 282 (163,445) | 220 (50,470) | 493 (389,625) | 320 (219,465) | 156 (81,274) | 265 (0,868) | 351 (211,517) | 465 (328,638) | 430 (345,527) |
| 15-17 | 205 (91,364) | 142 (0,345) | 331 (257,430) | 217 (134,314) | 112 (46,229) | 362 (0,990) | 258 (107,440) | 318 (208,450) | 290 (225,365) |
| 18-20 | 358 (190,596) | 274 (57,548) | 404 (306,512) | 261 (174,385) | 204 (99,382) | 456 (0,1189) | 461 (260,702) | 360 (235,511) | 348 (271,445) |
| 21-23 | 268 (138,439) | 103 (0,275) | 178 (127,242) | 115 (72,174) | 146 (66,274) | 234 (0,744) | 325 (171,530) | 148 (84,232) | 155 (113,207) |
| 12-23 | 271 (191,376) | 190 (98,294) | 354 (292,434) | 228 (148,323) | 151 (88,241) | 136 (0,373) | 340 (257,449) | 322 (251,422) | 307 (258,364) |
| <24 | 479 (356,632) | 781 (608,998) | 856 (722,1019) | 552 (383,778) | 265 (161,418) | 486 (205,827) | 595 (486,724) | 574 (460,706) | 743 (645,864) |
| 24-35 | 126 (80,185) | 80 (26,159) | 129 (101,160) | 84 (56,121) | 70 (36,119) | 169 (0,393) | 157 (102,229) | 124 (89,167) | 113 (91,138) |
| 36-47 | 81 (40,138) | 26 (0,67) | 78 (57,102) | 51 (32,74) | 45 (19,85) | 64 (0,208) | 100 (46,159) | 87 (55,123) | 68 (51,88) |
| 48-59 | 10 (0,25) | 12 (0,43) | 15 (9,23) | 10 (5,17) | 6 (0,15) | 0 (0,143) | 13 (0,30) | 22 (11,37) | 14 (8,21) |
| 24-59 | 70 (45,99) | 35 (14,66) | 73 (59,91) | 48 (30,67) | 38 (20,62) | 39 (0,101) | 86 (60,117) | 75 (56,98) | 64 (53,77) |
| <60 | 238 (184,308) | 339 (256,436) | 388 (328,460) | 252 (171,353) | 133 (84,211) | 200 (97,338) | 297 (249,356) | 268 (219,330) | 340 (292,396) |
| 2016 |  |  |  |  |  |  |  |  |  |
| <1 | 1453 (964,2112) | 2587 (1922,3351) | 2853 (2380,3448) | 1845 (1241,2638) | 799 (436,1355) | 2234 (748,4120) | 1812 (1215,2489) | 1743 (1250,2383) | 2478 (2108,2877) |
| 1 | 2508 (1619,3677) | 4478 (3322,5772) | 4964 (4129,6027) | 3231 (2098,4470) | 1392 (781,2477) | 3831 (1391,6917) | 3108 (2166,4437) | 3050 (2122,4079) | 4285 (3662,4974) |
| 2 | 1759 (1141,2593) | 3099 (2281,3971) | 3448 (2901,4141) | 2255 (1481,3126) | 982 (569,1700) | 2725 (915,4952) | 2167 (1437,2990) | 2100 (1481,2775) | 2987 (2582,3489) |
| 3 | 936 (640,1307) | 1468 (884,2174) | 1863 (1535,2241) | 1226 (842,1664) | 517 (293,842) | 2305 (749,4265) | 1158 (855,1539) | 1089 (815,1456) | 1620 (1400,1905) |
| 4 | 457 (316,624) | 719 (439,1074) | 919 (764,1108) | 606 (398,841) | 254 (145,429) | 1148 (378,1967) | 573 (422,753) | 537 (397,724) | 803 (679,937) |
| 5 | 794 (564,1130) | 1237 (738,1830) | 1599 (1312,1930) | 1044 (710,1458) | 444 (245,745) | 1972 (692,3579) | 990 (730,1310) | 938 (694,1230) | 1394 (1192,1616) |
| 6 | 580 (397,811) | 823 (465,1312) | 983 (810,1216) | 643 (434,908) | 321 (189,521) | 1863 (849,3099) | 730 (515,983) | 787 (590,1026) | 858 (722,1031) |
| 7 | 457 (309,644) | 658 (332,1074) | 777 (635,954) | 511 (335,725) | 259 (145,435) | 1451 (649,2432) | 573 (399,763) | 618 (460,824) | 679 (563,804) |
| 8 | 683 (465,970) | 989 (525,1581) | 1148 (940,1422) | 758 (495,1081) | 372 (209,616) | 2140 (1012,3497) | 839 (603,1144) | 913 (675,1215) | 1000 (841,1187) |
| 9 | 356 (235,524) | 455 (145,860) | 515 (411,651) | 337 (214,481) | 197 (112,337) | 1245 (561,2084) | 438 (293,630) | 414 (289,557) | 448 (364,544) |
| 10 | 288 (181,409) | 376 (122,714) | 426 (343,521) | 273 (176,392) | 164 (87,284) | 1003 (462,1711) | 366 (241,524) | 337 (238,470) | 368 (298,444) |
| 11 | 140 (89,207) | 177 (62,357) | 204 (164,255) | 134 (88,186) | 78 (42,138) | 495 (225,828) | 176 (116,250) | 163 (115,222) | 178 (146,217) |
| <12 | 712 (550,929) | 1452 (1111,1869) | 1401 (1161,1660) | 933 (609,1282) | 400 (238,619) | 1920 (1054,2913) | 891 (722,1089) | 867 (690,1067) | 1219 (1055,1417) |
| 12-14 | 197 (118,301) | 151 (0,344) | 346 (272,430) | 225 (145,319) | 111 (60,189) | 741 (0,1745) | 248 (157,362) | 327 (222,447) | 301 (242,376) |
| 15-17 | 237 (104,421) | 57 (0,198) | 372 (287,479) | 243 (157,367) | 128 (51,253) | 614 (0,1569) | 293 (127,481) | 361 (242,515) | 327 (251,419) |
| 18-20 | 222 (127,353) | 161 (0,366) | 241 (184,310) | 158 (100,230) | 125 (59,235) | 0 (0,1048) | 280 (156,445) | 216 (140,313) | 210 (162,267) |
| 21-23 | 367 (206,621) | 50 (0,172) | 256 (179,338) | 165 (99,254) | 206 (97,387) | 252 (0,796) | 468 (247,729) | 214 (116,340) | 220 (156,287) |
| 12-23 | 237 (166,333) | 107 (42,192) | 311 (254,371) | 203 (136,285) | 130 (78,222) | 391 (82,791) | 298 (219,391) | 285 (222,366) | 269 (229,320) |
| <24 | 466 (360,607) | 756 (584,974) | 840 (700,993) | 549 (354,778) | 258 (157,419) | 1070 (631,1597) | 584 (479,699) | 557 (444,693) | 724 (629,837) |
| 24-35 | 120 (74,179) | 62 (14,129) | 124 (97,152) | 80 (52,111) | 67 (36,113) | 178 (0,413) | 150 (101,211) | 117 (82,160) | 107 (86,129) |
| 36-47 | 59 (28,98) | 40 (0,94) | 59 (43,78) | 38 (24,57) | 33 (15,64) | 141 (0,368) | 74 (38,127) | 65 (43,92) | 51 (38,66) |
| 48-59 | 10 (0,25) | 13 (0,46) | 15 (8,23) | 9 (5,16) | 5 (0,15) | 0 (0,131) | 12 (0,29) | 21 (10,36) | 13 (7,20) |
| 24-59 | 62 (42,88) | 36 (13,66) | 66 (53,81) | 43 (29,62) | 35 (19,58) | 123 (39,238) | 77 (56,104) | 67 (52,89) | 58 (47,68) |
| <60 | 222 (168,285) | 324 (247,415) | 364 (306,435) | 236 (161,329) | 125 (74,196) | 469 (308,663) | 278 (230,330) | 253 (201,310) | 317 (276,368) |
| 2017 |  |  |  |  |  |  |  |  |  |
| <1 | 1028 (676,1510) | 1167 (763,1666) | 2005 (1658,2381) | 1305 (854,1775) | 575 (309,1009) | 2074 (0,4922) | 1267 (864,1758) | 1240 (887,1690) | 1746 (1509,2043) |
| 1 | 1278 (829,1828) | 1471 (958,2140) | 2522 (2075,3001) | 1646 (1091,2284) | 712 (380,1234) | 2583 (0,5748) | 1589 (1095,2172) | 1520 (1111,2048) | 2192 (1884,2579) |
| 2 | 732 (471,1086) | 841 (545,1229) | 1442 (1201,1739) | 937 (647,1324) | 408 (222,706) | 1471 (0,3361) | 917 (637,1247) | 881 (642,1178) | 1251 (1081,1465) |
| 3 | 557 (377,763) | 873 (448,1407) | 1111 (924,1333) | 733 (484,1010) | 304 (178,511) | 1781 (0,4167) | 685 (507,915) | 647 (485,859) | 962 (820,1136) |
| 4 | 421 (299,582) | 672 (345,1071) | 855 (709,1038) | 564 (368,799) | 235 (141,379) | 1399 (0,3170) | 532 (382,688) | 500 (380,657) | 746 (639,875) |
| 5 | 307 (211,428) | 489 (248,778) | 619 (514,750) | 403 (277,562) | 173 (98,285) | 987 (0,2286) | 386 (281,502) | 359 (268,475) | 540 (457,629) |
| 6 | 231 (161,333) | 309 (108,597) | 391 (316,475) | 257 (171,356) | 126 (72,214) | 198 (0,663) | 291 (207,393) | 309 (232,406) | 338 (287,407) |
| 7 | 266 (177,370) | 366 (115,689) | 452 (364,554) | 293 (193,419) | 148 (83,247) | 236 (0,757) | 332 (236,444) | 358 (263,470) | 393 (331,469) |
| 8 | 239 (161,337) | 317 (108,587) | 396 (324,492) | 258 (171,360) | 130 (77,210) | 210 (0,695) | 293 (205,390) | 316 (238,413) | 348 (293,409) |
| 9 | 233 (149,349) | 304 (69,610) | 335 (268,412) | 220 (148,319) | 125 (68,210) | 224 (0,738) | 287 (194,405) | 262 (189,362) | 290 (239,350) |
| 10 | 204 (127,303) | 257 (88,527) | 298 (237,366) | 194 (126,273) | 111 (60,189) | 206 (0,653) | 257 (172,363) | 239 (166,325) | 259 (211,315) |
| 11 | 203 (126,302) | 262 (59,522) | 293 (235,365) | 191 (126,273) | 112 (61,195) | 200 (0,676) | 251 (170,350) | 236 (167,318) | 256 (207,305) |
| <12 | 405 (307,539) | 641 (462,867) | 798 (660,956) | 516 (344,727) | 222 (140,353) | 621 (0,1353) | 502 (405,613) | 488 (394,610) | 694 (590,800) |
| 12-14 | 124 (74,188) | 106 (0,294) | 213 (168,272) | 140 (94,198) | 67 (34,123) | 264 (0,895) | 152 (92,220) | 203 (137,276) | 184 (147,227) |
| 15-17 | 88 (37,162) | 0 (0,114) | 144 (107,186) | 93 (59,138) | 49 (19,99) | 0 (0,715) | 113 (50,194) | 138 (89,197) | 125 (94,159) |
| 18-20 | 115 (65,184) | 53 (0,185) | 126 (96,162) | 83 (52,120) | 64 (30,118) | 0 (0,555) | 144 (80,224) | 114 (74,165) | 111 (87,139) |
| 21-23 | 128 (66,209) | 53 (0,172) | 84 (61,114) | 56 (34,82) | 69 (31,135) | 0 (0,506) | 159 (86,254) | 72 (39,110) | 74 (53,98) |
| 12-23 | 109 (78,148) | 53 (12,117) | 142 (117,174) | 93 (62,131) | 60 (35,100) | 152 (0,424) | 136 (101,175) | 131 (101,169) | 124 (105,146) |
| <24 | 250 (190,325) | 347 (252,470) | 447 (373,537) | 294 (194,412) | 142 (87,212) | 390 (133,728) | 314 (260,379) | 301 (240,373) | 390 (340,452) |
| 24-35 | 64 (40,93) | 26 (0,71) | 66 (52,82) | 43 (29,62) | 36 (20,61) | 100 (0,305) | 80 (53,115) | 63 (46,85) | 57 (46,70) |
| 36-47 | 31 (15,51) | 13 (0,45) | 30 (22,40) | 20 (12,29) | 17 (8,33) | 0 (0,191) | 38 (20,60) | 33 (21,49) | 26 (20,34) |
| 48-59 | 4 (0,11) | 14 (0,46) | 7 (4,11) | 4 (2,7) | 2 (0,6) | 0 (0,77) | 6 (0,14) | 10 (5,16) | 6 (3,10) |
| 24-59 | 32 (22,45) | 18 (3,38) | 34 (27,42) | 22 (15,30) | 17 (10,30) | 49 (0,133) | 40 (27,54) | 35 (26,44) | 30 (25,35) |
| <60 | 120 (90,153) | 151 (108,202) | 195 (164,230) | 127 (84,179) | 66 (41,106) | 176 (69,325) | 148 (122,178) | 135 (109,166) | 168 (146,193) |
| 2018 |  |  |  |  |  |  |  |  |  |
| <1 | 2557 (1625,3714) | 3864 (2793,5163) | 5080 (4224,6112) | 3324 (2166,4632) | 1427 (785,2408) | 6287 (0,13592) | 3246 (2178,4546) | 3099 (2232,4281) | 4437 (3826,5217) |
| 1 | 2116 (1356,3101) | 3163 (2215,4201) | 4131 (3485,5002) | 2700 (1804,3724) | 1193 (650,2027) | 5083 (0,11309) | 2634 (1779,3624) | 2564 (1812,3414) | 3645 (3108,4202) |
| 2 | 1286 (806,1837) | 1905 (1389,2553) | 2524 (2078,3073) | 1653 (1082,2269) | 703 (392,1234) | 3124 (0,6623) | 1603 (1068,2299) | 1545 (1093,2064) | 2194 (1877,2536) |
| 3 | 882 (611,1266) | 1418 (914,2082) | 1769 (1445,2129) | 1146 (768,1586) | 491 (292,801) | 2719 (615,4917) | 1108 (791,1468) | 1025 (765,1347) | 1531 (1303,1797) |
| 4 | 992 (683,1401) | 1631 (1016,2382) | 2017 (1652,2420) | 1301 (868,1799) | 539 (311,909) | 2977 (704,5453) | 1267 (927,1659) | 1181 (873,1577) | 1746 (1488,2052) |
| 5 | 777 (533,1084) | 1263 (807,1837) | 1575 (1302,1901) | 1015 (698,1452) | 430 (246,692) | 2241 (517,4355) | 972 (705,1300) | 913 (680,1228) | 1367 (1181,1596) |
| 6 | 612 (419,914) | 695 (362,1170) | 1049 (857,1279) | 697 (459,957) | 338 (198,558) | 2074 (688,3829) | 772 (544,1048) | 834 (613,1105) | 922 (760,1095) |
| 7 | 505 (334,708) | 554 (278,899) | 847 (676,1033) | 559 (374,778) | 280 (163,459) | 1680 (566,3022) | 629 (442,831) | 671 (503,891) | 738 (624,888) |
| 8 | 712 (484,975) | 802 (386,1290) | 1211 (985,1482) | 788 (547,1102) | 396 (215,644) | 2436 (838,4499) | 886 (629,1185) | 962 (735,1286) | 1050 (882,1270) |
| 9 | 401 (256,606) | 547 (246,921) | 584 (460,725) | 381 (258,543) | 222 (122,374) | 849 (0,1905) | 507 (326,711) | 465 (335,631) | 505 (413,622) |
| 10 | 446 (282,661) | 594 (281,1016) | 641 (512,804) | 420 (279,599) | 244 (139,415) | 944 (0,2080) | 559 (360,787) | 520 (373,716) | 559 (456,677) |
| 11 | 330 (212,482) | 460 (201,774) | 477 (384,601) | 315 (205,455) | 183 (102,341) | 707 (0,1545) | 421 (289,589) | 382 (273,531) | 416 (342,505) |
| <12 | 788 (591,1036) | 1364 (1021,1766) | 1561 (1307,1870) | 1023 (692,1391) | 431 (260,708) | 2128 (1243,3238) | 989 (788,1221) | 964 (773,1209) | 1367 (1173,1582) |
| 12-14 | 200 (114,310) | 181 (41,385) | 347 (274,435) | 228 (145,329) | 110 (54,188) | 237 (0,773) | 249 (151,368) | 331 (225,463) | 303 (242,375) |
| 15-17 | 161 (66,291) | 102 (0,277) | 265 (200,346) | 173 (112,254) | 90 (34,173) | 369 (0,870) | 210 (98,358) | 256 (171,363) | 228 (177,294) |
| 18-20 | 327 (172,532) | 154 (0,370) | 367 (280,465) | 235 (155,348) | 187 (87,327) | 290 (0,1126) | 420 (228,647) | 328 (217,460) | 316 (246,392) |
| 21-23 | 302 (159,502) | 57 (0,197) | 206 (146,279) | 133 (78,207) | 173 (78,314) | 271 (0,891) | 382 (203,599) | 171 (94,269) | 179 (130,239) |
| 12-23 | 230 (160,319) | 91 (28,173) | 298 (246,361) | 196 (132,274) | 127 (74,207) | 278 (58,579) | 286 (212,374) | 274 (213,360) | 260 (218,310) |
| <24 | 498 (382,647) | 693 (524,893) | 888 (745,1068) | 576 (399,796) | 275 (166,422) | 1016 (606,1498) | 621 (504,762) | 592 (469,731) | 771 (667,903) |
| 24-35 | 155 (94,236) | 63 (14,129) | 160 (127,198) | 105 (69,147) | 86 (45,148) | 246 (59,509) | 197 (126,273) | 151 (110,206) | 138 (113,171) |
| 36-47 | 66 (32,114) | 26 (0,75) | 66 (48,86) | 42 (26,62) | 37 (17,67) | 183 (43,383) | 83 (42,137) | 72 (47,105) | 57 (42,74) |
| 48-59 | 11 (0,27) | 28 (0,78) | 17 (9,27) | 11 (6,19) | 6 (0,16) | 26 (0,96) | 14 (0,33) | 24 (12,41) | 15 (8,23) |
| 24-59 | 74 (50,105) | 9 (0,24) | 79 (65,97) | 52 (33,73) | 41 (22,71) | 82 (28,163) | 94 (67,124) | 81 (60,106) | 68 (57,82) |
| <60 | 242 (186,315) | 280 (213,360) | 395 (333,469) | 259 (170,356) | 132 (83,215) | 388 (254,559) | 301 (251,365) | 276 (221,341) | 345 (299,394) |

**Table S8: Annual rates of non-hospitalized severe acute respiratory illness per 100,000 children by regions and age in months, 2010-2018**

| Year and Age in months | Central Rate (95% CI) | Coast Rate (95% CI) | Eastern Rate (95% CI) | North Eastern Rate (95% CI) | Nairobi Rate (95% CI) | Nyanza Rate (95% CI) | Rift Valley Rate (95% CI) | Western Rate (95% CI) | Kenya Rate (95% CI) |
| --- | --- | --- | --- | --- | --- | --- | --- | --- | --- |
| 2010-2018 |  |  |  |  |  |  |  |  |  |
| <1 | 18656 (14356,23246) | 21079 (17467,25153) | 26470 (22141,31175) | 32804 (21380,46260) | 11851 (7572,18094) | 28648 (24290,33719) | 26935 (23339,31322) | 14853 (12105,18014) | 21939 (19138,25179) |
| 1 | 26905 (20860,33992) | 30584 (25247,36878) | 38202 (31741,44815) | 47508 (30688,65565) | 16906 (10150,26723) | 41394 (34787,49015) | 38989 (33873,45379) | 21444 (17708,26002) | 31540 (27667,36469) |
| 2 | 19721 (15294,24589) | 22246 (18292,26826) | 28103 (23601,33340) | 34828 (23004,48481) | 12319 (7751,19223) | 30013 (25189,35713) | 28292 (24457,33043) | 15656 (12810,19167) | 23044 (20233,26463) |
| 3 | 18741 (14684,23456) | 21262 (17583,25599) | 26525 (22469,31485) | 32765 (22038,45208) | 11834 (7623,19161) | 28907 (24431,34009) | 27425 (23357,32095) | 14999 (12330,18428) | 22155 (19321,25429) |
| 4 | 17021 (13275,21155) | 19156 (15799,22966) | 24058 (20526,28753) | 30015 (20075,40614) | 10789 (6632,16773) | 26211 (21898,31022) | 24700 (21195,28839) | 13504 (11146,16167) | 19905 (17422,22663) |
| 5 | 18523 (14502,24113) | 21126 (17447,25415) | 26635 (22251,31359) | 32474 (22184,45598) | 11828 (7243,18936) | 28640 (24168,33754) | 27012 (23181,31366) | 14785 (11964,18183) | 21941 (19196,25045) |
| 6 | 18231 (14404,22702) | 20823 (17197,25166) | 26181 (22171,30766) | 32518 (21335,45610) | 11418 (7148,17645) | 28038 (23779,32705) | 26533 (22792,30674) | 14468 (12037,17586) | 21533 (18660,24871) |
| 7 | 15674 (12269,20061) | 17816 (14759,21619) | 22310 (18949,26655) | 27598 (18834,37947) | 10062 (6182,15002) | 24122 (20137,28512) | 22820 (19402,26430) | 12474 (10428,15078) | 18590 (16116,21498) |
| 8 | 14621 (11591,18133) | 16646 (13516,20141) | 20894 (17558,24872) | 26082 (18187,35833) | 9330 (5543,14556) | 22717 (18942,26872) | 21372 (18353,24861) | 11720 (9673,14377) | 17423 (15008,19930) |
| 9 | 11593 (9137,14609) | 13123 (10767,15809) | 16534 (13912,19663) | 20447 (13993,28099) | 7403 (4449,11641) | 17943 (15244,21380) | 17085 (14639,19694) | 9318 (7580,11349) | 13790 (12017,15841) |
| 10 | 12893 (10229,16276) | 14672 (11947,17436) | 18357 (15545,21761) | 22818 (15046,31329) | 8232 (5135,12866) | 19782 (16762,23151) | 18887 (16204,21895) | 10272 (8540,12485) | 15300 (13395,17515) |
| 11 | 10581 (8228,13358) | 12005 (9905,14572) | 15010 (12492,17811) | 18803 (12564,25714) | 6717 (4153,10485) | 16330 (13957,19399) | 15430 (13255,17885) | 8518 (6886,10118) | 12528 (10985,14328) |
| <12 | 16934 (13353,21061) | 19337 (15958,22983) | 24201 (20242,28674) | 29905 (20308,42087) | 10829 (6895,16805) | 26324 (22378,31435) | 24830 (21443,28370) | 13490 (11249,16574) | 20035 (17302,23013) |
| 12-14 | 9432 (7372,11962) | 10756 (8946,12886) | 13314 (11217,15981) | 16539 (11309,23037) | 5885 (3652,9283) | 14368 (12171,17102) | 13769 (11751,16026) | 7544 (6186,9173) | 11133 (9696,12830) |
| 15-17 | 7810 (6018,9863) | 8890 (7369,10661) | 11090 (9358,13282) | 13887 (9442,18888) | 4962 (2966,7369) | 12148 (10138,14453) | 11374 (9856,13168) | 6271 (5150,7632) | 9207 (8071,10676) |
| 18-20 | 7909 (6194,10019) | 9000 (7376,10616) | 11280 (9440,13195) | 13928 (9353,19204) | 4917 (3121,7683) | 12161 (10289,14351) | 11471 (9882,13279) | 6317 (5221,7686) | 9350 (8005,10795) |
| 21-23 | 5606 (4372,7098) | 6343 (5174,7670) | 7919 (6649,9391) | 9936 (6570,13554) | 3564 (2172,5517) | 8605 (7265,10235) | 8155 (7085,9468) | 4466 (3705,5428) | 6565 (5727,7605) |
| 12-23 | 7683 (6076,9733) | 8757 (7258,10520) | 10945 (9045,12897) | 13636 (9109,18837) | 4947 (2947,7630) | 11895 (9967,14199) | 11199 (9606,12983) | 6142 (5063,7498) | 9088 (7917,10469) |
| <24 | 12183 (9611,15467) | 13951 (11546,16996) | 17603 (14962,20643) | 21923 (14670,30036) | 7860 (4910,12021) | 18927 (16077,22233) | 18097 (15509,20923) | 9881 (8127,12088) | 14617 (12733,16748) |
| 24-35 | 3907 (3103,4910) | 4467 (3671,5372) | 5606 (4707,6658) | 6928 (4710,9389) | 2517 (1550,3806) | 6062 (5212,7161) | 5717 (4896,6603) | 3115 (2564,3829) | 4643 (4073,5319) |
| 36-47 | 2547 (1987,3176) | 2871 (2351,3429) | 3623 (3061,4275) | 4435 (3085,6094) | 1618 (961,2533) | 3895 (3276,4610) | 3684 (3222,4331) | 2028 (1657,2448) | 2998 (2608,3455) |
| 48-59 | 1490 (1167,1866) | 1684 (1392,2007) | 2098 (1747,2487) | 2646 (1726,3592) | 946 (582,1430) | 2302 (1953,2736) | 2169 (1863,2537) | 1183 (964,1438) | 1755 (1521,2015) |
| 24-59 | 2665 (2078,3338) | 3006 (2481,3617) | 3776 (3149,4377) | 4636 (3139,6356) | 1677 (1074,2664) | 4073 (3453,4823) | 3864 (3352,4458) | 2134 (1713,2607) | 3141 (2736,3593) |
| <60 | 6536 (5195,8288) | 7398 (6130,8931) | 9265 (7893,10994) | 11607 (7762,15892) | 4235 (2617,6615) | 10052 (8456,12039) | 9492 (8166,10978) | 5174 (4251,6378) | 7717 (6734,8855) |
| 2010 |  |  |  |  |  |  |  |  |  |
| <1 | 35215 (27512,45324) | 39999 (32843,49544) | 49897 (42239,59400) | 61890 (41392,86218) | 22391 (13798,35196) | 54213 (45815,64686) | 51083 (44082,59958) | 28159 (22979,34155) | 41381 (36319,47752) |
| 1 | 34944 (27650,44009) | 40018 (32777,47853) | 49853 (42154,58285) | 61917 (40556,87074) | 22035 (13006,34615) | 53983 (45556,64892) | 51126 (43683,59632) | 27900 (22971,34320) | 41450 (35769,47354) |
| 2 | 29288 (23426,37715) | 33408 (27767,39615) | 41284 (35189,49572) | 52359 (33407,72152) | 18338 (11173,28456) | 44855 (37976,53423) | 42793 (37117,49862) | 23319 (19184,28524) | 34722 (30181,39820) |
| 3 | 27222 (21156,34466) | 31037 (25591,37851) | 38926 (32510,46189) | 48389 (33207,65709) | 17457 (10963,27171) | 42191 (35371,49722) | 39883 (33864,46292) | 21792 (17650,26441) | 32323 (28065,36805) |
| 4 | 20574 (15996,25918) | 23418 (19285,28298) | 29100 (24488,34493) | 36436 (24675,50156) | 13076 (8181,20181) | 31663 (26582,37017) | 29783 (25652,34966) | 16538 (13341,19992) | 24230 (21126,27993) |
| 5 | 26752 (21139,33440) | 30694 (25378,37160) | 38277 (32408,45547) | 47650 (32313,67087) | 17272 (10649,27225) | 41508 (35257,48809) | 39230 (33658,45117) | 21680 (17730,26499) | 31756 (27810,36223) |
| 6 | 32210 (24826,40080) | 36244 (30143,43277) | 45684 (38004,53872) | 55928 (36091,77641) | 20357 (12743,31971) | 49236 (41644,57822) | 46657 (40444,54645) | 25642 (21232,31025) | 37677 (33363,43867) |
| 7 | 29624 (23031,36692) | 33125 (27617,39943) | 42101 (36037,49420) | 52082 (35198,70952) | 18564 (11444,29919) | 45674 (38146,53693) | 43055 (37391,49805) | 23475 (19092,28469) | 34655 (30389,39687) |
| 8 | 18413 (14436,23128) | 20842 (17703,25198) | 26130 (22158,30689) | 32648 (21377,43933) | 11736 (7241,17616) | 28362 (23855,33270) | 26881 (22994,31133) | 14653 (12058,17739) | 21759 (18669,25161) |
| 9 | 23831 (18639,30223) | 26603 (22153,32169) | 33507 (28341,39461) | 41809 (28541,57525) | 15094 (9298,23577) | 36281 (30609,42874) | 34360 (29858,39531) | 18641 (15387,22863) | 27675 (23887,31706) |
| 10 | 27413 (21721,34325) | 30834 (25809,37020) | 38513 (32275,45968) | 47626 (32436,65655) | 16988 (11049,27286) | 42110 (35386,49924) | 39654 (33942,46616) | 21722 (17933,26312) | 31963 (27863,37068) |
| 11 | 22490 (17645,28618) | 25532 (21210,30216) | 31990 (27121,37846) | 39603 (27288,53796) | 14331 (8891,21886) | 34995 (29449,41397) | 32885 (28322,37892) | 18155 (14753,21814) | 26627 (23234,30487) |
| <12 | 27513 (21423,34693) | 31101 (25094,37433) | 38745 (32847,45927) | 48085 (32345,65721) | 17409 (10750,26992) | 42059 (35237,49721) | 39854 (34301,46396) | 21745 (17735,26340) | 32153 (27887,36732) |
| 12-14 | 15632 (12113,19249) | 17755 (14661,21270) | 21943 (18686,26172) | 27063 (19011,38070) | 9860 (6041,15240) | 23988 (20099,28410) | 22516 (19304,26389) | 12408 (10348,14913) | 18306 (15957,21003) |
| 15-17 | 13446 (10465,16897) | 15033 (12460,18010) | 18850 (15759,22197) | 23297 (15682,31582) | 8309 (5158,12754) | 20496 (17508,24198) | 19342 (16804,22360) | 10590 (8644,12937) | 15666 (13733,18152) |
| 18-20 | 12479 (9761,15660) | 14119 (11543,17042) | 17698 (14727,20987) | 22008 (14598,30406) | 7961 (5126,12484) | 19235 (16019,22752) | 18045 (15623,21106) | 9912 (8176,12062) | 14670 (12783,16829) |
| 21-23 | 7192 (5562,8950) | 8106 (6757,9851) | 10108 (8524,12032) | 12522 (8581,16977) | 4476 (2871,6987) | 11049 (9273,13226) | 10389 (8859,12099) | 5715 (4604,6988) | 8416 (7340,9588) |
| 12-23 | 11980 (9601,15087) | 13658 (11271,16446) | 17103 (14605,20494) | 21657 (14463,29311) | 7546 (4728,12357) | 18576 (15730,21937) | 17509 (15125,20280) | 9597 (7883,11798) | 14224 (12463,16250) |
| <24 | 19171 (15021,23995) | 21924 (18375,26391) | 27464 (22850,32313) | 33760 (21847,46738) | 12129 (7701,18934) | 29553 (25139,34912) | 28101 (24120,32234) | 15278 (12667,18677) | 22639 (19762,25877) |
| 24-35 | 6125 (4734,7708) | 6925 (5657,8407) | 8666 (7361,10346) | 10798 (7029,14642) | 3862 (2398,5874) | 9370 (7886,11129) | 8874 (7683,10268) | 4886 (3987,5999) | 7214 (6258,8214) |
| 36-47 | 4528 (3643,5720) | 5187 (4277,6230) | 6430 (5370,7686) | 7979 (5376,11056) | 2852 (1779,4354) | 7037 (5877,8317) | 6620 (5662,7686) | 3597 (2966,4366) | 5380 (4680,6172) |
| 48-59 | 2530 (2023,3137) | 2860 (2399,3456) | 3554 (2993,4247) | 4402 (2947,6122) | 1574 (974,2429) | 3853 (3246,4536) | 3664 (3180,4225) | 2001 (1641,2427) | 2976 (2586,3428) |
| 24-59 | 4433 (3460,5536) | 5043 (4105,6044) | 6240 (5351,7416) | 7839 (5247,10570) | 2805 (1727,4312) | 6788 (5734,8001) | 6428 (5576,7400) | 3515 (2894,4217) | 5196 (4564,5995) |
| <60 | 10320 (7910,13109) | 11674 (9700,13891) | 14602 (12163,17219) | 18114 (12112,25388) | 6621 (4090,10249) | 15873 (13396,18883) | 14947 (12973,17305) | 8238 (6666,9938) | 12092 (10577,14072) |
| 2011 |  |  |  |  |  |  |  |  |  |
| <1 | 25011 (19543,31507) | 28462 (23610,34167) | 35667 (30001,42324) | 44073 (27886,59859) | 16118 (10183,24798) | 38691 (32939,45939) | 36651 (31164,42163) | 20043 (16458,24215) | 29544 (25890,33805) |
| 1 | 28120 (21501,35048) | 31730 (26358,37961) | 39702 (33997,47361) | 49193 (32334,67157) | 17902 (11087,27396) | 43287 (36349,50589) | 40877 (35117,47154) | 22388 (18421,27036) | 32966 (29003,38107) |
| 2 | 20832 (16391,26116) | 23562 (19603,28124) | 29692 (24871,35478) | 36906 (25134,51095) | 13188 (8201,20702) | 32137 (26996,38592) | 30683 (26352,35687) | 16652 (13438,20129) | 24600 (21490,28199) |
| 3 | 28527 (22432,35518) | 32146 (26754,39129) | 40329 (34238,48163) | 49854 (33654,68912) | 18113 (11094,28119) | 43748 (37100,51851) | 41376 (36104,47995) | 22567 (18511,27510) | 33471 (28983,38593) |
| 4 | 22450 (17434,28562) | 25492 (21227,30689) | 31761 (26682,37250) | 39557 (26091,54518) | 14161 (8600,21804) | 34443 (29150,40840) | 32704 (28001,37835) | 17845 (14504,21950) | 26274 (23064,30419) |
| 5 | 21496 (16773,26933) | 24500 (20180,29315) | 30487 (25855,36381) | 37703 (25346,52587) | 13341 (8281,21349) | 33184 (27956,38626) | 31272 (26748,36306) | 17118 (13741,20772) | 25245 (22052,29194) |
| 6 | 25058 (19804,31800) | 28484 (23080,34633) | 35369 (30212,42216) | 44262 (30529,60229) | 15829 (9867,25098) | 38555 (32194,45010) | 36296 (31647,42040) | 20024 (16353,24118) | 29611 (25983,33979) |
| 7 | 27636 (21735,34268) | 31262 (25837,37204) | 39072 (32597,46803) | 48423 (31925,67412) | 17463 (10866,28086) | 42219 (35509,49885) | 39857 (34189,45750) | 21677 (17884,26570) | 32426 (28280,37213) |
| 8 | 17505 (13704,22086) | 19804 (16500,23677) | 25037 (20876,29162) | 30649 (19878,42600) | 11140 (6860,17002) | 26952 (22555,31986) | 25445 (22055,29507) | 13953 (11477,17119) | 20579 (18084,23623) |
| 9 | 11992 (9374,15133) | 13653 (11253,16298) | 17026 (14512,20334) | 21346 (14317,29257) | 7619 (4698,11835) | 18613 (15919,21768) | 17640 (15076,20231) | 9644 (8031,11574) | 14189 (12356,16536) |
| 10 | 13835 (11179,17448) | 15844 (12977,18806) | 19648 (16427,23353) | 24592 (16603,33289) | 8790 (5222,13780) | 21390 (17974,25264) | 20234 (17432,23392) | 10999 (8926,13544) | 16427 (14180,18877) |
| 11 | 15357 (11918,19448) | 17411 (14359,20666) | 21857 (18594,25678) | 27091 (17792,37262) | 9643 (6036,15260) | 23580 (20119,28103) | 22415 (19421,26180) | 12300 (9966,14898) | 18150 (16051,21081) |
| <12 | 21346 (16530,26884) | 24388 (20123,29390) | 30535 (25653,35726) | 37254 (24836,51517) | 13408 (8401,20789) | 32793 (27847,38873) | 30993 (26726,36008) | 17028 (13957,20601) | 25171 (22058,28762) |
| 12-14 | 10336 (8071,13016) | 11576 (9686,14125) | 14592 (12453,17114) | 18032 (12309,25083) | 6566 (4135,10185) | 15815 (13446,18638) | 14968 (12775,17329) | 8220 (6749,9913) | 12114 (10454,13907) |
| 15-17 | 8528 (6803,10858) | 9807 (8059,11675) | 12162 (10267,14384) | 15261 (10125,20431) | 5508 (3469,8256) | 13210 (11044,15597) | 12509 (10771,14545) | 6854 (5657,8420) | 10108 (8868,11567) |
| 18-20 | 12607 (10049,15969) | 14397 (12045,17289) | 18008 (15098,21175) | 22094 (15062,30836) | 8035 (5017,12647) | 19470 (16178,22916) | 18446 (15781,21476) | 10132 (8201,12258) | 15042 (12995,17122) |
| 21-23 | 7858 (6096,9956) | 9018 (7499,10764) | 11287 (9591,13190) | 14074 (9168,18977) | 5104 (3060,7869) | 12133 (10251,14330) | 11550 (9829,13226) | 6315 (5289,7635) | 9356 (8170,10719) |
| 12-23 | 9707 (7728,12316) | 11094 (9175,13178) | 13815 (11733,16242) | 17246 (11927,23468) | 6207 (3835,9280) | 14944 (12614,17722) | 14229 (12137,16559) | 7832 (6322,9431) | 11517 (9948,13392) |
| <24 | 15868 (12449,20066) | 18049 (14901,21771) | 22587 (18923,26926) | 27968 (19029,37883) | 9846 (6186,15380) | 24554 (20837,28740) | 23090 (19939,26707) | 12690 (10421,15527) | 18754 (16234,21498) |
| 24-35 | 4668 (3622,5882) | 5311 (4457,6362) | 6607 (5576,7830) | 8181 (5593,11321) | 2943 (1838,4575) | 7132 (5999,8410) | 6790 (5800,7917) | 3723 (3004,4495) | 5501 (4803,6317) |
| 36-47 | 3095 (2364,3933) | 3498 (2870,4144) | 4405 (3685,5179) | 5400 (3577,7631) | 1955 (1187,3004) | 4743 (4020,5607) | 4488 (3858,5248) | 2464 (2033,2967) | 3653 (3179,4165) |
| 48-59 | 1940 (1525,2493) | 2200 (1798,2598) | 2737 (2313,3253) | 3373 (2314,4664) | 1235 (769,1914) | 2966 (2510,3513) | 2815 (2448,3264) | 1529 (1249,1885) | 2278 (2001,2623) |
| 24-59 | 3280 (2537,4146) | 3725 (3047,4524) | 4633 (3857,5471) | 5806 (3968,8005) | 2058 (1298,3187) | 5031 (4240,5918) | 4756 (4171,5496) | 2587 (2093,3163) | 3844 (3347,4395) |
| <60 | 8287 (6437,10484) | 9391 (7883,11258) | 11664 (9897,13811) | 14611 (9753,19900) | 5237 (3175,8235) | 12678 (10657,15069) | 12002 (10414,13959) | 6584 (5367,7961) | 9762 (8446,11137) |
| 2012 |  |  |  |  |  |  |  |  |  |
| <1 | 25156 (19296,32116) | 28595 (23651,34524) | 35774 (29988,42103) | 43796 (30332,61502) | 16159 (9578,24990) | 39229 (32795,46204) | 36724 (31528,42727) | 20070 (16661,24461) | 29755 (25803,33867) |
| 1 | 29046 (22541,36202) | 32715 (27263,39629) | 41180 (34640,48353) | 50447 (33258,69986) | 18312 (11147,28554) | 44567 (37641,52195) | 42091 (36650,48783) | 23121 (18851,28285) | 34133 (29671,39050) |
| 2 | 20626 (16273,26207) | 23485 (19748,28423) | 29507 (24827,34691) | 36042 (23344,51076) | 13139 (8074,20651) | 31974 (26879,37536) | 30209 (25876,34966) | 16439 (13442,20294) | 24498 (21237,28200) |
| 3 | 15026 (11734,18824) | 16956 (13963,20200) | 21209 (17813,25279) | 26175 (17614,36072) | 9620 (5917,14802) | 22905 (19201,27156) | 21718 (18631,25131) | 11931 (9599,14578) | 17626 (15390,20082) |
| 4 | 20057 (15554,25318) | 22725 (18544,27251) | 28244 (24031,33736) | 35315 (23684,48137) | 12991 (8001,19507) | 30742 (25621,36790) | 29104 (24951,33904) | 15991 (13061,19531) | 23469 (20614,26992) |
| 5 | 28134 (22108,35425) | 31949 (26483,38655) | 39953 (33544,47079) | 49964 (33628,69459) | 18144 (11003,27075) | 43542 (36852,52055) | 41217 (35422,47683) | 22382 (18403,27338) | 33174 (28811,38168) |
| 6 | 25943 (20516,32907) | 29612 (24451,35534) | 36876 (31505,43514) | 45902 (30434,62347) | 16344 (10581,25741) | 40045 (34165,47133) | 38031 (32650,43944) | 20707 (16832,24969) | 30885 (26860,35582) |
| 7 | 14695 (11529,18512) | 16839 (13809,20190) | 20900 (17512,24543) | 25698 (17313,35340) | 9529 (5882,14347) | 22801 (19019,26804) | 21565 (18395,24742) | 11765 (9612,14330) | 17354 (15211,20104) |
| 8 | 18411 (14524,23811) | 21100 (17436,25580) | 26187 (22254,30880) | 32276 (22675,43967) | 11571 (7201,17757) | 28481 (23686,33765) | 27002 (23100,31179) | 14726 (12100,17706) | 21666 (19000,25178) |
| 9 | 12940 (9994,16237) | 14733 (12233,17564) | 18377 (15781,21514) | 22923 (15057,31011) | 8255 (5110,12867) | 19881 (16690,23557) | 18921 (16101,21992) | 10257 (8383,12477) | 15239 (13362,17548) |
| 10 | 12733 (9898,16023) | 14459 (12048,17275) | 18034 (15187,21095) | 22370 (15422,31328) | 7940 (4838,12622) | 19578 (16508,23157) | 18463 (15957,21464) | 10134 (8202,12386) | 14918 (13099,17057) |
| 11 | 12061 (9399,15186) | 13646 (11126,16441) | 17121 (14367,20233) | 21515 (13686,29034) | 7546 (4675,11744) | 18526 (15642,21916) | 17444 (15095,20199) | 9587 (7898,11624) | 14198 (12361,16463) |
| <12 | 19509 (15237,24195) | 22180 (18392,26714) | 27674 (23428,33106) | 34229 (22975,48094) | 12478 (7844,19484) | 30053 (25452,35326) | 28255 (24407,32873) | 15486 (12674,18992) | 22867 (19979,26259) |
| 12-14 | 10508 (8245,13379) | 11985 (9873,14378) | 14886 (12627,17769) | 18670 (12606,25178) | 6655 (4057,10463) | 16221 (13439,19027) | 15288 (12973,17863) | 8409 (6930,10178) | 12366 (10743,14257) |
| 15-17 | 10446 (7955,13193) | 11771 (9670,14040) | 14681 (12322,17486) | 18128 (11947,25034) | 6592 (4056,10382) | 15947 (13557,18863) | 15051 (12881,17580) | 8231 (6793,10032) | 12201 (10661,13981) |
| 18-20 | 9604 (7510,12053) | 10967 (9015,13196) | 13704 (11617,16194) | 17009 (11591,23676) | 6070 (3616,9557) | 14775 (12397,17525) | 13997 (12058,16308) | 7644 (6331,9263) | 11359 (9895,13045) |
| 21-23 | 4827 (3846,6042) | 5499 (4471,6516) | 6920 (5784,8094) | 8511 (5895,11578) | 3093 (1887,4682) | 7440 (6268,8813) | 7038 (6095,8189) | 3861 (3147,4730) | 5716 (4989,6527) |
| 12-23 | 8939 (7054,11077) | 10134 (8389,12202) | 12569 (10609,14813) | 15551 (10443,21071) | 5612 (3604,8657) | 13612 (11492,16343) | 12833 (11118,14834) | 7095 (5835,8541) | 10434 (8970,12000) |
| <24 | 13951 (10857,17186) | 15905 (13113,19104) | 19825 (16627,23290) | 24505 (16439,33971) | 8861 (5125,13979) | 21547 (18281,25557) | 20337 (17434,23453) | 11124 (9109,13551) | 16528 (14366,19054) |
| 24-35 | 4210 (3284,5258) | 4773 (3924,5872) | 5931 (4996,7104) | 7426 (4824,10266) | 2627 (1686,4054) | 6458 (5442,7618) | 6104 (5260,7177) | 3339 (2759,4109) | 4959 (4334,5717) |
| 36-47 | 2399 (1899,3024) | 2714 (2230,3261) | 3424 (2841,4063) | 4287 (2858,5846) | 1543 (907,2369) | 3679 (3119,4346) | 3491 (3018,4062) | 1914 (1551,2329) | 2840 (2493,3242) |
| 48-59 | 1418 (1106,1771) | 1612 (1325,1938) | 2019 (1704,2356) | 2506 (1690,3518) | 901 (522,1405) | 2186 (1826,2595) | 2071 (1781,2417) | 1130 (930,1367) | 1677 (1463,1930) |
| 24-59 | 2629 (2035,3276) | 2971 (2452,3562) | 3726 (3119,4400) | 4690 (3184,6384) | 1674 (1067,2644) | 4065 (3435,4804) | 3847 (3290,4441) | 2110 (1740,2628) | 3101 (2722,3572) |
| <60 | 7173 (5769,9092) | 8183 (6840,9811) | 10242 (8725,12271) | 12807 (8672,17535) | 4623 (2805,6837) | 11193 (9476,13179) | 10484 (9019,12229) | 5787 (4744,7043) | 8532 (7431,9829) |
| 2013 |  |  |  |  |  |  |  |  |  |
| <1 | 11105 (8625,13839) | 12684 (10524,15007) | 15861 (13425,18742) | 19729 (12926,26827) | 7131 (4306,11438) | 16989 (14269,20171) | 16319 (13825,19009) | 8889 (7322,10953) | 13151 (11401,14978) |
| 1 | 16949 (13146,21335) | 19180 (16073,23224) | 24066 (19984,28243) | 29687 (19627,40668) | 10700 (6755,16495) | 25868 (21751,30587) | 24640 (21134,28457) | 13462 (11091,16279) | 19873 (17319,23036) |
| 2 | 14137 (11214,18001) | 16245 (13509,19744) | 20359 (17079,24342) | 25537 (17708,35100) | 9069 (5776,14257) | 22139 (18471,26205) | 20665 (17927,24265) | 11386 (9477,13885) | 16845 (14620,19331) |
| 3 | 11415 (8989,14441) | 12852 (10737,15367) | 16069 (13499,19080) | 20066 (13725,27594) | 7325 (4675,10838) | 17535 (14763,20839) | 16521 (14375,19285) | 9041 (7616,11117) | 13465 (11665,15513) |
| 4 | 13298 (10362,16796) | 15084 (12609,18246) | 18800 (15747,22360) | 23346 (16179,32519) | 8402 (5230,12858) | 20587 (17083,23921) | 19339 (16585,22258) | 10601 (8506,12781) | 15719 (13713,18061) |
| 5 | 15783 (12343,20020) | 17892 (14767,21429) | 22431 (18901,26549) | 27513 (18625,38173) | 9993 (6365,15349) | 24104 (20475,28795) | 22962 (19801,26435) | 12564 (10269,15260) | 18555 (16113,21326) |
| 6 | 13394 (10551,16740) | 15223 (12477,18277) | 19216 (16124,22248) | 23803 (15850,32600) | 8525 (5256,12919) | 20593 (17139,24509) | 19466 (16854,22767) | 10798 (8776,12852) | 15809 (13677,18110) |
| 7 | 10156 (7932,12740) | 11539 (9448,13815) | 14413 (12164,17005) | 17787 (11816,24720) | 6331 (4034,10287) | 15629 (13158,18310) | 14707 (12786,17061) | 8134 (6690,9858) | 11947 (10392,13691) |
| 8 | 8769 (6890,10915) | 10008 (8274,12022) | 12530 (10398,14696) | 15522 (10172,20770) | 5595 (3509,8588) | 13584 (11435,16023) | 12870 (11007,14950) | 7027 (5739,8523) | 10364 (9028,11957) |
| 9 | 3999 (3169,5099) | 4542 (3758,5501) | 5718 (4877,6797) | 7151 (4857,9937) | 2556 (1584,4071) | 6196 (5251,7416) | 5885 (5062,6755) | 3213 (2633,3855) | 4773 (4139,5490) |
| 10 | 8691 (6748,10938) | 9826 (8094,11798) | 12249 (10432,14411) | 15184 (10106,20940) | 5491 (3470,8636) | 13358 (11235,15695) | 12610 (10917,14696) | 6934 (5674,8347) | 10208 (8883,11693) |
| 11 | 6406 (5025,8096) | 7319 (5996,8697) | 9101 (7703,10907) | 11256 (7618,15735) | 4075 (2544,6154) | 9860 (8322,11742) | 9336 (8015,10886) | 5091 (4149,6218) | 7567 (6640,8575) |
| <12 | 11229 (8893,14095) | 12846 (10662,15511) | 16172 (13502,19058) | 19892 (13077,27818) | 7170 (4453,10966) | 17441 (14647,20736) | 16462 (14206,19031) | 8965 (7346,10950) | 13386 (11613,15308) |
| 12-14 | 9029 (7109,11351) | 10349 (8543,12410) | 12852 (10807,15221) | 15978 (10789,22460) | 5761 (3487,8526) | 13958 (11771,16369) | 13129 (11262,15294) | 7269 (5929,8811) | 10714 (9468,12368) |
| 15-17 | 4418 (3473,5543) | 4972 (4123,5929) | 6261 (5255,7435) | 7761 (5277,10431) | 2840 (1722,4339) | 6780 (5733,8151) | 6414 (5474,7466) | 3497 (2867,4315) | 5199 (4484,5948) |
| 18-20 | 4802 (3802,6162) | 5503 (4532,6611) | 6852 (5792,8109) | 8483 (5628,11928) | 3055 (1947,4873) | 7476 (6347,8844) | 7051 (6032,8123) | 3858 (3159,4649) | 5717 (4970,6517) |
| 21-23 | 4854 (3719,6047) | 5463 (4473,6559) | 6851 (5757,8134) | 8543 (5878,11832) | 3060 (1780,4749) | 7393 (6247,8685) | 6991 (6050,8137) | 3845 (3121,4604) | 5679 (4948,6495) |
| 12-23 | 5837 (4586,7375) | 6622 (5501,7987) | 8281 (6946,9788) | 10301 (6914,14397) | 3678 (2159,5767) | 8901 (7502,10565) | 8523 (7337,9903) | 4664 (3836,5615) | 6831 (5996,7890) |
| <24 | 8618 (6773,10890) | 9728 (7958,11723) | 12273 (10323,14604) | 15204 (10491,20775) | 5445 (3413,8516) | 13197 (11223,15735) | 12478 (10877,14487) | 6891 (5616,8395) | 10190 (8859,11755) |
| 24-35 | 2538 (1991,3176) | 2854 (2372,3439) | 3602 (2989,4291) | 4442 (3003,6126) | 1630 (967,2467) | 3893 (3285,4597) | 3698 (3207,4245) | 2030 (1661,2466) | 2988 (2622,3422) |
| 36-47 | 1491 (1153,1907) | 1695 (1415,2038) | 2120 (1798,2549) | 2610 (1720,3605) | 957 (601,1472) | 2307 (1942,2728) | 2179 (1886,2546) | 1196 (972,1469) | 1767 (1532,2015) |
| 48-59 | 833 (656,1067) | 958 (791,1136) | 1185 (1002,1416) | 1462 (1010,1960) | 531 (324,827) | 1287 (1080,1504) | 1215 (1049,1409) | 671 (546,813) | 987 (854,1137) |
| 24-59 | 1627 (1284,2065) | 1850 (1524,2232) | 2312 (1934,2704) | 2903 (1919,4008) | 1034 (626,1620) | 2507 (2085,2944) | 2356 (2039,2743) | 1301 (1072,1556) | 1921 (1670,2219) |
| <60 | 4426 (3517,5594) | 5028 (4116,5997) | 6240 (5218,7316) | 7675 (5135,10537) | 2793 (1696,4313) | 6745 (5656,8015) | 6373 (5439,7432) | 3502 (2884,4286) | 5176 (4504,5974) |
| 2014 |  |  |  |  |  |  |  |  |  |
| <1 | 14164 (10978,17933) | 16084 (13312,19496) | 20023 (16977,23797) | 24817 (16560,33384) | 9127 (5574,13884) | 21908 (18267,25873) | 20641 (17684,23942) | 11326 (9317,13815) | 16669 (14460,19021) |
| 1 | 27454 (21453,34378) | 30952 (25343,37033) | 38810 (32624,46404) | 47866 (31676,65708) | 17165 (10838,26370) | 42044 (35663,49813) | 39888 (34001,46169) | 21712 (17810,26468) | 32279 (28184,36809) |
| 2 | 18073 (14403,23536) | 20402 (16859,25114) | 25674 (21499,30215) | 32127 (21768,44742) | 11440 (6949,17694) | 27864 (23306,32888) | 26497 (22996,30557) | 14389 (11793,17322) | 21375 (18610,24361) |
| 3 | 17044 (12888,21605) | 19029 (15850,23096) | 24037 (20315,28461) | 29598 (19638,40484) | 10659 (6396,16770) | 25953 (21874,30631) | 24571 (21248,28858) | 13544 (11216,16218) | 19946 (17314,23036) |
| 4 | 17906 (14208,22474) | 20538 (16973,24753) | 25760 (21745,30461) | 31348 (21592,44080) | 11665 (6782,17410) | 27671 (23175,32863) | 26143 (22588,30488) | 14262 (11786,17442) | 21273 (18611,24224) |
| 5 | 16652 (13040,20745) | 18853 (15280,22792) | 23571 (20081,27486) | 29239 (20173,40284) | 10558 (6465,16259) | 25521 (21438,30157) | 24079 (20775,27791) | 13216 (10568,16171) | 19545 (17029,22447) |
| 6 | 18386 (14416,23371) | 20940 (17306,25116) | 26203 (22109,30954) | 32524 (22545,44446) | 11746 (7368,18560) | 28492 (23616,33802) | 26772 (23206,31222) | 14835 (12283,17901) | 21826 (18980,24918) |
| 7 | 19637 (15284,24101) | 22227 (18403,26505) | 27708 (23034,32819) | 34397 (23261,48127) | 12408 (7623,19172) | 30019 (25316,35817) | 28324 (24596,32892) | 15492 (12668,18940) | 23095 (20046,26442) |
| 8 | 15041 (11882,19175) | 17067 (14049,20436) | 21371 (18229,25275) | 26616 (18277,37180) | 9538 (5883,15133) | 23226 (19306,27348) | 21820 (19024,25525) | 11954 (9814,14784) | 17825 (15572,20350) |
| 9 | 11039 (8567,14064) | 12591 (10311,15149) | 15747 (13106,18706) | 19683 (12980,27184) | 6987 (4231,10907) | 17063 (14514,20002) | 16150 (13963,18773) | 8831 (7234,10700) | 13070 (11322,15141) |
| 10 | 12333 (9700,15462) | 13984 (11405,16832) | 17588 (14844,21051) | 21700 (14499,30198) | 7781 (4855,11860) | 18968 (16019,22721) | 18038 (15447,20806) | 9858 (7993,12173) | 14648 (12788,16859) |
| 11 | 8836 (6835,10990) | 9930 (8293,11920) | 12423 (10562,14520) | 15406 (10202,21009) | 5515 (3374,8746) | 13510 (11556,16015) | 12686 (10958,14660) | 6961 (5760,8562) | 10375 (8986,11929) |
| <12 | 16331 (12924,20680) | 18607 (15345,22117) | 23202 (19389,27621) | 28858 (19732,40045) | 10504 (6339,16038) | 25092 (21102,29935) | 23839 (20543,27575) | 13078 (10738,15944) | 19349 (16879,22174) |
| 12-14 | 8378 (6474,10550) | 9532 (7953,11435) | 11972 (10003,14183) | 14647 (9864,20598) | 5331 (3362,8285) | 12935 (10809,15259) | 12193 (10591,14161) | 6739 (5486,8115) | 9881 (8577,11342) |
| 15-17 | 6977 (5504,8823) | 7923 (6555,9532) | 9941 (8277,11683) | 12326 (8416,16710) | 4431 (2765,6654) | 10741 (9074,12722) | 10111 (8727,11849) | 5539 (4545,6866) | 8248 (7229,9409) |
| 18-20 | 7652 (5877,9620) | 8676 (7087,10368) | 10870 (9149,12815) | 13468 (9032,18794) | 4805 (2873,7564) | 11759 (10005,13795) | 11131 (9635,12842) | 6105 (4956,7326) | 9015 (7832,10359) |
| 21-23 | 6401 (5109,7953) | 7354 (6068,8821) | 9245 (7792,10990) | 11334 (7474,15446) | 4099 (2470,6295) | 9916 (8521,11737) | 9394 (8188,10853) | 5143 (4194,6195) | 7628 (6613,8675) |
| 12-23 | 7343 (5834,9304) | 8359 (6984,10197) | 10473 (8764,12388) | 13222 (8822,17930) | 4762 (2930,7524) | 11367 (9617,13748) | 10786 (9237,12536) | 5875 (4745,7195) | 8737 (7556,10086) |
| <24 | 12051 (9402,15276) | 13755 (11446,16250) | 17165 (14402,20124) | 21524 (15015,29227) | 7685 (4518,12189) | 18612 (15833,22217) | 17591 (15289,20361) | 9688 (7921,11782) | 14272 (12383,16276) |
| 24-35 | 4433 (3491,5553) | 5055 (4166,6007) | 6284 (5339,7492) | 7866 (5319,10959) | 2817 (1758,4389) | 6832 (5741,8009) | 6446 (5506,7590) | 3503 (2862,4245) | 5257 (4500,6029) |
| 36-47 | 2420 (1908,3033) | 2736 (2271,3298) | 3415 (2892,4016) | 4248 (2832,5838) | 1533 (950,2445) | 3689 (3121,4338) | 3514 (3036,4057) | 1917 (1572,2350) | 2829 (2459,3262) |
| 48-59 | 1352 (1030,1681) | 1532 (1274,1828) | 1914 (1619,2269) | 2369 (1590,3240) | 874 (540,1381) | 2077 (1737,2433) | 1956 (1700,2285) | 1071 (870,1282) | 1590 (1400,1805) |
| 24-59 | 2720 (2146,3474) | 3114 (2557,3784) | 3886 (3274,4624) | 4753 (3171,6669) | 1731 (1154,2685) | 4201 (3590,4944) | 3974 (3387,4633) | 2168 (1800,2683) | 3210 (2812,3686) |
| <60 | 6574 (5177,8328) | 7421 (6177,8883) | 9311 (7901,10972) | 11500 (7624,16068) | 4221 (2579,6478) | 10057 (8537,11849) | 9455 (8148,11061) | 5209 (4271,6301) | 7715 (6730,8867) |
| 2015 |  |  |  |  |  |  |  |  |  |
| <1 | 9936 (7790,12589) | 11285 (9230,13424) | 14103 (12011,16727) | 17201 (11505,23593) | 6216 (3838,9452) | 15236 (12927,18033) | 14418 (12531,16875) | 7936 (6486,9608) | 11666 (10233,13381) |
| 1 | 27013 (20949,33772) | 30601 (25159,37165) | 38615 (32653,45472) | 47466 (33081,65289) | 17054 (10619,26436) | 41554 (34820,49161) | 39536 (33985,45705) | 21515 (17677,26289) | 31971 (27898,36807) |
| 2 | 21707 (16984,27050) | 24489 (20501,29293) | 30591 (25624,36510) | 38040 (25026,51795) | 13620 (8602,21178) | 33096 (27819,39306) | 31331 (27309,36249) | 17124 (13777,21032) | 25329 (21861,29162) |
| 3 | 17995 (13931,22430) | 20463 (17028,24362) | 25599 (21486,30331) | 31674 (21193,43423) | 11628 (6808,17848) | 27767 (23283,33196) | 26290 (22792,30904) | 14359 (11543,17433) | 21272 (18448,24407) |
| 4 | 16623 (13167,20917) | 19026 (15594,22748) | 23675 (19842,28081) | 29239 (19949,40359) | 10630 (6713,16379) | 25689 (21586,30083) | 24435 (20936,28440) | 13300 (10987,16053) | 19652 (17174,22593) |
| 5 | 18844 (14364,23452) | 21445 (17817,26119) | 26744 (22552,32141) | 33500 (22817,46259) | 12190 (7333,18499) | 28975 (24424,34108) | 27395 (23875,31644) | 14991 (12324,18558) | 22287 (19188,25597) |
| 6 | 16475 (12794,21000) | 18764 (15417,22588) | 23490 (19709,27665) | 28537 (18891,39267) | 10467 (6396,16003) | 25419 (21387,29915) | 23875 (20633,27797) | 13128 (10696,16034) | 19401 (16850,22552) |
| 7 | 12625 (9865,15790) | 14417 (11840,17528) | 17962 (15028,21260) | 22152 (15315,30647) | 7935 (5041,12446) | 19504 (16315,22900) | 18371 (15733,21074) | 10025 (8206,12163) | 14946 (12976,17257) |
| 8 | 11399 (8834,14530) | 13025 (10687,15624) | 16216 (13746,19403) | 20013 (13494,27298) | 7211 (4386,11286) | 17692 (14902,20656) | 16795 (14385,19427) | 9125 (7518,11103) | 13472 (11891,15656) |
| 9 | 13018 (10264,16201) | 14804 (12529,17874) | 18380 (15625,21916) | 23072 (15569,31120) | 8282 (5037,12716) | 20117 (17040,23464) | 18884 (16119,22138) | 10384 (8459,12568) | 15415 (13315,17585) |
| 10 | 13871 (10791,17589) | 15702 (13059,19166) | 19722 (16678,23209) | 24242 (17047,33102) | 8821 (5267,13520) | 21380 (18028,25273) | 20233 (17566,23565) | 11092 (9122,13718) | 16338 (14325,18847) |
| 11 | 9937 (7823,12624) | 11254 (9365,13549) | 14026 (11970,16783) | 17507 (11889,24064) | 6332 (3921,9949) | 15427 (12971,18163) | 14507 (12502,16981) | 7934 (6595,9568) | 11683 (10276,13344) |
| <12 | 15296 (12094,19088) | 17459 (14365,21163) | 21853 (18664,25349) | 27262 (17520,37329) | 9673 (6056,14916) | 23629 (19785,27691) | 22439 (19119,25966) | 12240 (9990,15022) | 18109 (15684,20806) |
| 12-14 | 10581 (8296,13420) | 12095 (9904,14597) | 15079 (12683,17957) | 18648 (12708,25513) | 6809 (4277,10404) | 16337 (13825,19121) | 15424 (13219,17947) | 8442 (6878,10233) | 12509 (10967,14435) |
| 15-17 | 7883 (6062,10144) | 8897 (7425,10820) | 11186 (9396,13363) | 13930 (9100,18978) | 4919 (3077,7775) | 12174 (10260,14290) | 11446 (9854,13200) | 6258 (5171,7538) | 9321 (8151,10698) |
| 18-20 | 8673 (6797,10911) | 9849 (8100,11791) | 12354 (10432,14546) | 15330 (10424,21120) | 5514 (3404,8721) | 13347 (11219,15776) | 12531 (10771,14591) | 6881 (5653,8323) | 10185 (9006,11758) |
| 21-23 | 4707 (3640,5862) | 5320 (4313,6453) | 6635 (5593,7873) | 8233 (5534,11348) | 2994 (1831,4573) | 7224 (5981,8546) | 6824 (5767,7837) | 3728 (3093,4552) | 5513 (4824,6338) |
| 12-23 | 8109 (6340,10373) | 9115 (7516,10898) | 11434 (9625,13376) | 14014 (9222,19276) | 5140 (3128,7929) | 12308 (10442,14506) | 11676 (10127,13602) | 6379 (5245,7810) | 9436 (8214,10761) |
| <24 | 11716 (9105,14960) | 13251 (11045,15789) | 16609 (14082,19709) | 20256 (14048,28432) | 7428 (4547,11122) | 17976 (15119,21268) | 16954 (14565,19734) | 9375 (7588,11234) | 13791 (12052,15801) |
| 24-35 | 3660 (2813,4596) | 4113 (3426,4919) | 5179 (4334,6092) | 6352 (4335,8841) | 2294 (1428,3650) | 5612 (4755,6672) | 5295 (4564,6168) | 2888 (2378,3527) | 4285 (3731,4960) |
| 36-47 | 3013 (2324,3772) | 3379 (2752,4065) | 4228 (3582,4970) | 5285 (3498,7300) | 1932 (1191,2945) | 4606 (3837,5487) | 4337 (3716,5025) | 2398 (1944,2867) | 3532 (3073,4045) |
| 48-59 | 1499 (1168,1898) | 1709 (1406,2030) | 2128 (1803,2495) | 2650 (1760,3630) | 960 (601,1507) | 2318 (1953,2712) | 2180 (1884,2514) | 1195 (988,1460) | 1770 (1551,2049) |
| 24-59 | 2672 (2111,3406) | 3068 (2504,3732) | 3812 (3187,4470) | 4721 (3078,6432) | 1694 (1018,2610) | 4129 (3530,4886) | 3896 (3356,4514) | 2133 (1749,2619) | 3171 (2776,3651) |
| <60 | 6354 (5011,8025) | 7218 (5893,8754) | 9000 (7651,10722) | 11109 (7497,15285) | 4073 (2635,6292) | 9838 (8202,11550) | 9233 (7981,10721) | 5044 (4194,6124) | 7516 (6508,8637) |
| 2016 |  |  |  |  |  |  |  |  |  |
| <1 | 19552 (15354,24665) | 22216 (18355,26786) | 27682 (23238,32866) | 33842 (22682,47726) | 12117 (7409,18781) | 30017 (25303,35502) | 28367 (24337,32954) | 15556 (12639,18971) | 22961 (19976,26115) |
| 1 | 33878 (26338,42249) | 38533 (31337,46427) | 48108 (40563,57256) | 59192 (38961,81961) | 21599 (13535,33472) | 52065 (43696,61459) | 49017 (42109,56800) | 27018 (22078,32882) | 39684 (34496,45505) |
| 2 | 23454 (18219,29894) | 26708 (22071,32243) | 33365 (28359,39789) | 41321 (27158,56654) | 14994 (9401,23170) | 36263 (30600,42908) | 34103 (29269,39278) | 18758 (15292,22611) | 27644 (24106,31798) |
| 3 | 20021 (15861,24954) | 22789 (19194,27208) | 28410 (24154,33835) | 35425 (24578,47789) | 12767 (7980,19863) | 30997 (25986,36719) | 29200 (25282,34061) | 15900 (13008,19478) | 23675 (20775,27017) |
| 4 | 9852 (7606,12390) | 11243 (9285,13665) | 14020 (11910,16688) | 17403 (11425,23536) | 6276 (3952,9654) | 15303 (12928,18066) | 14432 (12283,16810) | 7918 (6501,9612) | 11658 (10128,13312) |
| 5 | 17108 (13162,21698) | 19492 (16065,23371) | 24285 (20191,28745) | 30088 (20493,41588) | 11061 (6479,17200) | 26402 (21971,31074) | 24911 (21368,28881) | 13728 (11153,16423) | 20229 (17715,23171) |
| 6 | 14759 (11599,18675) | 16855 (14098,20301) | 21121 (17887,25029) | 26384 (17472,35904) | 9471 (5877,14548) | 22953 (19544,27251) | 21780 (18652,25280) | 11873 (9708,14472) | 17605 (15476,20215) |
| 7 | 11676 (9141,14686) | 13292 (11211,16115) | 16679 (14123,19641) | 20863 (13815,28093) | 7563 (4550,11654) | 18139 (15142,21496) | 17115 (14698,19955) | 9379 (7804,11527) | 13879 (12124,15852) |
| 8 | 17214 (13637,21888) | 19715 (16305,24070) | 24654 (20582,29540) | 30462 (20294,42834) | 10853 (6666,16982) | 26660 (22323,31494) | 25124 (21819,29329) | 13748 (11222,16858) | 20442 (17810,23492) |
| 9 | 10192 (8086,12837) | 11525 (9450,13916) | 14448 (12043,17140) | 17946 (11790,24610) | 6521 (3959,10049) | 15582 (13139,18387) | 14758 (12833,17127) | 8087 (6666,9806) | 11992 (10455,13883) |
| 10 | 8311 (6501,10282) | 9362 (7769,11247) | 11829 (9997,13921) | 14549 (9801,20160) | 5332 (3231,8338) | 12813 (10814,15177) | 12142 (10460,13918) | 6634 (5341,8078) | 9767 (8548,11208) |
| 11 | 4056 (3149,5059) | 4584 (3702,5410) | 5723 (4814,6790) | 7092 (4846,9693) | 2542 (1620,3941) | 6179 (5155,7334) | 5826 (5021,6846) | 3199 (2614,3857) | 4755 (4109,5504) |
| <12 | 15912 (12558,19971) | 18082 (15020,21723) | 22564 (18856,26595) | 28438 (18571,38845) | 10163 (6182,15493) | 24555 (20681,29050) | 23146 (20079,26843) | 12619 (10321,15330) | 18773 (16336,21618) |
| 12-14 | 7376 (5802,9284) | 8433 (6901,10183) | 10594 (8937,12404) | 12937 (8840,17488) | 4862 (3062,7448) | 11592 (9721,13566) | 10879 (9389,12620) | 5947 (4958,7148) | 8721 (7666,10017) |
| 15-17 | 8914 (6975,11286) | 10123 (8441,12159) | 12668 (10673,15065) | 15737 (10855,22228) | 5611 (3452,8701) | 13779 (11687,16255) | 13020 (11195,14937) | 7097 (5813,8566) | 10516 (9270,12118) |
| 18-20 | 5243 (4038,6674) | 5890 (4875,7063) | 7368 (6260,8637) | 9148 (6164,12512) | 3348 (2020,5230) | 8013 (6743,9553) | 7595 (6589,8698) | 4166 (3423,5068) | 6158 (5332,6984) |
| 21-23 | 6679 (5300,8496) | 7604 (6291,9191) | 9505 (8005,11183) | 11803 (7831,16415) | 4331 (2680,6604) | 10297 (8778,12103) | 9690 (8326,11266) | 5350 (4318,6530) | 7893 (6867,9063) |
| 12-23 | 7056 (5536,8845) | 8014 (6681,9478) | 10004 (8424,11809) | 12404 (8493,17381) | 4487 (2821,6946) | 10831 (9083,12923) | 10257 (8864,11942) | 5637 (4588,6803) | 8321 (7242,9542) |
| <24 | 11414 (9094,14212) | 12859 (10634,15408) | 16245 (13648,19058) | 20178 (13187,28475) | 7132 (4469,11318) | 17481 (14755,20591) | 16565 (14346,19091) | 9029 (7465,11046) | 13415 (11731,15465) |
| 24-35 | 3471 (2689,4378) | 3911 (3244,4711) | 4931 (4159,5848) | 6108 (3952,8194) | 2206 (1346,3375) | 5359 (4596,6357) | 5035 (4300,5886) | 2731 (2259,3342) | 4079 (3588,4650) |
| 36-47 | 2218 (1756,2810) | 2539 (2072,3090) | 3174 (2691,3746) | 3911 (2635,5437) | 1410 (872,2285) | 3448 (2897,4094) | 3254 (2826,3774) | 1792 (1454,2175) | 2636 (2311,3024) |
| 48-59 | 1466 (1150,1836) | 1668 (1382,2006) | 2095 (1765,2485) | 2589 (1723,3584) | 935 (559,1461) | 2264 (1896,2703) | 2128 (1852,2472) | 1172 (969,1444) | 1735 (1526,2008) |
| 24-59 | 2410 (1876,2990) | 2720 (2276,3318) | 3412 (2906,4080) | 4246 (2824,5966) | 1518 (947,2362) | 3709 (3088,4431) | 3477 (2994,4065) | 1916 (1581,2354) | 2837 (2483,3239) |
| <60 | 5945 (4652,7502) | 6784 (5572,8106) | 8451 (7133,10069) | 10345 (7067,14416) | 3811 (2341,5905) | 9129 (7627,10696) | 8642 (7420,9997) | 4759 (3906,5727) | 7001 (6134,8086) |
| 2017 |  |  |  |  |  |  |  |  |  |
| <1 | 13656 (10770,17228) | 15421 (12806,18375) | 19391 (16407,22904) | 23945 (16030,32657) | 8683 (5319,13820) | 21145 (17579,25065) | 19894 (17157,23046) | 10934 (8917,13354) | 16144 (14203,18636) |
| 1 | 17028 (13182,21727) | 19587 (16212,23425) | 24460 (20448,28946) | 30125 (20319,41591) | 11054 (6603,17042) | 26420 (22013,31514) | 24888 (21671,28674) | 13627 (11308,16467) | 20277 (17560,23027) |
| 2 | 9862 (7614,12441) | 11096 (9266,13129) | 13913 (11762,16495) | 17280 (11902,23941) | 6283 (3789,9760) | 15102 (12740,17852) | 14237 (12296,16574) | 7786 (6379,9396) | 11590 (10123,13256) |
| 3 | 11912 (9373,15016) | 13490 (11305,16154) | 16879 (14426,19805) | 21215 (14195,29000) | 7603 (4643,11978) | 18452 (15595,21703) | 17278 (14671,20129) | 9579 (7799,11558) | 14067 (12228,16263) |
| 4 | 9140 (7165,11467) | 10405 (8529,12576) | 13018 (11066,15428) | 16183 (10657,22475) | 5847 (3727,8922) | 14199 (11819,16716) | 13394 (11623,15507) | 7293 (5979,8875) | 10816 (9479,12431) |
| 5 | 6632 (5058,8274) | 7539 (6289,8954) | 9456 (7900,11182) | 11627 (8033,16112) | 4214 (2593,6575) | 10194 (8601,11928) | 9689 (8410,11216) | 5315 (4301,6424) | 7816 (6854,8979) |
| 6 | 5898 (4541,7443) | 6702 (5593,8060) | 8384 (7035,9884) | 10448 (7110,14213) | 3689 (2307,5936) | 9120 (7639,10830) | 8624 (7354,9848) | 4661 (3837,5679) | 6955 (6123,8038) |
| 7 | 6821 (5382,8573) | 7739 (6340,9285) | 9668 (8118,11374) | 11851 (7933,16450) | 4294 (2605,6595) | 10471 (8792,12353) | 9858 (8452,11557) | 5425 (4421,6639) | 8012 (7020,9296) |
| 8 | 5979 (4689,7595) | 6792 (5550,8259) | 8527 (7188,10038) | 10454 (7055,14357) | 3815 (2457,5787) | 9291 (7641,11014) | 8739 (7505,10193) | 4791 (3950,5796) | 7072 (6163,8143) |
| 9 | 6599 (5196,8305) | 7513 (6224,9032) | 9318 (7869,11124) | 11668 (8007,16260) | 4140 (2609,6385) | 10159 (8599,11881) | 9617 (8294,11276) | 5202 (4285,6345) | 7764 (6861,8885) |
| 10 | 5822 (4550,7209) | 6661 (5508,8012) | 8339 (6953,9811) | 10337 (6854,14089) | 3688 (2238,5815) | 9013 (7569,10520) | 8529 (7284,9837) | 4656 (3815,5703) | 6941 (6032,7932) |
| 11 | 5794 (4482,7281) | 6528 (5417,7930) | 8209 (6888,9758) | 10158 (6814,13955) | 3620 (2223,5686) | 8970 (7460,10517) | 8450 (7249,9825) | 4609 (3803,5680) | 6801 (5953,7797) |
| <12 | 9021 (7045,11585) | 10197 (8476,12221) | 12829 (10695,15303) | 15696 (10529,22012) | 5707 (3603,8966) | 13868 (11648,16393) | 13076 (11354,15322) | 7148 (5842,8491) | 10659 (9179,12145) |
| 12-14 | 4603 (3538,5833) | 5157 (4279,6152) | 6519 (5464,7692) | 8025 (5569,11191) | 2936 (1826,4418) | 7056 (6011,8290) | 6675 (5773,7710) | 3670 (2994,4359) | 5395 (4732,6182) |
| 15-17 | 3409 (2687,4305) | 3864 (3186,4617) | 4844 (4114,5726) | 5993 (4066,8175) | 2154 (1344,3357) | 5244 (4384,6212) | 4990 (4266,5778) | 2698 (2228,3313) | 4038 (3500,4614) |
| 18-20 | 2740 (2116,3376) | 3097 (2547,3723) | 3859 (3279,4529) | 4820 (3190,6585) | 1732 (1039,2671) | 4221 (3559,5008) | 3967 (3432,4617) | 2178 (1797,2614) | 3221 (2790,3714) |
| 21-23 | 2245 (1762,2855) | 2552 (2148,3098) | 3207 (2664,3776) | 3940 (2688,5367) | 1417 (881,2201) | 3487 (2954,4095) | 3290 (2842,3845) | 1805 (1450,2195) | 2662 (2323,3040) |
| 12-23 | 3229 (2529,4052) | 3714 (3111,4422) | 4607 (3866,5429) | 5655 (3899,7966) | 2048 (1215,3178) | 4976 (4254,5934) | 4694 (4094,5461) | 2607 (2110,3132) | 3816 (3329,4381) |
| <24 | 6080 (4827,7649) | 6984 (5791,8385) | 8666 (7343,10330) | 10781 (7174,15091) | 3918 (2401,5899) | 9476 (7980,11156) | 8930 (7742,10305) | 4890 (3987,5928) | 7200 (6337,8341) |
| 24-35 | 1853 (1465,2318) | 2098 (1751,2489) | 2632 (2203,3092) | 3278 (2222,4534) | 1180 (746,1867) | 2840 (2408,3359) | 2687 (2327,3161) | 1484 (1230,1789) | 2178 (1901,2506) |
| 36-47 | 1164 (914,1443) | 1310 (1100,1564) | 1638 (1367,1926) | 2035 (1354,2881) | 733 (458,1132) | 1780 (1506,2108) | 1694 (1453,1937) | 924 (756,1122) | 1368 (1176,1571) |
| 48-59 | 686 (534,894) | 785 (643,941) | 977 (829,1159) | 1199 (812,1662) | 435 (267,690) | 1061 (892,1256) | 998 (861,1171) | 549 (446,659) | 807 (700,918) |
| 24-59 | 1228 (971,1560) | 1410 (1147,1671) | 1764 (1492,2082) | 2165 (1505,2935) | 777 (482,1249) | 1902 (1617,2279) | 1798 (1536,2099) | 991 (804,1190) | 1459 (1282,1669) |
| <60 | 3204 (2452,4041) | 3620 (2992,4366) | 4521 (3798,5266) | 5585 (3696,7898) | 2015 (1285,3248) | 4873 (4163,5749) | 4627 (4005,5325) | 2539 (2087,3072) | 3727 (3279,4255) |
| 2018 |  |  |  |  |  |  |  |  |  |
| <1 | 34653 (27390,42684) | 39455 (32723,47721) | 49480 (41496,58129) | 61598 (40016,84990) | 22122 (13590,33886) | 53321 (44563,63551) | 50371 (43293,58148) | 27587 (22596,33454) | 41165 (35893,47148) |
| 1 | 28255 (22582,35872) | 32214 (26785,38537) | 40168 (34108,48033) | 49769 (33585,67623) | 18040 (10960,28263) | 43284 (36802,52340) | 41412 (35529,47963) | 22757 (18588,27562) | 33581 (29234,38538) |
| 2 | 17132 (13600,21584) | 19533 (16232,23556) | 24480 (20602,28963) | 30149 (19790,41243) | 10886 (6550,17353) | 26575 (22826,31243) | 24790 (21287,29075) | 13667 (11216,16741) | 20297 (17717,23166) |
| 3 | 19099 (15082,23832) | 21596 (17968,25891) | 27086 (22524,31893) | 33029 (22264,45289) | 12149 (7437,18398) | 29225 (24741,34766) | 27645 (24060,32170) | 15041 (12233,18112) | 22284 (19367,25577) |
| 4 | 21646 (17091,27253) | 24493 (20244,29279) | 30631 (25568,36110) | 37671 (25241,51496) | 13589 (8259,20916) | 33115 (27704,39855) | 31428 (26823,36255) | 17178 (13837,21039) | 25359 (22243,29308) |
| 5 | 16792 (13147,21146) | 19014 (15728,22907) | 24048 (20192,28087) | 29501 (20373,40672) | 10697 (6147,16455) | 26007 (21975,30747) | 24576 (21102,28850) | 13321 (10907,16279) | 19900 (17382,22767) |
| 6 | 15758 (12598,19630) | 17851 (14876,21268) | 22519 (18926,26584) | 28034 (19127,38014) | 9969 (6238,15508) | 24292 (20417,28895) | 22960 (19613,26477) | 12558 (10258,15317) | 18664 (16208,21575) |
| 7 | 12766 (10023,15942) | 14361 (11951,17393) | 18111 (14991,21478) | 22602 (15507,30923) | 8157 (4944,12298) | 19597 (16483,23231) | 18585 (15808,21718) | 10193 (8263,12315) | 15028 (13105,17269) |
| 8 | 18111 (14222,22527) | 20677 (17095,24591) | 25883 (21658,30740) | 32058 (22208,43860) | 11548 (6942,17529) | 27940 (23693,32866) | 26406 (22678,30545) | 14487 (11745,17592) | 21450 (18780,24670) |
| 9 | 11448 (9078,14606) | 12999 (10797,15700) | 16374 (13599,19425) | 20310 (13553,27742) | 7284 (4631,11607) | 17815 (14740,20883) | 16806 (14371,19376) | 9167 (7480,11038) | 13491 (11779,15546) |
| 10 | 12662 (9710,15707) | 14335 (11885,17373) | 18097 (15169,21031) | 22448 (14993,30480) | 7979 (5000,12409) | 19442 (16361,23061) | 18430 (16062,21399) | 10160 (8296,12203) | 14919 (12983,17106) |
| 11 | 9504 (7353,11921) | 10726 (8820,12878) | 13349 (11190,15920) | 16661 (11026,23100) | 6001 (3667,9492) | 14602 (12408,17243) | 13728 (11733,16145) | 7461 (6160,9216) | 11181 (9790,12756) |
| <12 | 17667 (13759,22317) | 20159 (16451,24352) | 25124 (21225,29883) | 31231 (21309,42209) | 11105 (6668,17261) | 27261 (23128,31853) | 25806 (21967,30167) | 14042 (11628,17280) | 20980 (18206,23984) |
| 12-14 | 7486 (5823,9491) | 8518 (6933,10228) | 10564 (8980,12646) | 13205 (8739,17993) | 4805 (2913,7436) | 11615 (9753,13766) | 10937 (9376,12644) | 5974 (4915,7221) | 8864 (7745,10082) |
| 15-17 | 6250 (4861,7883) | 7138 (5857,8540) | 8939 (7604,10630) | 11214 (7638,15245) | 4008 (2422,5972) | 9704 (8046,11570) | 9136 (7945,10594) | 5026 (4128,6163) | 7429 (6488,8565) |
| 18-20 | 7814 (6040,9896) | 8901 (7304,10689) | 11163 (9438,13184) | 13634 (9295,19312) | 4925 (3083,7671) | 12079 (10019,14204) | 11379 (9866,13124) | 6256 (5151,7485) | 9232 (8077,10514) |
| 21-23 | 5408 (4246,6717) | 6104 (4998,7402) | 7726 (6512,9118) | 9560 (6264,13029) | 3505 (2084,5316) | 8320 (7031,9792) | 7896 (6832,9152) | 4304 (3535,5222) | 6390 (5545,7285) |
| 12-23 | 6775 (5337,8498) | 7726 (6409,9227) | 9620 (8133,11385) | 11976 (8227,16377) | 4323 (2639,6648) | 10477 (8824,12470) | 9874 (8456,11516) | 5399 (4443,6621) | 8034 (6878,9300) |
| <24 | 12087 (9645,15277) | 13625 (11223,16275) | 17236 (14553,20331) | 21167 (14396,28805) | 7724 (4789,11547) | 18594 (15847,21645) | 17640 (15051,20538) | 9590 (7896,11554) | 14245 (12413,16618) |
| 24-35 | 4442 (3508,5640) | 5051 (4174,6108) | 6352 (5453,7525) | 7987 (5424,10922) | 2854 (1835,4596) | 6924 (5779,8197) | 6572 (5662,7641) | 3552 (2890,4291) | 5281 (4561,6061) |
| 36-47 | 2514 (1975,3188) | 2837 (2348,3427) | 3551 (2975,4178) | 4391 (2920,6050) | 1586 (1013,2418) | 3871 (3275,4582) | 3647 (3151,4235) | 2001 (1607,2447) | 2951 (2572,3363) |
| 48-59 | 1656 (1340,2092) | 1876 (1573,2276) | 2357 (1975,2795) | 2947 (1928,3980) | 1063 (674,1699) | 2561 (2147,3008) | 2416 (2090,2794) | 1327 (1074,1629) | 1962 (1714,2247) |
| 24-59 | 2876 (2247,3585) | 3269 (2683,3921) | 4105 (3460,4884) | 5067 (3353,7004) | 1822 (1067,2898) | 4433 (3740,5228) | 4190 (3617,4878) | 2300 (1875,2803) | 3393 (2975,3909) |
| <60 | 6482 (5103,8251) | 7331 (6026,8876) | 9164 (7738,10841) | 11394 (7548,15557) | 4067 (2616,6378) | 9934 (8442,11683) | 9340 (8108,11070) | 5183 (4206,6297) | 7617 (6656,8663) |

**Table S9: Annual rates of RSV associated non-hospitalized severe acute respiratory illness per 100,000 children by regions and age in months, 2010-2018**

| Year and Age in months | Central Rate (95% CI) | Coast Rate (95% CI) | Eastern Rate (95% CI) | North Eastern Rate (95% CI) | Nairobi Rate (95% CI) | Nyanza Rate (95% CI) | Rift Valley Rate (95% CI) | Western Rate (95% CI) | Kenya Rate (95% CI) |
| --- | --- | --- | --- | --- | --- | --- | --- | --- | --- |
| 2010-2018 |  |  |  |  |  |  |  |  |  |
| <1 | 3988 (2624,5813) | 6312 (4972,8031) | 8831 (7224,10513) | 10886 (7260,15412) | 2551 (1455,4434) | 7043 (5028,9330) | 5829 (4031,8189) | 3811 (2800,5144) | 7302 (6301,8489) |
| 1 | 5816 (3797,8595) | 12383 (9846,15299) | 12725 (10517,15175) | 15865 (10271,22347) | 3643 (2033,6389) | 10140 (7237,13770) | 8468 (5828,11686) | 5529 (4105,7257) | 10553 (8983,12382) |
| 2 | 4238 (2752,6045) | 8143 (6607,10187) | 9359 (7744,11202) | 11650 (7686,16191) | 2604 (1472,4549) | 7367 (5235,9698) | 6090 (4225,8464) | 4005 (2836,5444) | 7692 (6611,8986) |
| 3 | 2509 (1734,3501) | 6160 (4834,7837) | 5619 (4630,6831) | 6935 (4643,9740) | 1608 (942,2652) | 4209 (3120,5513) | 3665 (2742,4845) | 2348 (1725,3032) | 4679 (4004,5524) |
| 4 | 2276 (1565,3183) | 5152 (3950,6636) | 5105 (4233,6167) | 6423 (4215,8766) | 1446 (831,2459) | 3863 (2897,4993) | 3312 (2341,4439) | 2086 (1538,2836) | 4234 (3607,4928) |
| 5 | 2494 (1670,3539) | 5317 (4004,6967) | 5646 (4658,6749) | 6926 (4597,9793) | 1571 (918,2647) | 4203 (3157,5512) | 3643 (2658,4840) | 2334 (1695,3123) | 4659 (3975,5450) |
| 6 | 2069 (1414,2867) | 4048 (3038,5357) | 3927 (3230,4790) | 4920 (3178,6907) | 1310 (745,2210) | 4038 (3082,5315) | 3016 (2177,4017) | 2223 (1639,2868) | 3246 (2706,3917) |
| 7 | 1784 (1218,2495) | 3190 (2326,4378) | 3360 (2774,4122) | 4141 (2808,5907) | 1147 (635,1892) | 3451 (2611,4582) | 2601 (1828,3475) | 1888 (1428,2500) | 2825 (2357,3353) |
| 8 | 1671 (1106,2273) | 2258 (1601,3218) | 3172 (2589,3903) | 3937 (2688,5558) | 1046 (597,1764) | 3260 (2424,4276) | 2431 (1752,3219) | 1782 (1328,2328) | 2632 (2203,3156) |
| 9 | 1170 (788,1677) | 1645 (1103,2368) | 1915 (1510,2356) | 2338 (1566,3321) | 738 (400,1342) | 2034 (1452,2798) | 1764 (1183,2431) | 1082 (759,1483) | 1583 (1287,1907) |
| 10 | 1321 (850,1993) | 1746 (1158,2492) | 2108 (1710,2648) | 2623 (1724,3737) | 828 (446,1450) | 2296 (1664,3058) | 1918 (1250,2692) | 1197 (829,1619) | 1773 (1465,2115) |
| 11 | 1076 (662,1564) | 1276 (815,1904) | 1744 (1377,2163) | 2157 (1435,3067) | 673 (373,1134) | 1883 (1310,2525) | 1588 (1091,2212) | 997 (699,1344) | 1434 (1202,1749) |
| <12 | 2177 (1667,2872) | 4921 (4012,5920) | 4863 (4035,5808) | 6010 (4015,8416) | 1403 (870,2224) | 3943 (3180,4875) | 3222 (2582,3915) | 2120 (1674,2675) | 4020 (3461,4650) |
| 12-14 | 721 (411,1101) | 1098 (815,1456) | 1415 (1119,1799) | 1745 (1165,2579) | 453 (238,826) | 1908 (1307,2580) | 1048 (650,1532) | 940 (630,1331) | 1183 (958,1439) |
| 15-17 | 590 (277,1040) | 727 (514,1025) | 1065 (810,1375) | 1321 (870,1951) | 375 (158,758) | 1399 (937,2011) | 863 (376,1521) | 730 (491,1056) | 878 (689,1118) |
| 18-20 | 968 (553,1568) | 749 (517,1032) | 1184 (909,1530) | 1477 (926,2170) | 608 (319,1134) | 1367 (874,1975) | 1431 (815,2184) | 758 (497,1097) | 982 (769,1233) |
| 21-23 | 922 (468,1502) | 343 (203,535) | 679 (490,920) | 851 (495,1258) | 570 (259,1095) | 826 (465,1255) | 1331 (712,2058) | 406 (218,627) | 567 (412,736) |
| 12-23 | 756 (539,1052) | 737 (589,914) | 1096 (888,1321) | 1367 (902,1909) | 484 (279,786) | 1381 (1075,1756) | 1090 (802,1435) | 711 (557,919) | 907 (766,1083) |
| <24 | 1464 (1112,1889) | 2815 (2318,3481) | 2930 (2458,3494) | 3642 (2443,5007) | 941 (583,1449) | 2586 (2120,3146) | 2156 (1747,2609) | 1388 (1116,1732) | 2439 (2117,2804) |
| 24-35 | 391 (242,574) | 270 (196,366) | 450 (356,564) | 560 (365,793) | 253 (134,437) | 622 (460,831) | 574 (372,821) | 302 (220,415) | 372 (304,456) |
| 36-47 | 197 (96,343) | 103 (63,157) | 214 (157,286) | 262 (169,388) | 124 (51,241) | 370 (242,541) | 286 (147,475) | 167 (109,241) | 179 (133,231) |
| 48-59 | 27 (0,71) | 14 (0,32) | 49 (27,75) | 61 (32,101) | 17 (0,49) | 99 (42,171) | 43 (0,104) | 48 (24,80) | 41 (23,63) |
| 24-59 | 199 (132,282) | 130 (95,173) | 235 (188,287) | 287 (196,407) | 123 (74,216) | 360 (278,461) | 289 (199,392) | 171 (129,224) | 195 (161,236) |
| <60 | 712 (545,939) | 1206 (988,1467) | 1292 (1091,1531) | 1619 (1073,2227) | 456 (280,710) | 1234 (1013,1486) | 1027 (841,1234) | 627 (511,789) | 1077 (934,1247) |
| 2010 |  |  |  |  |  |  |  |  |  |
| <1 | 7496 (5066,10976) | 14716 (11141,19171) | 16618 (13974,20006) | 20670 (13714,28845) | 4853 (2672,8445) | 14166 (9201,20922) | 11086 (7459,15293) | 7139 (5141,9790) | 13779 (11997,16088) |
| 1 | 7587 (5103,10941) | 14699 (11382,18652) | 16657 (13903,19683) | 20755 (13435,29048) | 4755 (2548,7876) | 14589 (8862,21082) | 11146 (7680,15351) | 7157 (5078,9792) | 13780 (11797,16138) |
| 2 | 6350 (4256,9220) | 12316 (9611,15685) | 13807 (11493,16753) | 17499 (11135,24476) | 3961 (2141,6906) | 12094 (7884,17116) | 9386 (6389,12974) | 6002 (4318,8204) | 11602 (9874,13547) |
| 3 | 3656 (2503,5103) | 8136 (5752,11305) | 8288 (6813,9987) | 10250 (6785,14212) | 2345 (1378,3788) | 6403 (4262,9165) | 5327 (3917,7123) | 3384 (2553,4479) | 6841 (5768,8044) |
| 4 | 2753 (1916,3828) | 6098 (4328,8316) | 6170 (5134,7456) | 7753 (5163,10628) | 1762 (1010,2839) | 4828 (3105,6870) | 4032 (2934,5428) | 2570 (1889,3430) | 5139 (4345,6107) |
| 5 | 3651 (2495,5005) | 8002 (5647,10804) | 8135 (6730,9801) | 10117 (6955,14305) | 2301 (1365,3759) | 6291 (3918,9011) | 5302 (3919,6849) | 3394 (2464,4595) | 6760 (5755,7949) |
| 6 | 3702 (2494,5113) | 5595 (3520,8221) | 6914 (5603,8344) | 8448 (5394,11922) | 2292 (1300,3929) | 5609 (3260,8585) | 5333 (3788,7435) | 3848 (2878,5056) | 5714 (4802,6856) |
| 7 | 3343 (2276,4666) | 5114 (3235,7688) | 6363 (5190,7621) | 7874 (5269,10958) | 2083 (1163,3491) | 5302 (3055,8209) | 4876 (3597,6645) | 3564 (2659,4775) | 5220 (4432,6237) |
| 8 | 2090 (1446,3009) | 3244 (2009,4702) | 3944 (3260,4735) | 4890 (3223,6723) | 1316 (775,2168) | 3259 (1863,4828) | 3050 (2175,4102) | 2201 (1650,2937) | 3291 (2717,3984) |
| 9 | 2406 (1539,3589) | 2749 (1640,4182) | 3859 (3085,4820) | 4785 (3208,6863) | 1515 (830,2607) | 4149 (2119,6823) | 3502 (2393,5056) | 2167 (1545,3020) | 3179 (2596,3850) |
| 10 | 2786 (1788,4011) | 3179 (1801,5077) | 4441 (3559,5614) | 5557 (3708,7858) | 1705 (995,3106) | 4880 (2420,7772) | 4026 (2654,5774) | 2550 (1825,3456) | 3699 (3061,4528) |
| 11 | 2282 (1458,3400) | 2618 (1455,3942) | 3715 (2988,4588) | 4587 (3064,6405) | 1434 (791,2373) | 4005 (1981,6370) | 3335 (2185,4666) | 2120 (1475,2852) | 3069 (2492,3707) |
| <12 | 3555 (2613,4652) | 7469 (5956,9399) | 7780 (6550,9363) | 9653 (6536,13349) | 2249 (1326,3632) | 6365 (4842,8211) | 5177 (4185,6231) | 3383 (2696,4239) | 6426 (5533,7492) |
| 12-14 | 1213 (724,1809) | 2257 (1185,3619) | 2314 (1866,2899) | 2868 (1895,4153) | 748 (398,1358) | 3298 (1590,5459) | 1743 (1060,2562) | 1580 (1114,2170) | 1939 (1554,2382) |
| 15-17 | 1042 (405,1823) | 2073 (1061,3160) | 1796 (1338,2315) | 2216 (1408,3263) | 630 (228,1244) | 1861 (636,3687) | 1469 (661,2504) | 1236 (813,1824) | 1497 (1151,1924) |
| 18-20 | 1541 (839,2471) | 1552 (610,2650) | 1860 (1414,2396) | 2300 (1482,3329) | 954 (446,1810) | 3633 (1689,6028) | 2225 (1238,3437) | 1190 (781,1720) | 1550 (1183,1942) |
| 21-23 | 1169 (608,1946) | 603 (139,1281) | 869 (632,1160) | 1085 (667,1619) | 727 (350,1335) | 916 (224,1811) | 1671 (911,2699) | 522 (297,782) | 725 (529,957) |
| 12-23 | 1180 (825,1609) | 1619 (1161,2209) | 1712 (1434,2070) | 2161 (1441,2951) | 738 (433,1253) | 2396 (1613,3307) | 1734 (1299,2218) | 1119 (837,1437) | 1426 (1196,1685) |
| <24 | 2264 (1721,2932) | 4356 (3489,5459) | 4574 (3776,5446) | 5627 (3596,7860) | 1444 (901,2282) | 4259 (3343,5331) | 3317 (2712,3981) | 2148 (1735,2701) | 3768 (3291,4345) |
| 24-35 | 607 (400,925) | 390 (166,681) | 697 (559,862) | 864 (557,1198) | 390 (207,678) | 793 (341,1355) | 898 (584,1279) | 472 (347,646) | 580 (473,713) |
| 36-47 | 350 (169,613) | 123 (0,301) | 385 (278,512) | 473 (297,693) | 220 (91,422) | 402 (95,860) | 507 (239,832) | 297 (192,442) | 322 (240,414) |
| 48-59 | 47 (0,114) | 41 (0,136) | 84 (45,130) | 102 (53,176) | 30 (0,81) | 278 (0,648) | 75 (0,170) | 84 (39,141) | 69 (40,104) |
| 24-59 | 330 (223,468) | 187 (98,308) | 389 (316,487) | 487 (317,680) | 209 (118,351) | 499 (286,808) | 480 (345,651) | 285 (216,362) | 325 (266,386) |
| <60 | 1112 (828,1453) | 1833 (1468,2274) | 2034 (1692,2410) | 2523 (1695,3501) | 716 (433,1113) | 2052 (1601,2615) | 1613 (1348,1958) | 998 (796,1222) | 1687 (1465,1976) |
| 2011 |  |  |  |  |  |  |  |  |  |
| <1 | 5387 (3526,7655) | 12882 (9884,16419) | 11878 (9836,14420) | 14684 (9200,20204) | 3470 (1963,5951) | 6178 (2187,11918) | 7959 (5407,10972) | 5129 (3644,6838) | 9852 (8455,11469) |
| 1 | 6091 (4018,8873) | 14338 (11230,18120) | 13267 (11092,15954) | 16453 (10847,22668) | 3899 (2198,6500) | 6982 (2297,13513) | 8830 (6025,12418) | 5723 (4142,7658) | 11000 (9477,13017) |
| 2 | 4525 (2903,6495) | 10660 (8234,13448) | 9907 (8282,11973) | 12323 (8357,17068) | 2791 (1559,4813) | 5068 (1741,10037) | 6636 (4606,9200) | 4307 (3009,5806) | 8189 (7001,9589) |
| 3 | 3822 (2610,5344) | 10114 (7731,13303) | 8538 (7087,10419) | 10529 (7070,15035) | 2421 (1434,4102) | 5330 (2279,9078) | 5619 (4044,7326) | 3525 (2608,4575) | 7101 (6033,8477) |
| 4 | 3017 (2070,4213) | 8102 (5741,10513) | 6778 (5532,8174) | 8363 (5505,11559) | 1880 (1076,3068) | 4149 (1735,7151) | 4388 (3272,5794) | 2780 (2060,3662) | 5562 (4745,6589) |
| 5 | 2914 (2008,3996) | 7671 (5776,10154) | 6488 (5292,7806) | 7979 (5386,11161) | 1809 (1056,3025) | 3956 (1744,6702) | 4238 (3055,5724) | 2684 (1940,3501) | 5364 (4588,6329) |
| 6 | 2850 (1970,4040) | 5821 (3888,8340) | 5364 (4386,6526) | 6673 (4501,9174) | 1773 (1051,3029) | 4701 (2220,7906) | 4113 (2944,5692) | 3002 (2271,3996) | 4507 (3764,5331) |
| 7 | 3116 (2131,4308) | 6358 (4153,8973) | 5931 (4798,7275) | 7312 (4730,10362) | 1983 (1089,3342) | 5286 (2413,8504) | 4574 (3268,6002) | 3284 (2477,4304) | 4900 (4076,5807) |
| 8 | 2003 (1325,2773) | 4095 (2657,5710) | 3775 (3058,4574) | 4606 (3024,6718) | 1256 (700,2098) | 3355 (1522,5517) | 2892 (2101,3899) | 2115 (1578,2809) | 3104 (2644,3698) |
| 9 | 1214 (791,1771) | 2256 (1332,3475) | 1959 (1592,2436) | 2438 (1614,3485) | 773 (433,1356) | 1839 (838,3070) | 1779 (1194,2522) | 1133 (787,1520) | 1642 (1341,1960) |
| 10 | 1411 (919,2111) | 2583 (1515,3842) | 2272 (1786,2804) | 2849 (1854,3936) | 882 (475,1538) | 2116 (961,3620) | 2061 (1400,2840) | 1281 (896,1734) | 1900 (1554,2304) |
| 11 | 1591 (1004,2363) | 2869 (1649,4378) | 2519 (2004,3162) | 3127 (1988,4456) | 967 (528,1768) | 2374 (993,4098) | 2292 (1550,3246) | 1437 (998,1949) | 2109 (1741,2564) |
| <12 | 2764 (2056,3629) | 7482 (5977,9406) | 6127 (5099,7295) | 7478 (4967,10321) | 1737 (1060,2810) | 3948 (2744,5539) | 4051 (3259,4940) | 2673 (2119,3336) | 5046 (4397,5809) |
| 12-14 | 796 (476,1219) | 1473 (678,2575) | 1552 (1210,1928) | 1906 (1249,2739) | 492 (267,869) | 1529 (543,2821) | 1147 (736,1660) | 1045 (749,1454) | 1290 (1037,1584) |
| 15-17 | 654 (274,1159) | 1051 (354,1979) | 1152 (882,1467) | 1434 (877,2055) | 420 (171,797) | 1538 (590,2777) | 957 (452,1633) | 805 (537,1145) | 965 (742,1209) |
| 18-20 | 1537 (837,2531) | 1207 (377,2328) | 1903 (1458,2476) | 2337 (1458,3498) | 962 (461,1797) | 1975 (425,4053) | 2228 (1279,3570) | 1203 (798,1718) | 1569 (1221,1991) |
| 21-23 | 1282 (647,2156) | 657 (159,1477) | 970 (698,1293) | 1196 (718,1791) | 788 (360,1516) | 2067 (711,4029) | 1888 (971,2971) | 572 (322,896) | 802 (587,1076) |
| 12-23 | 944 (667,1316) | 1135 (728,1669) | 1398 (1140,1666) | 1720 (1167,2410) | 604 (347,981) | 1745 (1107,2563) | 1386 (1040,1796) | 903 (699,1159) | 1157 (968,1372) |
| <24 | 1887 (1417,2451) | 4452 (3575,5542) | 3751 (3134,4502) | 4636 (3173,6340) | 1175 (734,1893) | 2921 (2176,3902) | 2753 (2264,3315) | 1793 (1422,2224) | 3116 (2682,3588) |
| 24-35 | 463 (282,688) | 509 (254,829) | 532 (418,663) | 661 (441,953) | 292 (156,521) | 695 (292,1182) | 680 (448,973) | 360 (263,483) | 445 (360,543) |
| 36-47 | 237 (119,393) | 156 (37,334) | 260 (192,343) | 321 (206,482) | 148 (69,290) | 676 (298,1167) | 348 (168,565) | 205 (132,296) | 219 (164,280) |
| 48-59 | 36 (0,90) | 36 (0,133) | 65 (36,98) | 77 (42,133) | 22 (0,65) | 205 (0,553) | 56 (0,139) | 64 (31,108) | 54 (31,83) |
| 24-59 | 246 (160,354) | 226 (125,356) | 287 (233,359) | 363 (231,518) | 152 (88,253) | 508 (277,771) | 356 (246,487) | 209 (157,272) | 240 (200,288) |
| <60 | 894 (678,1156) | 1906 (1535,2347) | 1626 (1368,1923) | 2033 (1336,2805) | 566 (338,899) | 1436 (1086,1875) | 1297 (1080,1578) | 795 (638,986) | 1360 (1170,1552) |
| 2012 |  |  |  |  |  |  |  |  |  |
| <1 | 5452 (3479,7838) | 12128 (9416,15459) | 11921 (9889,14303) | 14607 (10060,20534) | 3477 (1835,5886) | 4255 (0,11441) | 7850 (5322,10955) | 5127 (3742,6953) | 9906 (8507,11584) |
| 1 | 6160 (4066,9156) | 13843 (10617,18035) | 13698 (11400,16318) | 16850 (11191,23556) | 3961 (2137,6672) | 4777 (0,11815) | 9093 (6168,12690) | 5915 (4404,7982) | 11353 (9742,13347) |
| 2 | 4465 (2966,6316) | 9928 (7777,13244) | 9873 (8202,11749) | 12017 (7840,16977) | 2817 (1583,4875) | 3412 (0,9123) | 6515 (4385,9076) | 4237 (3042,5695) | 8205 (7002,9581) |
| 3 | 2011 (1383,2755) | 5957 (4396,7875) | 4479 (3639,5443) | 5582 (3699,7782) | 1293 (745,2153) | 3316 (1389,5706) | 2913 (2089,3873) | 1859 (1384,2465) | 3726 (3182,4385) |
| 4 | 2679 (1899,3733) | 8042 (5897,10535) | 6021 (4993,7352) | 7501 (4952,10309) | 1727 (1022,2781) | 4490 (1939,7625) | 3933 (2829,5242) | 2496 (1832,3247) | 4997 (4227,5923) |
| 5 | 3759 (2619,5311) | 11244 (8136,14742) | 8454 (6973,10162) | 10577 (7167,15228) | 2448 (1366,3947) | 6442 (2749,11375) | 5513 (4045,7279) | 3481 (2672,4615) | 7048 (6025,8304) |
| 6 | 2972 (2022,4191) | 7591 (5207,10612) | 5583 (4522,6796) | 6933 (4498,9623) | 1850 (1094,3166) | 7006 (3485,11251) | 4324 (3110,5737) | 3126 (2332,4082) | 4671 (3939,5504) |
| 7 | 1688 (1105,2384) | 4301 (2925,6074) | 3146 (2570,3833) | 3909 (2631,5415) | 1061 (633,1729) | 3914 (2093,6356) | 2441 (1740,3247) | 1789 (1313,2368) | 2619 (2212,3167) |
| 8 | 2104 (1431,2968) | 5408 (3643,7684) | 3962 (3206,4801) | 4878 (3374,6905) | 1305 (758,2173) | 4916 (2576,7806) | 3063 (2126,4051) | 2235 (1643,2960) | 3269 (2751,3897) |
| 9 | 1320 (816,1930) | 1618 (717,2787) | 2109 (1725,2606) | 2616 (1766,3678) | 828 (460,1440) | 1906 (626,3609) | 1929 (1317,2702) | 1208 (831,1654) | 1750 (1438,2137) |
| 10 | 1297 (837,1891) | 1594 (730,2686) | 2084 (1653,2540) | 2583 (1750,3673) | 801 (441,1367) | 1870 (583,3722) | 1870 (1233,2691) | 1169 (834,1608) | 1732 (1410,2098) |
| 11 | 1213 (799,1903) | 1482 (642,2514) | 1978 (1569,2444) | 2480 (1537,3423) | 768 (425,1337) | 1819 (604,3393) | 1791 (1176,2508) | 1127 (807,1528) | 1640 (1342,1994) |
| <12 | 2529 (1892,3275) | 6994 (5504,8784) | 5566 (4683,6661) | 6903 (4590,9706) | 1611 (990,2647) | 4155 (2778,5881) | 3653 (2970,4478) | 2415 (1917,3081) | 4598 (4001,5271) |
| 12-14 | 804 (492,1219) | 1981 (1084,3038) | 1591 (1258,2011) | 1989 (1307,2794) | 522 (268,910) | 3670 (1763,5993) | 1192 (718,1704) | 1055 (750,1485) | 1314 (1076,1598) |
| 15-17 | 800 (330,1428) | 1779 (895,2967) | 1391 (1070,1814) | 1718 (1123,2575) | 499 (201,1064) | 2802 (1097,5051) | 1156 (481,1995) | 960 (636,1373) | 1159 (894,1494) |
| 18-20 | 1191 (637,1938) | 1253 (518,2205) | 1447 (1123,1856) | 1791 (1151,2622) | 749 (358,1379) | 1933 (614,3837) | 1747 (981,2739) | 917 (610,1285) | 1208 (902,1515) |
| 21-23 | 789 (438,1272) | 465 (0,1044) | 585 (424,799) | 724 (433,1091) | 487 (236,963) | 492 (0,1312) | 1154 (644,1849) | 348 (215,544) | 491 (351,652) |
| 12-23 | 880 (606,1222) | 1411 (979,1943) | 1262 (1039,1528) | 1557 (1004,2189) | 550 (320,903) | 2220 (1405,3081) | 1249 (932,1622) | 821 (629,1048) | 1039 (879,1245) |
| <24 | 1662 (1254,2128) | 4073 (3262,5019) | 3295 (2744,3923) | 4095 (2758,5670) | 1049 (605,1724) | 3208 (2360,4269) | 2415 (2008,2913) | 1569 (1255,1931) | 2750 (2384,3209) |
| 24-35 | 425 (259,648) | 600 (324,973) | 480 (379,601) | 592 (377,862) | 264 (145,460) | 880 (492,1432) | 620 (409,905) | 323 (243,438) | 400 (323,493) |
| 36-47 | 186 (93,314) | 187 (41,377) | 204 (148,270) | 253 (158,384) | 118 (53,232) | 524 (213,962) | 267 (136,439) | 159 (101,230) | 169 (125,219) |
| 48-59 | 27 (0,64) | 39 (0,137) | 47 (26,71) | 58 (32,100) | 17 (0,47) | 105 (0,374) | 41 (0,94) | 47 (22,80) | 40 (24,60) |
| 24-59 | 195 (131,276) | 270 (160,417) | 233 (187,288) | 292 (195,407) | 123 (71,208) | 527 (307,775) | 287 (203,387) | 169 (130,223) | 194 (163,229) |
| <60 | 779 (604,1014) | 1813 (1463,2248) | 1433 (1200,1716) | 1779 (1208,2457) | 496 (302,761) | 1600 (1210,2078) | 1140 (932,1364) | 701 (560,864) | 1190 (1034,1384) |
| 2013 |  |  |  |  |  |  |  |  |  |
| <1 | 2389 (1550,3401) | 3790 (2760,5194) | 5302 (4412,6397) | 6582 (4359,9184) | 1523 (851,2725) | 5688 (2500,9468) | 3534 (2375,4860) | 2285 (1676,3112) | 4374 (3722,5107) |
| 1 | 3705 (2384,5442) | 5814 (4011,7679) | 8023 (6665,9613) | 9872 (6534,13828) | 2301 (1230,3921) | 8465 (3734,14256) | 5285 (3550,7368) | 3420 (2567,4598) | 6635 (5717,7864) |
| 2 | 3044 (1950,4361) | 4844 (3456,6614) | 6783 (5586,8177) | 8495 (5793,11853) | 1977 (1070,3252) | 7244 (3101,11641) | 4490 (3041,6212) | 2947 (2121,3919) | 5634 (4850,6545) |
| 3 | 1531 (1041,2112) | 2123 (1159,3419) | 3414 (2780,4128) | 4246 (2881,5893) | 970 (587,1579) | 1926 (620,3701) | 2213 (1590,2935) | 1421 (1061,1894) | 2843 (2428,3368) |
| 4 | 1778 (1244,2497) | 2553 (1388,3997) | 3992 (3266,4825) | 4948 (3380,7001) | 1135 (650,1822) | 2234 (767,4241) | 2593 (1903,3439) | 1653 (1256,2129) | 3333 (2808,3904) |
| 5 | 2102 (1470,2962) | 2958 (1615,4628) | 4783 (3942,5742) | 5869 (3956,8282) | 1332 (792,2124) | 2676 (901,5513) | 3078 (2232,4065) | 1959 (1462,2611) | 3935 (3319,4648) |
| 6 | 1525 (1036,2107) | 1177 (248,2279) | 2886 (2376,3493) | 3595 (2366,5072) | 964 (554,1536) | 3024 (1237,5273) | 2198 (1596,2952) | 1617 (1207,2108) | 2403 (2000,2821) |
| 7 | 1149 (787,1658) | 851 (179,1795) | 2182 (1762,2693) | 2678 (1748,3799) | 716 (430,1204) | 2353 (862,4153) | 1680 (1207,2213) | 1230 (906,1602) | 1808 (1530,2150) |
| 8 | 989 (690,1419) | 754 (179,1438) | 1894 (1516,2277) | 2343 (1518,3224) | 634 (372,1050) | 2044 (838,3563) | 1461 (1056,1965) | 1065 (790,1370) | 1561 (1311,1883) |
| 9 | 405 (264,600) | 204 (0,556) | 659 (532,818) | 818 (552,1162) | 257 (137,446) | 484 (120,1090) | 594 (390,843) | 374 (261,515) | 550 (458,666) |
| 10 | 894 (548,1246) | 458 (0,1294) | 1416 (1128,1760) | 1749 (1156,2519) | 559 (307,949) | 1072 (252,2360) | 1285 (835,1771) | 805 (575,1109) | 1177 (976,1448) |
| 11 | 647 (417,966) | 326 (0,882) | 1058 (857,1310) | 1302 (867,1854) | 406 (218,691) | 785 (192,1653) | 953 (633,1348) | 597 (427,811) | 870 (714,1055) |
| <12 | 1454 (1106,1916) | 2361 (1746,3093) | 3239 (2682,3862) | 3981 (2620,5657) | 931 (569,1441) | 2484 (1598,3632) | 2126 (1745,2593) | 1410 (1126,1730) | 2682 (2323,3088) |
| 12-14 | 696 (415,1076) | 784 (156,1575) | 1365 (1071,1708) | 1689 (1096,2479) | 436 (225,771) | 2157 (475,4162) | 1029 (626,1451) | 911 (637,1280) | 1137 (922,1408) |
| 15-17 | 333 (141,610) | 319 (0,905) | 598 (455,773) | 737 (462,1064) | 212 (76,423) | 651 (0,1526) | 494 (216,819) | 413 (270,573) | 492 (388,630) |
| 18-20 | 602 (317,966) | 703 (160,1442) | 725 (549,927) | 891 (574,1359) | 371 (180,696) | 209 (0,716) | 870 (436,1328) | 465 (311,658) | 599 (467,770) |
| 21-23 | 782 (415,1255) | 159 (0,524) | 588 (424,788) | 730 (468,1127) | 495 (235,967) | 733 (0,1660) | 1136 (611,1867) | 347 (207,542) | 488 (344,650) |
| 12-23 | 575 (401,786) | 495 (238,818) | 831 (685,1000) | 1035 (679,1469) | 357 (201,590) | 813 (403,1346) | 841 (623,1082) | 541 (415,704) | 686 (581,813) |
| <24 | 1030 (774,1339) | 1441 (1078,1938) | 2040 (1694,2429) | 2534 (1750,3488) | 647 (398,1019) | 1639 (1101,2320) | 1488 (1223,1805) | 971 (784,1198) | 1695 (1463,1975) |
| 24-35 | 256 (161,379) | 74 (0,204) | 291 (231,371) | 354 (232,512) | 161 (87,289) | 768 (408,1139) | 376 (244,529) | 197 (143,265) | 241 (196,297) |
| 36-47 | 113 (56,194) | . (.,.) | 125 (92,167) | 155 (99,230) | 74 (33,148) | 171 (0,403) | 171 (86,274) | 100 (65,148) | 105 (80,137) |
| 48-59 | 16 (0,39) | 0 (0,92) | 28 (15,44) | 34 (16,58) | 10 (0,26) | 64 (0,169) | 24 (0,57) | 27 (13,47) | 23 (13,35) |
| 24-59 | 121 (84,176) | 26 (0,72) | 144 (117,177) | 180 (115,256) | 78 (43,129) | 331 (206,498) | 176 (125,237) | 104 (79,138) | 119 (98,144) |
| <60 | 480 (366,614) | 587 (445,764) | 871 (728,1029) | 1069 (715,1467) | 304 (183,479) | 855 (619,1136) | 691 (567,832) | 425 (343,531) | 723 (621,838) |
| 2014 |  |  |  |  |  |  |  |  |  |
| <1 | 3036 (1952,4397) | 5894 (4464,7647) | 6666 (5590,8131) | 8243 (5586,11293) | 1959 (1078,3234) | 5297 (0,12922) | 4490 (2996,6147) | 2881 (2115,3905) | 5559 (4741,6455) |
| 1 | 5918 (3836,8369) | 11335 (8441,14870) | 13000 (10753,15483) | 15962 (10595,22252) | 3693 (2110,6351) | 10247 (0,24812) | 8696 (5888,12099) | 5614 (4095,7525) | 10769 (9156,12437) |
| 2 | 3928 (2530,5654) | 7523 (5526,9908) | 8575 (7122,10270) | 10751 (7125,15022) | 2467 (1351,4253) | 6673 (0,15734) | 5707 (3872,7874) | 3707 (2633,5002) | 7127 (6163,8302) |
| 3 | 2286 (1617,3222) | 6700 (4791,9089) | 5092 (4185,6198) | 6259 (4149,8744) | 1416 (809,2357) | 3329 (1082,6082) | 3275 (2373,4385) | 2104 (1588,2751) | 4240 (3588,5014) |
| 4 | 2396 (1660,3442) | 7205 (5149,9544) | 5471 (4514,6600) | 6694 (4557,9394) | 1546 (857,2664) | 3518 (1429,6673) | 3508 (2597,4711) | 2221 (1656,2930) | 4507 (3847,5243) |
| 5 | 2206 (1563,3113) | 6551 (4817,8788) | 4994 (4147,5996) | 6194 (4275,8683) | 1403 (828,2316) | 3279 (964,5889) | 3245 (2402,4256) | 2046 (1510,2719) | 4146 (3540,4825) |
| 6 | 2105 (1423,2976) | 3957 (2393,5775) | 3951 (3243,4773) | 4883 (3387,7099) | 1324 (771,2277) | 3568 (1178,7037) | 3029 (2169,4112) | 2229 (1678,2943) | 3279 (2785,3904) |
| 7 | 2236 (1512,3078) | 4135 (2604,6137) | 4204 (3405,5047) | 5188 (3468,7348) | 1377 (822,2258) | 3759 (1253,7078) | 3224 (2312,4291) | 2333 (1715,3142) | 3482 (2917,4167) |
| 8 | 1716 (1159,2434) | 3146 (1839,4692) | 3238 (2685,3903) | 3989 (2753,5656) | 1071 (612,1849) | 2958 (999,5623) | 2492 (1747,3347) | 1804 (1302,2418) | 2702 (2232,3182) |
| 9 | 1109 (719,1692) | 1346 (483,2393) | 1809 (1452,2256) | 2270 (1477,3210) | 724 (395,1227) | 1774 (0,4466) | 1651 (1086,2298) | 1019 (725,1378) | 1508 (1248,1843) |
| 10 | 1273 (805,1875) | 1532 (557,2742) | 2028 (1623,2510) | 2501 (1649,3617) | 801 (450,1380) | 2106 (0,4905) | 1834 (1203,2590) | 1148 (818,1556) | 1690 (1401,2029) |
| 11 | 879 (573,1343) | 1077 (446,1912) | 1430 (1139,1786) | 1767 (1164,2492) | 552 (301,978) | 1471 (0,3325) | 1291 (886,1810) | 807 (583,1108) | 1195 (981,1444) |
| <12 | 2116 (1575,2787) | 5193 (4087,6492) | 4654 (3911,5588) | 5819 (3947,7992) | 1345 (785,2133) | 3313 (1953,5070) | 3097 (2512,3770) | 2043 (1632,2574) | 3884 (3360,4447) |
| 12-14 | 645 (383,976) | 881 (286,1737) | 1272 (987,1583) | 1550 (995,2302) | 404 (220,733) | 1182 (0,2652) | 936 (568,1386) | 842 (595,1154) | 1053 (858,1295) |
| 15-17 | 520 (221,937) | 162 (0,569) | 945 (714,1224) | 1164 (752,1674) | 330 (122,686) | 828 (0,2105) | 773 (327,1298) | 652 (428,926) | 787 (603,992) |
| 18-20 | 931 (508,1523) | 359 (0,928) | 1147 (858,1491) | 1406 (925,2122) | 577 (283,1068) | 2042 (436,3919) | 1396 (763,2130) | 729 (485,1047) | 948 (730,1188) |
| 21-23 | 1049 (553,1660) | 330 (0,898) | 787 (553,1069) | 969 (580,1475) | 665 (305,1247) | 718 (0,2740) | 1550 (747,2400) | 470 (262,720) | 654 (484,864) |
| 12-23 | 720 (510,988) | 450 (205,795) | 1049 (862,1290) | 1329 (876,1814) | 463 (269,762) | 1108 (499,1814) | 1064 (787,1392) | 683 (519,867) | 880 (735,1034) |
| <24 | 1427 (1105,1880) | 2948 (2318,3648) | 2857 (2405,3380) | 3581 (2495,4928) | 918 (540,1484) | 2153 (1400,3108) | 2102 (1707,2524) | 1358 (1098,1698) | 2371 (2054,2727) |
| 24-35 | 442 (270,671) | 283 (85,537) | 508 (398,637) | 633 (419,911) | 280 (150,496) | 263 (0,605) | 654 (432,931) | 341 (246,460) | 422 (337,517) |
| 36-47 | 188 (87,325) | 114 (0,264) | 205 (152,266) | 251 (160,373) | 116 (52,225) | 273 (0,650) | 279 (134,454) | 157 (99,232) | 168 (126,220) |
| 48-59 | 26 (0,64) | 42 (0,143) | 45 (25,69) | 55 (29,91) | 16 (0,43) | 103 (0,255) | 38 (0,94) | 45 (22,74) | 38 (21,58) |
| 24-59 | 203 (142,294) | 136 (58,238) | 242 (194,300) | 297 (200,424) | 130 (78,221) | 100 (25,235) | 298 (210,413) | 177 (135,229) | 201 (166,241) |
| <60 | 711 (543,940) | 1271 (1007,1647) | 1300 (1093,1545) | 1608 (1068,2225) | 454 (275,717) | 797 (523,1122) | 1022 (849,1226) | 634 (506,776) | 1071 (930,1243) |
| 2015 |  |  |  |  |  |  |  |  |  |
| <1 | 2170 (1389,3150) | 3955 (2920,5143) | 4700 (3890,5654) | 5783 (3805,7980) | 1332 (775,2316) | 1685 (0,4378) | 3120 (2129,4348) | 2023 (1473,2713) | 3890 (3361,4547) |
| 1 | 5830 (3762,8589) | 10644 (7813,13897) | 12927 (10682,15451) | 15996 (10714,22157) | 3654 (2092,6310) | 4693 (0,12934) | 8512 (5844,11938) | 5541 (3986,7450) | 10660 (9129,12510) |
| 2 | 4709 (3106,6836) | 8514 (6471,11079) | 10262 (8531,12273) | 12658 (8178,17553) | 2958 (1658,5063) | 3593 (0,9439) | 6852 (4627,9141) | 4386 (3163,6005) | 8459 (7180,9828) |
| 3 | 2408 (1667,3446) | 5685 (3817,7756) | 5416 (4441,6477) | 6709 (4416,9229) | 1549 (875,2561) | 3160 (0,7484) | 3510 (2635,4670) | 2243 (1634,2879) | 4501 (3793,5308) |
| 4 | 2235 (1535,3123) | 5254 (3593,7317) | 5031 (4086,6003) | 6228 (4277,8694) | 1410 (799,2314) | 2890 (0,7487) | 3256 (2386,4434) | 2051 (1551,2748) | 4177 (3591,4960) |
| 5 | 2546 (1752,3528) | 5879 (4076,8237) | 5677 (4707,7053) | 7065 (4811,9894) | 1626 (921,2683) | 3226 (0,7940) | 3693 (2709,4746) | 2336 (1718,3058) | 4721 (4010,5554) |
| 6 | 1870 (1347,2666) | 3017 (1651,4834) | 3537 (2894,4289) | 4332 (2794,6043) | 1177 (666,1990) | 4027 (1334,7896) | 2699 (1903,3620) | 1971 (1462,2617) | 2933 (2460,3535) |
| 7 | 1421 (983,1984) | 2345 (1267,3708) | 2707 (2233,3277) | 3351 (2260,4649) | 899 (517,1513) | 3110 (976,5896) | 2063 (1520,2801) | 1509 (1136,2005) | 2258 (1897,2729) |
| 8 | 1285 (896,1852) | 2083 (1114,3300) | 2458 (2009,2990) | 3017 (2047,4191) | 828 (450,1367) | 2767 (972,5145) | 1904 (1355,2515) | 1383 (1008,1814) | 2049 (1719,2444) |
| 9 | 1323 (856,1932) | 1635 (754,2780) | 2132 (1708,2686) | 2665 (1732,3733) | 834 (445,1428) | 3226 (1059,5725) | 1923 (1291,2694) | 1200 (869,1680) | 1769 (1438,2140) |
| 10 | 1411 (869,2116) | 1751 (780,3009) | 2287 (1824,2807) | 2805 (1906,3946) | 884 (487,1531) | 3377 (1118,6484) | 2047 (1356,2900) | 1303 (927,1767) | 1886 (1564,2277) |
| 11 | 1016 (645,1491) | 1248 (532,2146) | 1625 (1314,2021) | 2038 (1349,2859) | 639 (353,1101) | 2419 (789,4599) | 1474 (980,2058) | 920 (666,1251) | 1354 (1102,1630) |
| <12 | 1988 (1527,2597) | 4255 (3299,5498) | 4366 (3713,5089) | 5443 (3516,7475) | 1248 (761,2023) | 3425 (1378,6151) | 2906 (2396,3561) | 1907 (1508,2403) | 3643 (3118,4203) |
| 12-14 | 818 (473,1288) | 683 (154,1459) | 1595 (1257,2020) | 1958 (1339,2844) | 517 (269,909) | 883 (0,2888) | 1188 (713,1749) | 1062 (748,1457) | 1327 (1065,1627) |
| 15-17 | 594 (265,1055) | 442 (0,1071) | 1070 (831,1392) | 1327 (823,1924) | 370 (153,760) | 1204 (0,3296) | 871 (361,1487) | 726 (475,1027) | 895 (694,1125) |
| 18-20 | 1038 (550,1727) | 852 (178,1702) | 1305 (990,1656) | 1596 (1067,2358) | 675 (329,1266) | 1517 (0,3957) | 1559 (879,2372) | 823 (537,1167) | 1075 (836,1374) |
| 21-23 | 777 (401,1271) | 319 (0,854) | 575 (411,782) | 703 (439,1067) | 483 (217,909) | 779 (0,2478) | 1100 (579,1793) | 339 (192,530) | 477 (350,638) |
| 12-23 | 785 (553,1090) | 589 (304,912) | 1145 (945,1404) | 1395 (903,1979) | 499 (293,800) | 452 (0,1242) | 1151 (869,1517) | 736 (574,964) | 948 (797,1122) |
| <24 | 1389 (1030,1831) | 2424 (1887,3100) | 2767 (2335,3293) | 3380 (2344,4759) | 879 (534,1386) | 1619 (682,2753) | 2012 (1643,2450) | 1312 (1050,1611) | 2293 (1991,2666) |
| 24-35 | 365 (230,536) | 248 (80,494) | 417 (326,518) | 512 (342,738) | 232 (121,395) | 561 (0,1310) | 529 (346,773) | 283 (202,382) | 349 (280,424) |
| 36-47 | 234 (116,398) | 82 (0,209) | 251 (185,329) | 313 (194,454) | 148 (63,283) | 212 (0,694) | 337 (155,538) | 199 (125,281) | 209 (158,271) |
| 48-59 | 28 (0,72) | 39 (0,134) | 50 (29,76) | 59 (31,103) | 18 (0,49) | 0 (0,477) | 43 (0,103) | 49 (25,84) | 42 (24,63) |
| 24-59 | 202 (131,288) | 109 (43,205) | 237 (190,294) | 295 (186,409) | 126 (68,206) | 130 (0,336) | 291 (205,395) | 172 (128,224) | 198 (162,237) |
| <60 | 690 (534,891) | 1052 (794,1353) | 1254 (1059,1488) | 1544 (1048,2158) | 440 (277,700) | 667 (323,1126) | 1003 (840,1203) | 612 (501,754) | 1048 (900,1222) |
| 2016 |  |  |  |  |  |  |  |  |  |
| <1 | 4209 (2792,6118) | 8030 (5966,10405) | 9222 (7695,11148) | 11289 (7593,16145) | 2648 (1446,4493) | 7438 (2491,13717) | 6128 (4110,8415) | 3981 (2855,5442) | 7648 (6506,8879) |
| 1 | 7262 (4690,10649) | 13904 (10313,17919) | 16049 (13349,19483) | 19775 (12841,27357) | 4616 (2589,8214) | 12754 (4632,23026) | 10509 (7322,15003) | 6966 (4848,9316) | 13224 (11299,15350) |
| 2 | 5093 (3304,7510) | 9620 (7082,12329) | 11145 (9379,13387) | 13799 (9061,19134) | 3256 (1886,5637) | 9073 (3047,16486) | 7327 (4859,10108) | 4797 (3383,6337) | 9217 (7968,10767) |
| 3 | 2711 (1855,3785) | 4556 (2745,6750) | 6024 (4961,7245) | 7502 (5152,10185) | 1714 (970,2793) | 7673 (2494,14199) | 3915 (2892,5203) | 2486 (1861,3325) | 4998 (4319,5878) |
| 4 | 1322 (916,1808) | 2233 (1363,3336) | 2971 (2469,3581) | 3707 (2437,5146) | 843 (481,1423) | 3821 (1258,6547) | 1939 (1428,2547) | 1225 (906,1654) | 2478 (2095,2890) |
| 5 | 2299 (1634,3272) | 3840 (2291,5682) | 5169 (4241,6239) | 6388 (4346,8923) | 1472 (812,2470) | 6564 (2303,11916) | 3347 (2467,4430) | 2143 (1585,2808) | 4302 (3677,4986) |
| 6 | 1680 (1149,2349) | 2555 (1443,4074) | 3176 (2618,3930) | 3936 (2656,5560) | 1064 (626,1726) | 6202 (2826,10317) | 2468 (1742,3325) | 1798 (1347,2343) | 2649 (2228,3180) |
| 7 | 1322 (895,1864) | 2044 (1032,3334) | 2513 (2054,3084) | 3130 (2049,4440) | 860 (480,1441) | 4832 (2161,8098) | 1938 (1349,2581) | 1411 (1051,1882) | 2096 (1738,2482) |
| 8 | 1978 (1348,2809) | 3071 (1629,4907) | 3712 (3039,4597) | 4637 (3027,6618) | 1235 (694,2043) | 7124 (3368,11640) | 2838 (2038,3868) | 2086 (1542,2775) | 3085 (2594,3661) |
| 9 | 1032 (680,1516) | 1411 (451,2669) | 1663 (1330,2104) | 2062 (1313,2944) | 655 (370,1116) | 4145 (1868,6936) | 1482 (989,2131) | 946 (661,1273) | 1383 (1122,1680) |
| 10 | 835 (524,1184) | 1168 (377,2215) | 1376 (1107,1686) | 1670 (1076,2400) | 544 (288,943) | 3339 (1538,5696) | 1236 (816,1771) | 769 (543,1075) | 1135 (920,1371) |
| 11 | 406 (259,598) | 548 (194,1109) | 659 (530,823) | 818 (537,1139) | 259 (138,456) | 1647 (749,2756) | 594 (392,844) | 373 (262,508) | 548 (449,669) |
| <12 | 2062 (1593,2689) | 4507 (3449,5802) | 4530 (3753,5367) | 5709 (3727,7848) | 1326 (790,2052) | 6392 (3509,9697) | 3013 (2440,3682) | 1980 (1577,2437) | 3761 (3256,4372) |
| 12-14 | 570 (342,873) | 469 (0,1068) | 1117 (879,1389) | 1378 (886,1951) | 367 (199,626) | 2465 (0,5810) | 839 (530,1225) | 746 (506,1021) | 928 (748,1159) |
| 15-17 | 688 (302,1219) | 177 (0,615) | 1203 (926,1549) | 1487 (964,2244) | 424 (169,838) | 2044 (0,5223) | 992 (431,1625) | 824 (553,1175) | 1010 (775,1293) |
| 18-20 | 644 (367,1022) | 500 (0,1138) | 779 (595,1003) | 967 (609,1408) | 415 (195,779) | 0 (0,3490) | 948 (526,1504) | 494 (321,714) | 647 (499,824) |
| 21-23 | 1064 (598,1798) | 154 (0,535) | 827 (580,1091) | 1013 (608,1555) | 684 (322,1282) | 838 (0,2650) | 1583 (833,2463) | 488 (266,777) | 678 (481,886) |
| 12-23 | 686 (482,966) | 332 (131,596) | 1004 (821,1199) | 1241 (835,1746) | 433 (259,735) | 1302 (273,2632) | 1007 (740,1323) | 651 (507,835) | 831 (708,987) |
| <24 | 1350 (1041,1757) | 2348 (1812,3022) | 2715 (2264,3210) | 3361 (2169,4764) | 856 (522,1389) | 3562 (2101,5317) | 1974 (1618,2363) | 1273 (1014,1583) | 2234 (1942,2583) |
| 24-35 | 349 (214,518) | 193 (42,402) | 399 (314,492) | 490 (321,681) | 221 (119,375) | 593 (0,1375) | 508 (341,713) | 266 (187,365) | 330 (266,399) |
| 36-47 | 170 (80,284) | 124 (0,292) | 191 (141,252) | 232 (145,347) | 110 (48,214) | 468 (0,1226) | 250 (129,430) | 149 (98,210) | 157 (117,205) |
| 48-59 | 29 (0,74) | 40 (0,143) | 49 (27,74) | 58 (31,101) | 17 (0,49) | 0 (0,436) | 41 (0,97) | 47 (23,82) | 40 (23,60) |
| 24-59 | 180 (123,254) | 112 (40,205) | 212 (172,262) | 264 (175,380) | 115 (64,193) | 408 (131,793) | 260 (189,351) | 154 (119,204) | 177 (145,211) |
| <60 | 642 (487,825) | 1004 (766,1287) | 1178 (989,1408) | 1445 (984,2013) | 414 (246,650) | 1560 (1024,2207) | 939 (778,1117) | 578 (460,708) | 978 (852,1135) |
| 2017 |  |  |  |  |  |  |  |  |  |
| <1 | 2978 (1958,4373) | 3624 (2370,5172) | 6481 (5360,7699) | 7986 (5229,10864) | 1906 (1024,3346) | 6906 (0,16385) | 4284 (2922,5945) | 2832 (2025,3859) | 5389 (4657,6305) |
| 1 | 3701 (2400,5295) | 4566 (2975,6644) | 8153 (6707,9702) | 10074 (6678,13982) | 2360 (1260,4092) | 8600 (0,19137) | 5372 (3701,7345) | 3473 (2539,4677) | 6763 (5815,7958) |
| 2 | 2119 (1363,3146) | 2611 (1691,3816) | 4660 (3883,5623) | 5734 (3960,8104) | 1353 (735,2339) | 4898 (0,11190) | 3102 (2153,4217) | 2012 (1466,2691) | 3859 (3335,4520) |
| 3 | 1613 (1091,2209) | 2712 (1390,4367) | 3591 (2987,4310) | 4486 (2963,6184) | 1007 (591,1695) | 5929 (0,13872) | 2316 (1716,3095) | 1479 (1109,1962) | 2969 (2530,3506) |
| 4 | 1219 (865,1687) | 2088 (1071,3325) | 2764 (2291,3357) | 3453 (2254,4889) | 780 (469,1257) | 4658 (0,10554) | 1798 (1291,2327) | 1141 (867,1500) | 2301 (1973,2701) |
| 5 | 888 (610,1239) | 1518 (771,2414) | 2002 (1662,2425) | 2469 (1696,3441) | 575 (325,944) | 3286 (0,7612) | 1306 (950,1699) | 821 (612,1084) | 1666 (1409,1942) |
| 6 | 668 (465,964) | 958 (336,1855) | 1263 (1020,1537) | 1574 (1049,2180) | 418 (238,710) | 660 (0,2206) | 984 (700,1330) | 706 (530,928) | 1043 (887,1256) |
| 7 | 771 (513,1070) | 1135 (358,2139) | 1461 (1178,1792) | 1794 (1183,2562) | 489 (274,818) | 787 (0,2521) | 1122 (797,1502) | 817 (602,1073) | 1213 (1023,1447) |
| 8 | 692 (468,975) | 983 (335,1822) | 1281 (1048,1592) | 1580 (1048,2201) | 431 (257,697) | 701 (0,2315) | 990 (693,1320) | 722 (544,943) | 1072 (904,1263) |
| 9 | 675 (432,1012) | 944 (213,1893) | 1082 (867,1331) | 1347 (907,1952) | 416 (225,697) | 747 (0,2456) | 971 (656,1369) | 598 (432,827) | 896 (738,1080) |
| 10 | 592 (369,877) | 798 (272,1637) | 962 (767,1182) | 1186 (768,1670) | 369 (200,626) | 684 (0,2175) | 869 (582,1228) | 547 (378,742) | 800 (653,971) |
| 11 | 587 (364,874) | 813 (185,1620) | 947 (760,1181) | 1170 (773,1668) | 371 (202,647) | 665 (0,2249) | 849 (575,1185) | 538 (382,727) | 789 (640,942) |
| <12 | 1173 (890,1560) | 1989 (1433,2693) | 2580 (2133,3090) | 3157 (2103,4449) | 735 (464,1171) | 2069 (0,4503) | 1698 (1368,2074) | 1114 (901,1393) | 2141 (1820,2467) |
| 12-14 | 358 (214,546) | 330 (0,912) | 688 (544,878) | 857 (572,1214) | 221 (114,408) | 879 (0,2980) | 513 (310,743) | 464 (314,630) | 568 (455,702) |
| 15-17 | 256 (107,469) | 0 (0,353) | 464 (345,600) | 568 (360,842) | 162 (62,327) | 0 (0,2381) | 380 (169,656) | 315 (204,450) | 386 (291,489) |
| 18-20 | 332 (187,533) | 163 (0,574) | 408 (309,525) | 510 (319,732) | 212 (98,392) | 0 (0,1848) | 485 (272,757) | 261 (169,378) | 342 (268,430) |
| 21-23 | 371 (192,604) | 163 (0,534) | 273 (197,369) | 342 (206,504) | 230 (101,449) | 0 (0,1686) | 539 (292,858) | 164 (90,251) | 228 (164,302) |
| 12-23 | 317 (227,429) | 166 (38,365) | 458 (378,562) | 568 (382,804) | 200 (115,331) | 507 (0,1412) | 460 (343,591) | 299 (230,387) | 382 (325,451) |
| <24 | 723 (550,942) | 1077 (781,1460) | 1446 (1205,1736) | 1798 (1187,2521) | 471 (289,701) | 1297 (442,2425) | 1060 (880,1280) | 688 (549,851) | 1202 (1050,1395) |
| 24-35 | 185 (116,269) | 81 (0,221) | 212 (168,266) | 263 (177,381) | 118 (66,204) | 332 (0,1016) | 270 (179,388) | 145 (105,193) | 175 (143,215) |
| 36-47 | 89 (44,149) | 39 (0,138) | 97 (71,131) | 121 (76,179) | 56 (25,108) | 0 (0,636) | 130 (68,203) | 76 (49,112) | 82 (61,105) |
| 48-59 | 13 (0,32) | 42 (0,143) | 23 (12,36) | 27 (14,45) | 8 (0,21) | 0 (0,256) | 20 (0,47) | 23 (11,38) | 19 (10,30) |
| 24-59 | 93 (65,130) | 54 (10,118) | 110 (87,137) | 135 (91,186) | 57 (33,99) | 165 (0,441) | 135 (92,183) | 80 (60,101) | 91 (76,108) |
| <60 | 347 (261,444) | 468 (334,627) | 631 (529,742) | 780 (516,1093) | 219 (136,351) | 585 (230,1081) | 500 (414,600) | 309 (250,379) | 517 (452,597) |
| 2018 |  |  |  |  |  |  |  |  |  |
| <1 | 7404 (4707,10757) | 11997 (8670,16030) | 16423 (13654,19760) | 20346 (13254,28352) | 4731 (2604,7985) | 20929 (0,45247) | 10977 (7363,15372) | 7078 (5097,9778) | 13693 (11806,16099) |
| 1 | 6128 (3928,8980) | 9820 (6876,13043) | 13356 (11266,16171) | 16528 (11041,22794) | 3956 (2154,6722) | 16921 (0,37648) | 8905 (6015,12252) | 5856 (4140,7798) | 11247 (9591,12965) |
| 2 | 3723 (2334,5320) | 5913 (4313,7927) | 8161 (6717,9935) | 10115 (6619,13889) | 2331 (1301,4091) | 10399 (0,22048) | 5421 (3613,7775) | 3529 (2495,4714) | 6769 (5791,7825) |
| 3 | 2554 (1770,3667) | 4403 (2838,6463) | 5719 (4672,6883) | 7015 (4699,9707) | 1627 (969,2655) | 9051 (2047,16369) | 3745 (2675,4963) | 2341 (1746,3076) | 4724 (4020,5545) |
| 4 | 2873 (1977,4056) | 5063 (3156,7395) | 6519 (5339,7824) | 7962 (5309,11013) | 1788 (1032,3013) | 9910 (2342,18154) | 4284 (3133,5608) | 2698 (1993,3603) | 5389 (4590,6333) |
| 5 | 2251 (1543,3140) | 3920 (2504,5704) | 5091 (4209,6147) | 6215 (4272,8886) | 1425 (816,2294) | 7461 (1722,14499) | 3285 (2385,4394) | 2084 (1553,2804) | 4219 (3646,4925) |
| 6 | 1774 (1212,2646) | 2157 (1124,3631) | 3392 (2771,4136) | 4268 (2808,5857) | 1121 (656,1851) | 6906 (2291,12745) | 2611 (1840,3544) | 1906 (1401,2524) | 2845 (2344,3379) |
| 7 | 1463 (966,2050) | 1721 (864,2791) | 2738 (2185,3340) | 3418 (2292,4763) | 929 (542,1523) | 5591 (1883,10062) | 2126 (1493,2811) | 1532 (1149,2035) | 2279 (1925,2741) |
| 8 | 2061 (1402,2823) | 2491 (1200,4006) | 3916 (3186,4791) | 4821 (3350,6747) | 1311 (713,2136) | 8110 (2791,14978) | 2997 (2125,4006) | 2197 (1679,2936) | 3239 (2723,3918) |
| 9 | 1162 (743,1755) | 1699 (763,2861) | 1887 (1487,2344) | 2332 (1581,3326) | 737 (403,1238) | 2828 (0,6343) | 1714 (1103,2404) | 1062 (765,1441) | 1559 (1274,1918) |
| 10 | 1292 (815,1913) | 1844 (871,3154) | 2073 (1655,2598) | 2573 (1711,3664) | 809 (461,1376) | 3143 (0,6924) | 1891 (1217,2662) | 1188 (852,1635) | 1726 (1406,2088) |
| 11 | 956 (615,1396) | 1429 (624,2401) | 1544 (1242,1944) | 1927 (1257,2784) | 608 (337,1130) | 2354 (0,5142) | 1425 (977,1991) | 872 (623,1213) | 1283 (1056,1559) |
| <12 | 2283 (1712,3001) | 4236 (3170,5481) | 5046 (4226,6044) | 6259 (4237,8516) | 1429 (862,2348) | 7084 (4137,10778) | 3344 (2666,4127) | 2203 (1766,2762) | 4218 (3621,4881) |
| 12-14 | 579 (330,898) | 561 (127,1195) | 1120 (887,1406) | 1397 (888,2013) | 365 (178,624) | 789 (0,2575) | 843 (511,1243) | 757 (513,1058) | 935 (745,1157) |
| 15-17 | 466 (191,842) | 316 (0,861) | 856 (645,1119) | 1056 (688,1556) | 298 (113,574) | 1230 (0,2897) | 710 (331,1209) | 585 (390,830) | 703 (548,906) |
| 18-20 | 946 (499,1540) | 477 (0,1148) | 1185 (905,1503) | 1436 (952,2128) | 619 (287,1085) | 967 (0,3747) | 1421 (771,2186) | 749 (496,1050) | 975 (760,1210) |
| 21-23 | 874 (462,1455) | 177 (0,613) | 666 (473,903) | 814 (478,1267) | 574 (259,1040) | 901 (0,2968) | 1293 (687,2026) | 390 (216,615) | 552 (400,738) |
| 12-23 | 665 (465,925) | 284 (88,536) | 963 (795,1165) | 1201 (810,1676) | 421 (244,687) | 924 (192,1927) | 968 (716,1264) | 627 (487,823) | 803 (674,956) |
| <24 | 1443 (1105,1873) | 2152 (1627,2771) | 2869 (2410,3453) | 3525 (2440,4871) | 911 (552,1399) | 3383 (2018,4988) | 2101 (1704,2576) | 1353 (1071,1669) | 2379 (2057,2786) |
| 24-35 | 448 (271,683) | 196 (42,400) | 517 (409,640) | 643 (422,900) | 286 (150,490) | 817 (196,1694) | 668 (427,925) | 346 (251,471) | 425 (350,527) |
| 36-47 | 191 (94,330) | 81 (0,232) | 212 (156,278) | 259 (161,381) | 122 (55,223) | 610 (143,1274) | 280 (144,462) | 165 (107,240) | 174 (130,228) |
| 48-59 | 33 (0,78) | 88 (0,243) | 54 (31,86) | 67 (36,118) | 20 (0,54) | 86 (0,321) | 47 (0,112) | 55 (28,94) | 45 (25,70) |
| 24-59 | 213 (146,305) | 27 (0,75) | 256 (209,314) | 316 (201,446) | 136 (74,235) | 275 (92,542) | 316 (225,419) | 186 (138,242) | 211 (175,254) |
| <60 | 702 (540,912) | 869 (661,1119) | 1278 (1077,1515) | 1588 (1041,2181) | 438 (276,714) | 1292 (845,1860) | 1017 (850,1235) | 631 (505,779) | 1064 (923,1214) |

**Table S10: Annual average number of RSV associated acute respiratory illnesses (ARI), severe acute respiratory illness (SARI) and deaths among children aged <5 years in Kenya, by County**

| Region | County | ARI | | SARI | | Deaths | |
| --- | --- | --- | --- | --- | --- | --- | --- |
|  |  | Medically attended | Non medically attended | Hospitalized | Non-Hospitalized | In-Hospital | Out of Hospital |
|  |  | Number (95% CI) | Number (95% CI) | Number (95% CI) | Number (95% CI) | Number (95% CI) | Number (95% CI) |
| National | Kenya | 1234613 (1114748,1372458) | 1354478 (1222626,1510303) | 20917 (18160,24213) | 64547 (55977,74736) | 539 (420,779) | 1921 (1495,2774) |
| Coast | Mombasa | 44075 (37567,51027) | 50435 (43040,58422) | 575 (470,700) | 1784 (1461,2170) | 15 (10,21) | 53 (37,74) |
|  | Kwale | 38826 (33094,44950) | 44429 (37914,51465) | 507 (414,616) | 1571 (1287,1911) | 13 (9,18) | 46 (32,65) |
|  | Kilifi | 59848 (51011,69287) | 68483 (58442,79328) | 781 (639,950) | 2422 (1984,2946) | 20 (14,28) | 72 (50,100) |
|  | Tana River | 15847 (13507,18346) | 18134 (15475,21005) | 207 (169,252) | 641 (525,780) | 5 (4,7) | 19 (13,27) |
|  | Lamu | 5584 (4759,6465) | 6390 (5453,7402) | 73 (60,89) | 226 (185,275) | 2 (1,3) | 7 (5,9) |
|  | Taita/Taveta | 11529 (9827,13348) | 13193 (11258,15282) | 151 (123,183) | 467 (382,568) | 4 (3,5) | 14 (10,19) |
| North Eastern | Garissa | 19421 (12836,26341) | 52794 (34824,71769) | 295 (195,406) | 1807 (1198,2486) | 8 (4,11) | 28 (16,40) |
|  | Wajir | 21372 (14125,28987) | 58097 (38322,78978) | 324 (215,447) | 1989 (1318,2735) | 9 (5,12) | 31 (18,44) |
|  | Mandera | 28030 (18525,38017) | 76196 (50260,103582) | 425 (282,586) | 2608 (1729,3587) | 11 (6,16) | 40 (23,57) |
| Eastern | Marsabit | 20541 (18120,23238) | 15008 (13279,17013) | 277 (234,328) | 894 (755,1059) | 8 (5,10) | 27 (17,34) |
|  | Isiolo | 12063 (10642,13647) | 8814 (7798,9992) | 162 (137,193) | 525 (443,622) | 4 (3,6) | 16 (10,20) |
|  | Meru | 50568 (44609,57208) | 36947 (32690,41884) | 681 (575,807) | 2200 (1858,2607) | 19 (12,24) | 67 (42,85) |
|  | Tharaka-Nithi | 11994 (10581,13569) | 8763 (7754,9934) | 162 (136,191) | 522 (441,618) | 4 (3,6) | 16 (10,20) |
|  | Embu | 18279 (16125,20680) | 13356 (11817,15141) | 246 (208,292) | 795 (671,942) | 7 (4,9) | 24 (15,31) |
|  | Kitui | 37478 (33062,42400) | 27383 (24228,31043) | 505 (427,598) | 1630 (1377,1932) | 14 (9,18) | 49 (31,63) |
|  | Machakos | 43617 (38477,49344) | 31868 (28197,36127) | 587 (496,696) | 1897 (1602,2248) | 16 (10,21) | 58 (37,73) |
|  | Makueni | 28638 (25263,32399) | 20924 (18514,23721) | 386 (326,457) | 1246 (1052,1476) | 11 (7,13) | 38 (24,48) |
| Central | Nyandarua | 10218 (8189,12458) | 10568 (8469,12878) | 172 (132,227) | 498 (381,657) | 5 (3,7) | 17 (10,25) |
|  | Nyeri | 10672 (8552,13011) | 11038 (8845,13450) | 180 (137,237) | 520 (398,686) | 5 (3,7) | 18 (10,26) |
|  | Kirinyaga | 8394 (6726,10233) | 8681 (6956,10578) | 141 (108,186) | 409 (313,540) | 4 (2,6) | 14 (8,20) |
|  | Murang'a | 16135 (12930,19671) | 16688 (13372,20335) | 272 (208,358) | 787 (602,1038) | 8 (4,11) | 28 (16,39) |
|  | Kiambu | 40609 (32542,49509) | 41999 (33655,51178) | 684 (523,901) | 1980 (1516,2612) | 19 (11,28) | 69 (40,99) |
| Rift Valey | Turkana | 23394 (19714,27732) | 20240 (16954,23920) | 400 (327,480) | 1350 (1105,1622) | 11 (8,14) | 37 (28,51) |
|  | West Pokot | 20290 (17099,24052) | 17554 (14705,20746) | 347 (284,416) | 1171 (959,1407) | 9 (7,13) | 32 (24,45) |
|  | Samburu | 9021 (7602,10694) | 7805 (6538,9224) | 154 (126,185) | 520 (426,625) | 4 (3,6) | 14 (11,20) |
|  | Trans Nzoia | 23061 (19434,27337) | 19952 (16713,23580) | 394 (323,473) | 1331 (1090,1599) | 10 (8,14) | 37 (28,51) |
|  | Uasin Gishu | 24960 (21033,29587) | 21594 (18089,25521) | 426 (349,512) | 1440 (1179,1730) | 11 (8,15) | 40 (30,55) |
|  | Elgeyo/Marakwet | 10846 (9140,12857) | 9384 (7860,11090) | 185 (152,222) | 626 (512,752) | 5 (4,7) | 17 (13,24) |
|  | Nandi | 18863 (15896,22360) | 16319 (13670,19287) | 322 (264,387) | 1088 (891,1308) | 8 (6,12) | 30 (23,41) |
|  | Baringo | 16916 (14255,20052) | 14635 (12259,17296) | 289 (237,347) | 976 (799,1173) | 8 (6,10) | 27 (20,37) |
|  | Laikipia | 11666 (9831,13829) | 10093 (8455,11928) | 199 (163,239) | 673 (551,809) | 5 (4,7) | 19 (14,26) |
|  | Nakuru | 48653 (41000,57673) | 42093 (35260,49746) | 831 (681,998) | 2807 (2299,3373) | 22 (16,30) | 78 (58,107) |
|  | Narok | 33445 (28184,39646) | 28936 (24239,34197) | 571 (468,686) | 1930 (1580,2319) | 15 (11,21) | 54 (40,74) |
|  | Kajiado | 27826 (23449,32985) | 24075 (20166,28452) | 475 (389,571) | 1605 (1315,1929) | 13 (9,17) | 45 (33,61) |
|  | Kericho | 19501 (16434,23117) | 16872 (14133,19940) | 333 (273,400) | 1125 (921,1352) | 9 (7,12) | 31 (23,43) |
|  | Bomet | 19754 (16646,23416) | 17090 (14316,20198) | 337 (276,405) | 1140 (933,1369) | 9 (7,12) | 32 (24,43) |
| Western | Kakamega | 44958 (38180,53317) | 80879 (68453,95790) | 621 (506,779) | 1417 (1154,1783) | 16 (11,23) | 56 (40,80) |
|  | Vihiga | 12863 (10924,15255) | 23140 (19585,27407) | 178 (145,223) | 405 (330,510) | 5 (3,6) | 16 (12,23) |
|  | Bungoma | 43616 (37040,51725) | 78464 (66410,92930) | 603 (491,756) | 1374 (1120,1729) | 15 (11,22) | 55 (39,78) |
|  | Busia | 22547 (19148,26740) | 40563 (34331,48041) | 312 (254,391) | 710 (579,894) | 8 (6,11) | 28 (20,40) |
| Nyanza | Siaya | 23115 (19465,27130) | 22872 (19222,26886) | 451 (370,543) | 1501 (1232,1808) | 12 (9,16) | 43 (30,56) |
|  | Kisumu | 26803 (22571,31459) | 26521 (22289,31176) | 523 (429,629) | 1741 (1429,2096) | 14 (10,18) | 50 (35,65) |
|  | Homa Bay | 29582 (24911,34720) | 29270 (24600,34408) | 578 (473,694) | 1921 (1577,2314) | 16 (11,20) | 55 (39,72) |
|  | Migori | 30671 (25828,35998) | 30348 (25505,35675) | 599 (491,720) | 1992 (1635,2399) | 16 (11,21) | 58 (40,75) |
|  | Kisii | 27266 (22961,32002) | 26979 (22674,31715) | 532 (436,640) | 1771 (1454,2133) | 14 (10,19) | 51 (36,66) |
|  | Nyamira | 12246 (10313,14373) | 12117 (10184,14244) | 239 (196,287) | 795 (653,958) | 6 (5,8) | 23 (16,30) |
| Nairobi | Nairobi | 15225 (8400,23624) | 18900 (9975,29924) | 724 (441,1123) | 2394 (1470,3727) | 21 (10,31) | 75 (37,112) |
