# Supplementary material for: Estimates of the national burden of respiratory syncytial virus in Kenyan children aged under 5 years, 2010–2018
Source: BMC Med. 2023 Mar 31;21:122. doi: 10.1186/s12916-023-02787-w (PMC10067313; doi:10.1186/s12916-023-02787-w)
Supplement: Supplementary file 3 — Additional file 3. Methods for estimating RSV associated severe acute respiratory illness (SARI). [file 12916_2023_2787_MOESM3_ESM.docx]

# **Additional file 3 – Methodology: The burden of hospitalized and non-hospitalized RSV-associated severe acute respiratory illness among children aged <5 years**

We used data collected from children aged <5 years visiting Kilifi County Referral Hospital (CRH) and residency data from participants in the Kilifi Health and Demographic Surveillance System (HDSS). We followed the methods and equations used by Dawa et al [1], an update of the methods used by Fuller et al [2] in estimating the national burden of influenza associated severe acute respiratory illness (SARI), to estimate the national burden of hospitalized and non-hospitalized - respiratory syncytial virus (RSV) associated SARI in Kenya. The steps are outlined below;

1. Annual base rates of hospitalized severe acute respiratory illness (SARI) were calculated for the Kilifi Health and Demographic Surveillance System (HDSS) participants for the years 2010-2018 by dividing the age-specific number of hospitalized SARI by the age-specific population of HDSS residents in Kilifi. This is then adjusted to include all hospitalizations within the Kilifi HDSS (Approximately 80% of hospitalizations occur at Kilifi County Referral hospital (KCRH) ) by multiplying the rate by the reciprocal of proportion of those hospitalized at KCRH.

$$I_{B}=\frac{{SARI}_{B}}{{Pop}_{B}}*\frac{1}{W}$$

Equation 1

Where:

$I_{B}$ = Base rate of hospitalized SARI

${SARI}_{B}$ =Total number of SARI cases in base region per year

${Pop}_{B}$ = Population of surveillance catchment area

*W* =Proportion of SARI cases hospitalized at KCRH out of all hospitalizations in Kilifi HDSS

1. An adjustment factor for hospitalized SARI within each region (province) was calculated using equation 2

$${Adj}_{Y}=\left( 1+\sum_{i} \left( P_{i,Y}-P_{i,B} \right)\times\left( {RR}_{i}-1 \right) \right)\times\frac{{DHS}_{Y}}{{DHS}_{B}}$$

Equation 2

Where:

${Adj}_{Y}$ = Adjustment factor for region Y

$P_{i,Y}$ = Prevalence of risk factor *i* in region Y (from Kenya Demographic and Health Survey (KDHS))

$P_{i,B}$ = Prevalence of risk factor *i* in base region

${RR}_{i}$ = Relative risk of SARI due to risk factor *I*

${DHS}_{Y}$ = Proportion of ARI cases seeking care in region Y (from KDHS)

${DHS}_{B}$ = Proportion of ARI cases seeking care in base region (from KDHS)

1. The incidence of hospitalized SARI in each region

– Equation 3 = Equation 1 multiplied by Equation 2

$$I_{H,Y}=I_{B} \times{Adj}_{Y}$$

Equation 3

Where:

$I_{H,Y}$ = Incidence of hospitalized SARI in region Y

1. We estimated the rates of non-hospitalized (NH) SARI in the base region by applying an adjustment that accounts for health care seeking for acute respiratory illness in the Base region (Coast) using the 2018 Health Utilization Survey (HUS) data (Unpublished). HUS was conducted in four Counties in Kenya namely Siaya, Marsabit, Nakuru and Kakamega where the proportion of individuals hospitalized for pneumonia was used as a proxy for SARI hospitalization. For the rates of non-hospitalized (NH) SARI in Nyanza, Eastern, Rift Valley and Western we used the 2018 HUS estimates whereas for the other regions i.e. central, Nairobi and North Eastern we adjusted similar to the Coast region.

$$I_{NH,Y}=\left( I_{H,Y} \times\frac{1}{{HUS}_{Y}} \right)- I_{H,Y}$$

Equation 4

Where:

$I_{H,Y}$ = Incidence of hospitalized SARI in region Y

$I_{NH,Y}$ = Incidence of non-hospitalized SARI in region Y

${HUS}_{Y}$ = Proportion of all SARI cases that are hospitalized in region Y

$${HUS}_{Y}={HUS}_{2018}\times\frac{{DHS}_{Y}}{{DHS}_{2014}}$$

Equation 5

Where:

${HUS}_{2018}$ = Proportion of all hospitalized SARI cases in the four counties

${HUS}_{Y}$ = Proportion of all hospitalized SARI cases in region Y

${DHS}_{Y}$ = Proportion of ARI cases seeking care in region Y (from KDHS)

${DHS}_{2014}$ = Proportion of ARI cases seeking care (from KDHS)

1. We obtained hospitalized RSV associated SARI rates by applying the proportion of RSV positive SARI by age groups for each region as defined in the methods to the regional adjusted rate of hospitalized SARI.

$${IF}_{H,Y}=I_{H,Y} \times F_{Y}$$

Equation 6

Where:

${IF}_{H,Y}$ = Incidence of hospitalized RSV-associated SARI in region Y

$F_{Y}$ = Proportion of SARI due to RSV

Note:

NPOP samples obtained from patients hospitalized with SARI were systematically tested for RSV in Kilifi and Siaya Counties, therefore these represented the Coastal and Nyanza regions respectively. Samples were however intermittently tested for RSV in the other regions; we therefore used data from Siaya County Referral Hospital (CRH) together with Kakamega CRH for the Western region. We used data from Kenyatta National hospital (NH) and Nakuru and Nyeri CRHs to estimate the rates for Rift valley, Central and Nairobi regions. For Eastern and North Eastern regions we used the national average percent positive from all the mentioned health facilities including Coast General Teaching and Referral Hospital (GTRH).

1. We obtained the region specific rates of non-hospitalized RSV-associated SARI by multiplying the incidence of non-hospitalized SARI by the percent positivity for RSV in each region.

$${IF}_{NH,Y}=I_{NH,Y} \times F_{,Y}$$

Equation 7

Where:

${IF}_{NH,Y}$ = Incidence of non-hospitalized RSV-associated SARI in region Y

$F_{Y}$ = Proportion of SARI due to RSV

1. We then obtained the number of hospitalized and non-hospitalized cases of SARI and RSV-associated SARI by multiplying the rates in each region by the county population data from the 2019 census. Since census data were only available in broader age categories, we combined data from Siaya and Kilifi HDSS to build an age structure for every year and applied population proportions to the Census data to obtain fine age bands used in this study.

$${NI}_{H,Y}=I_{H,Y} \times P{op}_{Y}$$

Equation 8

$${NI}_{NH,Y}=I_{NH,Y} \times P{op}_{Y}$$

Equation 9

$${NF}_{H,Y}={IF}_{H,Y} \times P{op}_{Y}$$

Equation 10

$${NF}_{NH,Y}={IF}_{NH,Y} \times P{op}_{Y}$$

Equation 11

Where:

${NI}_{H,Y}$ = Number of hospitalized SARI cases in region Y / County Y

${NI}_{NH,Y}$ = Number of non-hospitalized SARI cases in region Y / County Y

${NF}_{H,Y}$ = Number of hospitalized RSV-associated SARI cases in region Y / County Y

${NF}_{NH,Y}$ = Number of non-hospitalized RSV-associated SARI cases in region Y / County Y

$P{op}_{Y}$ = Population in region Y / County Y
